# Hepatocyte IRE1α–XBP1s axis prevents pro-metastatic niche formation through metabolic rewiring of inflammatory monocytes

**DOI:** 10.1038/s41467-026-78812-2

**Published:** 2026-11-03

**Authors:** Haiyan Zhang, Peibin Liang, Wei Yang, Xiaocong Chen, Yizhen Fu, Chonghao Chen, Zeren Yin, Xiaolin Li, Xin Pei, Josh Haipeng Lei, Xiangpeng Chu, Zhi-Bin Zhao, Hai Hu, Minshan Chen, Hong Pan, Zhe-Xiong Lian, Xiaofan Ding, Nils Halberg, Grant A. Ramm, Jiajie Hou

**Affiliations:** 1 QIMR Berghofer Medical Research Institute, Brisbane, QLD Australia; 2 Cancer Center, Faculty of Health Sciences, University of Macau, Macau, SAR China; 3 State Key Laboratory of Oncology in South China, Collaborative Innovation Center for Cancer Medicine, Sun Yat-sen University Cancer Center, Guangzhou, China; 4 Department of Medical Oncology, Sun Yat-sen University Cancer Center, Guangzhou, China; 5 Guangdong Key Laboratory of Nanomedicine, CAS-HK Joint Lab of Biomaterials, Shenzhen Institutes of Advanced Technology (SIAT), Chinese Academy of Sciences, Shenzhen, China; 6 Department of Liver Surgery, Sun Yat-sen University Cancer Center, Guangzhou, China; 7 Breast Cancer Center, Zhejiang Cancer Hospital, Hangzhou Institute of Medicine, Chinese Academy of Sciences, Hangzhou, China; 8 Guangdong Provincial People’s Hospital (Guangdong Academy of Medical Sciences), Southern Medical University, Guangzhou, China; 9 Faculty of Medicine, The University of Queensland, Brisbane, QLD Australia

**Keywords:** Cancer microenvironment, Hepatocytes, Cancer metabolism

## Abstract

Inositol-requiring enzyme 1α (IRE1α) and the downstream spliced form of X-Box Binding Protein 1 (XBP1s) have been identified as tumor promotors. However, how IRE1α–XBP1s signaling affects tumor progression in a non-cell autonomous manner remains unclear. Here we show that the hepatocyte IRE1α–XBP1s axis unexpectedly inhibits liver metastasis by confining local metainflammation. Genetic ablation of IRE1α or XBP1 in mouse hepatocytes exacerbates liver metastasis due to the accumulation of inflammatory monocytes (IMs). Mechanistically, a non-canonical Forkhead box O1 (FoxO1)-glutamine synthetase (GS) pathway endows the IRE1α- or XBP1-null hepatocytes with the metabolic activity to secrete glutamine, which is necessary and sufficient for IM fitness. Reintroduction of hepatocyte XBP1s or blockade of glutamine metabolism effectively reduces IMs and mitigates disease progression. Importantly, clinical patient survival with liver metastases positively correlates with hepatocyte XBP1s expression. Our study defines a paradigm of hepatocyte–IM interaction that can be leveraged to treat liver metastasis.

## Abstract (web summary)

Targeting the IRE1α–XBP1s stress-sensor axis is widely viewed as a promising anti-tumor strategy. Here, the authors uncover an unexpected hepatocyte-specific role for this pathway in suppressing liver metastasis through metabolic regulation of inflammatory monocytes.

## Introduction

In the tumor microenvironment (TME), stress conditions such as hypoxia and nutrient deprivation may disrupt the protein-folding capacity of the endoplasmic reticulum (ER) in both cancer cells and immune cells. By engaging ER stress sensors and their downstream signaling pathways, these cancer cells can acquire superiority in proliferation and invasion^1^. In contrast, ER-stressed immune cells, which are generally associated with an immunosuppressive TME, facilitate tumor growth and metastasis^2^. Inositol requiring enzyme 1α (IRE1α), the most evolutionarily conserved ER stress sensor, acts in conjugation with its downstream transcription factor, the spliced form of X-Box Binding Protein 1 (XBP1s), evoking a variety of tumor-promoting events in cancer cells^3–5^, dendritic cells^6^, T cells^7^, and macrophages^8^. Although IRE1α also engages the regulated IRE1α-dependent RNA decay (RIDD) pathway, the therapeutic focus in cancer has largely centered on the IRE1α–XBP1s axis. Targeting this axis has been demonstrated as a promising anti-cancer avenue in preclinical mouse models^4,5,9,10^. However, the phase 1/2 trial of ORIN1001, a first-in-class IRE1α inhibitor, has not yielded encouraging results from patients with advanced solid tumors (NCT03950570)^11,12^. This discrepancy highlights the need for further investigation into both the therapeutic potential and unintended consequences of targeting the IRE1α–XBP1s signaling.

The IRE1α–XBP1s axis is a versatile regulator of cellular pathophysiology, acting through both ER stress-dependent and independent mechanisms^13^. In the liver, particularly under high-calorie conditions, this axis has been linked to the development of both steatohepatitis and tumorigenesis^14–18^. Upon exposure to a chemical carcinogen or a high-fat diet, elevated hepatocyte IRE1α expression sensitizes the liver to hepatocellular carcinoma (HCC) development by promoting hepatocyte proliferation via STAT3 activation and exacerbating obesity-associated inflammation through the IKKβ–NF-κB pathway^17^. Clinically, elevated IRE1α expression in HCC tumor tissues correlates with poorer patient survival. In addition, XBP1s enhances HCC cell survival by activating Wnt/β-catenin signaling through both transcriptional regulation and direct interaction with LEF1^19^. However, broad pharmacological inhibition of the IRE1α–XBP1s signaling still raises concerns. For instance, hepatocyte XBP1s is critical for maintaining physiological liver functions such as glucose homeostasis, through transcriptional activation of genes that enhance ER protein-folding capacity or suppression of Forkhead box O1 (FoxO1)-mediated gluconeogenesis^20–24^. In light of its pro-tumoral and pro-inflammatory effects under unresolved ER stress, we extend our investigation of the hepatocyte IRE1α–XBP1s axis to an ER stress-independent context.

The liver is one of the most common sites for metastases^25^. Unlike the pre-existing pathological state of HCC, hepatocytes can be free of ER stress prior to, or during, the early stages of liver metastasis^25^. However, responsive to TME signals, hepatocytes serve as a key component of the pro-metastatic niche. A previous report showed that hepatocytes with acquired STAT3 activation could coordinate myeloid cell accumulation, increasing the susceptibility of the liver to metastatic seeding of disseminated pancreatic cancer cells^26^. Instead, hepatocytes with YAP and TAZ upregulation can trigger regression of melanoma-derived liver metastases through a direct cell competition mechanism^27^. As shown by a recent study using an in vivo cell interaction-based screen approach, hepatocyte-derived plexin B2 enables metastatic seeding of colorectal cancer cells by inducing their epithelialization^28^. In terms of such non-cell autonomous regulations within a pro-metastatic niche, we seek to reexamine the significance of hepatocyte IRE1α–XBP1s signaling under the condition of liver metastasis.

Here in our mouse models, we show that cancer cell lines responsive to IRE1α antagonism during subcutaneous growth acquire resistance to the same treatment upon dissemination to the liver. Correspondingly, we uncover an unexpected protective role of hepatocyte IRE1α–XBP1s signaling, whose loss creates a glutamine-rich TME that fuels inflammatory monocyte (IM) expansion, ultimately exacerbating metastasis. Reintroducing hepatocyte XBP1s or blocking glutamine metabolism effectively reduces IMs and mitigates disease progression. Furthermore, we demonstrate that this protective axis also exists in patients with colorectal cancer liver metastases. In conclusion, hepatocyte IRE1α–XBP1s signaling functions as a metabolic gatekeeper against IM-associated pro-metastatic niche formation.

## Results

### Hepatocyte IRE1α–XBP1s axis suppresses liver metastasis

**Fig. 1:**
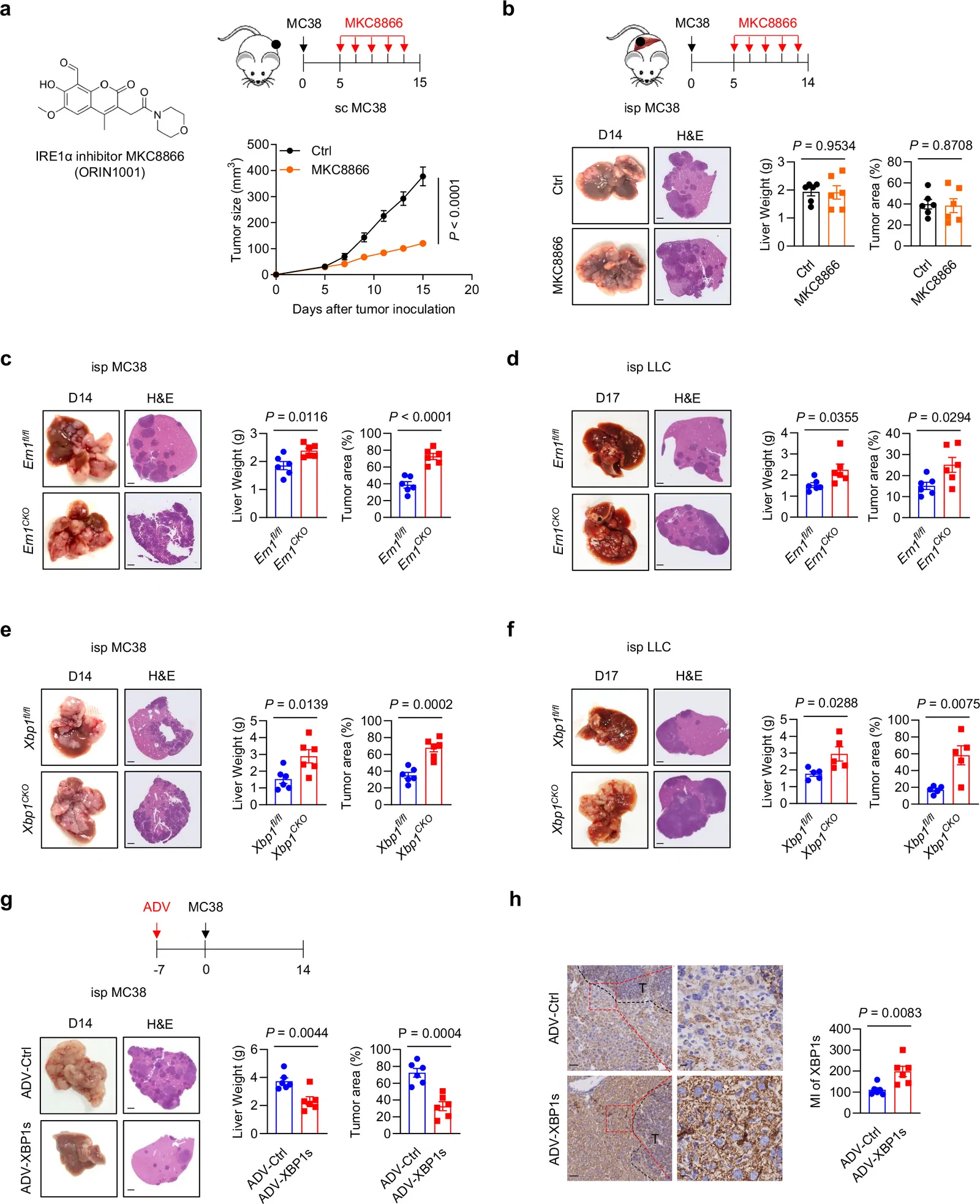
Hepatocyte IRE1α–XBP1s axis constitutes a cancer-inhibitory context in the liver. **a** MKC8866 structure, experimental design, and tumor growth in WT mice after subcutaneous injection (sc) of MC38 cells and treatment with MKC8866 or vehicle (*n* = 6 per group). **b** Liver metastasis in WT mice after intrasplenic injection (isp) of MC38 cells and treatment with MKC8866 or vehicle, showing experimental scheme, liver weight, H&E staining (Scale bars, 1000 μm), and quantification (*n* = 6 per group). **c, d** Liver mass, representative H&E images (Scale bars,1000 μm) and quantification of metastases of MC38 (**c**) or LLC (**d**) -bearing *Ern1*^*CKO*^ and *Ern1*^*fl/fl*^ mice (*n* = 6 per group). **e, f** Liver mass, representative H&E images (Scale bars,1000 μm) and quantification of metastases of MC38 (**e**) or LLC (**f**) -bearing *Xbp1*^*CKO*^ and *Xbp11*^*fl/fl*^ mice (*n* = 6 per group). **g, h** Liver mass, H&E staining (Scale bars,1000 μm) (**g**), and XBP1s immunohistochemistry (**h**) in MC38-bearing WT mice pretreated with XBP1s-overexpressing or control adenoviruses (*n* = 6 per group). Scale bars, 50 μm (h left) or 10 μm (h right). T, tumor. Data are represented as mean ± SEM. Statistical analysis was performed using two-way ANOVA (a) and two-tailed, unpaired Student’s *t* test (**b**–**h**). Some elements in a and b were created by Figdraw (www.figdraw.com). Source data are provided as a Source Data file.

The selective IRE1α RNase inhibitor MKC8866 (ORIN1001) is currently being assessed in phase 1/2 clinical trials for patients with advanced solid tumors (NCT03950570)^11^. We demonstrated that MKC8866 significantly suppresses the growth of subcutaneous tumors formed by colon (MC38) or lung (LLC) adenocarcinoma cells (Fig. 1a and Supplementary Fig. 1a), in agreement with previous reports^4,5,9,10^. To understand how IRE1α-targeted therapeutics affect liver metastases, we subjected wild-type C57BL/6 mice to intrasplenic injection (isp) of MC38 or LLC cells followed by administration of MKC8866 or vehicle. Metastatic burdens were evaluated on day 14 or 17 post isp. Unexpectedly, in MC38-bearing mice, liver weights of the MKC8866-treated group were comparable to those of the vehicle group (Fig. 1b). MKC8866 treatment even slightly increased the liver weights of LLC-bearing mice (Supplementary Fig. 1b). Quantification of metastatic foci on hematoxylin and eosin (H&E)-stained liver sections further confirmed that MKC8866 failed to control liver metastasis (Fig. 1b and Supplementary Fig. 1b). To minimize potential technical bias, we examined a 4T1-based orthotopic breast cancer model that gives rise to spontaneous metastases in multiple sites. As shown in Supplementary Fig.1c, d, treatment with MKC8866 significantly reduced lung metastases but did not confer any therapeutic benefit in the liver. These results suggest that the IRE1α–XBP1s signaling might be harnessed in the liver for non-cell-autonomous tumor suppression, which counteracted its tumor-intrinsic function.

We then conditionally depleted the IRE1α-encoding gene *Ern1* from mouse hepatocytes (*Ern1*^*flox/flox*^;*Alb*^*Cre*^, hereafter referred to as *Ern1*^*CKO*^) and utilized these mice in liver metastasis models. Surprisingly, on day 14 or 17 post isp of MC38 or LLC cells, respectively, the *Ern1*^*CKO*^ mice exhibited larger liver metastasis burdens than did the *Ern1*^*flox/flox*^ (*Ern1*^*fl/fl*^) littermates (Fig. 1c, d). A similar phenotype was observed in mice with hepatocyte-specific deletion of *Xbp1* (*Xbp1*^*CKO*^) (Fig. 1e, f), suggesting that XBP1s is the predominant effector downstream of IRE1α in the context of liver metastasis. To prove that XBP1s dictates tumor suppression in hepatocytes, wild-type and *Ern1*^*CKO*^ mice were intravenously injected with XBP1s-expressing or control adenoviruses 7 days before MC38 inoculation. As shown in Fig. 1g, h and Supplementary Fig. 1e, both the liver weights and the liver areas covered by metastatic lesions were substantially reduced when exogenous XBP1s was introduced in hepatocytes. These results reveal that hepatocyte XBP1s attenuates the intrahepatic outgrowth potential of metastatic cells. Supporting the notion that the MKC8866 resistance observed in Fig.1b arises from disruption of this tumor-suppressive hepatocyte signaling pathway, liver metastases in *Xbp1*^*CKO*^ mice exhibited enhanced sensitivity to the drug (Supplementary Fig. 1f).

### Hepatocyte IRE1α deficiency supports metastatic outgrowth by increasing extracellular glutamine

**Fig. 2:**
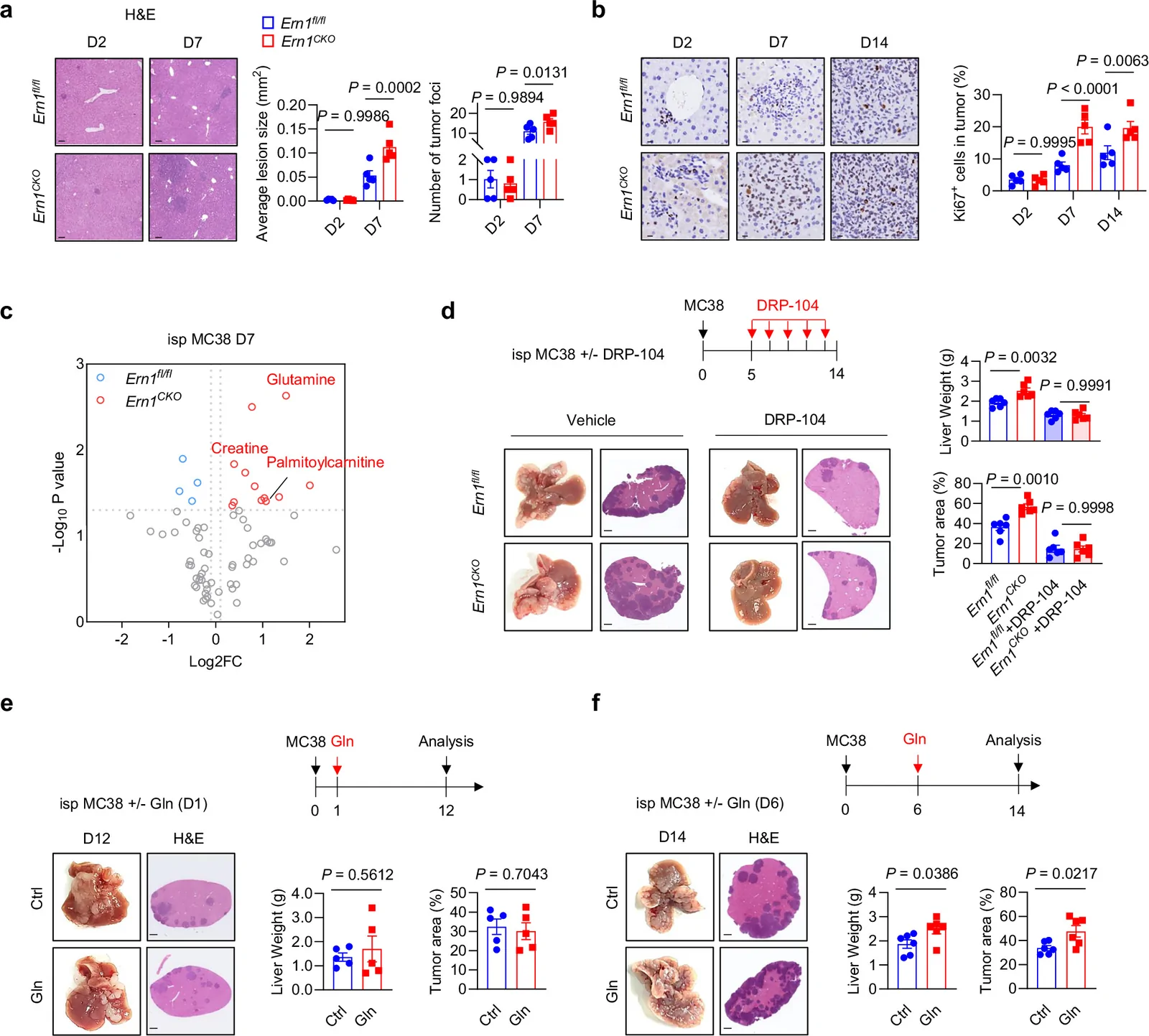
Hepatocyte IRE1α deficiency supports metastatic outgrowth by increasing extracellular glutamine. **a** Representative H&E images and quantification of average lesion size of MC38-bearing *Ern1*^*fl/fl*^ and *Ern1*^*CKO*^ livers collected at indicated timepoints (*n* = 5 per group). Scale bars, 100 μm. **b** Representative images and quantification of Ki67 immunohistochemistry of MC38-bearing *Ern1*^*CKO*^ and *Ern1*^*fl/fl*^ livers collected at indicated timepoints (*n* = 5 per group). Scale bars, 10 μm. **c** Volcano plots depicting differential metabolites in peritumor liver tissues from the indicated groups, as determined by LC–MS analysis (*n* = 3 per group). **d** Liver mass, representative H&E images (Scale bars,1000 μm) and quantification of metastases on MC38-bearing *Ern1*^*CKO*^ and *Ern1*^*fl/fl*^ mice treated with DRP-104 or vehicle control (*n* = 6 per group). **e, f** Liver metastasis in MC38-bearing WT mice supplemented with glutamine or vehicle at day 1 (**e**, *n* = 5 per group) or day 6 (**f**, *n* = 6 per group) post-isp (Scale bars,1000 μm). Data are represented as mean ± SEM and analyzed by two-tailed, unpaired Student’s *t* test. Source data are provided as a Source Data file.

Following extravasation, metastasis-initiating cells colonize the liver and transition from a dormant to a proliferative state, enabling micrometastasis outbreak into macrometastasis^25^. To address the biological basis underlying metastasis progression driven by the loss of hepatocyte IRE1α–XBP1s signaling, we collected tissue samples at timepoints corresponding to initial colonization and intermediate proliferation. As indicated by H&E staining, the numbers of micrometastases were similar between *Ern1*^*fl/fl*^ and *Ern1*^*CKO*^ mice on day 2 post isp; however, by day 7, the metastatic foci were markedly larger in *Ern1*^*CKO*^ mice (Fig. 2a). These findings suggest that hepatic IRE1α loss does not substantially affect the earliest detectable stage of liver colonization but instead promotes subsequent metastatic outgrowth. Next, we evaluated whether tumor cell proliferation was affected in the niches deprived of hepatocyte IRE1α–XBP1s signaling. Consistently, histological analysis revealed increased numbers of Ki67-expressing cells within metastatic foci in *Ern1*^*CKO*^ livers compared with *Ern1*^*fl/fl*^ livers at day 7 and day 14 post isp (Fig.2b). TUNEL staining suggested that the apoptotic cell numbers were comparable between the two groups (Supplementary Fig. 2a). No proliferation or apoptosis was detected in hepatocytes regardless of their genotypes (Supplementary Fig. 2b, c), consistent with findings from an original report of the naïve Ern1^CKO^ mice^29^. Together, these findings suggest that the *Ern1*^*CKO*^ liver provides a microenvironment more permissive for metastatic outgrowth.

Extracellular nutrients generated in the liver may provide survival support to metastatic cells^30^. Given that hepatocyte IRE1–XBP1s signaling regulates liver metabolism, we collected peritumor liver tissues on day 7 post isp of MC38 cells and performed liquid chromatography–mass spectrometry (LC–MS) to investigate whether the loss of hepatocyte IRE1α affects the metabolomic feature of the pro-metastatic niche. Among endogenous energy substrates, several amino acids were detected, with glutamine being the most significantly enriched in the peritumor regions of *Ern1*^*CKO*^ livers compared with *Ern1*^*fl/fl*^ livers (Fig. 2c and Supplementary Data 1). Nonetheless, neither glutamine nor other amino acids were detectable in tumor-free livers from both *Ern1*^*fl/fl*^ and *Ern1*^*CKO*^ livers (Supplementary Data 2). Next, we measured glutamine concentrations in portal vein blood from both tumor-free and metastasis-bearing mice. As shown in Supplementary Fig. 2d, portal vein glutamine concentrations were comparable between tumor-free *Ern1*^*fl/fl*^ and *Ern1*^*CKO*^ mice. Upon liver metastasis formation, portal vein glutamine levels declined in *Ern1*^*fl/fl*^ mice but remained unchanged in *Ern1*^*CKO*^ mice. These results reveal glutamine accumulation and consumption within the metastatic niche, which appears to be largely supported by the loss of hepatocyte IRE1α.

Glutamine metabolism has been extensively implicated in cancer progression^31–33^. Cancer cells encounter energy stress when disseminating to a secondary site, where nutrient flexibility enables their survival for potential metastatic colonization^30,34^. First, to determine the dependency of exacerbated liver metastases on environmental glutamine, we administrated metastasis-bearing *Ern1*^*fl/fl*^ and *Ern1*^*CKO*^ mice with the glutamine antagonist DRP-104 (also known as Sirpiglenastat), which is under clinical evaluation in patients with advanced solid tumors (NCT04471415)^35–37^. As shown in Fig. 2d and Supplementary Fig. 2e, glutamine antagonism abolished the metastasis-supporting effect of IRE1α or XBP1 depletion. To examine the importance of glutamine uptake by cancer cells, we depleted SLC38A2, one of the primary glutamine transporters^33^, from MC38 cells, and verified this reduces their intracellular glutamine level (Supplementary Fig. 2f, g). Histological analyses confirmed that SLC38A2 deficiency inhibited metastasis formation in the liver (Supplementary Fig. 2h); however, microlesions were still observed in *Ern1*^*CKO*^ (but not *Ern1*^*F/F*^) mouse livers (Supplementary Fig. 2i). While liver metastases actively consume and depend on glutamine, the role of excess glutamine in shaping a pro-metastatic niche remains unclear. Therefore, we investigated the effect of glutamine supplementation on liver metastasis through intravenous injection of glutamine or PBS to wild-type mice. In vivo glutamine supplementation during early metastatic seeding (day 1 post isp) did not affect the final liver tumor burden (Fig. 2e). Instead, glutamine supplementation on day 6 post isp significantly accelerated metastatic progression (Supplementary Fig. 2j and Fig. 2f). It is conceivable that metastatic outgrowth induces glutamine consumption, as also reflected by reduced portal vein glutamine levels (Supplementary Fig.2d); its insufficiency, which occurred more likely on day 6 rather than day 1, can be overcome by either hepatic IRE1α depletion or exogenous glutamine supplementation. These findings suggest that hepatic IRE1α deletion promotes liver metastasis by exploiting environmental glutamine availability, an effect that is functional in a context-dependent manner.

### Hepatocyte IRE1α deficiency hijacks inflammatory monocytes to promote metastasis

**Fig. 3:**
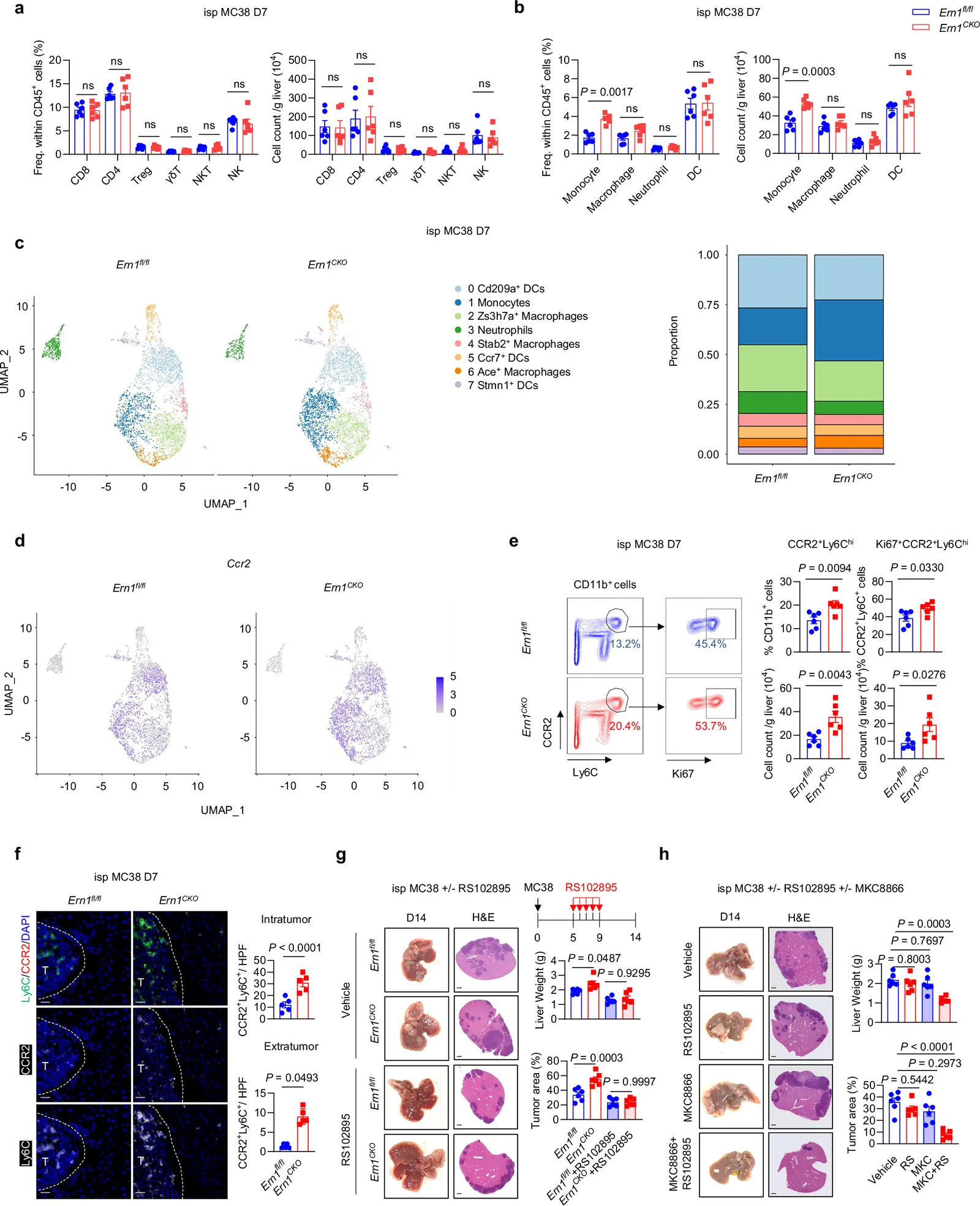
Hepatocyte IRE1α deficiency hijacks inflammatory monocytes to promote metastasis. **a, b** Flow cytometric analysis of lymphoid (**a**) and myeloid (**b**) cell populations within day 7 MC38-bearing *Ern1*^*fl/fl*^ and *Ern1*^*CKO*^ livers (*n* = 6 per group). **c** UMAP visualization (left) and quantification (right) showing subclusters within CD45⁺CD11b⁺CD3⁻ myeloid cells sorted from day 7 MC38-bearing *Ern1*^*fl/fl*^ and *Ern1*^*CKO*^ livers (*n* = 3 per group). **d** UMAP illustrating *Ccr2* expression among subclusters. **e** Flow cytometric analysis of inflammatory monocytes (IMs) in MC38-bearing *Ern1*^*fl/fl*^ and *Ern1*^*CKO*^ mice (*n* = 6 per group). **f** Representative immunofluorescence staining and quantification of CCR2^+^Ly6c^+^ cells in MC38 tumor-bearing *Ern1*^*fl/fl*^ and *Ern1*^*CKO*^ mice. Scale bars, 10 μm (*n* = 6 per group). **g** Liver mass and representative H&E images (Scale bars,1000 μm) and quantification of metastases of MC38-bearing *Ern1*^*fl/fl*^ and *Ern1*^*CKO*^ mice treated with RS102895 or vehicle control (*n* = 6 per group). **h** Liver mass and representative H&E images (Scale bars,1000 μm) and quantification of metastases in MC38-bearing *Ern1*^fl/fl^ and *Ern1*^CKO^ mice receiving vehicle, RS102895, MKC8866, or combination treatment (*n* = 6 per group). Data are represented as mean ± SEM. Statistical analysis was performed using one-way ANOVA (**a**, **b**, **g**, **h**) and two-tailed, unpaired Student’s *t* test (**e**, **f**). Source data are provided as a Source Data file.

The above observations prompted us to focus on the intermediate phase of liver metastasis (day 7), when cancer cells grow from microlesions into macrometastases^38,39^. We asked whether the immune composition of the pro-metastatic niche at this timepoint is altered by hepatic IRE1α deletion. Flow cytometry analyses of liver-infiltrating immune cells showed that the lymphoid compartment was similar between the metastasis-bearing *Ern1*^*fl/fl*^ and *Ern1*^*CKO*^ livers, however, the frequencies and numbers of CD11b^+^Ly6C^hi^ monocytes were greater in *Ern1*^*CKO*^ livers than in *Ern1*^*fl/fl*^ livers (Fig. 3a, b and Supplementary Fig. 3a). Other myeloid populations (CD11b^+^F4/80^+^Ly6C^-/lo^ macrophages, CD11b^+^CD11c^+^ dendritic cells, and CD11b^+^Ly6G^+^ neutrophils) were unaffected by hepatocyte IRE1α depletion (Fig. 3b and Supplementary Fig. 3b).

Next, we examined the relevance of natural killer (NK) or CD8 T cell-mediated immunosurveillance to the differential metastatic burdens by administering *Ern1*^*fl/fl*^ and *Ern1*^*CKO*^ mice with an anti-NK1.1 or anti-CD8 neutralizing antibody. Depletion of NK or CD8 T cells did not perturb the difference between *Ern1*^*fl/fl*^ and *Ern1*^*CKO*^ mice (Supplementary Fig. 3c–e). This excludes the possibility that the superior tumor growth in the *Ern1*^*CKO*^ liver was a result of impaired cancer immunosurveillance. Instead, an anti-Ly6C antibody that deleted circulating monocytes markedly abrogated the increase of metastatic burden caused by hepatocyte IRE1α depletion (Supplementary Fig. 3f), demonstrating that monocytes are essential components of the pro-metastatic niche.

To dissect the myeloid compartment regulated by IRE1α-deficient hepatocytes, we sorted CD11b^+^ liver-infiltrating immune cells from *Ern1*^*fl/fl*^ and *Ern1*^*CKO*^ mice on day 7 post isp of MC38 cells and performed single-cell RNA sequencing (scRNA-seq). Unbiased profiling of 7,515 single cells (*Ern1*^*fl/fl*^: 3,715; *Ern1*^*CKO*^: 3,800) produced a robust 8-cluster resolution irrespective of genotype, defining 1 cluster (C1) for monocytes, 1 cluster (C3) for neutrophils, 3 clusters (C2, 4, and 6) for macrophages, and 3 cluster (C5, 7, and 0) for dendritic cells (DCs) (Fig. 3c and Supplementary Fig. 4a, b). Trajectory analysis predicted that cluster 1 cells retained the potential to differentiate into either macrophages or DCs, but this trend was not affected by hepatocyte IRE1α deficiency (Supplementary Fig. 4c). Consistent with flow cytometry analysis, hepatocyte IRE1α deficiency resulted in a prominent increase in the cluster 1 subpopulation (Fig. 3c). Inflammatory monocytes (IMs), which highly express CCR2 (the receptor for chemokine CCL2), as well as monocyte-derived macrophages are considered fundamental for pro-metastatic niche formation through their interaction with metastasizing tumor cells^38,40^. Of note, clusters 0, 1, and 6 cells were enriched for *Ccr2* expression, with cluster 1 dominating in cell quantity and *Ccr2* intensity (Fig. 3d). Cluster 1 cells also expressed high levels of lineage markers (*Ly6c2*, *Ly6i*, *Cd14*, etc) and pro-inflammatory molecules (*C1qa*, *C1qb*, *C1qc*, *Mif*, *Ccl2*, etc)^38,40–42^ (Supplementary Fig. 4d), which characterize them as cancer-promoting IMs in this context. Although per-cell expression of pro-inflammatory genes was comparable between *Ern1*^*fl/fl*^ and *Ern1*^*CKO*^ groups, both E2F target genes and ATP synthesis pathway genes were significantly upregulated in the *Ern1*^*CKO*^ group (Supplementary Fig. 4e), enabling active expansion of IMs.

Flow cytometry analysis revealed similarly low levels of monocyte infiltration in tumor-free *Ern1*^*fl/fl*^ and *Ern1*^*CKO*^ livers (Supplementary Fig. 5a). The percentages of CD11b^+^ myeloid cells and CCR2^+^ IMs peaked at day 2 and declined thereafter (Supplementary Fig. 5b), which might be owing to the resolution of acute inflammation. Interestingly, the proportion of CCR2^+^ IMs increased at day 7, indicative of disease-associated inflammation (Supplementary Fig. 5b). These cells not only enhanced cancer cell proliferation in an ex vivo co-culture assay but also promoted metastatic progression in an adoptive transfer model (Supplementary Fig. 5c, d). Importantly, both *Ern1*^*CKO*^ and *Xbp1*^*CKO*^ livers exhibited a marked increase in CCR2^+^ IMs at day 7 (but not day 2) compared with their counterparts (Fig.3e and Supplementary Fig. 5e–g). Meanwhile, immunofluorescence staining confirmed the enrichment of these cells in *Ern1*^*CKO*^ livers at tumor edges, indicative of a disease-accompanying pattern (Fig. 3f). As a potential explanation for the therapeutic failure of IRE1α antagonism, treatment with MKC8866 led to the accumulation of CCR2^+^ IMs in the wild-type metastasis-bearing liver (Supplementary Fig. 5h). Supporting their essential role in the pro-metastatic niche, a CCR2 antagonist RS102895 was sufficient to abolish the progression difference between *Ern1*^*fl/fl*^ and *Ern1*^*CKO*^ (or *Xbp1*^*fl/fl*^ and *Xbp1*^*CKO*^) livers as well as to resensitize liver metastases to IRE1α antagonism in wild-type mice (Fig. 3g, h and Supplementary Fig. 3g, h). To confirm that CCR2^+^ IMs were differentially expanded in response to microenvironmental cues, Ly6C^+^ cells were isolated from the bone marrow of wild-type mice, labeled in vitro with CellTrace Violet (CTV), and adoptively transferred into *Xbp1*^*fl/fl*^ and *Xbp1*^*CKO*^ mice on day 6. Remarkably, when traced 48 h later, a 3-fold greater proportion of CTV^+^Ki67^+^CCR2^+^ monocytes were found within the *Xbp1*^*CKO*^ liver relative to the *Xbp1*^*fl/fl*^ counterpart (Supplementary Fig. 5i). These findings suggest that hepatocyte IRE1α or XBP1 deficiency fosters a pro-metastatic niche where monocytes can sustain a cancer-promoting inflammatory state.

### Glutamine released from IRE1α or XBP1-deficient hepatocytes facilitates monocyte expansion

**Fig. 4:**
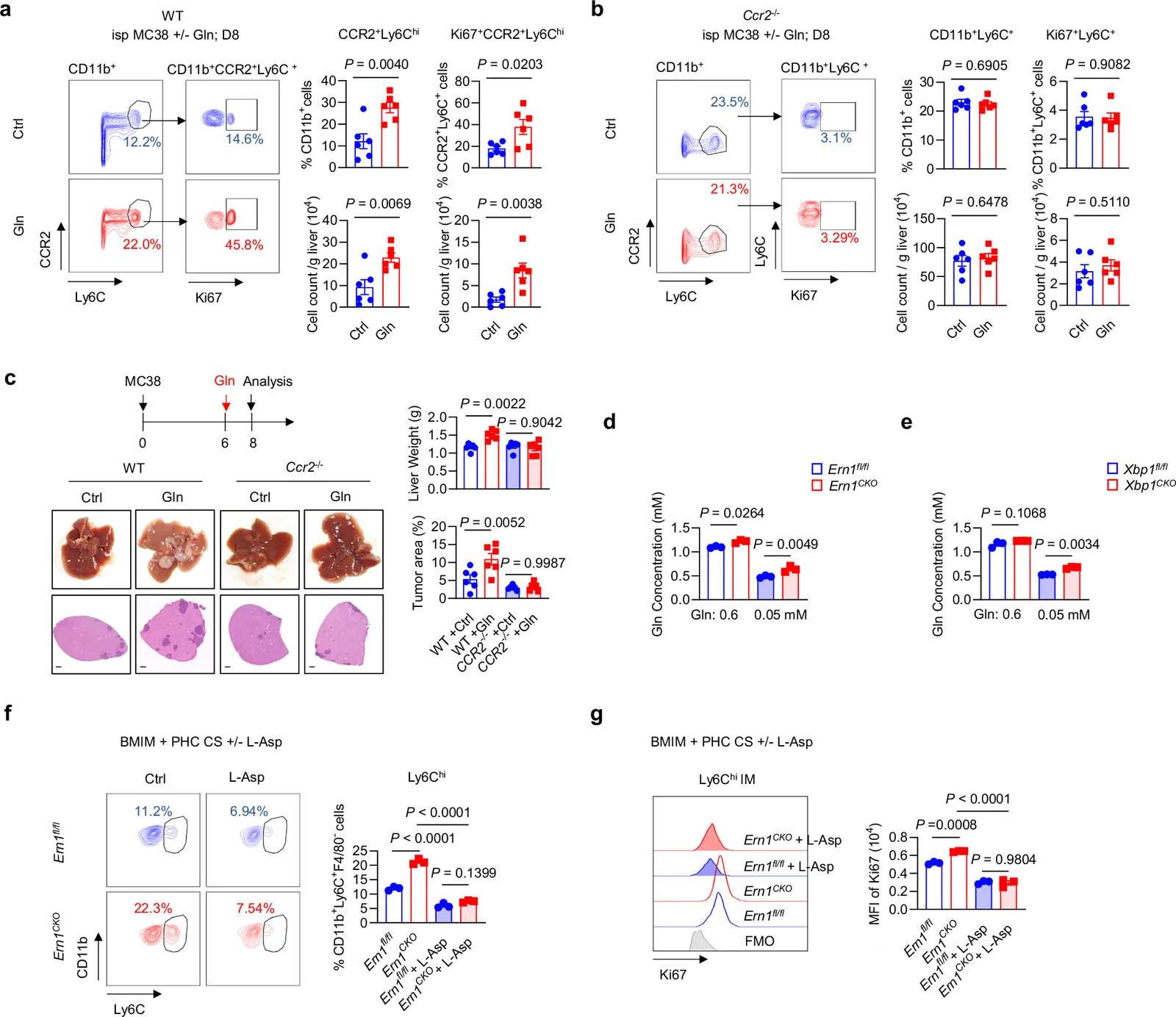
Glutamine released from IRE1α or XBP1-deficient hepatocytes facilitates monocyte expansion. **a** Flow cytometric analysis of IMs in MC38-bearing WT mice with or without glutamine supplementation (*n* = 6 per group). **b, c** Flow cytometric analysis (**b**) and quantification of metastases (**c**) in MC38-bearing *Ccr2*^-/-^ mice with or without glutamine supplementation (*n* = 6 per group). Scale bars,1000 μm). **d, e** Glutamine levels in hepatocyte supernatants from *Ern1*^CKO^ (**d**) and *Xbp1*^CKO^ (**e**) or control mice under indicated conditions (*n* = 3 per group). **f, g **Flow cytometric analysis of BMIMs stimulated by conditioned medium from *Ern1*^*fl/fl*^ or *Ern1*^*CKO*^ hepatocytes with or without L-asparaginase (L-Asp), showing Ly6C^hi^ percentages (**f**) and Ki67 MFI (**g**) (*n* = 3 per group). The left plots show CD11b^+^Ly6C^+^F4/80^-^ cells. Data are represented as mean ± SEM. Statistical analysis was performed using two-tailed, unpaired Student’s *t* test (**a**, **b**) and one-way ANOVA (**c**–**g**). Source data are provided as a Source Data file.

Based on the above findings, we inferred that intrahepatic glutamine might be responsible for IM fitness during metastasis formation. To examine this causative link, we revisited the above glutamine-treated liver metastasis-bearing mice. Potentially due to a glutamine-saturated microenvironment at the early timepoint, IMs displayed a highly proliferative state (Supplementary Fig.5e). This may explain why early glutamine supplementation failed to exacerbate liver metastasis (Fig.2e). At the intermediate stage, decreased portal vein glutamine levels in IRE1α-competent mice indicate a relative insufficiency of glutamine within the liver (Supplementary Fig.2d). Glutamine supplementation around this timepoint markedly increased the percentage and number of CCR2^+^ IMs within the tumor-bearing liver (Fig. 4a), consistent with the enhanced metastasis phenotype (Supplementary Fig.2j and Fig. 2f). Conversely, to test whether extracellular glutamine relies on IMs to facilitate liver metastasis, we regenerated the liver metastasis model using *Ccr2*^-/-^ mice, in which monocytes are unable to be recruited from bone marrow^43^. As shown in Fig. 4b, c, glutamine supplementation failed to promote both myeloid cell expansion and metastatic progression in the liver, thereby reinforcing the role of the glutamine–IM axis in establishing a pro-metastatic niche.

To investigate whether IRE1α deletion directly affects glutamine production by hepatocytes, we isolated primary hepatocytes from *Ern1*^*fl/fl*^ and *Ern1*^*CKO*^ mice and cultured them either in medium supplemented with a physiological concentration (0.6 mM) of glutamine or under glutamine-restricted condition (0.05 mM). Notably, after 24 h of culture under either condition, the glutamine concentration in the hepatocyte supernatant exceeded that of the corresponding medium, suggesting that hepatocytes are capable of releasing glutamine to the extracellular space (Fig. 4d). Under glutamine-restricted conditions—metabolically reflective of the liver metastasis context—the glutamine concentration in the *Ern1*^*CKO*^ cell supernatant was 30% higher than that in the *Ern1*^*fl/fl*^ cell supernatant (Fig. 4d). Elevated glutamine production could also be recapitulated in the supernatant of *Xbp1*^*CKO*^ hepatocytes (Fig. 4e). Moreover, when infected with XBP1s-expressing adenoviruses, these cells again showed decreased glutamine secretion (Supplementary Fig. 6a). Surprisingly, a mutant form of XBP1s which lacks the DNA-binding domain (ΔDBD)^21^ was also able to diminish glutamine production (Supplementary Fig. 6a), defining this metabolic restriction as a transcription-independent effect of XBP1s.

Next, we sought to establish a bone marrow-derived IM (BMIM) culture system by stimulating BM cells with macrophage colony-stimulating factor (M-CSF) for 5 days. Conditioned medium from *Ern1*^*CKO*^ hepatocytes more significantly promoted the expansion of CD11b^+^F4/80^-^Ly6C^hi^ monocytes (but not CD11b^+^F4/80^+^ macrophages), relevant to conditioned medium from *Ern1*^*fl/fl*^ hepatocytes (Fig. 4f, g and Supplementary Fig. 6b, c). To ascertain that IM fitness depends on hepatocyte-derived glutamine, we utilized L-asparaginase to hydrolyze glutamine in the above media^44^. As expected, L-asparaginase diminished the inflammatory response conferred by *Ern1*^*CKO*^ hepatocytes-conditioned medium (Fig. 4f, g). Therefore, we demonstrated that extracellular glutamine derived from IRE1α or XBP1-deficient hepatocytes is a key driver of monocyte transitioning into an inflammatory state.

### Monocytes depend on glutamine to sustain their pro-inflammatory expansion

**Fig. 5:**
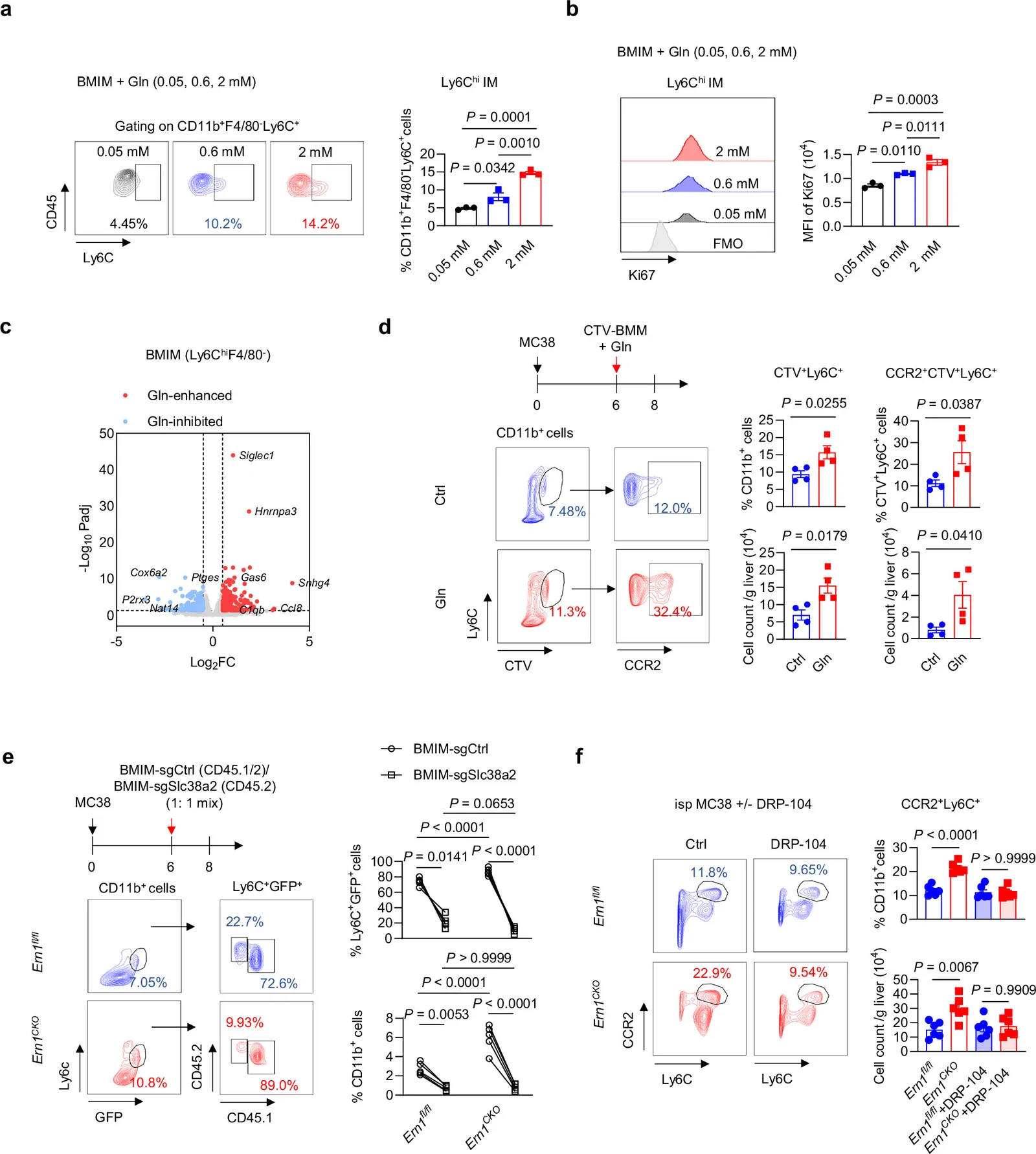
Monocytes depend on glutamine to sustain their pro-inflammatory expansion. **a, b** Flow cytometric analysis of BMIMs exposed to indicated concentrations of glutamine, showing Ly6C^hi^ percentages (**a**) and Ki67 MFI (**b**) (*n* = 3 per group). **c** Volcano plots depicting differentially expressed genes in Ly6C^hi^ F4/80^-^ monocytes cultured under glutamine-replete (0.6 mM) versus glutamine-restricted (0.05 mM) conditions. **d** Flow cytometric analysis of CTV-labeled monocytes that were transferred into MC38-bearing WT mice, with or without concurrent glutamine supplementation (n = 4 per group). **e** Co-transfer of BMIM-sgCtrl (CD45.1/2) and BMIM-sgSlc38a2(CD45.2) cells (1:1 mix) into MC38 metastasis-bearing *Ern1*^*fl/fl*^ and *Ern1*^*CKO*^ mice, showing the experimental scheme, representative flow cytometric plots, and quantification of Ly6C^+^GFP^+^ IMs in the liver (*n* = 5 per group). **f** Flow cytometric analysis of IMs in MC38-bearing *Ern1*^*fl/fl*^ and *Ern1*^*CKO*^ mice that were treated with DRP-104 (*n* = 5 per group). Data are represented as mean ± SEM. Statistical analysis was performed using one-way ANOVA (**a**, **b**, and **e**) and two-tailed, unpaired Student’s *t* test (**d**, **f**). Source data are provided as a Source Data file.

To corroborate that glutamine directly impacts monocyte function, we exposed BMIMs to different concentrations of glutamine ex vivo. While a physiological (0.6 mM) or high (2 mM) level of glutamine enabled BMIM induction, glutamine restriction (0.05 mM) substantially decreased monocyte expansion (Fig. 5a, b), implying an indispensable role for glutamine in sustaining IM fitness. Possibly because pro-inflammatory macrophages use glucose as a major energy source^45^, glutamine restriction did not impair the proliferative capacity of more differentiated CD11b^+^F4/80^+^Ly6C^+^ macrophages (Supplementary Fig. 6d). To characterize the glutamine-dependent inflammatory traits, we analyzed the transcriptome data of monocytes and macrophages that were cultured under glutamine-replete vs. -restricted condition. As shown by Fig. 5c, Supplementary Fig. 6g and Supplementary Data 3, monocytes but not macrophages relied on glutamine to express pro-inflammatory genes (e.g., *Siglec1*, *C1qb*, and *Ccl8*)^46^, showing consistency with IMs from the *Ern1*^*CKO*^ liver metastasis niche. Additionally, CTV-labeled monocytes were transferred to wild-type tumor-bearing mice with or without simultaneous glutamine supplementation. Flow cytometry showed that glutamine supplementation was sufficient to induce a CCR2⁺ phenotype in exogenous monocytes (Fig. 5d), reminiscent of our observations from *Ern1*^*CKO*^ mice.

Subsequently, we asked whether the turnover of IM in the *Ern1*^*CKO*^ liver is fully due to extra glutamine consumption. Among the known glutamine transporters, SLC38A2 is preferentially expressed in both mouse and human monocytes^33^, so we sought to evaluate IM fitness in the context of SLC38A2 deficiency. As anticipated, BMIMs lacking SLC38A2 expression exhibited impaired glutamine consumption and expansion in vitro (Supplementary Fig. 6e, f). Moreover, we transferred congenic BMIMs harboring either an sgSlc38a2 construct or a control Cas9 vector to *Ern1*^*fl/fl*^ and *Ern1*^*CKO*^ tumor-bearing mice (Fig. 5e). When traced 48 h later, SLC38A2-deficient BMIMs were accumulated in the liver at markedly lower frequencies than control BMIMs, a difference that was further amplified in *Ern1*^*CKO*^ recipients. In addition, SLC38A2 deficiency abolished the difference in BMIM frequencies between *Ern1*^*fl/fl*^ and *Ern1*^*CKO*^ livers (Fig. 5e). In keeping with this, treatment with the glutamine antagonist DRP-104 also equalized the abundance of endogenous IMs between *Ern1*^*fl/fl*^ and *Ern1*^*CKO*^ (or *Xbp1*^*fl/fl*^ and *Xbp1*^*CKO*^) livers (Fig. 5f and Supplementary Fig. 6h). These results have highlighted extracellular glutamine as the pivotal mediator for IM expansion conferred by the loss of hepatocyte IRE1α–XBP1 signaling.

### IRE1α–XBP1s signaling suppresses hepatocyte glutamine production through regulation of the FoxO1–GS pathway

**Fig. 6:**
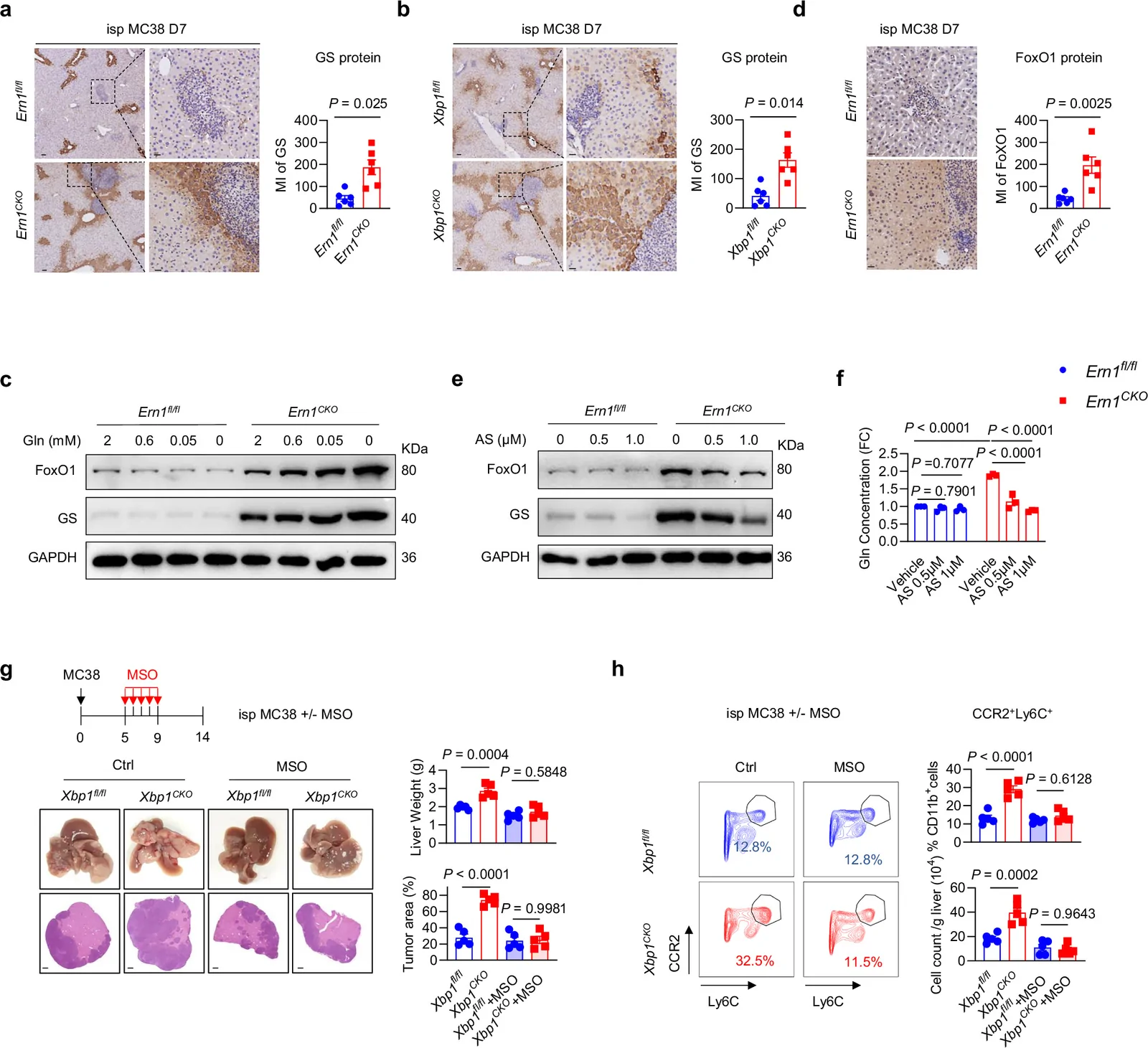
IRE1α–XBP1s signaling suppresses hepatocyte glutamine production through regulation of the FoxO1–GS pathway. **a, b** Representative GS immunohistochemistry images of day 7 MC38-bearing *Ern1*^*fl/fl*^ vs. *Ern1*^*CKO*^ (a) or *Xbp1*^*fl/fl*^ vs. *Xbp1*^*CKO*^ livers (**b**) (*n* = 6 per group). Scale bars, 50 μm (left) or 20 μm (right). Zone 3 GS expression was excluded for quantification. **c** Immunoblotting analysis of *Ern1*^*fl/fl*^ and *Ern1*^*CKO*^ hepatocytes cultured under indicated concentrations of glutamine. The samples derive from the same experiment but different gels for FoxO1 and GAPDH, another for GS was processed in parallel. The similar strategy applies to (**e**). Representative results from three independent experiments are shown. **d** Representative FoxO1 immunohistochemistry images of day 7 MC38-bearing *Ern1*^*fl/fl*^ vs. *Ern1*^*CKO*^ livers (*n* = 6 per group). Scale bars, 50 μm (left) or 20 μm (right). **e, f** Immunoblotting analysis (**e**) and measurement of supernatant glutamine levels (**f**) in *Ern1*^*fl/fl*^ and *Ern1*^*CKO*^ hepatocyte treated with the indicated concentrations of AS1842856 (*n* = 3 per group). Representative results from three independent experiments are shown. **g, h** Metastatic tumor burden (**g**) and flow cytometric analysis of IMs (**h**) in MC38-bearing *Ern1*^*fl/fl*^ and *Ern1*^*CKO*^ mice that were treated with MSO (*n* = 5 per group). Scale bars,1000 μm. Data are represented as mean ± SEM. Statistical analysis was performed using two-tailed, unpaired Student’s *t* test (**a**, **b**, **d**) and one-way ANOVA (**f**–**h**). Source data are provided as a Source Data file.

We next sought to elucidate the molecular mechanism through which hepatocyte IRE1α–XBP1s signaling regulates glutamine production. Glutamine is synthesized by the enzyme glutamine synthetase (GS), which is selectively expressed by hepatocytes surrounding the central veins (zone 3 hepatocytes) under physiological conditions^47^. Immunohistochemistry confirmed that hepatocytes are the primary GS-positive cells in the liver, with only a negligible fraction of metastatic cells exhibiting GS expression (Fig. 6a). Importantly, in addition to zone 3 hepatocytes, which highly expressed GS regardless of genotypes, *Ern1*^*CKO*^ (but not *Ern1*^*fl/fl*^) hepatocytes proximal to the metastatic foci were also enriched for GS expression (Fig. 6a). This enhanced GS expression pattern was not seen in the tumor-free *Ern1*^*CKO*^ liver (Supplementary Fig. 7a). Supporting the regulatory role of the IRE1α–XBP1s axis, tumor-adjacent hepatocytes deficient in XBP1 exhibited a significant increase in GS expression, whereas those overexpressing XBP1s showed a marked decrease (Fig. 6b and Supplementary Fig. 7b, c). As validated by immunoblotting, in vitro-cultured *Ern1*^*CKO*^ mouse hepatocytes exhibited a greater GS protein level compared to *Ern1*^*fl/fl*^ hepatocytes (Fig. 6c). When glutamine was added to the culture medium in gradually decreasing doses, GS expression remained stable in *Ern1*^*fl/fl*^ hepatocytes. By contrast, *Ern1*^*CKO*^ hepatocytes exhibited increased GS expression in response to decreasing glutamine levels (Fig. 6c), consistent with the corresponding liver metastasis niche, where nutrient uptake by cancer cells underpinned this compensatory metabolic phenotype.

A previous report has shown that the transcription factor FoxO1 can directly activate the transcription of GS-encoding gen *Glul*^48^. Since XBP1s was known to guide FoxO1 degradation independently of ER stress^21^, we inferred that the upregulated GS expression in the absence of IRE1α–XBP1 signaling is due to accumulated FoxO1. As shown by Fig. 6c and Supplementary Fig. 7d, the FoxO1 protein expression level was significantly higher in *Ern1*^*CKO*^ and *Xbp1*^*CKO*^ hepatocytes than that in *Ern1*^*fl/fl*^ and *Xbp1*^*fl/fl*^ hepatocytes, respectively. Of note, in the absence of IRE1α or XBP1, FoxO1 expression was further upregulated as glutamine concentrations declined. However, this trend was not observed upon treatment with the ER stress inducer tunicamycin (Tm) (Supplementary Fig. 7e). Immunohistochemistry showed that FoxO1 protein was predominantly localized to tumor-adjacent hepatocytes in *Ern1*^*CKO*^ livers, but not to zone 3 hepatocytes (Fig. 6d), consistent with the newly acquired GS expression pattern. Supporting a causal relationship between FoxO1 and GS, treatment with increasing doses of the FoxO1 inhibitor AS1842856^49^ progressively suppressed GS expression and glutamine production in both *Ern1*^*CKO*^ and *Xbp1*^*CKO*^ hepatocytes (Fig. 6e, f and Supplementary Fig. 7f, g). To confirm that XBP1s restricts GS expression, we transfected *Xbp1*^*CKO*^ hepatocytes with XBP1s- or XBP1s-ΔDBD-expressing adenoviruses. In concert with their capability to diminish glutamine production (Supplementary Fig. 6a), both forms of XBP1s were able to reduce GS expression (Supplementary Fig. 7h). As reported^21^, XBP1s could interact with FoxO1 independently of its DNA-binding activity (Supplementary Fig. 7i). Taken together, these results have demonstrated that the hepatocyte IRE1α–XBP1s signaling suppresses glutamine synthesis through inactivation of the FoxO1–GS pathway.

To ascertain whether GS overexpression is responsible for the pro-inflammatory and pro-metastatic properties of hepatocytes lacking IRE1α–XBP1 signaling, we administered the GS inhibitor methionine sulfoximine (MSO) to tumor-bearing *Xbp1*^*fl/fl*^ and *Xbp1*^*CKO*^ mice. GS inhibition abrogated the difference in liver metastatic burden between *Xbp1*^*fl/fl*^ and *Xbp1*^*CKO*^ mice, effectively eliminating the pro-metastatic capacity of XBP1-deficient hepatocytes (Fig. 6g and Supplementary Fig. 7j). As shown by flow cytometry and immunohistochemistry analyses, GS inhibition rectified the increased accumulation of IMs in the *Xbp1*^*CKO*^ mouse liver (Fig. 6h and Supplementary Fig. 7k), an effect similar to that which was induced by DRP-104 (Fig. 5f). Thus, these findings suggest that hepatocyte GS plays a fundamental role in IRE1α/XBP1 depletion-induced local “metainflammation” and pro-metastatic niche formation.

### Hepatocyte IRE1α–XBP1s signaling impede IM expansion in mouse and human liver metastases

**Fig. 7:**
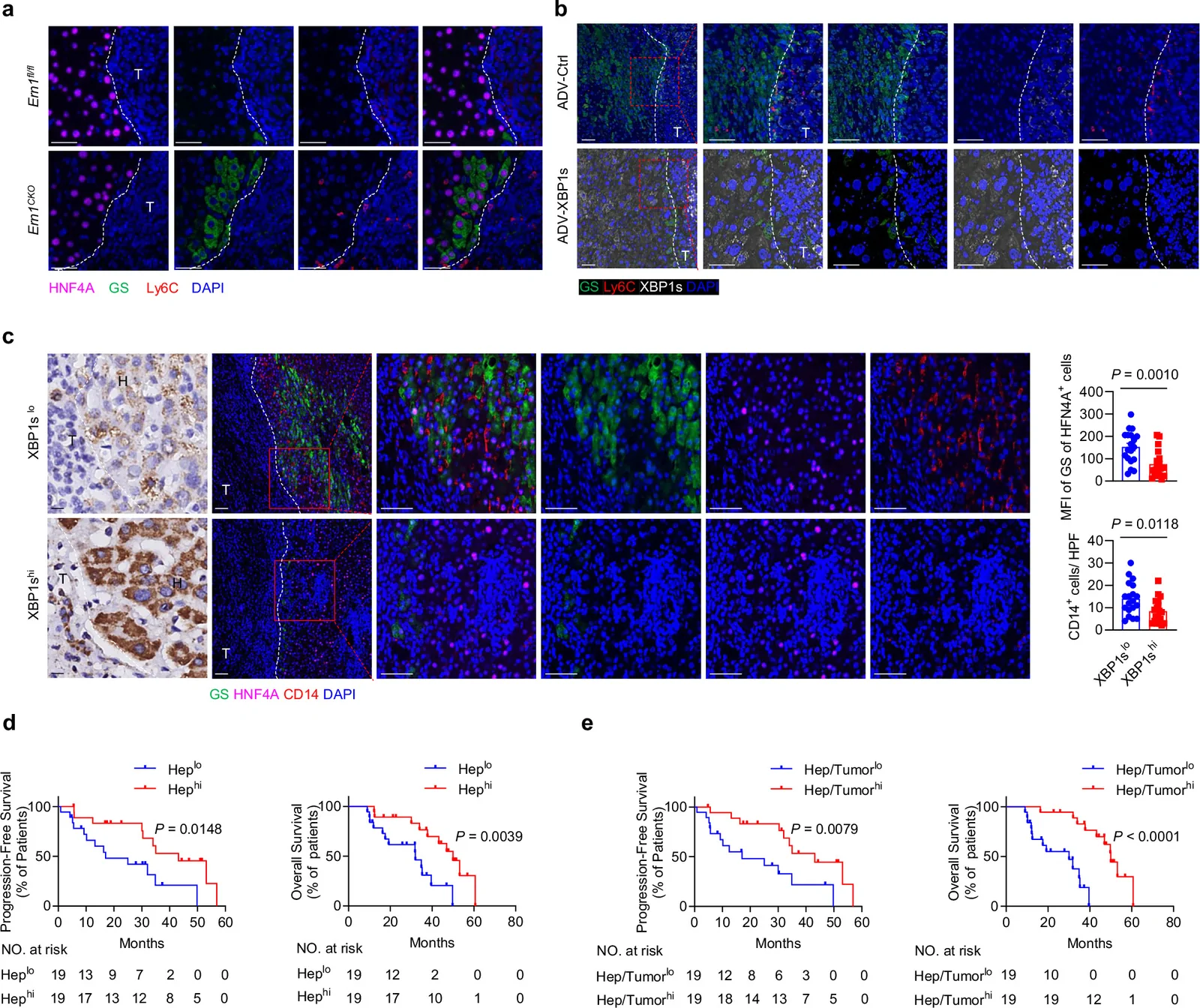
Hepatocyte IRE1α–XBP1s signaling impede IM expansion in mouse and human liver metastases. **a, b** Representative multiplex immunofluorescence images of day 7 MC38-bearing livers from *Ern1*^*fl/fl*^ and *Ern1*^*CKO*^ mice (**a**) or *Ern1*^*CKO*^ mice pretreated with XBP1s-overexpressing or control adenoviruses (**b**). Scale bars, 50 μm. T, tumor. *n* = 6 per group. **c** Representative immunohistochemistry and multiplex immunofluorescence images of tumor-adjacent liver tissue from CRLM patients (left) and quantitative comparison (right) based on XBP1s expression (XBP1s^hi^ vs XBP1s^lo^, *n* = 19 per group). Scale bars, 10 μm for IHC or 50 μm for IF. T, tumor; H, hepatocytes. **d, e** Kaplan–Meier analysis of progression-free survival and overall survival in 38 CRLM patients based on XBP1s expression in adjacent liver tissue (**d**) or its liver-to-tumor expression ratio (*n* = 19 per group) (**e**). Data are represented as mean ± SEM. Statistical analysis was performed using two-tailed, unpaired Student’s *t* test (**c**) and log rank test (**d**, **e**). Source data are provided as a Source Data file.

To explore the hepatocyte-IM communication in the pro-metastatic niche, we performed multiplex immunofluorescence staining on mouse liver sections, using an anti-HNF4A antibody^50^ to visualize hepatocytes. In *Ern1*^*CKO*^ and *Xbp1*^*CKO*^ livers, metastatic foci were adjacent to hepatocytes expressing abundant GS, with the invasive margins highly infiltrated with Ly6C^+^ monocytes (Fig. 7a and Supplementary Fig. 8a, c, d). Nonetheless, GS-expressing peritumoral hepatocytes and proximally infiltrating monocytes were less present in *Ern1*^*fl/fl*^ and *Xbp1*^*fl/fl*^ livers (Fig. 7a and Supplementary Fig. 8a, c, d). By contrast, in adenovirus-treated mice, monocyte infiltration was prominent within metastatic lesions adjacent to XBP1s^-/low^GS⁺ hepatocytes but notably absent from areas proximal to XBP1s^+^GS⁻ hepatocytes (Fig. 7b and Supplementary Fig. 8b, e, f). Thus, these results support the proposal that the hepatocyte IRE1α–XBP1s axis effectively curtails the GS-IM signaling in the liver metastasis niche.

Finally, we conducted immunohistochemistry and multiplex immunofluorescence staining on liver sections from colorectal cancer liver metastasis (CRLM) patients who had undergone neoadjuvant chemotherapy followed by radical surgery. XBP1s protein expression in hepatocytes was heterogeneous across patients but appeared modestly elevated and more uniformly distributed within tumor regions (Supplementary Fig. 8g). In tumor-adjacent hepatocytes, higher XBP1s expression was associated with lower GS expression, accompanied by less infiltration of CD14⁺ monocytes at the invasive margins (Fig. 7c). Moreover, patients with higher XBP1s levels in adjacent liver tissue or lower levels in tumor tissue exhibited reduced recurrence or improved survival—those with the highest liver-to-tumor ratio of XBP1s expression demonstrated the most favorable prognosis (Fig. 7d, e and Supplementary Fig. 8h).

Taken together, these findings demonstrate that the hepatocyte IRE1α–XBP1s axis exerts a tumor-suppressive function by disrupting the monocyte-instructed pro-metastatic niche.

## Discussion

Liver metastases occur in up to 80% of pancreatic cancer patients and 50% of colorectal cancer patients, and are also frequently observed in melanoma, lung cancer, breast cancer, and other malignancies—representing a refractory disease state associated with poor survival outcomes^25^. Despite the anatomical trapping of circulating cancer cells, liver metastases also depend, at least in part, on the formation of “pro-metastatic” niches. IMs—characterized by high Ly6C expression in mice or CD14 expression in humans, together with CCR2 positivity—are preferentially recruited to the pre-metastatic lung or liver, mediating tumor cell extravasation and pro-metastatic niche formation^38,40^. Although IM-derived macrophages share the pro-metastatic functions, IMs themselves are essential for driving early-stage metastasis in the presence of active immune surveillance. Recent clinicopathological evidence suggests that therapy resistance in patients with CRLM is associated with both the spatial proximity of tumor cells and hepatocytes and the abundance of intrahepatic monocytes^51^. Extrahepatic tumor-derived cues can induce hepatocytes, the major parenchymal cells of the liver, to adopt a pro-inflammatory, IM-supporting phenotype^26^; however, it was unclear whether unaffected hepatocytes retain functions that counteract IM accumulation. In this study, we uncover an anti-metastatic function of the hepatocyte IRE1α–XBP1s axis in mice, that restricts glutamine supply and thereby limits IM expansion and disease progression. Notably, in tumor-adjacent liver tissues from patients with CRLM, elevated XBP1s protein levels are associated with less IM infiltration and longer survival.

Extracellular nutrients are highly demanded for cancer cell survival during early metastatic colonization^34^. While a high glycolytic rate is essential for cancer cells metastasizing to the liver, access to additional energy sources can further enhance their colonization of the hepatic niche^52,53^. For instance, colorectal cancer cells exploit circulating creatine and hepatic phosphocreatine to support their metastatic potential^30,54^. In our mouse model, loss of hepatocyte IRE1α leads to glutamine enrichment within the liver metastatic niche. Despite evidence that glutamine serves as an alternative energy source in other metastatic sites^53^, its role in liver metastasis has not been reported. Here we demonstrate that glutamine is essential for intrahepatic metastatic cell outgrowth—the glutamine antagonist DRP-104 markedly inhibits liver metastasis formation. As glutaminolysis underpins cancer cell invasiveness^55^, MC38 cells lacking the glutamine transporter SLC38A2 fail to colonize the hepatic niche. Surprisingly, glutamine supplementation during the early metastatic stage does not enhance MC38 colonization and disease progression. Instead, at the intermediate stage—when macrometastases begin to form—exogenous glutamine substantially promotes MC38 outgrowth in the liver. Therefore, we infer that cancer cell–intrinsic glutamine uptake appears insufficient to drive liver metastasis. Further in vivo and in vitro experiments indicate that monocytes rely on glutamine to proliferate and transition to an inflammatory state, thereby facilitating pro-metastatic niche formation in the IRE1α-deficient liver. Glutamine addiction is a metabolic hallmark of proliferating cells^56^, explaining why IMs are adept at exploiting excess extracellular glutamine. Together, we have revealed a glutamine-IMs axis, initiated by hepatocyte IRE1α loss, which indirectly fuels liver metastasis.

Therapeutic strategies targeting the IRE1α–XBP1s axis have shown promise in preclinical cancer models and progressed to early clinical trials in patients with metastatic tumors (NCT03950570)^1,2,11,12^. However, most preclinical studies have focused on the primary TME, leaving its implications in metastatic contexts poorly understood. Notably, in a latent liver metastasis model, pancreatic cancer cells adopt a quiescent state accompanied by reduced IRE1α–XBP1s activity. Inducible XBP1s expression allows these cells to regain proliferative capacities and grow as macrometastases^57^. Paradoxically, reactivated cancer cells upregulate MHC I expression, rendering them visible to the immune system and calling into question the fundamental role of the IRE1α–XBP1s axis, despite the prevailing view that its tumor-intrinsic loss is immunogenic^4,5,10^. In our experiments, the IRE1α inhibitor MKC8866 (ORIN1001) effectively suppresses subcutaneous MC38 and LLC tumors but fails to ameliorate their liver metastases. Because the metastatic niche involves distinct cell-cell interactions compared to the primary TME, we conducted an in-depth investigation of the bystander effects mediated by peritumor hepatocytes. Genetic ablation of hepatocyte IRE1α or XBP1 exacerbates liver metastasis by activating the glutamine–IMs axis. Building on the previously known XBP1s–FoxO1 signaling^21^, we demonstrate that GS overexpression in peritumor hepatocytes is a downstream event, distinct from the transcriptional regulation of ER stress-related genes. However, based on our findings, we speculate that 1) activation of the XBP1s–FoxO1–GS signaling axis may occur under microenvironmental nutrient restriction—such as during competition with metastatic cancer cells; 2) healthy hepatocytes in the pre- or early-metastatic liver, which are not subject to ER stress, can engage the IRE1–XBP1s machinery as a protective mechanism to limit local pro-metastatic metainflammation.

In summary, we define a paradigm-shifting mechanism in liver metastasis which enables a context-dependent hepatocyte-monocyte interaction and provides a non-canonical rationale for targeting peritumor hepatocytes. Although clinical data on anti-metastatic efficacy are not yet available, IRE1α antagonism will likely require careful therapeutic design and strategic combination approaches for effective clinical application.

## Methods

### Animals

Wild-type (WT) C57BL/6 J and BALB/c mice were purchased from BesTest Bio-Tech (Zhuhai, China). *Ern1*^*fl/fl*^ and *Xbp1*^*fl/fl*^ mice on a C57BL/6 J background were generously provided by Profs. Y. Liu (Wuhan University) and L.H. Glimcher (Dana-Farber Cancer Institute). *Alb*^*Cre*^ (003574) and CD45.1 (006584) mice were obtained from The Jackson Laboratory. To achieve hepatocyte-specific gene depletion, *Ern1*^*fl/fl*^ and *Xbp1*^*fl/fl*^ mice were crossed with *Alb*^*Cre*^ mice, yielding *Ern1*^*CKO*^ and *Xbp1*^*CKO*^ mice, respectively. For experiments involving *Ern1*^*CKO*^ or *Xbp1*^*CKO*^ mice, littermate controls expressing WT ERN1 (*Ern1*^*fl/fl*^) or XBP1 (*Xbp1*^*fl/fl*^) were used. Cd45.1 mice were crossed with WT B6 (CD45.2) mice to yield heterozygous CD45.1/2 mice. All mice were housed in a specific pathogen-free facility maintained at 22–23 °C on a 12-h light/dark cycle, with ad libitum access to food and water. Mice aged 8–12 weeks were used for all experiments. Animals were randomly assigned to experimental groups. All animal procedures were approved by the Research Ethics Committee of the University of Macau (UMARE 034 2022). For subcutaneous tumor models, the maximum permitted tumor volume was 2,000 mm³. For metastatic liver tumor models, disease burden was monitored longitudinally, with humane endpoints defined as a maximum body weight loss of 10% relative to baseline. These pre specified limits were not exceeded in any experimental group, and all animals were humanely euthanized immediately upon reaching the defined endpoints.

### Cell cultures

MC38, LLC and 4T1 cells were grown in Dulbecco’s modified Eagle’s medium (DMEM) (Invitrogen) supplemented with 10% fetal bovine serum (FBS) (Gibco), 10 mM HEPES (Gibco), and 1% Penicillin/Streptomycin (Gibco). All cell lines were maintained at 37 °C, 5% CO_2_, and routinely tested negative for Mycoplasma.

Mouse primary hepatocytes were isolated using a two-step collagenase perfusion technique as described^58^. Following isolation, cells were seeded into 6-well plates at 1 × 10^6^/well and cultured in William’s E Medium (Gibco) supplemented with 10% FBS (Gibco), 1% penicillin/streptomycin (Gibco), 0.25 μg/mL amphotericin B (Gibco), 1% insulin-transferrin-selenium (ITS, Gibco), and 5 nM dexamethasone (Sigma-Aldrich) at 37 °C with 5% CO_2_.

BMIMs were generated by flushing bone marrow cells from mouse tibias and femurs, followed by red blood cell lysis using RBC lysis buffer (BioLegend). Cells were seeded into 6-well plates containing complete DMEM supplemented with 0.6 mM glutamine, 20 ng/ml M-CSF (PeproTech), 10% FBS (Gibco), 1% penicillin–streptomycin (Gibco), and 55 μM β-mercaptoethanol (Gibco), and cultured for 3 days. On day 3, the medium was replaced with fresh complete DMEM containing the indicated concentrations of glutamine (0.05 mM, 0.6 mM, or 2 mM) and 20 ng/ml M-CSF, and cells were cultured for an additional 2 days. On day 4, 500 μl of MC38 culture supernatant was added directly to each well. Loosely adherent cells were harvested on day 5.

For experiments involving conditioned medium supplementation and L-asparaginase treatment, bone marrow cells were isolated and cultured under identical initial conditions. On day 3, the medium was replaced with conditioned medium derived from *Ern1*^*fl/fl*^ or *Ern1*^*CKO*^ hepatocytes (0.6 mM Gln condition) and 20 ng/ml M-CSF, with or without 40 nM L-asparaginase (MCE), and cells were cultured for an additional 2 days. On day 4, 500 μl of MC38 culture supernatant was added to each well. Loosely adherent cells were harvested on day 5.

SLC38A2 knockout in MC38 cells was achieved with lentiviruses expressing Cas9 and either a non-targeting control sgRNA (sgNTC: ATGACACTTACGGTACTCGT) or an sgRNA targeting *Slc38a2* (sgSlc38a2: ATTAAATACTGACATTCCAA), as previously described^59^, GFP-positive cells were sorted by flow cytometry, expanded, and screened for Sl*c38a2* deletion by PCR (F: TAATCTGAGCAATGCGATTGTGG, R: AGATGGACGGAGTATAGCGAAAA).

To generate SLC38A2-deficient BMIMs, bone marrow cells were harvested from mouse femurs and tibias, and monocytes were isolated using the EasySep™ Mouse Monocyte Isolation Kit (STEMCELL). Cells were infected with lentiviruses (MOI 20) expressing Cas9 and either a non-targeting control sgRNA or sgSlc38a2, as described above, via spinfection at 800 × g for 2 h at 32 °C. Transfected cells were cultured in complete DMEM supplemented with 0.6 mM glutamine and 20 ng/ml M-CSF (PeproTech) for 3 days. Knockout of *Slc38a2* was confirmed by PCR. GFP-positive cells were traced by flow cytometry in in vitro or ex vivo experiments.

### Tumor models and treatments

To establish subcutaneous tumors, 1 × 10^6^ MC38 or LLC cells were injected into the right flank of C57BL/6 J mice. Tumor growth was monitored every 2–3 days using caliper measurements, and volumes were calculated using the formula: volume = (length × width^2^)/2.

Liver metastases were induced via intrasplenic injection of 1 × 10^6^ MC38 or LLC cells, following previously described protocols^60,61^. To model spontaneous breast cancer metastasis to the lungs and liver, female BALB/c mice were orthotopically inoculated with 1 × 10^6^ 4T1 cells into the mammary fat pad. Primary tumors were surgically resected 5 days after inoculation to permit metastatic dissemination. Mice were euthanized at indicated timepoints post-injection for downstream analyses. Portal vein blood was collected for glutamine quantification using an ELISA-based Glutamine Assay Kit (Abcam).

For drug treatment settings, tumor-bearing mice were randomized prior to treatment initiation. Therapeutic agents were administered as follows: 1) 300 mg/kg MKC8866 (Aladdin) via oral gavage every other day from Day 5 to 13 (MC38 or LLC model) or from Day 20 to 28 (4T1 model); 2) 200 mg/kg glutamine (Gibco) via intravenous injection on designated treatment days; 3) 15 mg/kg RS102895 (MCE) via intraperitoneal injection daily from Day 5 to Day 9; 4) 3 mg/kg DRP-104 (MCE) via intraperitoneal injection every other day from Day 5 to Day 13; 5) 10 mg/kg MSO (MCE) via intraperitoneal injection daily from Day 5 to Day 9.

In vivo transfection of XBP1s in hepatocytes was achieved via tail vein injection of an adenovirus type 5 (Ad5)-based vector (4 × 10⁷ PFU/g body weight)^21^ administered 7 days prior to tumor inoculation. An empty vector was used as a control.

To deplete specific immune cell populations, mice received intraperitoneal injections of monoclonal antibodies (100 µg per dose) targeting CD8 (BioXCell, BE0061, clone 2.43), NK1.1 (BioXCell, BE0036, clone PK136), or Ly6C (BioXCell, BE0203, clone Monts 1) one day prior to tumor inoculation and on Days 3 and 6 post-injection.

For adoptive cell transfer experiments, tumor-bearing mice were intravenously injected with CTV-labeled bone marrow monocytes or sgRNA/GFP-expressing BMIMs (1 × 10^5^) at designated timepoints.

### Patient samples

Human CRLM tissue specimens were collected from 38 patients who received standard neoadjuvant chemotherapy and radical resection of both primary and liver tumors between February 2012 and July 2016 at Sun Yat-sen University Cancer Center^62^. Liver tumors were confirmed to be of colorectal origin by an independent pathologist. The clinicopathological information of patients is summarized in Supplementary Data 4. All patients were followed up on a regular basis. The study was approved by the Medical Ethics Committee of SYSUCC. Written informed consent was obtained from the patients who donated samples.

### Histology and immunohistochemistry

Liver tissues were collected, fixed in 10% formaldehyde overnight before transferring to 70% ethanol. Following paraffin embedding and sectioning at 4 μm, tissues were stained with hematoxylin and eosin (H&E). Immunohistochemistry was performed according to standard protocols, as previously described. Tissue sections were dewaxed, rehydrated, and subjected to antigen retrieval. After blocking with goat serum at room temperature, the sections were incubated with primary antibodies: anti-GS (Abcam, ab228590, 1:1600), anti-XBP1s (Proteintech, 83959-5-RR, 1:1800), anti-IRE1α (p-S724) (Abcam, ab48187, 1:400), anti-Ki67 (Cell Signaling Technology, 9129, 1:400), followed by staining with horseradish peroxidase (HRP)-conjugated secondary antibodies. 3,3′-Diaminobenzidine tetrahydrochloride (DAB) was used as the chromogenic substrate. TUNEL staining was performed with the In Situ Cell Death Detection Kit (Roche, 11684817910). Whole-slide overview images at ×20 magnification were acquired using the NanoZoomer®S360 (HAMAMATSU PHOTONICS). Digital slides were visualized and analyzed using CaseViewer 2.3.0 software (3DHISTECH).

Quantification was performed on sections collected from the same liver lobe across experimental groups to ensure consistency. Metastatic burden was determined from H&E-stained sections and expressed as the percentage of tumor area relative to total liver area. For immunohistochemistry analysis, signal quantification was performed on standardized high-power fields (HPFs) acquired under a 40× objective, each corresponding to a fixed field size of approximately 310 μm × 310 μm. For each liver section, 5 independent, non-overlapping HPFs were analyzed at the tumor–liver interface. For GS immunolabeling analysis, the anatomical zone-3 pericentral region was excluded to avoid confounding physiological GS expression. Staining intensity and positive area percentage were quantified using ImageJ software. Data were averaged across biological replicates (*n* = 5–6 per group).

### Flow cytometry

Whole-liver tissues were minced and enzymatically dissociated using the Mouse Liver Dissociation Kit (130-105-807, Miltenyi) in conjunction with the gentleMACS Octo Dissociator with Heaters (Miltenyi), following the manufacturer’s protocol. The resulting cell suspension was filtered through a 70-μm cell strainer (Miltenyi), then subjected to a 40% Percoll gradient and centrifuged at 900 × *g* for 20 min at room temperature to enrich immune cells. Red blood cells were lysed using RBC lysis buffer (BioLegend), and the remaining cells were resuspended in staining buffer (PBS supplemented with 2% FBS and 1 mM EDTA). To block Fc receptors, cells were incubated with TruStain FcX™ PLUS (anti-mouse CD16/32) Antibody (Clone S17011E, BioLegend, 156603) for 10 min. Subsequently, surface staining was performed by incubating cells with specific antibodies for 30 min at 4 °C in the dark. For CellTrace™ Violet (Thermo Fisher) (CTV) staining, cells were incubated with 5 μM CTV in RPMI medium for 20 min at 37 °C following surface marker staining. For intracellular staining, cells were fixed, and permeabilized using the Foxp3/Transcription Factor Staining Buffer Set (eBioscience) according to the manufacturer’s instructions, then stained with intracellular or isotype control antibodies. Flow cytometric analysis was performed using either the Cytek Aurora (Cytek) or CytoFLEX (Beckman) instruments. Data were analyzed using FlowJo v10.8.1 (Treestar). Dead cells stained with Zombie NIR (BioLegend, 423106) or Zombie Aqua (BioLegend, 423102) were excluded from analysis. Gating strategies were validated using fluorescence-minus-one (FMO) controls for low-density antigens. The following antibodies were used: CD45 (Clone 30-F11, BioLegend, 103138), CD11b (Clone M1/70, BioLegend, 101251), F4/80 (Clone BM8, BioLegend, 123135), Ly6C (Clone HK1.4, BioLegend, 128005), CCR2 (Clone SA203G11, BioLegend, 150609), Ly6G (Clone 1A8, BioLegend, 127652), CD3 (Clone 145-2C11, BioLegend, 100328), CD4 (Clone RM4-5, BioLegend, 100536), TCR γ/δ Antibody (Clone GL3, BioLegend, 118120), FOXP3 (Clone 150D, BioLegend, 320008), CD8 (Clone 53-6.7, BioLegend, 100761), NK1.1 (Clone S17016D, 156513), Ki67 (Clone 11F6, BioLegend, 151211).

### Immunoprecipitation

Cells were washed with 2 ml of phosphate-buffered saline twice and then lysed with IP lysis buffer (Beyotime). After incubation on ice for 15 min and centrifugation at 13,523 × *g* at 4 °C for 20 min, the supernatant was saved and the protein concentration was determined with the BCA assay (Thermo Fisher). Proteins were either directly analyzed by immunoblotting as input or used for immunoprecipitation analysis. Briefly, the proteins were incubated with anti-Flag or Negative Control Nanobody Agarose Beads (AlpaLifeBio, KTSM1308 or KTSM1356) overnight. After washing and centrifugation, the supernatant was electrophoresed through SDS-PAGE.

### Western blot analysis

Cells were lysed on ice for 15 min using RIPA buffer (Beyotime), supplemented with a protease and phosphatase inhibitor cocktail (Thermo Fisher). Lysates were centrifuged at 13,523 × *g* at 4 °C for 20 min. Protein concentrations were quantified using a BCA assay kit (Thermo Fisher) and normalized accordingly. Equal amounts of protein were loaded onto a 10% SDS-PAGE gel and transferred to PVDF membranes (Life Technologies). Membranes were blocked in TBST containing 5% skim milk for 1 h, followed by overnight incubation at 4 °C with primary antibodies (anti-IRE1α, Cell Signaling Technology, 3294; anti-XBP1s, Cell Signaling Technology, 40435; anti-GS, Abcam, ab228590; anti-FoxO1, Cell Signaling Technology, 2880; anti-Flag, Cell Signaling Technology, 14793; GAPDH, YEASEN, 30203E350) diluted in blocking buffer. After three washes with TBST, membranes were incubated with secondary antibodies for 1 h at room temperature. Signal detection was performed using the ECL method, and images were acquired with the ChemiDoc Touch Imaging System (Bio-Rad). Quantitative densitometry of immunoblots was conducted using ImageJ software.

### Mass spectrometry

Peritumor (within a 5 mm radius) liver tissues from *Ern1*^*fl/fl*^ and *Ern1*^*CKO*^ mice (day 7 post-MC38 injection) were snap-frozen and stored at −80 °C. Metabolites were extracted using methanol: acetonitrile (1:1, v/v) after tissue homogenization. Liquid chromatography–mass spectrometry (LC–MS) analysis was conducted using an Agilent 1290 system coupled to a TripleTOF™ 6600plus (SCIEX) mass spectrometer. Chromatographic separation was performed on an ACQUITY UPLC BEH C18 column (100 × 2.1 mm, 1.7 μm) with a 12-min gradient. Data were acquired in both positive and negative ionization modes using information-dependent acquisition (IDA) across m/z 60–1200. Raw data were processed with XCMS for feature detection and alignment. Metabolite identification was performed using MetDNA, and differential metabolites were selected via MetaboAnalyst (fold change > 1.5, P < 0.05, VIP > 1).

For the tumor-free cohort, independent LC–MS analysis was conducted using a Thermo Scientific Vanquish UHPLC system coupled to a Q Exactive™ HF/Q Exactive™ HF-X (Thermo Fisher) mass spectrometer. Chromatographic separation was achieved on a Hypersil Gold (100 × 2.1 mm, 1.9 μm) column. Data were acquired in both positive and negative electrospray ionization modes across a mass range of m/z 100–1500.

### Glutamine measurement by enzyme-linked immunosorbent assay (ELISA)

Glutamine concentrations in portal vein serum and cell lysates were measured using a commercial ELISA kit (Elabscience, E-BC-K853-M) according to the manufacturer’s instructions. Portal vein blood was collected at sacrifice and serum was isolated by centrifugation. MC38 cells or BMIMs were lysed in ice-cold lysis buffer and centrifuged to obtain clarified lysates. Standards and samples were analyzed in duplicate, and absorbance was measured at 450 nm using a microplate reader. Glutamine concentrations were calculated from standard curves and expressed as mM for serum samples and normalized to total protein content for cell lysates.

### Multiplex immunofluorescence staining

Multiplex immunofluorescence staining was performed according to the protocol provided with the Tyramide Manual IHC kits (Biossci). Tyramide signal amplification (TSA) dyes (iFluor® 488, iFluor® 555, and iFluor® 647 (AAT Bioquest)) were used to enhance signal following secondary antibody incubation. Primary antibodies targeting mouse or human antigens included: anti-CCR2 (Abcam, ab314120, 1:400), anti-Ly6C (Abcam, ab314120, 1:400), anti-GS (Abcam, ab228590, 1:4000), anti-XBP1s (Proteintech, 83959-5-RR, 1:1000), anti-CD14 (Cell Signaling Technology, 75181S, 1:1000), and anti-HNF4α (Abcam, ab181604, 1:2000). Nuclei were counterstained with 4′,6-diamidino-2-phenylindole (DAPI; Solarbio). Whole-slide overview images at ×20 magnification were acquired using the Pannoramic MIDI scanner (3DHISTECH). Digital slides were visualized and analyzed using CaseViewer 2.3.0 software (3DHISTECH).

Quantification of multiplex immunofluorescence signals was performed using standardized high-power fields (HPFs) acquired under a ×40 objective, each corresponding to a fixed field size of approximately 310 μm × 310 μm. Mean Fluorescence Intensity (MFI) was quantified at single-cell resolution, representing the average pixel grayscale value extracted strictly within automated single-cell segmentation masks generated via DAPI and specific markers. For each liver section, 5 independent, non-overlapping HPFs at the tumor–liver interface were batch-processed to determine the average values for GS and XBP1s expression densities, as well as the absolute monocyte counts.

### Bulk RNA-seq

RNA-seq was performed as described^63^. Briefly, BMIM were cultured as indicated. BM cells were first stained with F4/80-APC antibody (BioLegend) and purified using the EasySep™ Mouse APC Positive Selection Kit II (STEMCELL). RNA was isolated from F4/80^+^ or F4/80^-^ cells with TRIzol® (Thermo) reagent for library construction. Libraries were sequenced on Illumina NovaSeq 6000 platform (Lc-Bio, Hangzhou, China) in150-bp pair-end mode (PE150).

### Single-cell RNA-seq

Single-cell suspensions were prepared from whole-liver tissues of *Ern1*^*fl/fl*^ and *Ern1*^*CKO*^ mice eight days after intrasplenic injection of MC38 tumor cells, as described above. Cells were stained with Zombie Aqua® and antibodies against CD45, CD11b, and CD3 (BioLegend). Zombie Aqua⁻CD45⁺CD11b⁺CD3⁻ single cells were sorted using a BD FACSAria II flow cytometer. Sorted cells were processed using the 10x Genomics Chromium Controller according to the manufacturer’s protocol. Libraries were constructed using the Chromium Next GEM Single Cell 3’ Kit v2 and the associated Library Construction Kit (10x Genomics). Briefly, cells were suspended in cold PBS containing 0.04% BSA, lysed, and barcoded via reverse transcription within GEMs using a Bio-Rad S1000™ Touch Thermal Cycler. Sequencing was performed on an Illumina NovaSeq 6000 platform (Lc-Bio, Hangzhou, China) with paired-end 150 bp reads, achieving a depth of ≥100,000 reads per cell.

Raw sequencing data were processed using CellRanger (v7.2.0) for alignment, barcode assignment, and UMI counting. Reads were aligned to the GRCh38 reference genome to generate feature-barcode matrices. Count matrices from all samples were aggregated for downstream analysis using Seurat (v4.3.0). A multi-step quality control procedure was implemented to exclude low-quality cells. To ensure a pure immune cell population enriched via flow cytometry sorting, only cells expressing *Ptprc* (CD45) were retained. Further filtering was applied based on the following criteria: (1) total UMI count >1000; (2) number of detected genes between 300 and 4000; and (3) mitochondrial gene percentage <15%. Genes detected in fewer than three cells were excluded.

After quality control, 7515 high-quality myeloid cells from two experimental groups were retained. The filtered gene-cell matrix was normalized using the LogNormalize method. The top 3000 highly variable genes were identified using the FindVariableFeatures function. Data scaling was performed with the ScaleData function, followed by principal component analysis (PCA) using the selected variable genes. The first 15 principal components were used to construct a shared nearest neighbor (SNN) graph and generate cell clusters. Dimensionality reduction and visualization were performed using UMAP.

Cluster-specific marker genes were identified using the FindAllMarkers function (minimum log₂ fold-change of 0.2, expressed in at least 25% of cells within the cluster). Cell types were annotated by evaluating the expression of canonical myeloid markers alongside cluster-specific differentially expressed genes. Eight distinct myeloid cell subpopulations were identified and grouped into four major categories. The activity of the “GOBP_ATP_METABOLIC_PROCESS” pathway was assessed per cell using the AddModuleScore function in Seurat, enabling comparison of metabolic activity across cell types and conditions. To investigate myeloid cell differentiation trajectories, pseudotime analysis was performed using the Monocle R package (v2.28.0) on data subsets to reconstruct and compare developmental paths between experimental groups.

### Statistical analysis

Histological assessments of mouse and human tissues were performed in a blinded manner. Each experimental group included a minimum of four mice. For cellular assays, a minimum of three independent biological replicates was used per condition. Immunoblot and immunofluorescence images are representative of experiments independently repeated at least three times with consistent results. Data are presented as mean ± standard error of the mean (SEM). Statistical analyses were performed using two-tailed unpaired Student’s *t* tests, one-way or two-way ANOVA, or two-tailed unpaired Wilcoxon rank-sum tests, as appropriate. Associated *P* values were calculated to assess relationships between continuous variables. Survival analyses were conducted using the Kaplan–Meier method, with statistical significance determined by log-rank tests. A *P* value < 0.05 was considered statistically significant. All analyses were performed using R (v4.2.2) or GraphPad Prism (v9.5.1).

### Reporting summary

Further information on research design is available in the Nature Portfolio Reporting Summary linked to this article.

## Supplementary information

Supplementary Information

Description of Additional Supplementary Files

Supplementary Data 1

Supplementary Data 2

Supplementary Data 3

Supplementary Data 4

Reporting Summary

Transparent Peer Review file

## Source data

Source Data

## Data Availability

Data for single-cell RNA-seq and bulk RNA-seq are available through the BioProject portal (PRJNA1322094 https://www.ncbi.nlm.nih.gov/bioproject/?term=PRJNA1322094). All the other data are available within the article and its Supplementary Information. Source data are provided with this paper.
