# Supplementary material for: Hepatocyte IRE1α–XBP1s axis prevents pro-metastatic niche formation through metabolic rewiring of inflammatory monocytes
Source: Nat Commun. 2026 Nov 3;17:10961. doi: 10.1038/s41467-026-78812-2 (PMC13731655; doi:10.1038/s41467-026-78812-2)
Supplement: Supplementary file 1 [file 41467_2026_78812_MOESM1_ESM.pdf]

Supplementary Information

Hepatocyte IRE1α–XBP1s axis prevents pro-metastatic niche formation through metabolic rewiring of inflammatory monocytes

Authors: Haiyan Zhang[1#*], Peibin Liang[2#], Wei Yang[3,4#], Xiaocong Chen[2,5#], Yizhen Fu[3,6#], Chonghao Chen[2], Zeren Yin[2], Xiaolin Li[2,7], Xin Pei[2], Josh Haipeng Lei[2], Xiangpeng Chu[2], Zhi-Bin Zhao[8], Hai Hu[7], Minshan Chen[3,6], Hong Pan[5], Zhe-Xiong Lian[8], Xiaofan Ding[2], Nils Halberg[1], Grant A Ramm[1,9], Jiajie Hou[1*]

Affiliations:

[1]QIMR Berghofer Medical Research Institute, Brisbane, Queensland, Australia

[2]Cancer Center, Faculty of Health Sciences, University of Macau, Macau SAR, China

[3]State Key Laboratory of Oncology in South China, Collaborative Innovation Center for Cancer Medicine, Sun Yat-sen University Cancer Center, Guangzhou 510060, China

[4]Department of Medical Oncology, Sun Yat-sen University Cancer Center, Guangzhou 510060, China

[5]Guangdong Key Laboratory of Nanomedicine, CAS-HK Joint Lab of Biomaterials, Shenzhen Institutes of Advanced Technology (SIAT), Chinese Academy of Sciences, Shenzhen 518055, China

[6]Department of Liver Surgery, Sun Yat-sen University Cancer Center, Guangzhou 510060, China

[7]Breast Cancer Center, Zhejiang Cancer Hospital, Hangzhou Institute of Medicine, Chinese Academy of Sciences, Hangzhou 310022, China

[8]Guangdong Provincial People's Hospital (Guangdong Academy of Medical Sciences), Southern Medical University, Guangzhou 510080, China

[9]Faculty of Medicine, The University of Queensland, Brisbane, Queensland, Australia

[#]These authors contributed equally

*Correspondence: haiyan.zhang@qimrb.edu.au (H.Z.); jiajie.hou@qimrb.edu.au (J.H.)

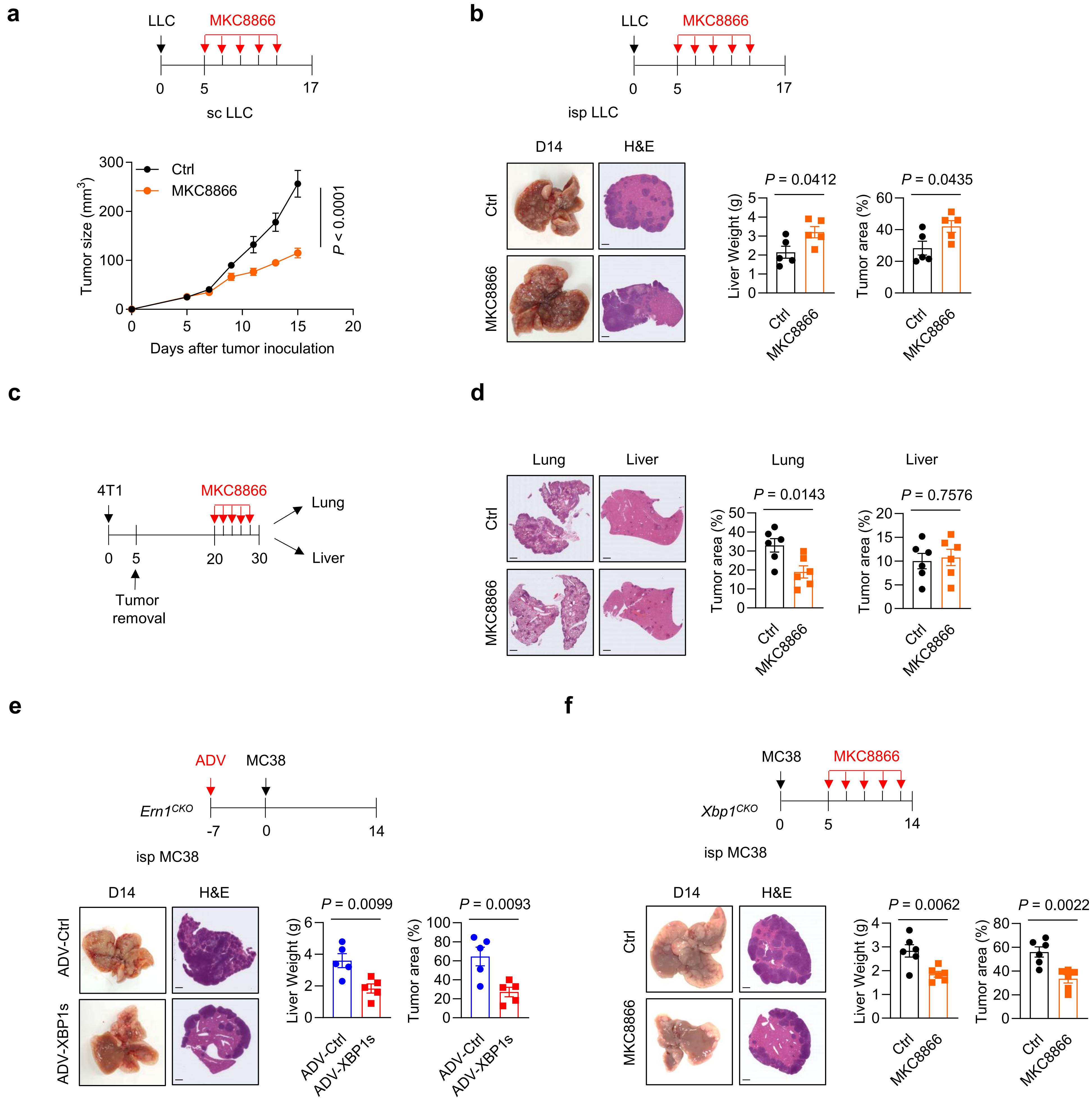
a
LLC
MKC8866
0
5
17
sc LLC
Tumor size (mm3)
Ctrl
MKC8866
P < 0.0001
Days after tumor inoculation
b
LLC
MKC8866
0
5
17
isp LLC
D14
H&E
Ctrl
MKC8866
P = 0.0412
Liver Weight (g)
P = 0.0435
Tumor area (%)
c
4T1
MKC8866
Lung
Liver
0
5
20
30
Tumor
removal
d
Lung
Liver
Ctrl
MKC8866
P = 0.0143
P = 0.7576
Tumor area (%)
e
ADV
MC38
Ern1CKO
-7
0
14
isp MC38
D14
H&E
ADV-Ctrl
ADV-XBP1s
P = 0.0099
Liver Weight (g)
P = 0.0093
Tumor area (%)
f
MC38
MKC8866
Xbp1CKO
0
5
14
isp MC38
D14
H&E
Ctrl
MKC8866
P = 0.0062
Liver Weight (g)
P = 0.0022
Tumor area (%)

Supplementary Fig. 1 | Targeting IRE1α–XBP1 signaling promotes liver metastasis in an environmental glutamine-dependent manner.

a, Experimental design and tumor growth in WT mice after subcutaneous injection (sc) of LLC cells and treatment with MKC8866 or vehicle (n = 5 per group). **b,** Liver metastasis in WT mice after intrasplenic injection (isp) of LLC cells and treatment with MKC8866 or vehicle, showing experimental scheme, liver weight, H&E staining (Scale bars,1000 μm), and quantification (n = 5 per group). **c, d,** Spontaneous lung and liver metastases following orthotopic injection of 4T1 cells and treatment with MKC8866 or vehicle. **c,** Experimental scheme of the 4T1 orthotopic model. **d,** Representative H&E staining images (left) (Scale bars,1000 μm) and quantification (right) (n = 6 per group). **e,** Liver mass and H&E staining images (Scale bars,1000 μm) of MC38-bearing $Ern1^{CKO}$ mice pretreated with XBP1s-overexpressing or control adenoviruses (ADV) (n = 5 per group). **f,** Liver mass and H&E staining images (Scale bars,1000 μm) of MC38-bearing $Xbp1^{CKO}$ mice treated with MKC8866 or vehicle (n = 6 per group). Data are represented as mean ± SEM (a–f). Statistical analysis was performed using two-way ANOVA (a) or two-tailed, unpaired Student's t test (b–f). Source data are provided as a Source Data file.

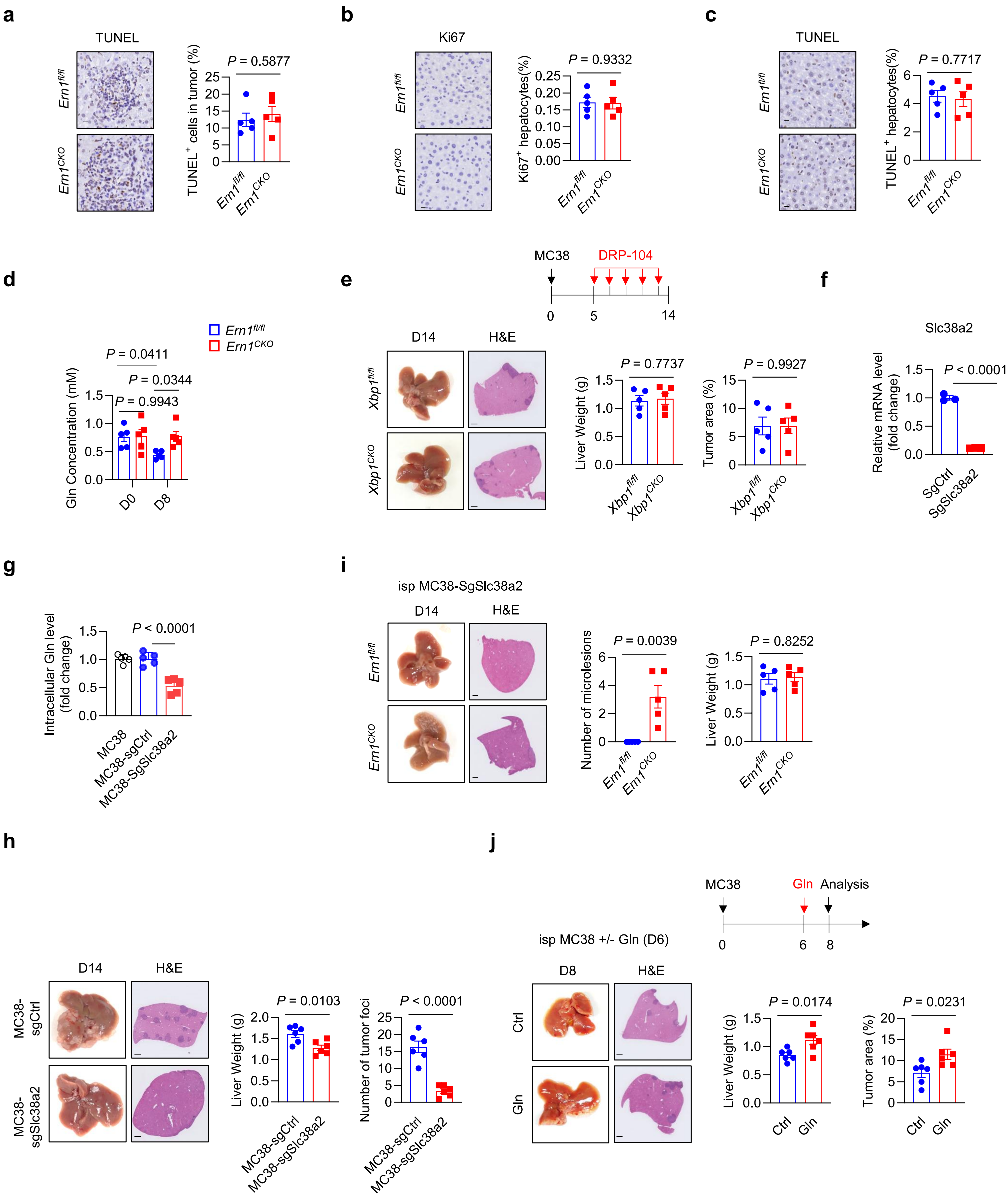

a
TUNEL
Ern1fl/fl
Ern1CKO
TUNEL+ cells in tumor (%)
P = 0.5877
b
Ki67
Ki67+ hepatocytes(%)
P = 0.9332
c
TUNEL
TUNEL+ hepatocytes(%)
P = 0.7717
d
Ern1fl/fl
Ern1CKO
Gln Concentration (mM)
P = 0.0411
P = 0.0344
P = 0.9943
D0
D8
e
MC38
DRP-104
0
5
14
D14
H&E
Xbp1fl/fl
Xbp1CKO
Liver Weight (g)
P = 0.7737
Tumor area (%)
P = 0.9927
f
Slc38a2
Relative mRNA level (fold change)
P < 0.0001
SgCtrl
SgSlc38a2
g
Intracellular Gln level (fold change)
P < 0.0001
MC38
MC38-sgCtrl
MC38-SgSlc38a2
i
isp MC38-SgSlc38a2
D14
H&E
Number of microlesions
P = 0.0039
Liver Weight (g)
P = 0.8252
h
D14
H&E
MC38-sgCtrl
MC38-sgSlc38a2
Liver Weight (g)
P = 0.0103
Number of tumor foci
P < 0.0001
j
MC38
Gln
Analysis
0
6
8
isp MC38 +/- Gln (D6)
D8
H&E
Ctrl
Gln
Liver Weight (g)
P = 0.0174
Tumor area (%)
P = 0.0231

Supplementary Fig. 2 | Excessive glutamine supports liver metastasis in a context-dependent manner.

a–c, Representative images and quantification of immunohistochemistry of day 7 MC38-bearing *Ern1*$^{fl/fl}$ and *Ern1*^{CKO} livers (n = 5 per group), showing TUNEL staining on tumor areas (a) and Ki67 (b) and TUNEL (c) staining on non-tumor areas. Scale bars, 10 μm. **d,** Glutamine levels in portal vein blood from *Ern1*$^{fl/fl}$ and *Ern1*^{CKO} mice (n = 5 per group). **e,** Liver mass, representative H&E images (Scale bars,1000 μm) and quantification of metastases of MC38-bearing *Xbp1*$^{fl/fl}$ and *Xbp1*^{CKO} mice that were treated with DRP-104 or vehicle control (n = 5 per group). **f**, *Slc38a2* mRNA levels in MC38 cells as quantified by qPCR (n = 3 per group). **g**, Intracellular glutamine levels of MC38 cells as measured by ELISA (n = 5 per group). **h,** Liver mass, representative H&E images (Scale bars,1000 μm) and quantification of liver metastases formed by MC38-sgCtrl or MC38-sgSlc38a2 cells in WT mice (n = 6 per group). **i**, Liver metastasis in MC38-sgSlc38a2-bearing *Ern1*$^{fl/fl}$ and *Ern1*^{CKO} mice (n = 5 per group). Scale bars, 1000 μm. **j**, Liver metastasis in MC38-bearing WT mice supplemented with glutamine or vehicle at day 6 post-isp (n = 5 per group). Scale bars,1000 μm. Data are represented as mean ± SEM (a–j). Statistical analysis was performed using two-way ANOVA (a, b) or two-tailed, unpaired Student’s t test (c–j). Source data are provided as a Source Data file.

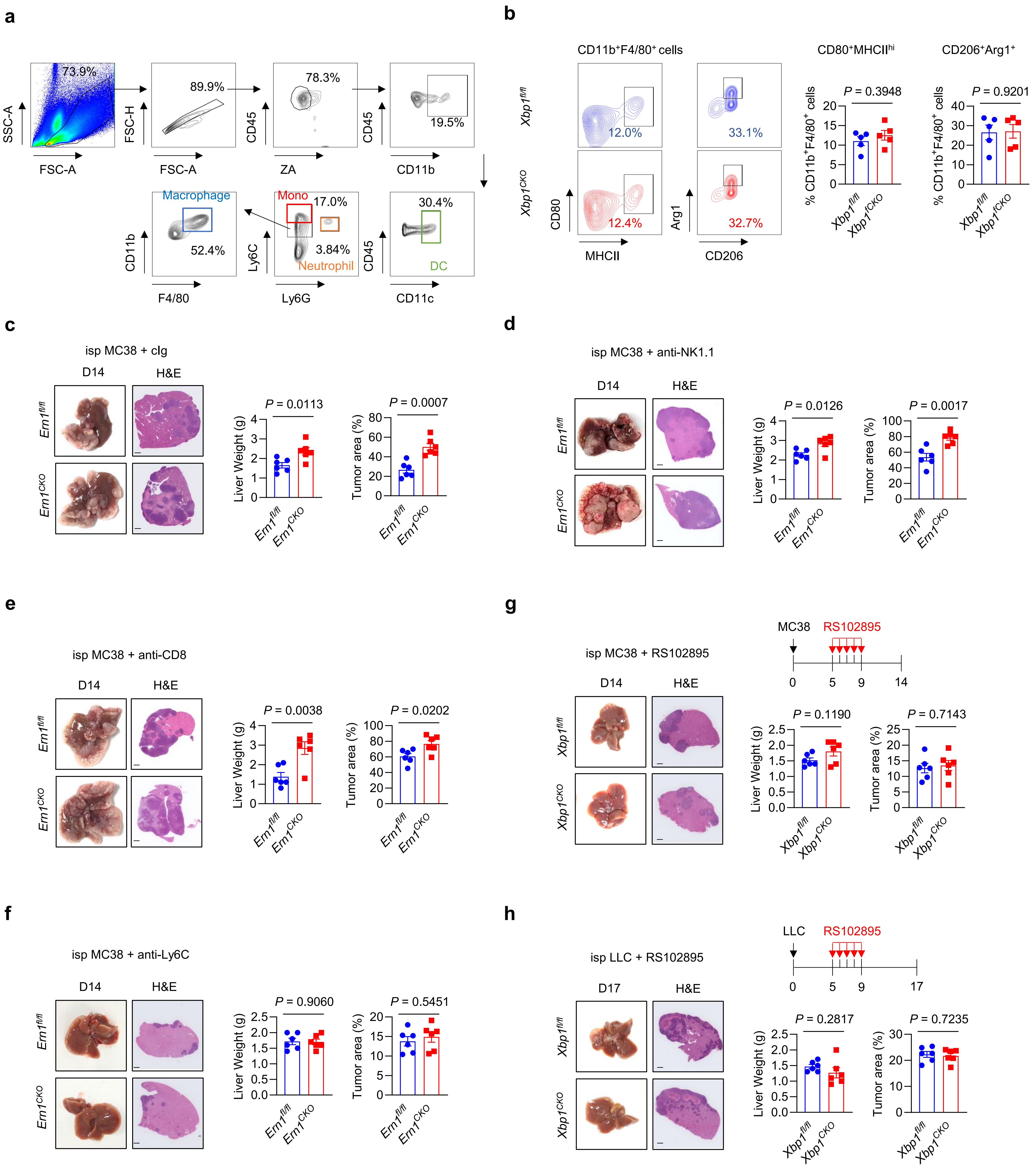
a
SSC-A
FSC-A
73.9%
FSC-H
FSC-A
89.9%
CD45
ZA
78.3%
CD45
CD11b
19.5%
CD11b
F4/80
Macrophage
52.4%
Ly6C
Ly6G
Mono
17.0%
3.84%
Neutrophil
CD45
CD11c
30.4%
DC
b
CD11b⁺F4/80⁺ cells
Xbp1fl/fl
Xbp1CKO
12.0%
33.1%
12.4%
32.7%
CD80
MHCII
Arg1
CD206
CD80⁺MHCIIhi
CD206⁺Arg1⁺
P = 0.3948
P = 0.9201
% CD11b⁺F4/80⁺ cells
Xbp1fl/fl
Xbp1fCKO
c
isp MC38 + cIg
D14
H&E
Ern1fl/fl
Ern1CKO
P = 0.0113
P = 0.0007
Liver Weight (g)
Tumor area (%)
d
isp MC38 + anti-NK1.1
D14
H&E
Ern1fl/fl
Ern1CKO
P = 0.0126
P = 0.0017
Liver Weight (g)
Tumor area (%)
e
isp MC38 + anti-CD8
D14
H&E
Ern1fl/fl
Ern1CKO
P = 0.0038
P = 0.0202
Liver Weight (g)
Tumor area (%)
g
MC38
RS102895
0
5
9
14
isp MC38 + RS102895
D14
H&E
Xbp1fl/fl
Xbp1CKO
P = 0.1190
P = 0.7143
Liver Weight (g)
Tumor area (%)
f
isp MC38 + anti-Ly6C
D14
H&E
Ern1fl/fl
Ern1CKO
P = 0.9060
P = 0.5451
Liver Weight (g)
Tumor area (%)
h
LLC
RS102895
0
5
9
17
isp LLC + RS102895
D17
H&E
Xbp1fl/fl
Xbp1CKO
P = 0.2817
P = 0.7235
Liver Weight (g)
Tumor area (%)

Supplementary Fig. 3 | Inflammatory monocytes constitute the pro-metastatic niche in IRE1α-deficient livers.

a, Gating strategy used to delineate liver-infiltrating myeloid populations in MC38-bearing mice. The monocyte gating scheme shown here was applied consistently across all subsequent flow-cytometric analyses. **b,** Flow cytometric analysis of $CD11b^{+}F4/80^{+}$ cells within day 7 MC38-bearing *Xbp1*$^{fl/fl}$ and *Xbp1*^{CKO} livers (n = 5 per group). **c–f**, Liver metastasis in MC38-bearing *Ern1*$^{fl/fl}$ and *Ern1*^{CKO} mice that were treated with control IgG (c), anti-NK1.1 (d), anti-CD8 (e), anti-Ly6C (f) (n = 6 per group). **g, h,** Liver metastasis in MC38-bearing (g) or LLC-bearing (h) *Xbp1*$^{fl/fl}$ and *Xbp1*^{CKO} mice that were treated with RS102895 or vehicle control (n = 6 per group). Scale bars,1000 μm (c–h). Data are represented as mean ± SEM and analyzed by two-tailed, unpaired Student's t test (b–f). Source data are provided as a Source Data file.

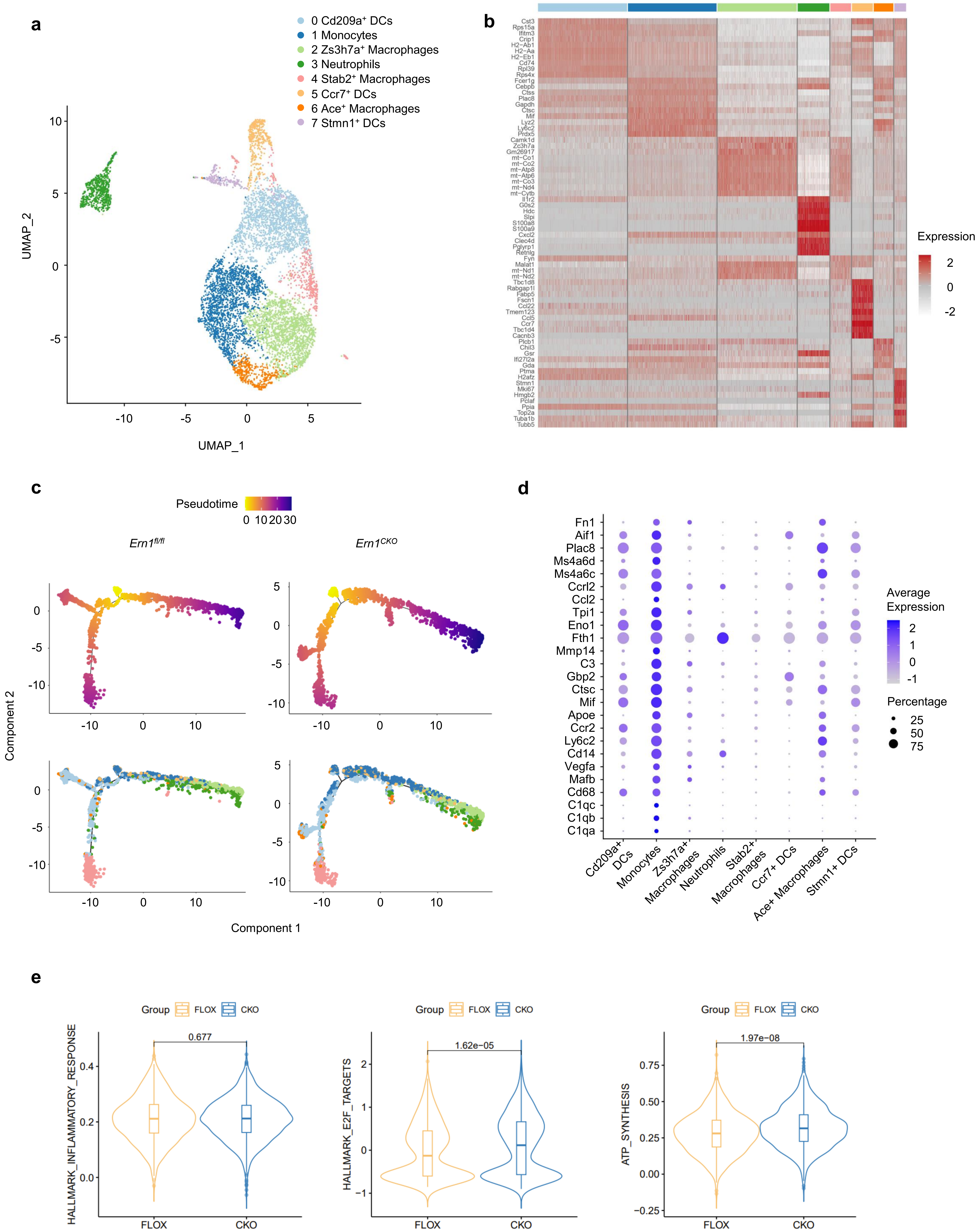
a
0 Cd209a+ DCs
1 Monocytes
2 Zs3h7a+ Macrophages
3 Neutrophils
4 Stab2+ Macrophages
5 Ccr7+ DCs
6 Ace+ Macrophages
7 Stmn1+ DCs
UMAP_2
UMAP_1
b
Expression
c
Pseudotime
Ern1fl/fl
Ern1CKO
Component 2
Component 1
d
Fn1
Aif1
Plac8
Ms4a6d
Ms4a6c
Ccrl2
Ccl2
Tpi1
Eno1
Fth1
Mmp14
C3
Gbp2
Ctsc
Mif
Apoe
Ccr2
Ly6c2
Cd14
Vegfa
Mafb
Cd68
C1qc
C1qb
C1qa
Cd209a+ DCs
Monocytes
Zs3h7a+ Macrophages
Neutrophils
Stab2+ Macrophages
Ccr7+ DCs
Ace+ Macrophages
Stmn1+ DCs
Average Expression
Percentage
e
Group FLOX CKO
HALLMARK_INFLAMMATORY_RESPONSE
0.677
HALLMARK_E2F_TARGETS
1.62e−05
ATP_SYNTHESIS
1.97e−08
FLOX
CKO

Supplementary Fig. 4 | scRNA-seq analysis reveals IM accumulation upon hepatocyte IRE1α deletion.

a, UMAP visualization of 7,515 $CD45^{+}CD11b^{+}CD3^{-}$ myeloid cells sorted from day 7 MC38-bearing *Ern1*$^{fl/fl}$ and *Ern1*^{CKO} livers (n = 3 per group), annotated into eight major cell lineages. **b,** Heatmap displaying the expression of marker genes across $CD11b^{+}$ myeloid-cell subpopulations. **c,** Cells colored by pseudotime, illustrating dynamic progression from the root to terminal states. **d,** Dot plot showing canonical markers used to identify IMs. **e,** Violin plots comparing the pathway scores of inflammatory responses, E2F targets, and ATP synthesis in IMs between *Ern1*$^{fl/fl}$ and *Ern1*^{CKO} groups. Data are represented as violin plots showing density, median, and interquartile range. Statistical analysis was performed using two-tailed, unpaired Wilcoxon rank-sum test (e). Source data are provided as a Source Data file.

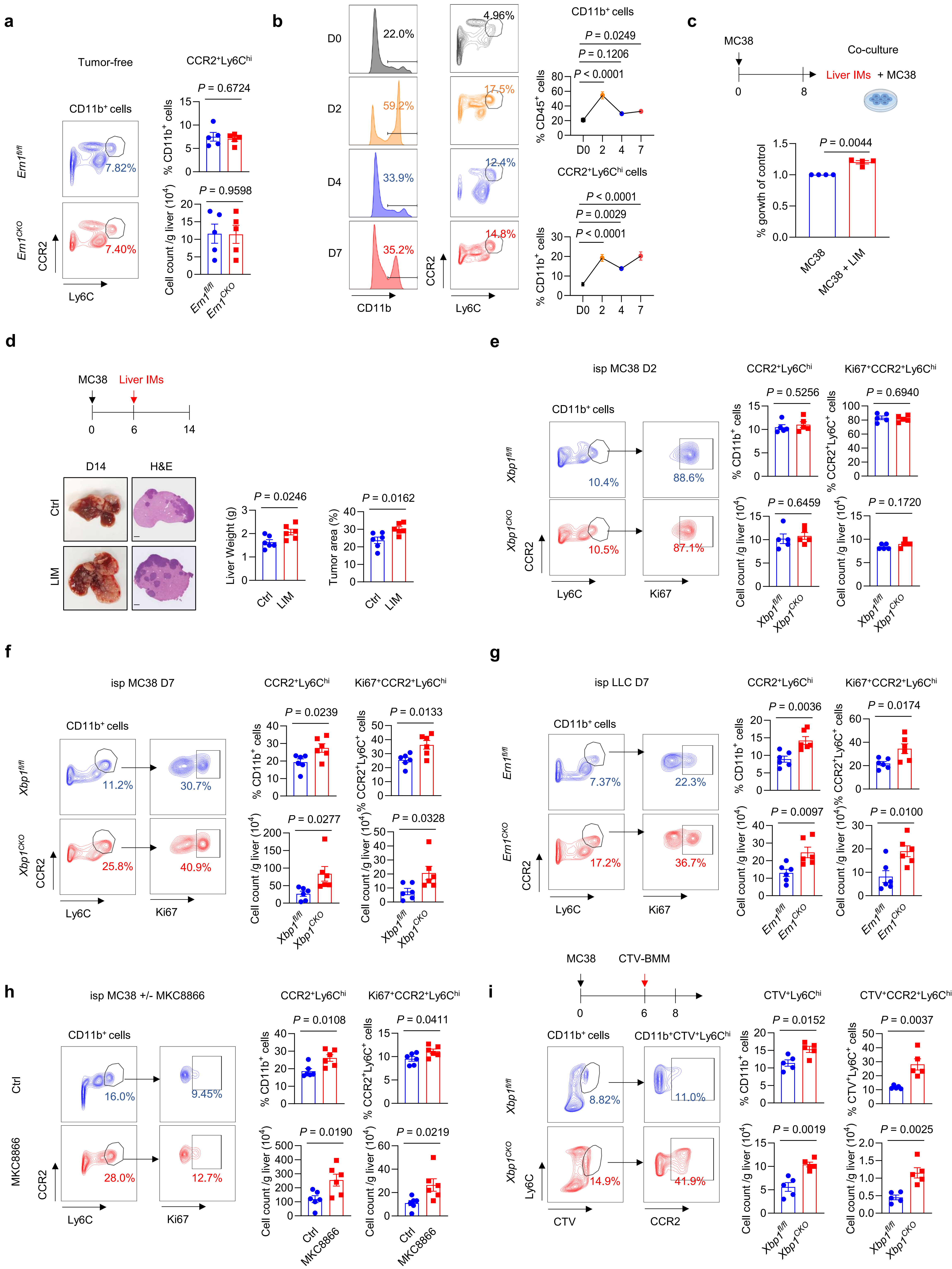
a
Tumor-free
CD11b+ cells
Ern1fl/fl
Ern1CKO
7.82%
7.40%
CCR2
Ly6C
CCR2+Ly6Chi
P = 0.6724
% CD11b+ cells
P = 0.9598
Cell count /g liver (10^4)
b
D0
D2
D4
D7
22.0%
59.2%
33.9%
35.2%
4.96%
17.5%
12.4%
14.8%
CD11b
Ly6C
CD11b+ cells
P = 0.0249
P = 0.1206
P < 0.0001
% CD45+ cells
CCR2+Ly6Chi cells
P < 0.0001
P = 0.0029
P < 0.0001
% CD11b+ cells
c
MC38
Co-culture
Liver IMs + MC38
% gorwth of control
P = 0.0044
MC38
MC38 + LIM
d
MC38
Liver IMs
D14
H&E
Ctrl
LIM
Liver Weight (g)
P = 0.0246
Tumor area (%)
P = 0.0162
e
isp MC38 D2
CD11b+ cells
Xbp1fl/fl
Xbp1CKO
10.4%
88.6%
10.5%
87.1%
Ki67
CCR2+Ly6Chi
Ki67+CCR2+Ly6Chi
P = 0.5256
P = 0.6940
P = 0.6459
P = 0.1720
% CCR2+Ly6C+ cells
f
isp MC38 D7
11.2%
30.7%
25.8%
40.9%
P = 0.0239
P = 0.0133
P = 0.0277
P = 0.0328
g
isp LLC D7
7.37%
22.3%
17.2%
36.7%
P = 0.0036
P = 0.0174
P = 0.0097
P = 0.0100
h
isp MC38 +/- MKC8866
Ctrl
MKC8866
16.0%
9.45%
28.0%
12.7%
P = 0.0108
P = 0.0411
P = 0.0190
P = 0.0219
i
MC38
CTV-BMM
CD11b+CTV+Ly6Chi
8.82%
11.0%
14.9%
41.9%
CTV
CTV+Ly6Chi
CTV+CCR2+Ly6Chi
P = 0.0152
P = 0.0037
P = 0.0019
P = 0.0025
% CTV+Ly6C+ cells

Supplementary Fig. 5 | Disrupting hepatocyte IRE1α–XBP1 axis enhances IM expansion.

a, Flow cytometric analysis of liver-infiltrating IMs in naïve *Ern1*$^{fl/fl}$ and *Ern1*^{CKO} mice (n = 5 per group). **b,** Flow cytometric analysis of IMs in MC38-bearing WT mice at indicated timepoints (n = 4 per group). **c,** Experimental scheme (top) and cell proliferation analysis (bottom) of MC38 cells cultured with or without $CCR2^+$ IMs sorted from WT MC38 metastasis-bearing livers (n = 4 per group). **d,** Adoptive transfer of liver IMs into MC38 tumor-bearing mice, showing experimental timeline (top), representative liver images and H&E staining (bottom left), liver weight, and quantification of tumor area (n = 6 per group). Scale bars,1000 μm. **e,** Quantification of IMs in MC38-bearing *Xbp1*^{CKO} and *Xbp1*$^{fl/fl}$ mice at day 2 (n = 5 per group) post isp. **f, g,** Quantification of IMs in MC38 (f) and LCC (g) -bearing *Ern1*$^{fl/fl}$ and *Ern1*^{CKO} mice at day 7 (n = 6 per group) post isp. **h,** Flow cytometric analysis of liver-infiltrating IMs in MC38-bearing WT mice that were treated with MKC8886 or vehicle control (n = 6 per group). **i,** Flow cytometric analysis of CTV-labeled monocytes after transferred into MC38-bearing *Xbp1*^{CKO} and *Xbp1*$^{fl/fl}$ mice (n = 5 per group). Data are represented as mean ± SEM and analyzed by two-tailed, unpaired Student's t test (a–i). Some elements in c were created by Figdraw (www.figdraw.com). Source data are provided as a Source Data file.

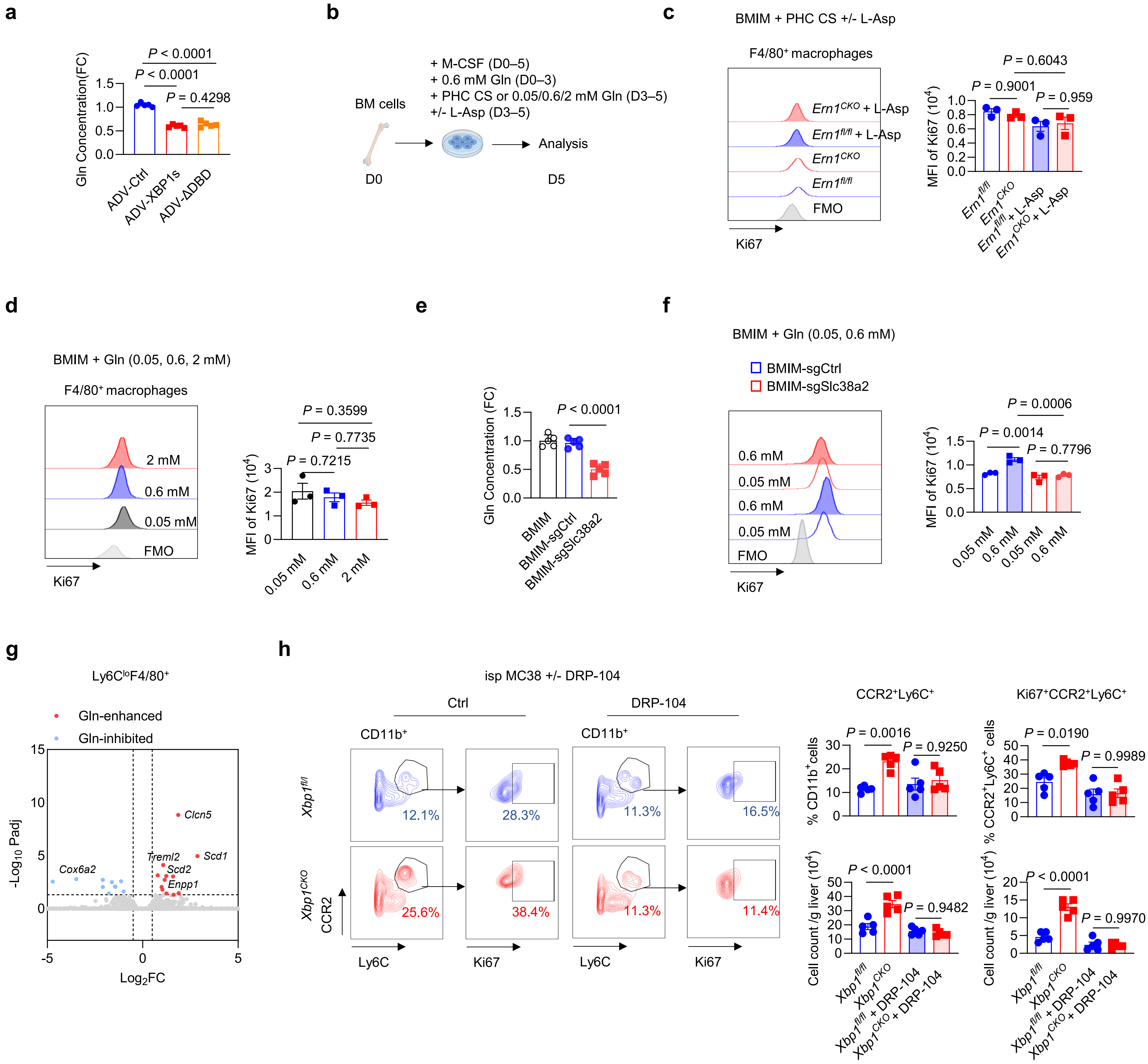
a
b
c
d
e
f
g
h
Gln Concentration(FC)
ADV-Ctrl
ADV-XBP1s
ADV-ΔDBD
P < 0.0001
P < 0.0001
P = 0.4298
BM cells
+ M-CSF (D0–5)
+ 0.6 mM Gln (D0–3)
+ PHC CS or 0.05/0.6/2 mM Gln (D3–5)
+/- L-Asp (D3–5)
Analysis
D0
D5
BMIM + PHC CS +/- L-Asp
F4/80+ macrophages
Ern1CKO + L-Asp
Ern1fl/fl + L-Asp
Ern1CKO
Ern1fl/fl
FMO
Ki67
MFI of Ki67 (10^4)
P = 0.6043
P = 0.9001
P = 0.959
BMIM + Gln (0.05, 0.6, 2 mM)
F4/80+ macrophages
2 mM
0.6 mM
0.05 mM
FMO
Ki67
MFI of Ki67 (10^4)
P = 0.3599
P = 0.7735
P = 0.7215
0.05 mM
0.6 mM
2 mM
Gln Concentration (FC)
P < 0.0001
BMIM
BMIM-sgCtrl
BMIM-sgSlc38a2
BMIM + Gln (0.05, 0.6 mM)
BMIM-sgCtrl
BMIM-sgSlc38a2
0.6 mM
0.05 mM
0.6 mM
0.05 mM
FMO
Ki67
MFI of Ki67 (10^4)
P = 0.0006
P = 0.0014
P = 0.7796
0.05 mM
0.6 mM
0.05 mM
0.6 mM
Ly6C^lo F4/80+
Gln-enhanced
Gln-inhibited
-Log10 Padj
Log2FC
Clcn5
Scd1
Treml2
Scd2
Enpp1
Cox6a2
isp MC38 +/- DRP-104
Ctrl
DRP-104
CD11b+
CD11b+
Xbp1fl/fl
Xbp1CKO
CCR2
Ly6C
Ki67
Ly6C
Ki67
12.1%
28.3%
11.3%
16.5%
25.6%
38.4%
11.3%
11.4%
CCR2+Ly6C+
Ki67+CCR2+Ly6C+
% CD11b+ cells
P = 0.0016
P = 0.9250
% CCR2+Ly6C+ cells
P = 0.0190
P = 0.9989
Cell count /g liver (10^4)
P < 0.0001
P = 0.9482
Cell count /g liver (10^4)
P < 0.0001
P = 0.9970
Xbp1fl/fl
Xbp1CKO
Xbp1fl/fl + DRP-104
Xbp1CKO + DRP-104

Supplementary Fig. 6 | Glutamine produced by IRE1α/XBP1-deficient hepatocytes sustains IM fitness.

a, Glutamine levels in supernatants from hepatocytes infected with WT or mutant XBP1s-expressing or control adenoviruses (n = 3 per group). **b,** Schematic representation of the in vitro culture system for BMIMs. **c,** Flow cytometric analysis of BMIMs stimulated with conditioned media from *Ern1*$^{fl/fl}$ or *Ern1*^{CKO} hepatocytes, with or without L-asparaginase (L-Asp) treatment, showing the MFI of Ki67 in F4/80^{+} macrophages (n = 3 per group). **d,** Flow cytometric analysis of BMIMs exposed to graded concentrations of glutamine, showing the MFI of Ki67 in F4/80^{+} macrophages (n = 3 per group). **e,** Intracellular glutamine levels of BMIMs as measured by ELISA (n = 5 per group). **f,** Flow cytometric analysis of Ki67 MFI in BMIMs under indicated glutamine conditions. **g,** Volcano plots depicting differentially expressed genes in Ly6C^{lo}F4/80^{+} macrophages cultured under glutamine-replete (0.6 mM) versus glutamine-restricted (0.05 mM) conditions. **h,** Flow cytometric analysis of IMs in DRP-104-treated MC38-bearing *Xbp1*$^{fl/fl}$ and *Xbp1*^{CKO} mice (n = 5 per group). Data are represented as mean ± SEM and analyzed by one-way ANOVA (a, c–f, h). Some elements in b were created by Figdraw (www.figdraw.com). Source data are provided as a Source Data file.

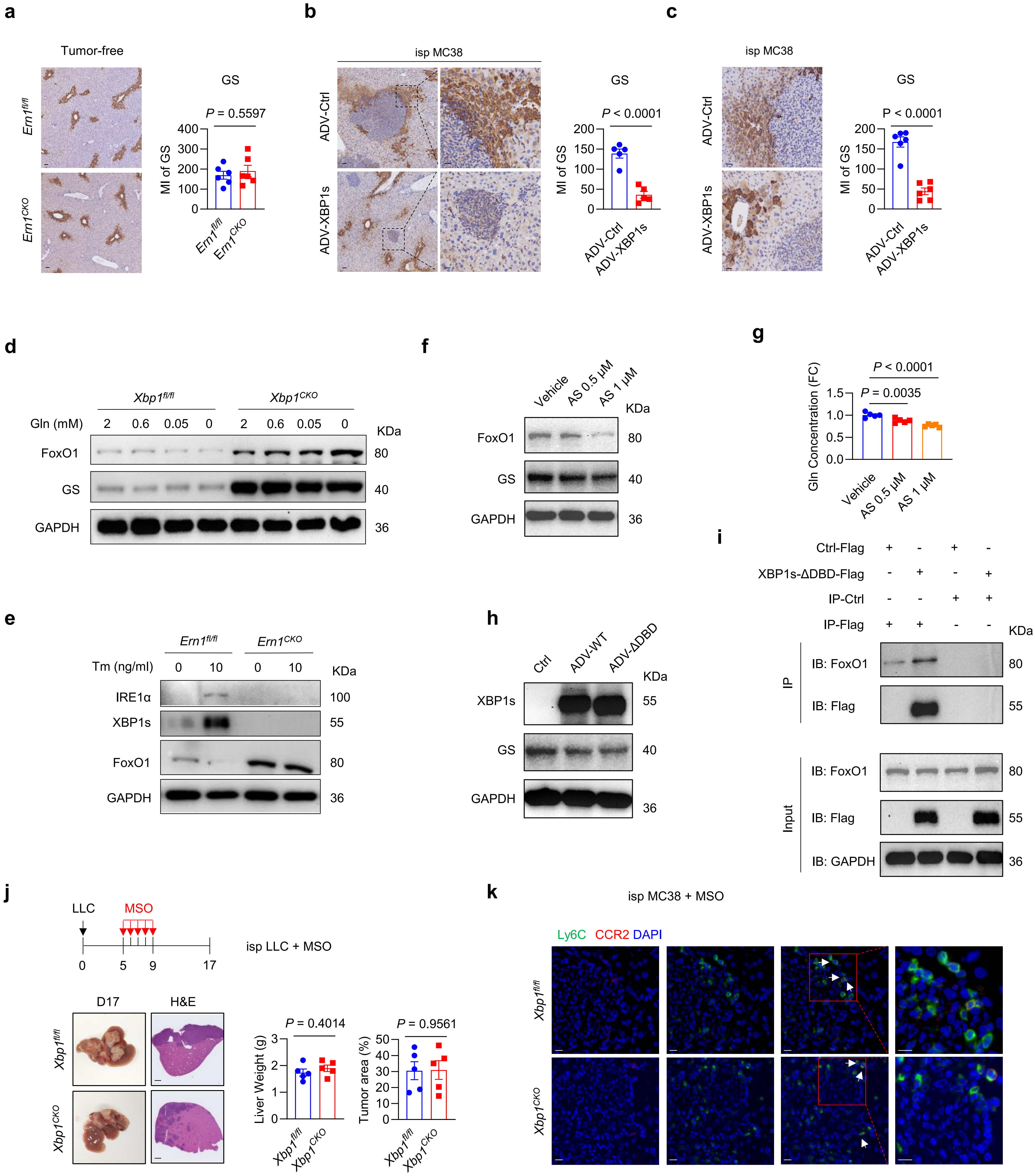
a
Tumor-free
Ern1fl/fl
Ern1CKO
GS
P = 0.5597
MI of GS
b
isp MC38
ADV-Ctrl
ADV-XBP1s
P < 0.0001
c
d
Xbp1fl/fl
Xbp1CKO
Gln (mM)
FoxO1
GAPDH
KDa
e
Tm (ng/ml)
IRE1α
XBP1s
f
Vehicle
AS 0.5 μM
AS 1 μM
g
Gln Concentration (FC)
P = 0.0035
h
Ctrl
ADV-WT
ADV-ΔDBD
i
Ctrl-Flag
XBP1s-ΔDBD-Flag
IP-Ctrl
IP-Flag
IP
Input
IB: FoxO1
IB: Flag
IB: GAPDH
j
LLC
MSO
isp LLC + MSO
D17
H&E
Liver Weight (g)
Tumor area (%)
P = 0.4014
P = 0.9561
k
isp MC38 + MSO
Ly6C CCR2 DAPI

Supplementary Fig. 7 | Hepatocyte GS plays a critical role in IRE1α/XBP1 depletion-related pro-metastatic niche formation.

a, Representative GS immunohistochemistry images of naïve *Ern1*$^{fl/fl}$ and *Ern1*^{CKO} mouse livers (n = 6 per group). **b,** Representative GS immunohistochemistry images of liver sections from MC38-bearing WT mice that were pretreated with XBP1s-overexpressing or control adenoviruses (n = 5 per group). **c,** Representative GS immunohistochemistry images of liver sections from MC38-bearing *Ern1*^{CKO} mice that were pretreated with XBP1s-overexpressing or control AAVs (n = 6 per group). **a–c,** Scale bars, 50 μm (left) or 20 μm (right). Zone 3 GS expression was excluded for quantification. **d,** Immunoblotting analysis of *Xbp1*$^{fl/fl}$ and *Xbp1*^{CKO} hepatocytes cultured under indicated concentrations of glutamine. The samples derive from the same experiment but different gels for FoxO1 and GAPDH, another for GS was processed in parallel. The similar strategy applies to (f and h). Representative results from three independent experiments are shown. **e,** Immunoblotting analysis of *Ern1*$^{fl/fl}$ and *Ern1*^{CKO} hepatocytes cultured under indicated concentrations of tunicamycin (Tm). The samples derive from the same experiment but different gels for FoxO1 and GAPDH, another for GS and another for XBP1s were processed in parallel. Representative results from three independent experiments are shown. **f, g,** Immunoblotting analysis (f) and measurement of supernatant glutamine levels (g) in *Xbp1*^{CKO} hepatocyte treated with the indicated concentrations of AS1842856 (n = 3 per group). Representative results from three independent experiments are shown. **h,** Immunoblotting analysis of hepatocytes infected with WT or mutant XBP1s-expressing or control adenoviruses. Representative results from three independent experiments are shown. **i,** Immunoprecipitates from hepatocytes overexpressed with mutant XBP1s or a control construct were analyzed by immunoblotting. The samples derive from the same experiment but different gels for FoxO1 and GAPDH, another for Flag was processed in parallel. Representative results from three independent experiments are shown. **j,** Metastatic burden in LLC-bearing *Xbp1*$^{fl/fl}$ and *Xbp1*^{CKO} mice treated with MSO (n = 5 per group). Scale bars,1000 μm. **k,** Representative immunofluorescence staining of CCR2^{+}Ly6c^{+} cells (white arrows) in MC38-bearing *Xbp1*$^{fl/fl}$ and *Xbp1*^{CKO} mice treated with MSO. Scale bars, 10 μm. Data are represented as mean ± SEM and analyzed by two-tailed, unpaired Student's t test (a, b, c and j). Source data are provided as a Source Data file.

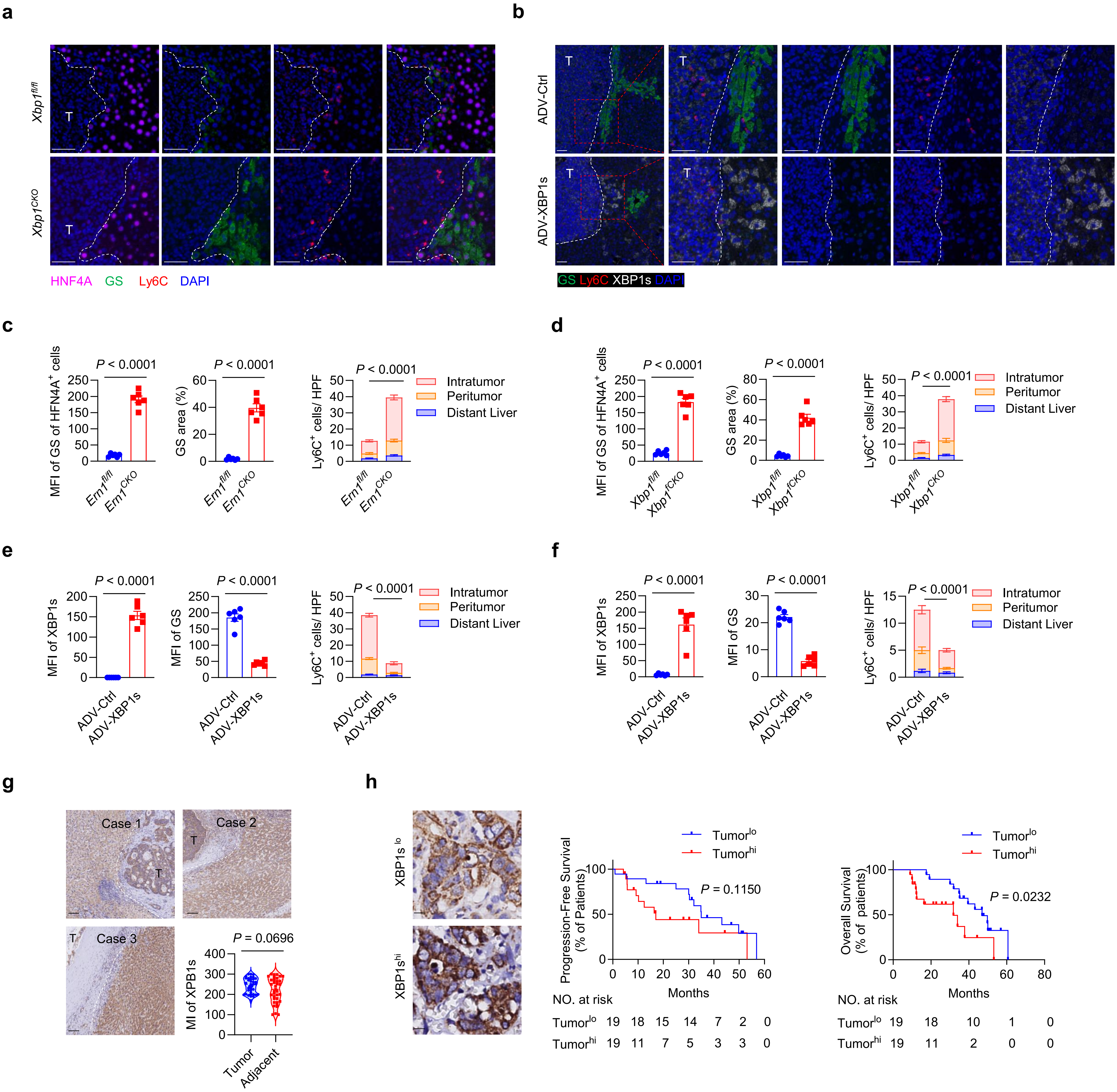

a
b
HNF4A GS Ly6C DAPI
GS Ly6C XBP1s DAPI
ADV-Ctrl
ADV-XBP1s
c
d
e
f
Intratumor
Peritumor
Distant Liver
P < 0.0001
g
Case 1
Case 2
Case 3
P = 0.0696
MI of XPB1s
Tumor
Adjacent
h
Tumor^lo
Tumor^hi
P = 0.1150
P = 0.0232
Progression-Free Survival (% of Patients)
Overall Survival (% of patients)
Months
NO. at risk

Supplementary Fig. 8 | Immunobiological significance of hepatocyte IRE1α–XBP1s axis in liver metastasis.

a, b, Representative multiplex immunofluorescence images of day 7 MC38-bearing livers from *Xbp1*$^{fl/fl}$ and *Xbp1*^{CKO} mice (a) or WT mice pretreated with XBP1s-overexpressing or control AAVs (b). Scale bars, 50 μm. T, tumor. **c–f,** Quantification of immunofluorescence data on metastasis-bearing liver sections from *Ern1*$^{fl/fl}$ and *Ern1*^{CKO} (c), *Xbp1*$^{fl/fl}$ and *Xbp1*^{CKO} mice (d) and AAV-treated *Ern1*^{CKO} (e) and WT (f) mice. Bar graphs show the MFI of GS or XBP1s (left), GS area (middle), and Ly6C^{+} cell counts per HPF across different liver zones (right) (n = 6 per group). Zone 3 GS expression was excluded for quantification. **g,** Representative immunohistochemistry images (left) and quantitative comparison (right) of XBP1s expression between metastatic tumor and adjacent liver tissue from CRLM patients (n = 38 per group). Scale bars, 100 μm. T, tumor. **h,** Kaplan–Meier analysis of progression-free survival and overall survival in CRLM patients based on XBP1s expression in tumor areas (n = 19 per group). Data are represented as mean ± SEM. Statistical analysis was performed using two-tailed, unpaired Student's t test (**c–g**) and log-rank test (**h**). Source data are provided as a Source Data file.

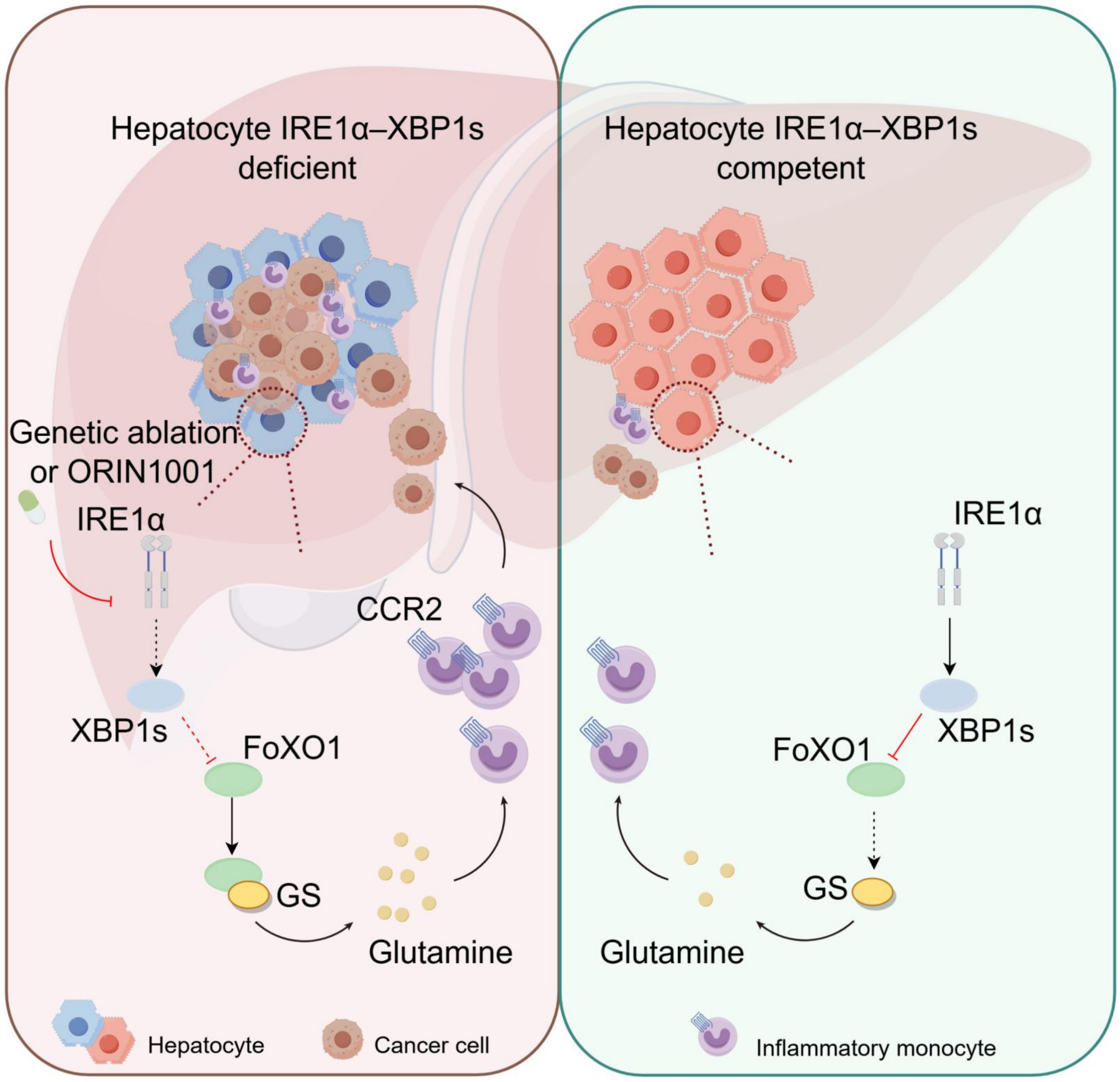

Hepatocyte IRE1α–XBP1s deficient
Genetic ablation or ORIN1001
IRE1α
XBP1s
FoXO1
GS
Glutamine
CCR2
Hepatocyte IRE1α–XBP1s competent
IRE1α
XBP1s
FoXO1
GS
Glutamine
Hepatocyte
Cancer cell
Inflammatory monocyte

Supplementary Fig. 9 | Schematic diagram of the immunobiological role of the hepatocyte IRE1α–XBP1s axis in liver metastasis.

Deficiency of the hepatocyte IRE1α–XBP1 axis activates the intrinsic FoxO1–GS pathway, resulting in increased glutamine production. This glutamine-rich liver microenvironment promotes the expansion of pro-metastatic inflammatory monocytes and facilitates metastatic progression. Illustrations were created using Figdraw (https://www.figdraw.com).
