# Supplementary material for: Hepatocyte IRE1α–XBP1s axis prevents pro-metastatic niche formation through metabolic rewiring of inflammatory monocytes
Source: Nat Commun. 2026 Nov 3;17:10961. doi: 10.1038/s41467-026-78812-2 (PMC13731655; doi:10.1038/s41467-026-78812-2)
Supplement: Supplementary file 2 [file 41467_2026_78812_MOESM2_ESM.pdf]

Description of Additional Supplementary Files

Supplementary Data 1 | Endogenous metabolites accumulated in peritumor *Ern1*$^{fl/fl}$ (n = 3) and *Ern1*^{CKO} (n = 3) livers. Differential metabolites in peritumor liver tissues evaluated by LC–MS analysis, with *P*-values determined using an unpaired, two-sided Student's t-test.

Supplementary Data 2 | Endogenous metabolites accumulated in tumor-free *Ern1*$^{fl/fl}$ (n = 3) and *Ern1*^{CKO} (n = 4) livers. Differential metabolites in naïve liver tissues evaluated by LC–MS analysis, with *P*-values determined using an unpaired, two-sided Student's t-test without multiple comparison adjustments.

Supplementary Data 3 | Glutamine-regulated genes in in-vitro-generated IMs and macrophages. Differential expression of genes evaluated by DESeq2 analysis, with statistical significance and Wald test *P*-values adjusted using the Benjamini–Hochberg False Discovery Rate (FDR) method (*P* adj).

Supplementary Data 4 | Clinicopathological information of 38 CRLM patients.
