# Supplementary material for: Hepatocyte IRE1α–XBP1s axis prevents pro-metastatic niche formation through metabolic rewiring of inflammatory monocytes
Source: Nat Commun. 2026 Nov 3;17:10961. doi: 10.1038/s41467-026-78812-2 (PMC13731655; doi:10.1038/s41467-026-78812-2)
Supplement: Supplementary file 3 [file 41467_2026_78812_MOESM7_ESM.pdf]

nature portfolio

Corresponding author(s): H.Z. and J.H.

Last updated by author(s): Sep 14, 2026

Reporting Summary

Nature Portfolio wishes to improve the reproducibility of the work that we publish. This form provides structure for consistency and transparency in reporting. For further information on Nature Portfolio policies, see our Editorial Policies and the Editorial Policy Checklist.

Statistics

For all statistical analyses, confirm that the following items are present in the figure legend, table legend, main text, or Methods section.

n/a	Confirmed	
☐	☒	The exact sample size (*n*) for each experimental group/condition, given as a discrete number and unit of measurement
☐	☒	A statement on whether measurements were taken from distinct samples or whether the same sample was measured repeatedly
☐	☒	The statistical test(s) used AND whether they are one- or two-sided<br>*Only common tests should be described solely by name; describe more complex techniques in the Methods section.*
☒	☐	A description of all covariates tested
☐	☒	A description of any assumptions or corrections, such as tests of normality and adjustment for multiple comparisons
☐	☒	A full description of the statistical parameters including central tendency (e.g. means) or other basic estimates (e.g. regression coefficient) AND variation (e.g. standard deviation) or associated estimates of uncertainty (e.g. confidence intervals)
☐	☒	For null hypothesis testing, the test statistic (e.g. *F*, *t*, *r*) with confidence intervals, effect sizes, degrees of freedom and *P* value noted<br>*Give P values as exact values whenever suitable.*
☒	☐	For Bayesian analysis, information on the choice of priors and Markov chain Monte Carlo settings
☒	☐	For hierarchical and complex designs, identification of the appropriate level for tests and full reporting of outcomes
☐	☒	Estimates of effect sizes (e.g. Cohen's *d*, Pearson's *r*), indicating how they were calculated

Our web collection on statistics for biologists contains articles on many of the points above.

Software and code

Policy information about availability of computer code

Data collection	Standard software provided by instrument manufacturers was used for data acquisition:<br>• Histology: NanoZoomer® S360 (Hamamatsu Photonics) and Pannoramic MIDI scanner (3DHISTECH)<br>• Western blot: ChemiDoc Touch Imaging System (Bio-Rad)<br>• Flow cytometry (FACS) analysis and cell sorting: Cytek Aurora (Cytek), CytoFLEX (Beckman), and BD FACSAria II (BD Biosciences)<br>• Single-cell library preparation: Chromium Controller with Chromium Next GEM Single Cell 3' Kit v2 and associated Library Construction Kit (10x Genomics)<br>• Next-generation sequencing: NovaSeq 6000 (Illumina)<br>• Liquid chromatography–mass spectrometry (LC–MS): Agilent 1290 system (Agilent) coupled with a TripleTOF™ 6600plus mass spectrometer (AB SCIEX)
Data analysis	The following software tools were used for data analysis and visualization:Flow cytometry (FACS): FlowJo v10.8.1 (Treestar)Statistical analysis: R v4.2.2 and GraphPad Prism v9.5.1Single-cell RNA sequencing (scRNA-seq): CellRanger v7.2.0, Seurat R package v4.3.0, and Monocle R package v2.28.0Imaging analysis: CaseViewer v2.3.0 (3DHISTECH) and ImageJ (National Institutes of Health, USA)

For manuscripts utilizing custom algorithms or software that are central to the research but not yet described in published literature, software must be made available to editors and reviewers. We strongly encourage code deposition in a community repository (e.g. GitHub). See the Nature Portfolio guidelines for submitting code & software for further information.

Data

Policy information about availability of data

All manuscripts must include a data availability statement. This statement should provide the following information, where applicable:
- Accession codes, unique identifiers, or web links for publicly available datasets
- A description of any restrictions on data availability
- For clinical datasets or third party data, please ensure that the statement adheres to our policy

Data for single-cell RNA-seq and bulk RNA-seq are available through the BioProject portal (BioProject ID: PRJNA1322094). All other data are available from the corresponding author on reasonable request.

Research involving human participants, their data, or biological material

Policy information about studies with human participants or human data. See also policy information about sex, gender (identity/presentation), and sexual orientation and race, ethnicity and racism.

Reporting on sex and gender	Sex and gender were not considered in the study design or analysis
Reporting on race, ethnicity, or other socially relevant groupings	Not applicable. No race, ethnicity, or other socially relevant variables were collected or analyzed in this study.
Population characteristics	Clinical parameters of the patients are presented in the Supplementary Table2.
Recruitment	Data were obtained under the approval of Sun Yat-sen University Cancer Center.
Ethics oversight	The study was approved by the Medical Ethics Committee of Sun Yat-sen University Cancer Center. Written informed consent was obtained from the patients who donated samples.

Note that full information on the approval of the study protocol must also be provided in the manuscript.

Field-specific reporting

Please select the one below that is the best fit for your research. If you are not sure, read the appropriate sections before making your selection.

☒ Life sciences ☐ Behavioural & social sciences ☐ Ecological, evolutionary & environmental sciences

For a reference copy of the document with all sections, see nature.com/documents/nr-reporting-summary-flat.pdf

Life sciences study design

All studies must disclose on these points even when the disclosure is negative.

Sample size	No statistical methods were used to predetermine the sample size. Sample sizes were determined based on feasibility, pilot experiments, and previously published literature.
Data exclusions	No data were excluded from analyses.
Replication	Experiments were conducted and successfully replicated with sufficient numbers of biological replicates as indicated in the Figure Legends section. All experiments were successfully repeated at least three times.
Randomization	Randomization was not required in this study because there is no statistic that requires randomization of samples. For all animal experiments, control or knock-out mice were randomly selected from the littermates. For other experiments, all samples were treated in the same way to decrease the variability.
Blinding	The experiments were not performed blindly due to the researchers needed to verify the genotypes of mice.

Reporting for specific materials, systems and methods

We require information from authors about some types of materials, experimental systems and methods used in many studies. Here, indicate whether each material, system or method listed is relevant to your study. If you are not sure if a list item applies to your research, read the appropriate section before selecting a response.

Materials & experimental systems

n/a	Involved in the study
☐	☒ Antibodies
☐	☒ Eukaryotic cell lines
☒	☐ Palaeontology and archaeology
☐	☒ Animals and other organisms
☐	☒ Clinical data
☒	☐ Dual use research of concern
☒	☐ Plants

Methods

n/a	Involved in the study
☒	☐ ChIP-seq
☐	☒ Flow cytometry
☒	☐ MRI-based neuroimaging

Antibodies

Antibodies used

TruStain FcX™ PLUS (anti-mouse CD16/32) Antibody (Clone S17011E) BioLegend Cat# 156603
FITC anti-mouse Ly-6c (Clone HK1.4) BioLegend Cat# 128005
APC anti-mouse F4/80 (Clone BM8) BioLegend Cat# 123115
APC Fire750 anti-mouse Ly6G (Clone 1A8) BioLegend Cat# 127652
Brilliant Violet 421™ anti-mouse/human CD11b (Clone M1/70) BioLegend Cat# 101251
PE/Cyanine7 anti-mouse CD45 (Clone 30-F11) BioLegend Cat# 103114
PerCP/Cyanine5.5 anti-mouse CD3ε (Clone 145-2C11) BioLegend Cat# 100328
Alexa Fluor 700 anti-mouse CD4 (Clone RM4-5) BioLegend Cat# 100536
FITC anti-mouse Ki67(Clone 11F6) BioLegend Cat# 151211
PerCP/Cy5.5 anti-mouse CD11c(Clone N418) BioLegend Cat# 117327
Brilliant Violet 421™ anti-mouse TCR γ/δ Antibody (Clone GL3) BioLegend Cat# 118120
Brilliant Violet 510™ anti-mouse CD45 (Clone 30-F11) BioLegend Cat# 103138
PE anti-mouse/rat/human FOXP3 (Clone 150D) BioLegend Cat# 320008
PE/Dazzle™ 594 anti-mouse CD8a (Clone 53-6.7) BioLegend Cat# 100761
PE/Cyanine7 anti-mouse NK-1.1 Antibody (Clone S17016D) BioLegend Cat# 156513
APC anti-mouse Ki-67 (Clone 16A8) BioLegend Cat# 652405
Brilliant Violet 510™ anti-mouse F4/80 (Clone BM8) BioLegend Cat# 123135
PE anti-mouse CD192(CCR2)(Clone SA203G11) BioLegend Cat# 150609
FITC anti-mouse CD45.1(Clone A20) BioLegend Cat#110705
APC/Cyanine7 anti-mouse CD45.2 (Clone 104) BioLegend Cat#109824
Alexa Fluor 700 anti-mouse I-A/I-E(Clone M5/114.15.2)" BioLegend Cat#107621
BV650 anti-mouse CD206(Clone C068C2) BioLegend Cat#141723
Arginase 1 Monoclonal Antibody (Clone A1exF5) PE eBioscience Cat#12-3697-82
APC anti-mouse CD80 (Clone 16-10A1) BioLegend Cat#104713
anti-IRE1α (p-S724) antibody Abcam, Cat# ab48187
Rabbit anti-IRE1 antibody (clone 14C10) Cell Signaling Technology Cat# 3294
Rabbit anti-XBP-1s antibody (clone E9V3E) Cell Signaling Technology Cat# 40435
Rabbit anti-FoxO1 antibody (clone C29H4) Cell Signaling Technology Cat# 2880
Rabbit anti-DYKDDDDK Tag (Flag) antibody (clone D6W5B) Cell Signaling Technology Cat# 14793
Rabbit anti-IRE1 (phospho S724) antibody Abcam Cat# ab48187
Rabbit anti-Glutamine Synthetase antibody Abcam Cat# ab228590
HRP-conjugated Goat Anti-Rabbit IgG(H+L) Proteintech Cat# SA00001-2
Rabbit anti-Lamin B1 Polyclonal antibody Proteintech Cat# 12987-1-AP
Mouse anti-HRP-conjugated GAPDH antibody (clone 1A6) YEASEN Cat#30203E350
Anti Flag Nanobody Agarose Beads AlpaLifeBio Cat# KTSM1308
Negative Control Agarose Beads AlpaLifeBio Cat# KTSM1356
Rabbit anti-Ly6c antibody (clone EPR27220-23) Abcam Cat# ab314120
Rabbit anti-CCR2 antibody (clone EPR20844-15) Abcam Cat# ab273050
Rabbit anti-Ki67 antibody (clone D3B5) Cell Signaling Technology Cat# 9129
XBP1S-specific Recombinant antibody (clone 241004B6) Proteintech Cat# 83959-5-RR
Rabbit anti-CD14 antibody (clone D7A2T) Cell Signaling Technology Cat#75181S
Goat Anti-Rabbit IgG H&L (HRP) Abcam Cat#ab205718
iFluor® 430 tyramide AAT Bioquest Cat#45096
iFluor® 488 tyramide AAT Bioquest Cat#11060
iFluor® 555 tyramide AAT Bioquest Cat#45105
iFluor® 647 tyramide AAT Bioquest Cat#45110
InVivoMAb mouse IgG2a isotype control (clone C1.18.4) BioXCell Cat#BE0085
InVivoMAb anti-mouse NK1.1 (clone PK136) BioXCell Cat#BE0036
InVivoMAb anti-mouse CD8α (clone 2.43) BioXCell Cat#BE0061
InVivoMAb anti-mouse Ly6C (clone Montd 1) BioXCell Cat#BE0203

Validation

All antibodies were obtained from reputable vendors. Refer to their websites for validation data and relevant citations for the species and application used in this study.

Eukaryotic cell lines

Policy information about cell lines and Sex and Gender in Research

Cell line source(s)	The MC38 cell line was originally from Prof. J. Yan laboratory (Xuzhou Medical University). The LLC cell line (ATCC, CRL-1642) was purchased from the American Type Culture Collection.
Authentication	All cells were authenticated by STR.
Mycoplasma contamination	All cell lines were routinely detected and had negative results for mycoplasma.
Commonly misidentified lines (See ICLAC register)	None.

Animals and other research organisms

Policy information about studies involving animals; ARRIVE guidelines recommended for reporting animal research, and Sex and Gender in Research

Laboratory animals	Wild-type (WT) C57BL/6J mice were purchased from BesTest Bio-Tech (Zhuhai, China). Ern1fl/fl and Xbp1fl/fl mice on a C57BL/6J background were generously provided by Profs. Y. Liu (Wuhan University) and L.H. Glimcher (Dana Farber Cancer Institute). AlbCre transgenic mice were obtained from The Jackson Laboratory (003574). To achieve hepatocyte-specific gene depletion, Ern1fl/fl and Xbp1fl/fl mice were crossed with AlbCre mice, yielding Ern1CKO and Xbp1CKO mice, respectively. For experiments involving Ern1CKO or Xbp1CKO mice, littermate controls expressing WT ERN1 (Ern1fl/fl) or XBP1 (Xbp1fl/fl) were used. All mice were housed in a specific pathogen-free facility maintained at 22–23°C on a 12-h light/dark cycle, with ad libitum access to food and water. Mice aged 8–12 weeks were used for all experiments. Animals were randomly assigned to experimental groups.
Wild animals	Our study did not involve wild animals.
Reporting on sex	Mice of both sexes were included in this study.
Field-collected samples	This study does not involve field-collected samples.
Ethics oversight	All procedures were conducted in accordance with protocols approved by the Research Ethics Committee of the University of Macau.

Note that full information on the approval of the study protocol must also be provided in the manuscript.

Clinical data

Policy information about clinical studies

All manuscripts should comply with the ICMJE guidelines for publication of clinical research and a completed CONSORT checklist must be included with all submissions.

Clinical trial registration	*Provide the trial registration number from ClinicalTrials.gov or an equivalent agency.*
Study protocol	*Note where the full trial protocol can be accessed OR if not available, explain why.*
Data collection	*Describe the settings and locales of data collection, noting the time periods of recruitment and data collection.*
Outcomes	*Describe how you pre-defined primary and secondary outcome measures and how you assessed these measures.*

Plants

Seed stocks	*Report on the source of all seed stocks or other plant material used. If applicable, state the seed stock centre and catalogue number. If plant specimens were collected from the field, describe the collection location, date and sampling procedures.*
Novel plant genotypes	*Describe the methods by which all novel plant genotypes were produced. This includes those generated by transgenic approaches, gene editing, chemical/radiation-based mutagenesis and hybridization. For transgenic lines, describe the transformation method, the number of independent lines analyzed and the generation upon which experiments were performed. For gene-edited lines, describe the editor used, the endogenous sequence targeted for editing, the targeting guide RNA sequence (if applicable) and how the editor was applied.*
Authentication	*Describe any authentication procedures for each seed stock used or novel genotype generated. Describe any experiments used to assess the effect of a mutation and, where applicable, how potential secondary effects (e.g. second site T-DNA insertions, mosiacism, off-target gene editing) were examined.*

Flow Cytometry

Plots

Confirm that:

☒ The axis labels state the marker and fluorochrome used (e.g. CD4-FITC).

☒ The axis scales are clearly visible. Include numbers along axes only for bottom left plot of group (a 'group' is an analysis of identical markers).

☒ All plots are contour plots with outliers or pseudocolor plots.

☒ A numerical value for number of cells or percentage (with statistics) is provided.

Methodology

Sample preparation	Livers were harvested from mice as described in the Fig. legends. Tissue samples were minced and enzymatically dissociated using the Mouse Liver Dissociation Kit (130-105-807, Miltenyi) in conjunction with the gentleMACS Octo Dissociator with Heaters (Miltenyi), following the manufacturer's protocol. The resulting cell suspension was filtered through a 70-µm cell strainer (Miltenyi), then subjected to a 40% Percoll gradient and centrifuged at 900 × g for 20 minutes at room temperature to enrich immune cells. Red blood cells were lysed using RBC lysis buffer (BioLegend), and the remaining cells were resuspended in staining buffer (PBS supplemented with 2% FBS and 1 mM EDTA). To block Fc receptors, cells were incubated with TruStain FcX™ PLUS (anti-mouse CD16/32) Antibody (Clone S17011E, BioLegend, 156603) for 10 minutes. Subsequently, surface staining was performed by incubating cells with specific antibodies for 30 minutes at 4°C in the dark. For CellTrace™ Violet (Thermo Fisher) (CTV) staining, cells were incubated with 5 µM CTV in RPMI medium for 20 minutes at 37°C following surface marker staining. For intracellular staining, cells were fixed, and permeabilized using the Foxp3/Transcription Factor Staining Buffer Set (eBioscience) according to the manufacturer's instructions, then stained with intracellular or isotype control antibodies. Flow cytometric analysis was performed using either the Cytek Aurora (Cytek) or CytoFLEX (Beckman) instruments. Data were analyzed using FlowJo v10.8.1 (Treestar). Dead cells stained with Zombie NIR (BioLegend, 423106) or Zombie Aqua (BioLegend, 423102) were excluded from analysis. Gating strategies were validated using fluorescence-minus-one (FMO) controls for low-density antigens.
Instrument	Cytek Aurora (Cytek) , CytoFLEX (Beckman), BD FACSAria™ II
Software	Flowjo (Treestar)
Cell population abundance	A minimum of one million viable immune cells were recorded per sample during analysis. The purity of the sorted cells exceeded 95%.
Gating strategy	All samples were initially gated using forward and side scatter parameters to identify cellular events. Single cells were then enriched by gating forward scatter height versus area, followed by selection of viable cells based on exclusion of the viability dye. The subsequent gating strategy is illustrated in the figures.

☒ Tick this box to confirm that a figure exemplifying the gating strategy is provided in the Supplementary Information.
