# Supplementary material for: Hepatocyte IRE1α–XBP1s axis prevents pro-metastatic niche formation through metabolic rewiring of inflammatory monocytes
Source: Nat Commun. 2026 Nov 3;17:10961. doi: 10.1038/s41467-026-78812-2 (PMC13731655; doi:10.1038/s41467-026-78812-2)
Supplement: Supplementary file 4 [file 41467_2026_78812_MOESM8_ESM.pdf]

nature portfolio

Peer Review File

Hepatocyte IRE1α–XBP1s axis prevents pro-metastatic niche formation through metabolic rewiring of inflammatory monocytes

Corresponding Author: Dr Jiajie Hou

This file contains all reviewer reports in order by version, followed by all author rebuttals in order by version.

Version 0:

Reviewer comments:

Reviewer #1

(Remarks to the Author)
This manuscript reports the mechanistic investigation of hepatocyte IRE1α-XBP1s axis in suppressing local secondary inflammation, thereby inhibiting hepatic metastasis of tumor cells. Through a combination of genetically engineered mouse models and biochemistry approaches, the authors provided evidence showing that XBP1s suppresses glutamine synthesis in hepatocytes via negative regulation of the FoXO1-GS pathway, which limits monocyte expansion and subsequent tumor cell colonization in the liver. The findings reveal a crucial mechanism by which the IRE1α–XBP1s axis modulates hepatocyte–monocyte interactions, suggesting a potential therapeutic strategy against hepatic metastasis.
Overall, this study was well executed, and the presented data clearly provided an interesting mechanism by which the IRE1α-XBP1s pathway regulates hepatocyte-monocyte crosstalk in suppressing hepatic metastasis. Some minor points can be considered for further enhancing the conclusions as follows:
1. How the IRE1α-XBP1 pathway in hepatocytes changes at different time points (D2、D4、D7) after isp remains unclear, which should be analyzed in parallel with the FoXO1-GS pathway.
2. The upregulation of FoXO1 in in vitro-cultured Ern1CKO mouse hepatocytes does not appear to be significant in Figure 6c. Quantitative analysis is required to support this conclusion. Protein expression levels of IRE1α and XBP1s should also be shown. Additionally, it is necessary to assess FoXO1 and GS expression in XBP1CKO mouse hepatocytes.
3. In Extended Data Figure 4e, MKC8866 treatment led to the accumulation of CCR2+ inflammatory monocytes in the wild-type metastasis-bearing livers. And yet it did not appear to affect MC38 liver metastasis as shown in Figure 1b. This discrepancy should be clarified.
4. The H&E staining in Figure 1c requires a representative flox control tissue section showing more comprehensive liver analysis.
5. AV- or AAV-mediated restoration of XBP1s expression in IRE1α-CKO livers can be employed to confirm the results in vivo, including the effects on the FoXO1-GS pathway along with the hepatic metastasis.
6. What may underlie the regulation by hepatocyte IRE1α of the FoXO1-GS pathway in the context of hepatic metastasis instead of HCC development? May some factors from metastatic tumor cells be involved in specifically activating the IRE1α branch or inducing ER stress to activate all the UPR branches?

Reviewer #2

(Remarks to the Author)
The manuscript entitled “Hepatocyte IRE1α–XBP1s axis prevents pro-metastatic niche formation through metabolic rewiring of inflammatory monocytes” by Zhang, Lian, Chen, Yang, Fu, et al, aims to determine how hepatocyte-specific IRE1α–XBP1s axis affects liver tumor progression. Results show that inhibition of IRE1α impairs the growth of subcutaneous tumors but conversely increase their progression in models of metastatic hepatic burdens. Conditional IRE1α–XBP1s deletion in hepatocytes inhibits liver metastasis by promoting the expansion of CCR2+ inflammatory monocytes (IMs). Conversely, hepatocytes specific XBP1s-overexpression before tumor inoculation reduced metastatic lesions. Notably, glutamine availability was increased in the peritumor areas of hepatocyte IRE1α–XBP1s deficient mice compared to controls, which promoted tumor metastasis growth via glutamine transporter SLC38A2. Mechanistically, IRE1α- or XBP1-null hepatocytes increase the expression of FoxO1-regulated glutamine synthetase (GS), which primes the secretion of glutamine to support CCR2+ IM fitness. As such, rescue expression of hepatocyte XBP1s or blockade of glutamine metabolism effectively

reduces IMs and mitigates metastasis progression. Validation of the mouse data in liver cancer patient settings include results showing that liver metastases positively correlates with higher XBP1s expression in hepatocyte. Together, results suggest that Hepatocyte IRE1α–XBP1s limits metastasis by impairing glutamine availability for IMs. Results are timely and highlights an unexpected role of IRE1α–XBP1s in liver tumors and in the regulation of monocyte function. However, there were multiple limitations that prevented the full support to the report. Comments are as following:

Major Comments:

1) Role of Hepatocyte IRE1α–XBP1s in metastasis of spontaneous tumor models
Report shows an antitumor effect of IRE1α RNase inhibitor MKC8866 (ORIN1001) in subcutaneous LLC and MC-38 models, but an opposite effect after intra-splenic injection of the tumors (liver tumor growth). To provide clarity, it would be interesting to test the effect of this inhibitor in spontaneous tumor models that metastasize to the liver.

2) Metastatic burden models
The models used for metastatic burdens are artificial by nature. Validation of the studies in models showing spontaneous metastasis in the liver would be primary.

3) Glutamine as a mechanistic mediator
It would be important to determine whether catabolism of glutamine via glutaminase could overcome the pro-metastatic and high frequency of iMs phenotype observed in hepatocyte-specific IRE1α- or XBP1-null mice bearing tumors. This could complement the results showing that glutamine antagonism abolished the metastasis supporting effect of IRE1α or XBP1 depletion.

4) Glutamine role outside of metastatic tumors
Glutamine uptake inhibition alters the growth of tumor cells in the liver, but could also impact growth outside of the liver. Thus, it would be important to evaluate the importance of glutamine uptake by cancer cells in primary tumor growth. Therefore, is the effect of SLC38A2 only observed in metastasis settings, or also in the tumors injected sc?

5) How does CCR2+ iMs promote metastasis
The mechanisms whereby CCR2+ IMs support metastasis expansion remain unclear. Further characterization of these processes and identification of major functional differences would be highly impactful. Also, validation of the inflammatory and pro-tumor nature of the iMs would be needed.

6) How does glutamine support the activity of iMs?

7) Rationale and interpretation of Fig. 1e–f
The rationale for including Fig. 1e and 1f remains unclear. Specifically, the early administration of glutamine (Gln) failed to induce tumor progression without explaining the possible cause. Additionally, the decision to assess endpoints two days after Gln administration (Fig. 1f) rather than on Day 14 post-tumor implantation, as done in earlier experiments, requires further justification.

8) Lack of tumor burden comparison between sgCtrl and sgSLC38A2
The authors did not provide a direct comparison of tumor burden between sgCtrl and sgSLC38A2 groups. Therefore, the claim that "SLC38A2 deficiency profoundly inhibited metastasis formation" is not adequately supported by the presented data.

9) Interpretation of monocyte-depletion experiments (Fig. 2)
If tumor-intrinsic glutamine uptake drives tumor proliferation, as proposed, it is unclear why monocyte depletion using anti-Ly6C or pharmacologic CCR2 inhibition (RS102895) completely abolishes the ERN1 CKO associated increase in tumor burden, rather than producing only a partial effect. This questions whether the previous proposed mechanisms are relevant drivers of the found phenotype.

10) MKC8866 combination treatments
Does combining MKC8866 with DRP-104 or RS102895 re-sensitizes tumor cells to MKC8866, which would strengthen the mechanistic interpretation.

11) Flow cytometry issues in Extended Fig. 4f
In Extended Fig. 4f, the CTV-labeled cells are not clearly distinguishable from host cells. The authors should optimize CTV labeling intensity or use congenic markers (e.g., CD45.1/CD45.2) and GFP expressing cells that used in later part of the story to ensure reliable discrimination. Additionally, it would be important to determine whether ERN1 CKO mice receiving labeled $Ly6C^+$ cells similarly show an increased $Ki67^+CCR2^+$ population found in XBP1 CKO mice.

12) Overinterpretation of inflammatory phenotype (Fig. 4f–g)
The authors measure Ki67 as a marker of monocyte proliferation but do not assess additional inflammatory markers. Therefore, the statement that "extracellular glutamine… is an upstream driver of monocyte transitioning into an inflammatory state" should be moderated to reflect the limited scope of the data.

13) GS protein analysis (Fig. 6c)
Fig. 6c should include either a shorter WB exposure for GS or densitometric quantification to support the conclusion that GS

expression increases under low extracellular Gln.

14) Inclusion of XBP1s KO in Fig. 6c
Including XBP1s KO in Fig. 6c would strengthen the conclusion that XBP1s-deficient conditions lead to elevated FOXO1 compared to WT controls.

15) XBP1s overexpression rescue experiment
The authors should evaluate whether XBP1s overexpression (via ADV-XBP1s) in ERN1 KO hepatocytes reverses monocyte accumulation and tumor progression. This model may also be more suitable for immunofluorescence imaging, as ERN1 KO mice exhibit elevated Ly6C^{+} infiltrates that should be reduced with XBP1s restoration.

Minor Concerns:

1. Assessment of extracellular Gln consumption (Extended Fig. 1f)
The glutamine levels measured in the portal vein do not directly reflect cellular consumption. A more appropriate approach would be to quantify tumor Gln uptake in hepatocyte-specific Ern1 KO mice, similar to the analysis in Extended Fig. 1i.

2. Justification for focusing on SLC38A2
The emphasis on SLC38A2 is derived from findings in cDC1s (ref. 32). The authors should consider evaluating other transporters relevant to glutamine uptake, such as SLC1A5 (ASCT2), SLC38A1, and SLC38A5. Inclusion of both mRNA and protein expression levels of these transporters would strengthen the rationale for prioritizing SLC38A2.

3. Mislabeled axes in Extended Fig. 1j
Extended Fig. 1j is mislabeled; the bar graph should indicate ERN1 flox and ERN1 CKO.

4. Verification of immune cell depletion
The authors should provide data confirming the depletion of CD8^{+} T cells, NK cells, and monocytes following administration of anti-CD8, anti-NK1.1, and anti-Ly6C antibodies, respectively.

5. Flow cytometry axis inconsistency (Extended Fig. 4b)
In Extended Fig. 4b, the Day 7 CCR2/Ly6C dot plot appears to use a different X-axis scale compared to the adjacent plot. This should be corrected for consistency.

6. Figure 3e model consistency
Fig. 3e should be replaced with Extended Fig. 4c to maintain consistency of the experimental model across the figure.

7. Inclusion of WT controls in Fig. 4b–c
Fig. 4b and Fig. 4c should include WT controls to demonstrate the decrease in monocyte recruitment and its impact on tumor burden.

8. CCR2 KO and Gln supplementation experiment
For CCR2 KO mice, it would be informative to include outcomes at later time points (e.g., Day 14) to determine whether Gln supplementation eventually promotes tumor growth, given the ability of tumor cells to utilize Gln for proliferation.

9. Missing Y-axis label in Fig. 4b
In Fig. 4b, the Y-axis label is missing for the Ki67 plot. If the axis represents Ly6C, this should be clearly indicated. Additionally, the first bar graph should be labeled as CD11b^{+}Ly6C^{+} on top.

10. Missing sgCtrl control in Extended Fig. 5g
Extended Fig. 5g is incomplete without the sgCtrl control group and should be revised accordingly.

11. Mislabeled cells in Extended Fig. 5g
Extended Fig. 5g incorrectly identifies the cells as CTV-labeled; this should be corrected.

12. Overstated conclusion in Fig. 7a
The statement that "hepatocyte–IM communication did not exist in Ern1fl/fl or Xbp1fl/fl livers" is not fully supported by the data. Ly6C^{+} cells are still present in proximity to hepatocytes, and the images do not provide direct evidence of "communication." This conclusion should be moderated.

Reviewer #3

(Remarks to the Author)
In this manuscript, Zhang and colleagues uncover a metastasis-suppressing role for the IRE1-XBP1 axis in hepatocytes. In absence of XBP1, Foxo1 directs the expression of glutamine synthetase in response to tumor-induced glutamine starvation. In turn, increased glutamine concentration promotes metastasis growth and the accumulation of CCR2+ monocytes. Collectively, the authors have conducted a broad analysis of the effects of glutamine synthesis on hepatic metastasis. The findings are novel, however I find that the conclusions are sometimes overstated and speculative, and the data overly interpreted. Most prominently, I don't think that the experiments presented in the manuscript are enough to dissect the effect

of glutamine synthesis on seeding vs growth of metastases. Moreover, the authors should investigate the mechanisms of monocyte recruitment/accumulation further, as well as dissect how monocytes exert their antitumorigenic functions.
I hope the following comments will help authors improve their manuscript.

Figure 1:
a) Minor points:
- It is mentioned in the text that MKC8866 is and RNAse inhibitor, while in the introduction the authors only mention the enzymatic activity of IRE1. An explication of the dual role of IRE1 as kinase and splicing factor of Xbp1 would help the clarity of the manuscript.
- Small note regarding nomenclature: to simplify I would simply abbreviate Alb-Cre;Ern1flox/flox mice as Ern1KO and Ern1flox/flox as Ern1wt.
e) They authors delete Xbp1, observe a similar phenotype than with Ern1 deletion, and conclude that Xbp1 is the main downstream effector. However, if suggested, epistasis should be formally tested, for example by injecting Xbp1-overexpressing AAV in Ern1Cko mice.
Minor point:
- The authors should mention in the methods which adenovirus capsid they used.

Figure 2:
a) Both tumor area and tumor foci are increased at D7, therefore the authors cannot exclude that there were more lesions at D2, but they were simply too small to be picked up. The authors mention in the text that metastatic foci were markedly larger, but they quantify tumor area. I would suggest that the authors divide the tumor area by number of foci to obtain the average lesion size. In general, authors should stick to the same quantification method for metastases throughout all figures. In figure 1 it is liver weight and tumor area, in figure 2 it is metastasis numbers. The authors should try to quantify tumor foci and obtain average tumor size for their Figure 1 experiments.
Minor points:
o Was quantification performed on two or more non-consecutive sections?
o Was it carried out always on the same lobe?
o For fair comparison, the same lobe should be shown in all figures.
b) Authors mention survival, however Ki67 is a measure of proliferation. It is impossible to distinguish from H&E stainings whether Ki67+ cells are tumor cells or infiltrating immune cells. The authors should include a pancytokeratin staining and only quantify Ki67 positive cells among those. Especially since there is an accumulation of monocytes.
c) The authors state in the introduction that XBP1 is critical for maintaining physiological liver functions. However, they do not assess these liver metabolic functions in Xbp1 or Ern1 KO mice prior to tumor inoculation. Constitutive deletion of these factors might induce imbalances in the liver that make it more susceptible to colonization. The authors should measure metabolic parameters by LC-MS in KO vs WT livers prior to tumor colonization and subtract those baseline differences to those observed in peritumoral areas.
Minor points:
- How were peritumoral areas defined? How were tumors cells excluded?
- In the volcano plot of figure 2C, the authors should label all significantly enriched metabolites/endogenous energy sources.
- The authors conclude that glutamine is actively consumed by the pro-metastatic niche but then go on to show that cancer cells heavily rely on it, and also refer to figure 2b. So, it would be more accurate to say that the tumor actively consumes glutamine. Further experiments are needed to elucidate whether the niche also consumes glutamine, for example measuring glutamine levels after monocyte depletion or in Ccr2 KO mice.
d) Authors delete SLC38A2 form MC38 cells and show that those completely fail to metastasize. This, and the fact that increased tumor foci are observed in Ern1 KO mice actually indicates that seeding is also affected by IRE1 deficiency. I am not entirely sure that Fig 2a alone justifies the conclusion that seeding is not affected. The authors should dissect this point further, if they want to stress it.
Minor points:
- How was the fitness and proliferation of these SLC38A2 KO cells in vitro?
- Extended Data Fig 1j: quantification is either mislabeled or belonging to a different experiment.
e) Metastatic seeding might happen within hours. Authors should supplement glutamine on the same day to make a statement about seeding.

Figure 3:
a) The authors should assess the baseline immune composition in non-tumor bearing Ern1 WT vs KO livers to assess whether altered liver metabolism affects resident immune cell types.
b) Extended data Figure 2b,c,d) authors should show the increase in tumor load in treated vs non-treated mice, not only the difference between Ern1 WT and KO mice.
c) scRNAseq data shows an expansion of monocyte compartment at expense of neutrophil and DC compartment; this would indicate that monocytes recruited to the tumor fail to differentiate or exhibit delayed differentiation. However, this is not evident by flow, where macrophages are also slightly increased. The authors should investigate whether the accumulation of monocytes is due to increased influx or delayed differentiation.
Extended data Figure 3c) did authors subset the two experimental conditions, re-scale and re-normalize and only then calculate monocle trajectories separately? It looks like a unique trajectory onto which cells of either conditions were subjected. This would represent an average trajectory, while two distinct trajectories should be calculated, and then can be aligned within Monocle.
d) The authors should calculate logFC of Ccr2 across clusters of interests. What other genes are differentially expressed in monocytes between experimental conditions? What about cell cycle genes? Can the authors assess proliferation the clusters of interest?

e) The accumulation of monocytes at day 2 suggests, that these cells are important during seeding. Authors should quantify monocytes in Ern1 and Xbp1 KO mice at day 2 as well.
Further points:
- The authors mention a peak in monocytes in the tumor at day 2, however without a day 0 this cannot be called a peak, it might be the baseline.
- The suggested cause is acute inflammation, however the authors should include a sham operated control to support this statement. Was splenectomy performed after intrasplenic injection?
- For consistency, authors should show Ern1 KO mice in the main figure and Xbp1 KO in the supplementary.
f) What about Ly6C negative CCR2 cells? They seem to be expanded in the KO too.
Minor point:
- For clarity, individual channels should be shown in black and white, and the composite using colors.
g) Also here, non-treated groups should be show.
Extended data Fig.4 b and e) what is the Ccr2+Ly6Cintermediate population? It seems to be depleted in MKC treated mice. This would again suggest that monocytes recruited to the tumor fail to differentiate.
Extended data Fig.4 f) The authors should use congenic markers of fluorophores for adoptive transfer, as CTV will be diluted if monocytes proliferate in situ. Adoptive transfer and fate mapping is instrumental to characterize the differentiation trajectories of monocytes in Ern1 KO vs WT mice.
Minor point:
- The authors could also transfer monocytes from tumor bearing mice to assess systemic effects of increase glutamine production (see below).
- At the end of this paragraph, the authors state that monocytes can sustain a cancer-promoting inflammatory state. However, no data are provided to support this claim. It is unclear how monocyte accumulation promotes metastasis growth. Can the authors perform in vitro cell killing assays to show direct cytotoxicity of monocytes towards tumor cells?

Figure 4 and 6:
To improve the flow of the manuscript, which is slightly hard to follow, I would suggest moving figure 6 after figure 4, and moving both 4 and 6 after figure 2. In figure 4, the authors suggest that glutamine consumption by tumor cells reduces local glutamine concentrations and therefore induces glutamine production by Ern1 KO but not Ern1 WT hepatocytes. In figure 6, authors show ectopic GS expression by Ern1 KO hepatocytes.
Figure 6c) how is GS expression regulated in vivo? Is FoxO1 only expressed in zone 3 hepatocytes?

Figure 5:
e) The authors mention anti-inflammatory phenotype but only assess accumulation of CCR2+ cells. Here again, congenic markers should be used, while CTV or EdU can be used to differentiate accumulation vs. in situ proliferation.
Extended Data Fig. 5e) Authors mention that Slc38A is only expressed by monocytes, but did they not knock it out from MC38 cells as well?
Extended Data Fig. 5g) sgCtrl monocytes should also be transferred in both hosts for comparison.
Extended Data Fig. 5h) non-treated should also be shown for comparison.
f) Authors suggest that extracellular glutamine supports expansion of monocytes in the liver. However, they previously show that glutamine levels are elevated in the portal vein of Ern1 KO mice. This suggests that there might be systemic effects. The authors should assess changes in myelopoiesis by looking at the bone marrow of Ern1 KO vs WT mice. The authors should clarify whether accumulation of monocytes in the metastatic liver is due to increased recruitment or in situ proliferation. Of note, the fact that CTV+ monocytes accumulate suggests that these do not proliferate in situ, otherwise CTV would be diluted.

Figure 7:
a) and b) and Extended Fig 7a) The authors talk about hepatocyte IM-communication, however their data show increased IM infiltration in the tumor, which suggests IM-tumor communication. There is no data supporting the notion that hepatocytes and IM directly communicate, only that, in response to tumor-induced glutamine starvation, Ern1 KO hepatocytes produce glutamine that induces accumulation of IM.
Minor points:
- the amount of tumor infiltrating Ly6C monocytes should be quantified.
- For clarity, individual channels should be shown in black and white, and the composite using colors.
Extended Data Fig 7b) the authors state that Xbp1 expression in patients is heterogenous, however from the images show it does not look like it. Is this heterogeneity also observed in WT mice? What is the baseline expression pattern of Xbp1 and IRE1 in non-tumor bearing human/mice liver? Does it correlate with GS expression or liver zonation? How do the authors explain the suggested heterogeneity in expression? Is there a link to ER stress, or to local tumor cell proliferation?
- Can the authors stratify patients in microsatellite stable vs unstable? MC38 cells are a model for microsatellite unstable tumors.
- The authors mention in the discussion that monocytes are known to instruct the pre-metastatic niche, however in this manuscript they only use intrasplenic injection, no primary tumor model. This should be either tested (for example by transferring monocytes from tumor-bearing mice), or stated in the discussion as a limitation of the study.

Reviewer #4

(Remarks to the Author)
The manuscript by Zhang et al. investigates the role of the hepatocyte IRE1α–XBP1s axis in liver metastasis and reveals an

unexpected anti-tumorigenic function. Using genetic hepatocyte-specific knockouts (Ern1CKO and Xbp1CKO) and pharmacologic inhibitors, the authors show that this pathway suppresses metastatic outgrowth by restricting glutamine synthesis and release. Loss of hepatocyte IRE1α–XBP1s activates a non-canonical FoxO1–glutamine synthetase (GS) pathway, leading to increased hepatocyte-derived glutamine, which promotes inflammatory monocyte (IM) survival and proliferation. These IMs, in turn, create a pro-metastatic microenvironment that enhances cancer cell growth. The study presents a novel and conceptually important mechanism suggesting that hepatocyte IRE1α–XBP1s exerts tumor-suppressive effects by limiting glutamine-driven inflammatory monocyte expansion and pro-metastatic niche formation. However, several key mechanisms remain unresolved, including:
- How inflammatory monocytes (typically anti-tumor) promote metastasis in this context, and whether macrophage differentiation and reprogramming is involved.
- Direct mechanistic proof of the FoxO1–GS axis.
- Clarification if monocyte recruitment rather than proliferation is necessary.
These issues, along with missing methodological and quantification details, justify a recommendation of major revision.

Major Comments
1. Monocytes versus macrophages: missing mechanistic explanation
The manuscript attributes tumor promotion to increased inflammatory monocytes, but it remains unclear how pro-inflammatory monocytes promote tumor growth, and how this is distinguished from monocyte-derived (M2) macrophages, which are classical mediators of metastatic niche formation.
- The anti-Ly6C antibody (Extended Fig. 2c) and CCR2 inhibitor (Fig. 3g) experiments prevent monocytes and consecutevly prevent differentiation of macrophages. Therefore, a destinction can not be made basted on those experiments.
- The authors should clarify the mechanistic link between glutamine, IM accumulation, and tumor promotion. If the monocytes are indeed pro-inflammatory, what specific cytokines, factors, or interactions make them pro-tumorigenic?
- The single-cell RNA-seq data could be leveraged to examine macrophage polarization (e.g. CD206, Mrc1, Arg1, CD80, CD86, MHCII, CD163).
- Additional flow cytometry using these markers should be included to verify macrophage abundance and polarization (M1 vs M2).
- Given that glutamine metabolism is known to drive M2-like macrophage polarization, macrophage reprogramming may also contribute to the observed pro-metastatic phenotype and should be thorougly investigated.

2. Monocyte recruitment versus local proliferation
The differentiation between recruitment and local proliferation of IMs in IRE1α/XBP1-hepatocyte deficient livers remains ambiguous. Fig. 4c shows that glutamine effects are lost in CCR2-/- mice, suggesting recruitment is required.
- Fig. 3g demonstrates that CCR2 inhibition (RS102895) abolishes the metastatic increase in Ern1CKO mice, further implying dependency on recruitment.
- The adoptive transfer experiment (Extended Fig. 4f) is showing elevated monocyte numbers, but this could equally reflect increased recruitment rather than proliferation. If most cells within the livers were really derived from proliferation, this could be shown by the decrease of CTV in those newly devided cells.
- Real proliferation should also be shown by CTV assay not indirectly by Ki67 in all in-vitro experiments.
- Investigate whether IRE1α/XBP1-deficient hepatocytes secrete higher levels of CCL2 or other chemokines responsible for increased monocyte recruitment.
- Extended Fig. 4e: Since MKC8866 treatment increases IMs, also test MKC8866 in CCR2-/- mice to proof resistance to MKC8866 in the liver is really monocyte-depndent (effect should be restored in this setup, as the direct tumor effect remains).

Given loss of effect in CCR-/- mice and with CCR2 inhibition, together with the common concept that mature monocytes outside the bonemarrow rarely proliferate, a proliferation-based hypothesis needs more substantial proof.

3. Mechanistic link between IRE1α–XBP1s and FoxO1–GS signaling
The connection between IRE1α–XBP1s loss and FoxO1–GS activation is not directly shown.
- Fig. 6c demonstrates that FoxO1 inhibition reduces GS and glutamine in vitro, but it does not establish that FoxO1 mediates GS induction in Ern1CKO hepatocytes.
- → Show that FoxO1 inhibition restores GS expression and glutamine secretion in Ern1CKO hepatocytes to WT levels.
- → In vivo, validate that FoxO1 intensity correlates in histology with GS expression in peritumoral hepatocytes (Fig. 6a).

4. Validation of the tumor-suppressive hepatocyte model
To support the claim that hepatocyte IRE1α–XBP1s signaling is tumor-suppressive and causes resistance to MKC8866 in the liver, the authors should directly test MKC8866 treatment in Ern1CKO and Xbp1CKO mice.
- Include four experimental groups per model (WT ± inhibitor, CKO ± inhibitor).
- If IRE1α–XBP1s protection is lost in knockout hepatocytes, MKC8866 should once again reduce tumor growth by acting directly on tumor cells.
- This is essential to confirm the interpretation of Fig. 1c–f and to support the claim that the MKC8866 inefficacy in liver metastases is due to loss of hepatocyte protection.

5. Consistency between models
The manuscript alternates between Ern1CKO and Xbp1CKO models.
→ Use one consistently in main figures and include confirmatory results from the other model in the Supplementary Figures. This would improve narrative flow and clarity.

Minor Comments
6. Metabolomics (Fig. 2c) – report all metabolites and rationale
- The metabolomics analysis focuses solely on glutamine, but the Supplementary Table with complete data was not provided (to the reviewer).
- Justify the specific focus on glutamine over other altered metabolites.
7. Experimental controls and missing methodological details
- Fig. 4f: Include WT ± glutamine control groups to confirm that glutamine supplementation was effective in this setup.
- Hepatocyte-conditioned medium: Culture conditions, baseline glutamine levels, and glutamine concentrations during co-culture with BMIMs are insufficiently described.
- → Include media controls with equivalent glutamine concentrations (compareble to the difference in hepatocyte-conditioned medium) ± L-asparaginase to confirm that observed effects are glutamine-specific.

8. Fig. 3f: Quantify $CCR2^+$ IMs both within tumor lesions and in surrounding tissue to confirm local enrichment.
9. Blood and spleen: Include IM frequencies to determine whether changes are local or systemic.
10. Fig. 6c: Provide full Western blot images, importantly quantifications, and potentially reduced exposure times to enhance band distinction.
11. Fig. 4f: Monocytes are expressed as % of $CD45^+$ cells—could this reflect a differentiation defect rather than proliferation? Include cell counts, show macrophages, increase sample size for statistical robustness (there is a clear trend in the L-Asp treated groups as well).
12. Extended Fig. 4f: Confirm whether data are expressed as % of $CD45^+$ or $CD11b^+$ cells, as $CD11b^+$ would better match the representative plots in Extended Fig. 4b.
13. Clinical trial rationale:
- The rationale that IRE1α inhibitors (e.g., ORIN1001 / MKC8866) are ineffective in metastasis is insufficiently supported.
- NCT03950570: published only as a safety abstract with limited efficacy data.
- NCT04834778: investigates HC-5404, a PERK inhibitor, not an IRE1α inhibitor.
- Please provide accurate references or clarify that no clinical data on anti-metastatic efficacy are yet available.

14. Timing of glutamine supplementation (Fig. 2e–f)
- If glutamine is crucial for IMs, and IM frequency peaks at day 2 (Extended Fig. 4b), why is supplementation at day 1 ineffective?
- Harmonize time points between Fig. 2e and Fig. 2f (e.g., day 5 and 7, as used in Fig. 2a). Currently, the timely distance between early Gln and sacrefice is 11 days and for late Gln only 2 days.
- → Discuss whether monocyte glutamine dependence changes dynamically during metastatic niche formation.

15. Surgery-induced hepatic stress (Fig. 3)
- Since intrasplenic injection and surgery can independently cause ER stress, control sham-groups are needed.
- → Include sham-operated WT and CKO mice, measure ALT, AST, serum glutamine, and assess monocyte flow and counts at day 2 post-surgery to exclude stress-related artifacts.
16. Extended Fig. 1j: The manuscript refers to Ern1CKO mice, but only Ctrl and Gln groups are shown—correct this labeling.

17. Fig. 5c: Provide a supplementary table with transcriptomic data. The upregulated macrophage genes (Enpp1, Treml2, Scd1/2) indicate a lipid metabolism–biased, M2-like macrophage profile, consistent with published data linking glutamine to M2 polarization.
18. Extended Fig 5.: The authors should perform co-transfer experiments (e.g., CD45.1.2 WT and CD45.1 sgSlc38a2 monocytes 1:1 ratio in the same host, ideally Ern1CKO). Then, compare counts, % and proliferation within this host to disentangle cell-intrinsic versus environmental effects.

Reviewer #5

(Remarks to the Author)
The study investigates how hepatocyte-intrinsic IRE1α- XBP1 signaling shapes the liver metastatic microenvironment. Contrary to the commonly tumor-promoting roles of IRE1a and XBP1, the authors propose that hepatocyte IRE1a XBP1 activity suppresses liver metastasis by limiting local metainflammation. They report that hepatocyte-specific deletion of IRE1a or XBP1 increases inflammatory monocyte accumulation and metastatic burden through a proposed non-canonical FoxO1 glutamine synthetase pathway that enhances glutamine secretion. Restoration of XBP1 or inhibition of glutamine metabolism is suggested to reduce monocyte levels and limit metastasis. Limited patient data are presented to support a positive correlation between hepatocyte XBP1 expression and survival. The manuscript therefore aims to redefine hepatocyte unfolded protein response signaling as a regulator of monocyte-supported metastatic growth.

While the manuscript uses an impressive number of mouse models, a central concern is the lack of essential control experiments validating the mouse models. The Ern and Xbp1 hepatocyte specific knockout models are not adequately confirmed. It remains unclear how hepatocyte specific deletion was verified, whether IRE1 signaling is demonstrably reduced, and whether baseline liver phenotypes differ in a way that could influence metastatic susceptibility. These questions directly affect interpretation of downstream findings.

Other major points

• L133: The XBP1 overexpression model seems to lack validation. The manuscript does not seem to convincingly show that functional XBP1 is increased. Since both knockout and overexpression models underpin major mechanistic claims, they require much stronger characterization.
• Control experiments for SLC38A2 depletion are missing.
• Extended Data Fig 2b to f: Control tumor areas vary from ten to fifty percent despite samples being collected the same day. Intra experimental variation is smaller than inter experimental variation. Additional antibody treatment controls would improve interpretability.
• l370 and l374: Claims regarding hepatocytes with abundant GS expression and invasive margins heavily infiltrated by Ly6C positive monocytes rely on a single small image (roughly two hundred fifty micrometers squared) without quantification. Assertions about XBP1 low GS positive areas are not supported by shown images. GS localization differs between ADV Ctrl and ADV XBP1s treated mice, and monocyte distribution appears similar across most images except for the XBP1 fl/fl example. Proper quantification across multiple samples is required.
• CCR2 antagonist and related treatments: Figures lack side by side comparisons between treated and control animals. For instance, treated mice show a mean tumor area of around seven point five percent versus roughly forty percent in untreated mice, yet the direct comparison is not displayed (Fig 3g).
• Figure 6a: The upper panel does not convincingly show tumor cells. Morphology differs from verified tumor cells in earlier figures. In the lower panel, GS positive cells are perivascular, and the inset suggests a chance alignment with regions designated as tumor. These images do not convincingly support the stated differences in GS positivity.
• Figure 7: The Kaplan Meier plots lack essential information. It is not clear how many patients were censored or the size of each group. Censoring indicators for the red curves are nearly invisible.
• Extended Data Fig 7b: Tissue labeled as tumor is in fact a fibroinflammatory rim or stromal capsule, not tumor. Interpretation of related immunofluorescence panels is difficult because HE images are missing.
Other points:
• l87: Hepatocytes may not be functionally intact in premetastatic niches. They can already be altered, as shown for example by Bojmar in Nature Medicine twenty twenty four.
• l119: Liver weights are used as surrogate markers for tumor burden, which is not established. Tumor size should be quantified directly.
• The protein folding roles of XBP1 and IRE1 should be mentioned for context.
• XBP1s should be referred to as XBP1.
• l190: The term metastatic outbreak is unclear and should be defined.
• l237: The phrase disease associated expansion is misleading. The data only show the presence of cells, not their expansion. The figure also does not compare tumor versus normal liver but rather shows very limited regions containing a few double positive cells.
• l75 to l76: The phrase in the presence of hepatocyte IRE1a may be imprecise. It might be more accurate to refer to conditions where IRE1a is activated.
• The manuscript does not clarify which liver regions were used for single cell RNA sequencing or flow cytometry. It is unclear whether whole liver tissue including tumor and surrounding parenchyma was analyzed.
• Fig 3f immunofluorescence: The precise location of the imaged region is unclear, likely intratumoral but not confirmed.
• HE quantification based size appears in Fig 1b and Extended Data Fig 1b, not in Fig 1a and Extended Data Fig 1a as stated in l123 to l124.
• In vitro SLC38A2 transporter depletion is shown in Extended Data Fig 1h, i, j, not 1h and l as written in l181.
• Extended Data Fig 1j is labeled Gln on the x axis, but appears instead to compare Ern1 fl fl in blue versus Ern1 CKO in red.
• l310 and following: The figure does not show a reduction in Ki67 positive CCR2 positive cells relative to BMM sgCtrl injection. Instead, it compares Ern1 fl fl to Ern1 CKO. The text therefore does not accurately describe the displayed data. Similarly, in Fig 5f with DRP 104 treatment, the authors claim a significant reduction in endogenous monocytes, but the comparison shown does not support these conclusions.
• l226 and following: Cluster one is described as expressing lineage markers and pro inflammatory signatures. However, complement components such as C1qa, C1qb, and C1qc, as well as Mif and Ccl2, are commonly expressed by macrophages and do not necessarily reflect a pro inflammatory or cancer promoting phenotype. The interpretation should be refined.
• The manuscript proposes that IRE1a and XBP1 deficient hepatocytes foster monocyte accumulation, but the causal evidence remains incomplete.
• Fig 5d: The y axis range obscures transcripts above the significance threshold. Genes such as Siglec1 appear likely to exceed the cutoff but cannot be clearly seen. Plot scaling should be adjusted.
• Fig 6a immunohistochemistry quantification: The marker index is not defined, and measurement locations are unclear.
• Fig 6 western blots: Quantitative summaries of reproducible experiments seem to be missing. Reported changes, such as GS increases with decreasing glutamine or higher FoxO1 in Ern1 CKO, are not evident in the images. Replicate numbers must be stated.
• Fig 6f: Non treated control data should be shown alongside treated data.
• Fig 7b: Control mice treated with ADV Ctrl do not express XBP1s near metastases, which seems inconsistent with Fig 1h. This discrepancy needs to be addressed.
• In situ data generally show small tissue regions with limited quantification. Monocyte phenotyping is restricted to Ly6C or CD14, although CD14 is also expressed by macrophage subsets. More detailed phenotyping is needed.
• The term metainflammation is used without sufficient introduction. It is primarily associated with metabolic disease and needs clearer explanation when invoked in the context of hepatocyte derived signals near metastases.

Reviewer #6

(Remarks to the Author)
I co-reviewed this manuscript with one of the reviewers who provided the listed reports. This is part of the Nature Communications initiative to facilitate training in peer review and to provide appropriate recognition for Early Career Researchers who co-review manuscripts.

Version 1:

Reviewer comments:

Reviewer #1

(Remarks to the Author)
The authors have experimentally addressed all the questions and concerns that were raised previously. I have no further questions with the revised manuscript. Thanks for their effort for improving the work before publication.

Reviewer #2

(Remarks to the Author)
Authors have addressed most of the major raised comments. However, I still have reservations about their metastasis model, which is does not endogenously occur. Also, I don't understand why they have removed all the axis labels in the revised figures to address the formatting issues pointed out in the original manuscript. That said, the additional experiments have significantly strengthened the study, and I think the manuscript has now reached an acceptable standard for publication.

Reviewer #3

(Remarks to the Author)
The authors have addressed my concerns.

Reviewer #4

(Remarks to the Author)
The authors address my concerns and this current format present compelling results for supporting their exciting findings.

Reviewer #5

(Remarks to the Author)
The authors have responded to all concerns raised during the previous review round. Any points from the first revision not revisited below are considered satisfactorily addressed.

In response to the point that the authors use liver weight as a surrogate for tumor burden, they state that they quantify tumor burden directly, which is acknowledged. However, the text continues to focus on liver weight when comparing experimental groups, e.g., in the first section of the results (L121 and following). As mentioned earlier, liver weight is not an established readout of tumor burden, and its baseline values vary markedly across experimental groups. This is especially evident in the control group shown in Fig. 1g when compared to the control groups shown in Fig. 1b–f, among others. We suggest amending the wording and focusing on tumor size throughout instead.

Regarding validation of the hepatocyte-specific knockout mouse models, the authors state that the models have been characterized previously. They also provide one single Western blot showing that hepatocytes isolated from Ern1CKO mice no longer respond to tunicamycin treatment (at one selected concentration) with upregulation of IRE1α and XBP1 protein levels, while showing increased FoxO1 protein levels compared to hepatocytes isolated from Ernfl/fl mice. This result validates the loss of stress response pathway components in the main knockout model used. In addition, the authors provide Ki67 and TUNEL immunolabeling results as indicators of hepatocyte proliferation and apoptosis in tumor-distant livers, as well as broad immune cell composition data from the tumor microenvironment. Both datasets compare Ernfl/fl and Ern1CKO mice in the metastatic context.

However, whether baseline liver phenotypes that may affect metastatic seeding, outgrowth, or progression are present in the models used in vivo remains incompletely resolved. The manuscript would benefit from additional characterization of the naïve (non-tumor-bearing) livers of Ern1fl/fl versus Ern1CKO mice, for example, liver architecture/histology, and markers of hepatocyte stress or the unfolded protein response, to clearly establish whether loss of Ern1 alters the hepatic microenvironment independent of metastatic colonization.

We argued previously that the in situ evidence provided does not sufficiently support the cell sorting–based findings on monocyte infiltration, and that the abundant presence of these cells in the tumor microenvironment upon hepatocyte modification was not adequately demonstrated. In particular, the methodological description of these results was insufficient. The revised manuscript still does not provide sufficient detail on how multiplex immunofluorescence labeling results were quantified. In particular, the terms MFI and HPF are not explained in sufficient detail, limiting reproducibility. As it stands, the

manuscript does not describe the size of the analyzed tissue regions or the methods used to quantify labeling intensity.

The authors commented that zone 3 GS expression was excluded from quantification; however, Extended Data Fig. 7b appears to show that liver zonation is lost in peritumoral liver. Thus, exclusion of GS-positive areas based on GS labeling intensity is no longer a valid approach in the tumor microenvironment. The authors are encouraged to provide a more detailed justification for this exclusion than is currently presented.

We suggested providing evidence for tumor cell presence and fractions within the images shown in Fig. 6a, and noted that the areas shown were not representative of the quantification data presented in the adjacent graph. In response, the authors replaced the representative image in the Ern1fl/fl panel. To interpret the new data, the manuscript would benefit from tumor cell–specific immunolabeling on a consecutive tissue section to unambiguously confirm tumor cell identity in the images shown. In addition, the method used to quantify the GS labeling signal in the accompanying graph is not described in sufficient detail. In particular, it remains unclear how the MI is defined (see comment above), and which image areas and of what size were included in the quantification.

We suggested clarifying in the Methods section which liver regions were used for single-cell RNA sequencing and flow cytometry. The authors answered this question in the rebuttal but did not add this information to the current version of the manuscript.

We commented that in Fig. 7b, XBP1s expression is not visible in the representative multiplex immunofluorescence images chosen from ADV-Ctrl samples, whereas it is visible by immunohistochemistry in Fig. 1h. The authors responded that this reflects methodological differences between multiplex immunofluorescence and immunohistochemistry, with the latter being more sensitive to low signal intensities. However, low signal intensities can also be corrected for in immunofluorescence-based techniques. As such, it remains a concern how consistent and spatially homogeneous XBP1 protein levels actually are.

We commented that the in situ data generally show small tissue regions with limited quantification, and in the same comment addressed the choice of Ly6C and CD14 as the sole monocyte phenotype markers. The authors responded by citing literature establishing CCR2 as a well-established inflammatory monocyte marker, and referred to Fig. 3, where co-immunolabeling for CCR2 and Ly6C is shown. However, this does not address the general concern raised in our comment. Fig. 3f, in particular, exemplifies the generally very small regions shown for in situ labeling throughout the manuscript; these small representative regions do not capture intra- and intertumoral heterogeneity. This comment was of a general nature and applies not only to Fig. 3f but also to Fig. 6d, 7a, Extended Data Fig. 2a–c, and Extended Data Fig. 8a, g, and h. Beyond showing greater heterogeneity in the in situ data, selecting larger or multiple representative regions per figure requires adding a full description of the methods used to summarize the data to the manuscript. We suggest including annotation procedures, how mean intensity was summarized per sample/region, and a transparent account of imaging field size and cell numbers included in the analysis. In this context, the Methods section also lacks a methodological definition of positive cell populations, including thresholding as shown in Fig. 7c, Fig. 3f, and Extended Data Fig. 8c–f.

We commented that the term "metainflammation" is used without sufficient introduction. The authors replied that they replaced the term with "local metainflammation." However, this still does not sufficiently introduce the term. It remains unclear whether the data on monocyte infiltration, without accompanying cytokine profiling or functional assessment of the cells support the use of the term in the way it is done in the current version of the manuscript.

Reviewer #6

(Remarks to the Author)
I co-reviewed this manuscript with one of the reviewers who provided the listed reports. This is part of the Nature Communications initiative to facilitate training in peer review and to provide appropriate recognition for Early Career Researchers who co-review manuscripts.

Version 2:

Reviewer comments:

Reviewer #5

(Remarks to the Author)
We appreciate the authors’ clarification of several aspects of the methodology. However, important details regarding the annotation strategy and region selection remain insufficiently described.

We believe that we can assess tumor morphology irrespective of tumor size. Nevertheless, in the representative images provided, it is not always possible to unambiguously distinguish tumor cells from other cell populations, such as inflammatory infiltrates. Cytokeratins, although acknowledged by the authors as not entirely specific, are routinely used in human pathology to distinguish epithelial tumor cells from epithelial non-tumor cells. Moreover, cytokeratin immunolabeling profiles continue to play an important role in the classification and diagnosis of tumor subtypes.

Therefore, some of our concerns remain unresolved, and we leave it to the Editors to determine whether the current level of methodological clarification is sufficient.

Reviewer #6

(Remarks to the Author)
I co-reviewed this manuscript with one of the reviewers who provided the listed reports. This is part of the Nature Communications initiative to facilitate training in peer review and to provide appropriate recognition for Early Career Researchers who co-review manuscripts.

Open Access This Peer Review File is licensed under a Creative Commons Attribution 4.0 International License, which permits use, sharing, adaptation, distribution and reproduction in any medium or format, as long as you give appropriate credit to the original author(s) and the source, provide a link to the Creative Commons license, and indicate if changes were made.
In cases where reviewers are anonymous, credit should be given to 'Anonymous Referee' and the source.
The images or other third party material in this Peer Review File are included in the article's Creative Commons license, unless indicated otherwise in a credit line to the material. If material is not included in the article's Creative Commons license and your intended use is not permitted by statutory regulation or exceeds the permitted use, you will need to obtain permission directly from the copyright holder.
To view a copy of this license, visit https://creativecommons.org/licenses/by/4.0/

Reviewer #1 (Remarks to the Author):

This manuscript reports the mechanistic investigation of hepatocyte IRE1α-XBP1s axis in suppressing local secondary inflammation, thereby inhibiting hepatic metastasis of tumor cells. Through a combination of genetically engineered mouse models and biochemistry approaches, the authors provided evidence showing that XBP1s suppresses glutamine synthesis in hepatocytes via negative regulation of the FoXO1-GS pathway, which limits monocyte expansion and subsequent tumor cell colonization in the liver. The findings reveal a crucial mechanism by which the IRE1α–XBP1s axis modulates hepatocyte–monocyte interactions, suggesting a potential therapeutic strategy against hepatic metastasis.

Overall, this study was well executed, and the presented data clearly provided an interesting mechanism by which the IRE1α-XBP1s pathway regulates hepatocyte-monocyte crosstalk in suppressing hepatic metastasis.

Response: We thank the reviewer for the appreciation of our work and the suggestions for improving it.

Some minor points can be considered for further enhancing the conclusions as follows:

1. How the IRE1α-XBP1 pathway in hepatocytes changes at different time points (D2, D4, D7) after isp remains unclear, which should be analyzed in parallel with the FoXO1-GS pathway.

Response: As indicated by our immunohistochemical analyses across different time points in the MC38 liver metastasis model using wild-type mice, p-IRE1α and XBP1s protein levels were undetectable in healthy wild-type livers but became modestly elevated in peritumor hepatocytes during metastatic progression. A similar temporal pattern was observed for FoxO1 and non-zone 3 GS expression, both of which modestly increased as metastases developed, particularly around day 7.

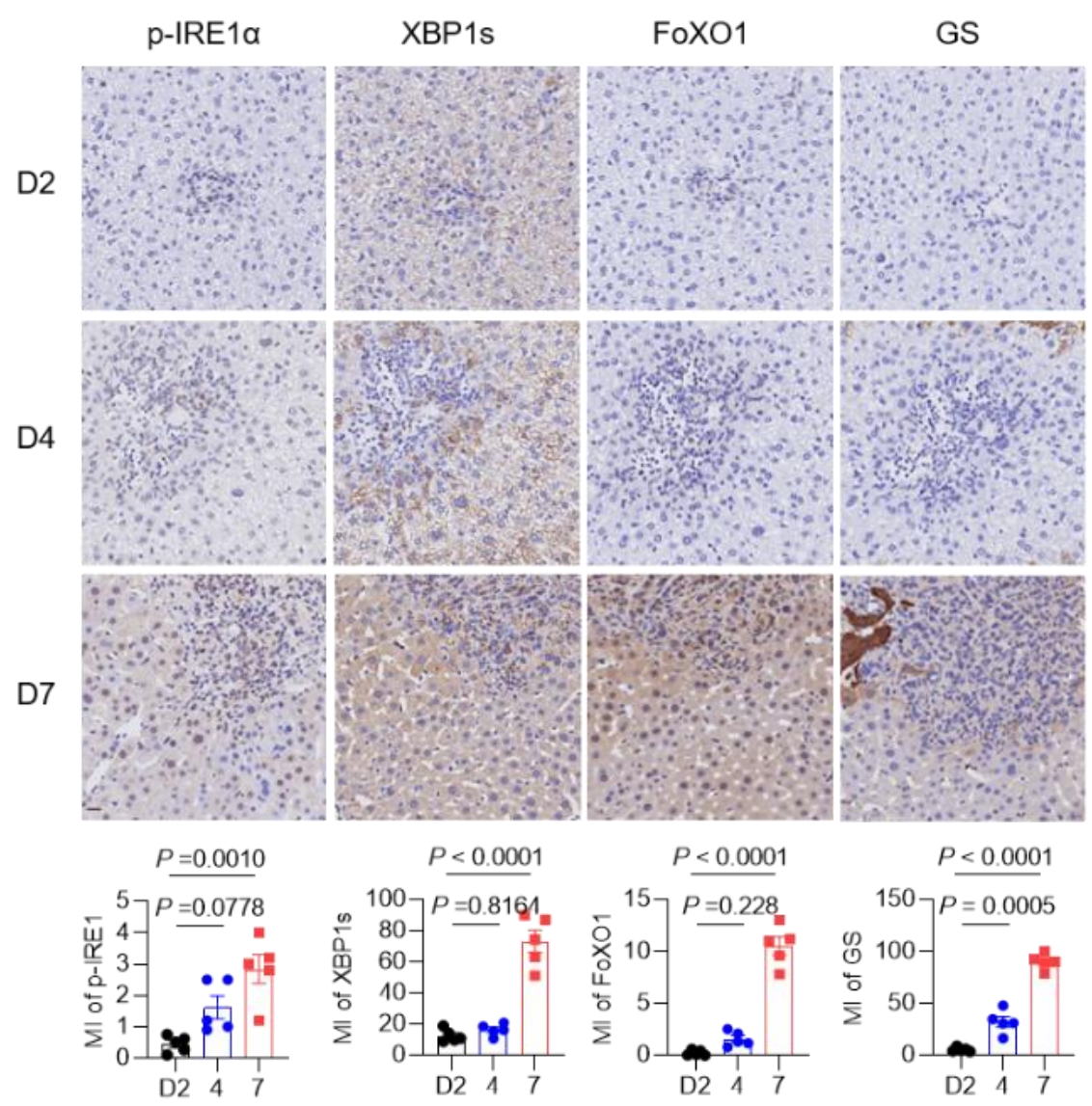

2. The upregulation of FoXO1 in in vitro-cultured Ern1CKO mouse hepatocytes does not appear to be significant in Figure 6c. Quantitative analysis is required to support this conclusion. Protein expression levels of IRE1α and XBP1s should also be shown. Additionally, it is necessary to assess FoXO1 and GS expression in XBP1CKO mouse hepatocytes.

Response: We repeated the western blot analyses of *Ern1*$^{fl/fl}$ and *Ern1*^{CKO} hepatocytes (**Fig. 6c**) and confirmed that both FoxO1 and GS protein levels were increased following IRE1α depletion and were further elevated under glutamine-restricted conditions. Deficiency of the IRE1α–XBP1 pathway was validated in a parallel experiment: expression of IRE1α and XBP1s was robustly induced only in tunicamycin (Tm)-treated *Ern1*$^{fl/fl}$ hepatocytes, whereas little to no expression was detected in untreated *Ern1*$^{fl/fl}$ hepatocytes or in *Ern1*^{CKO} hepatocytes under either condition (**Extended Data Fig. 7e**).

We additionally evaluated FoxO1 and GS protein levels in *Xbp1*$^{fl/fl}$ and *Xbp1*^{CKO} hepatocytes and confirmed the differential expression of both proteins between the two genotypes (**Extended Data Fig. 7d**). Although glutamine restriction further increased FoxO1 expression in *Xbp1*^{CKO} hepatocytes, GS expression was not further upregulated. This may be because GS expression is already substantially derepressed following XBP1 depletion. Alternatively, XBP1 deficiency is known to trigger compensatory activation of IRE1α (PMID: 18556558), which may exert additional XBP1-independent effects on GS regulation. However, any such contribution is likely modest and is beyond the scope of the current work.

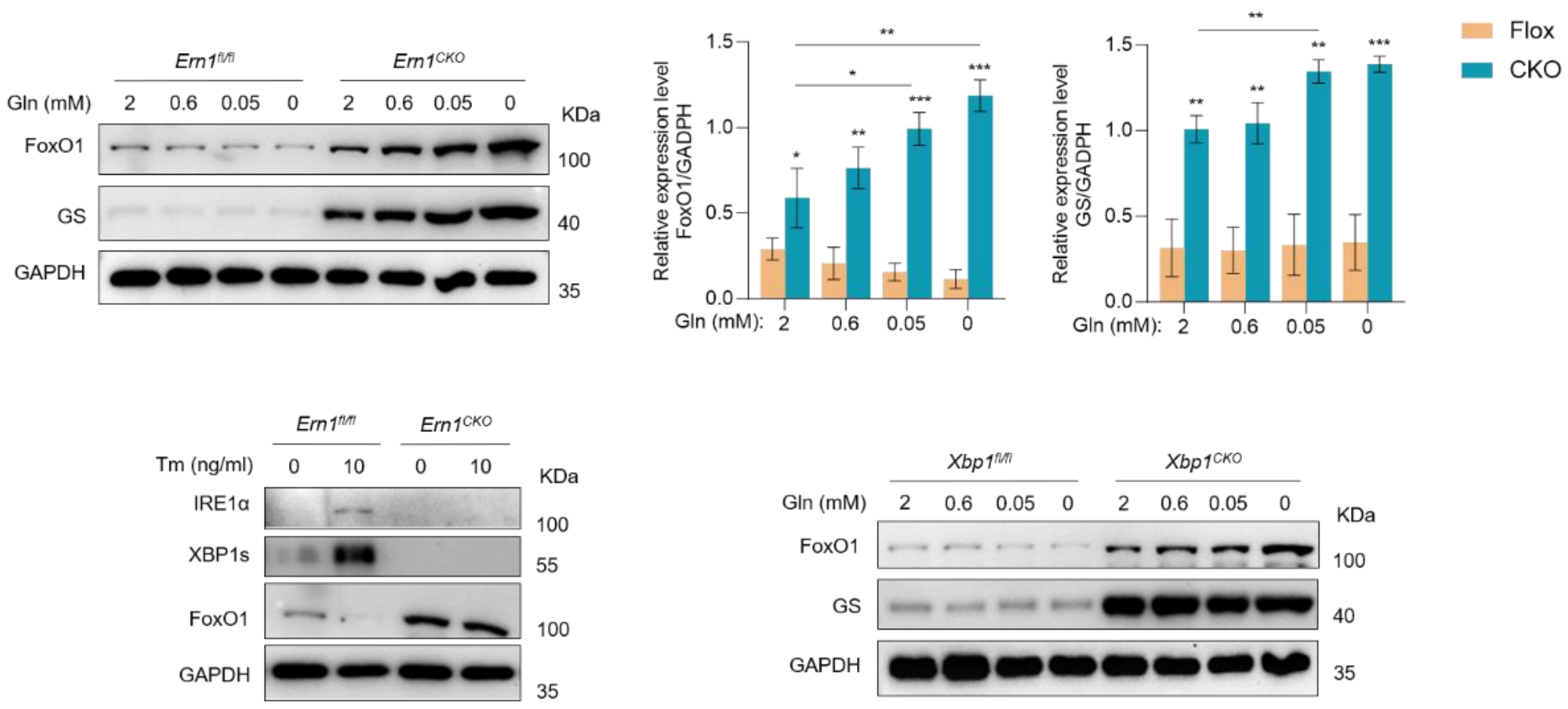

3. In Extended Data Figure 4e, MKC8866 treatment led to the accumulation of CCR2+ inflammatory monocytes in the wild-type metastasis-bearing livers. And yet it did not appear to affect MC38 liver metastasis as shown in Figure 1b. This discrepancy should be clarified.

Response: We believe that the intrahepatic accumulation of $CCR2^+$ IMs resulted from MKC8866 treatment is an offset to its anticancer effect. This explains why MKC8866 destroyed MC38 subcutaneous tumors (**Fig. 1a**) but failed to reduce MC38 liver metastases (**Fig. 1b**).

4. The H&E staining in Figure 1c requires a representative flox control tissue section showing more comprehensive liver analysis.

Response: We appreciate this advice and have replaced it with a more qualified image from the same group (**Fig. 1c**).

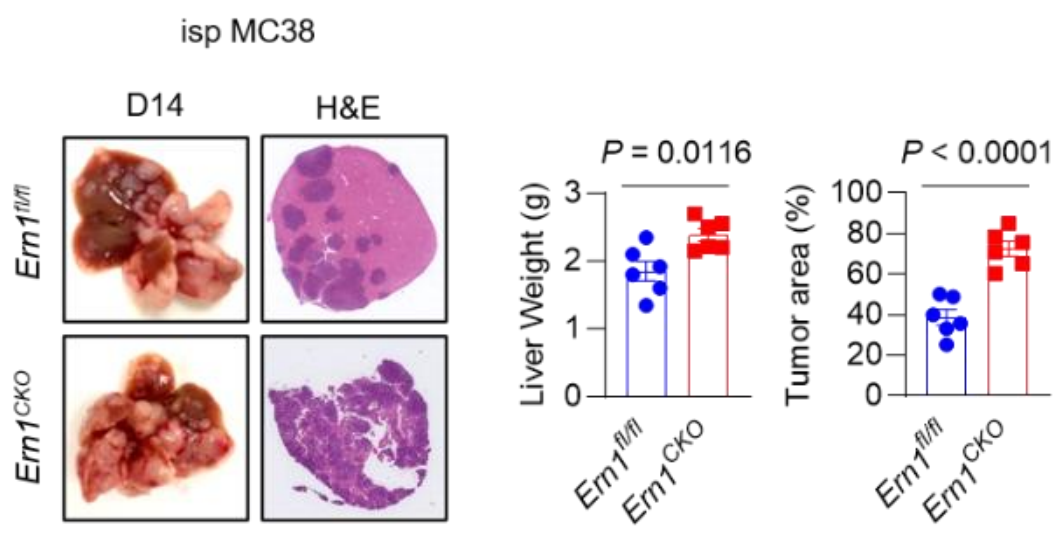

5. AV- or AAV-mediated restoration of XBP1s expression in IRE1α-CKO livers can be employed to confirm the results in vivo, including the effects on the FoXO1-GS pathway along with the hepatic metastasis.

Response: We appreciate this advice and have introduced a XBP1s-expressing adenoviral vector (or a control vector) into *Ern1*^{CKO} livers 7 days before intrasplenic tumor inoculation. As expected, restoring XBP1s in the IRE1α-deficient context was sufficient to mitigate metastatic progression as well as to reduce FoxO1/GS expression and monocyte infiltration (**Extended Data Fig. 1e, 7c** and **Fig. 7b**).

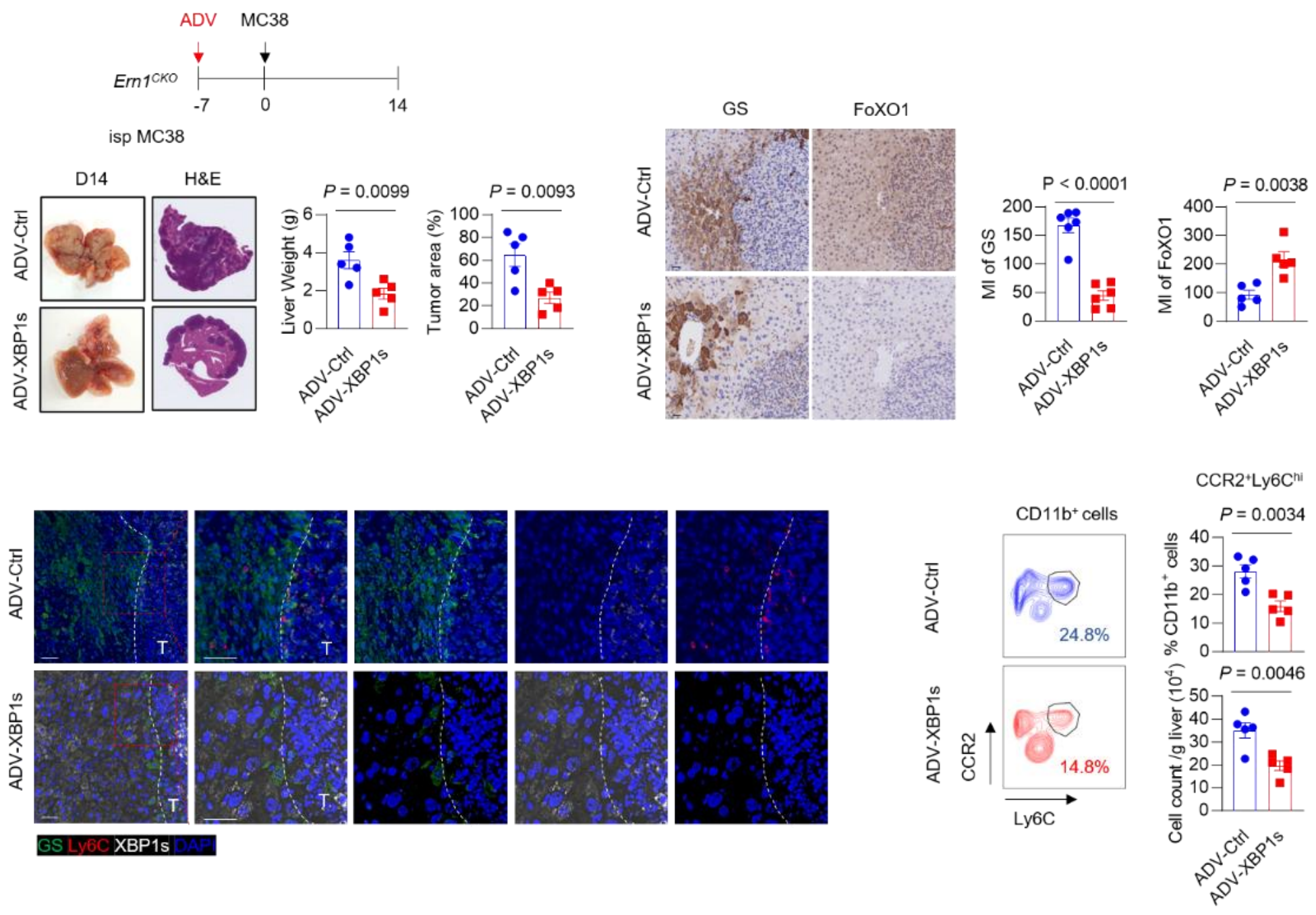

6. What may underlie the regulation by hepatocyte IRE1α of the FoXO1-GS pathway in the context of hepatic metastasis instead of HCC development? May some factors from metastatic tumor cells be involved in specifically activating the IRE1α branch or inducing ER stress to activate all the UPR branches?

Response: It is well established that hepatocyte IRE1α drives ER stress-associated HCC, a setting characterized by hyperactivation of the IRE1α–XBP1 pathway and stress-related events (PMID: 29506314; 38418544). By contrast, our data indicate that, under conditions of modest IRE1α activity and environmental glutamine restriction, the downstream XBP1s–FoxO1–GS axis functions to restrain local pro-metastatic inflammation. These two outputs appear to be mutually exclusive, as FoxO1 expression was suppressed under ER stress conditions (**Extended Data Fig. 7e**). Therefore, healthy hepatocytes in the pre- or early-metastatic liver, which are not subjected to substantial ER stress, may utilize the IRE1α–XBP1s pathway as a protective mechanism (discussed in **Lines 482–488**).

Reviewer #2 (Remarks to the Author):

The manuscript entitled "Hepatocyte IRE1α–XBP1s axis prevents pro-metastatic niche formation through metabolic rewiring of inflammatory monocytes" by Zhang, Lian, Chen, Yang, Fu, et al, aims to determine how hepatocyte-specific IRE1α–XBP1s axis affects liver tumor progression. Results show that inhibition of IRE1α impairs the growth of subcutaneous tumors but conversely increase their

progression in models of metastatic hepatic burdens. Conditional IRE1α–XBP1s deletion in hepatocytes inhibits liver metastasis by promoting the expansion of CCR2+ inflammatory monocytes (IMs). Conversely, hepatocytes specific XBP1s-overexpression before tumor inoculation reduced metastatic lesions. Notably, glutamine availability was increased in the peritumor areas of hepatocyte IRE1α–XBP1s deficient mice compared to controls, which promoted tumor metastasis growth via glutamine transporter SLC38A2. Mechanistically, IRE1α- or XBP1-null hepatocytes increase the expression of FoxO1-regulated glutamine synthetase (GS), which primes the secretion of glutamine to support CCR2+ IM fitness. As such, rescue expression of hepatocyte XBP1s or blockade of glutamine metabolism effectively reduces IMs and mitigates metastasis progression. Validation of the mouse data in liver cancer patient settings include results showing that liver metastases positively correlates with higher XBP1s expression in hepatocyte. Together, results suggest that Hepatocyte IRE1α–XBP1s limits metastasis by impairing glutamine availability for IMs. Results are timely and highlights an unexpected role of IRE1α–XBP1s in liver tumors and in the regulation of monocyte function. However, there were multiple limitations that prevented the full support to the report. Comments are as following:

Response: We thank the reviewer for the appreciation of our findings and the constructive critique.

Major Comments:

1) Role of Hepatocyte IRE1α–XBP1s in metastasis of spontaneous tumor models

Report shows an antitumor effect of IRE1α RNase inhibitor MKC8866 (ORIN1001) in subcutaneous LLC and MC-38 models, but an opposite effect after intra-splenic injection of the tumors (liver tumor growth). To provide clarity, it would be interesting to test the effect of this inhibitor in spontaneous tumor models that metastasize to the liver.

Response: We appreciate the reviewer’s concern and therefore evaluated several previously reported models of spontaneous liver metastasis, including subcutaneous injection of MC38 or EL4 cells and intramammary injection of 4T1 cells followed by primary tumor resection. Among these models, liver metastases were consistently observed only in the 4T1 setting. Notably, in this model, treatment with MKC8866 reduced lung metastases but did not exhibit any therapeutic benefit in the liver (**Extended Data Fig. 1c, d**).

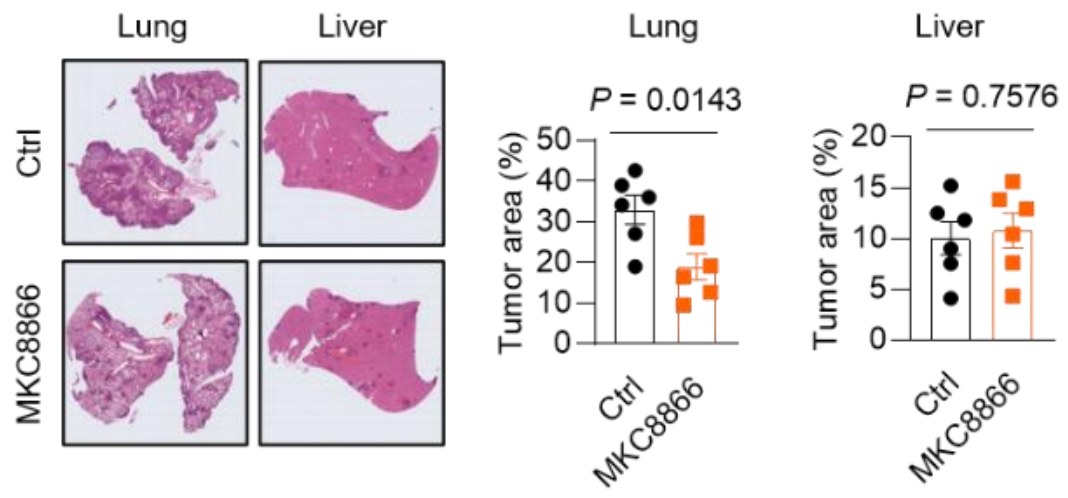

2) Metastatic burden models

The models used for metastatic burdens are artificial by nature. Validation of the studies in models showing spontaneous metastasis in the liver would be primary.

Response: Spontaneous liver metastases following intramammary injection of 4T1 cells corroborated our findings from the intrasplenic injection model (**Extended Data Fig. 1c, d**), supporting a liver-specific resistance to IRE1α-targeted therapy. However, intrasplenic injection-based liver metastasis proceeds through the physiologically relevant portal vein route and has been extensively validated in landmark studies for investigating the immunobiology of liver metastases derived from gastrointestinal cancers and melanoma (PMID: 29443964, 41203813, 23081697, 30842658, 41690948, 39048831), including the seminal work that first established the fundamental role of $CCR2^+$ IMs. Although this approach does not recapitulate the initial intravasation stage, it provides a unique and well-controlled experimental window to interrogate intercellular communication within the liver—a process that is most relevant to our study—while minimizing systemic confounding variables commonly encountered in spontaneous metastasis models.

3) Glutamine as a mechanistic mediator

It would be important to determine whether catabolism of glutamine via glutaminase could overcome the pro-metastatic and high frequency of iMs phenotype observed in hepatocyte-specific IRE1α- or XBP1-null mice bearing tumors. This could complement the results showing that glutamine antagonism abolished the metastasis supporting effect of IRE1α or XBP1 depletion.

Response: We appreciate the reviewer's suggestion. However, although this approach is intended to mitigate extracellular glutamine release, enhanced glutaminolysis via glutaminase would inevitably generate additional glutamate and ammonia (and subsequently α-ketoglutarate), thereby confounding the interpretation of hepatocyte–immune communications.

4) Glutamine role outside of metastatic tumors

Glutamine uptake inhibition alters the growth of tumor cells in the liver, but could also impact growth outside of the liver. Thus, it would be important to evaluate the importance of glutamine uptake by

cancer cells in primary tumor growth. Therefore, is the effect of SLC38A2 only observed in metastasis settings, or also in the tumors injected sc?

Response: We performed a subcutaneous tumor model using MC38-sgSlc38a2 versus MC38-sgCtrl cells and demonstrated that glutamine uptake is essential for primary tumor growth, consistent with a recent report (PMID: 37407815). Although the dependence of cancer cells on glutamine uptake remains largely unchanged across different tumor sites, the liver metastatic niche uniquely involves additional consumers of extracellular glutamine, notably inflammatory monocytes. Glutamine concentration within this niche directly contributes to the differential metastatic burden observed between *Ern1*$^{fl/fl}$ and *Ern1*^{CKO} livers.

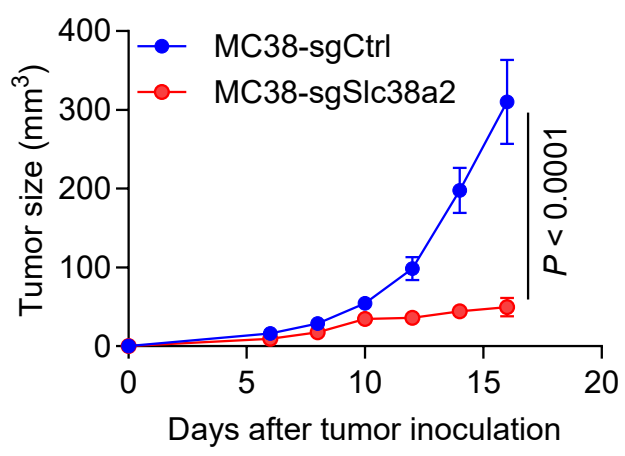

5) How does CCR2+ iMs promote metastasis

The mechanisms whereby CCR2+ IMs support metastasis expansion remain unclear. Further characterization of these processes and identification of major functional differences would be highly impactful. Also, validation of the inflammatory and pro-tumor nature of the iMs would be needed.

Response: Given that CCR2^{+} IMs is well established as pro-metastatic, and that our study primarily focuses on the upstream regulatory mechanism governing their accumulation, we initially managed to summarize how they functionally promote metastasis, supported by key references (**Line 431–440**).

In investigating how hepatic IRE1 depletion accelerates metastasis, we have excluded altered cancer cell seeding/extravasation and local/systemic immunosuppression as contributing factors (**Fig.2** and **Extended Data Fig.1–3**). Therefore, in the revision, we directly examined whether CCR2^{+} IMs promote metastatic progression by enhancing cancer cell growth. As anticipated, liver-derived CCR2^{+} IMs enhanced MC38 proliferation in an ex vivo co-culture assay and promoted metastatic progression in an adoptive transfer model (**Extended Data Fig. 5c, d**).

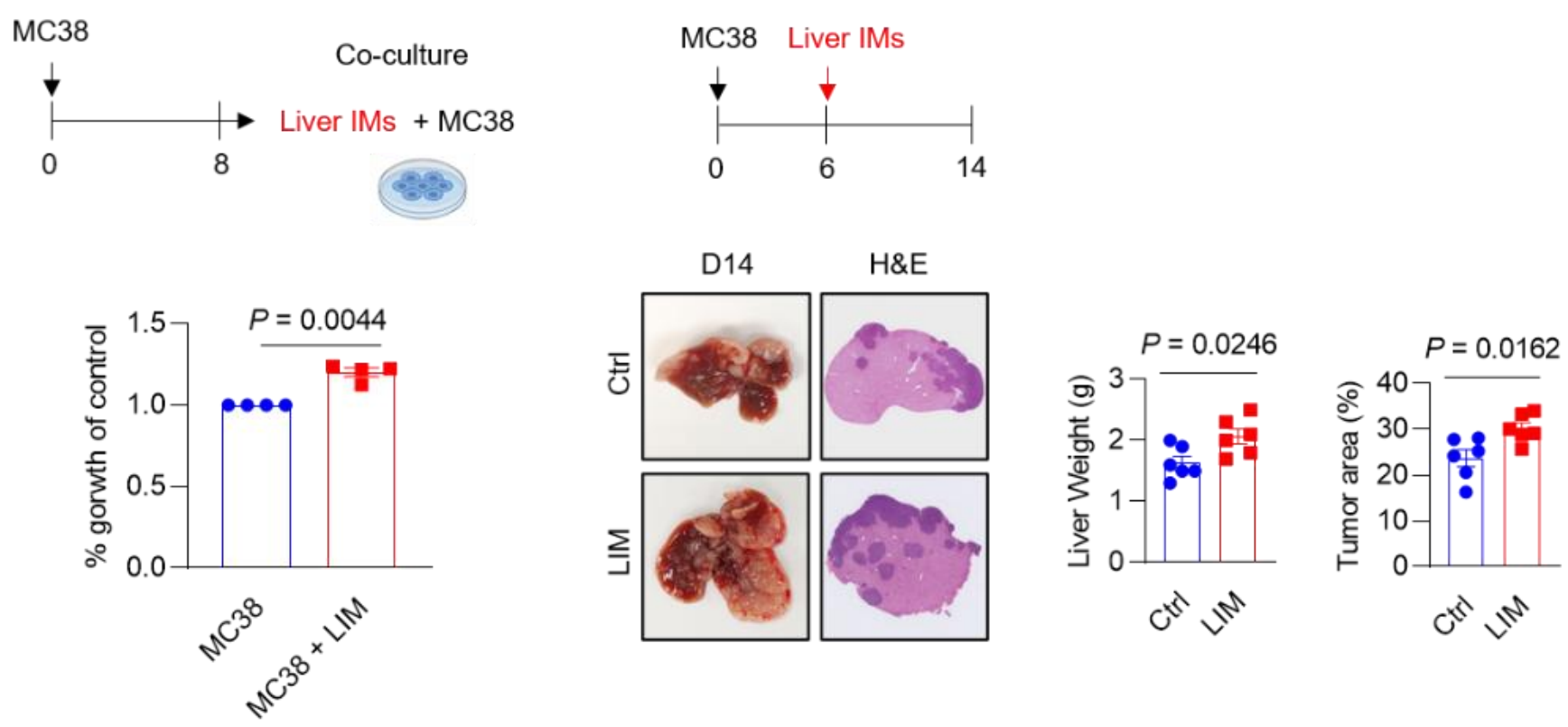

6) How does glutamine support the activity of iMs?

Response: Comprehensive in vivo and in vitro experiments demonstrated that glutamine directly supports the expansion of IMs (**Fig. 4a, 5a,** and **5b**). Transcriptomic analyses further confirmed that glutamine sustains the pro-inflammatory program of IMs (**Fig. 5c**). Consistently, their expansion was markedly attenuated upon ablation of the glutamine transporter SLC38A2 (**Fig. 5e**).

7) Rationale and interpretation of Fig. 1e–f

The rationale for including Fig. 1e and 1f remains unclear. Specifically, the early administration of glutamine (Gln) failed to induce tumor progression without explaining the possible cause. Additionally, the decision to assess endpoints two days after Gln administration (Fig. 1f) rather than on Day 14 post-tumor implantation, as done in earlier experiments, requires further justification.

Response: Because metastatic cells require nutrients to withstand metabolic stress during the early seeding phase, we initially administered glutamine at an early timepoint (day 1) based on this rationale. The absence of an effect indicates that extra glutamine does not functionally promote metastatic seeding, potentially due to the glutamine-saturated microenvironment at this early stage. In contrast, when glutamine became relatively limiting at an intermediate stage (day 6), exogenous glutamine supplementation was able to facilitate liver metastatic progression (see also in **Line 199–204**). Moreover, we have included a later endpoint (day 14) to further support the conclusion (**Fig. 2f**).

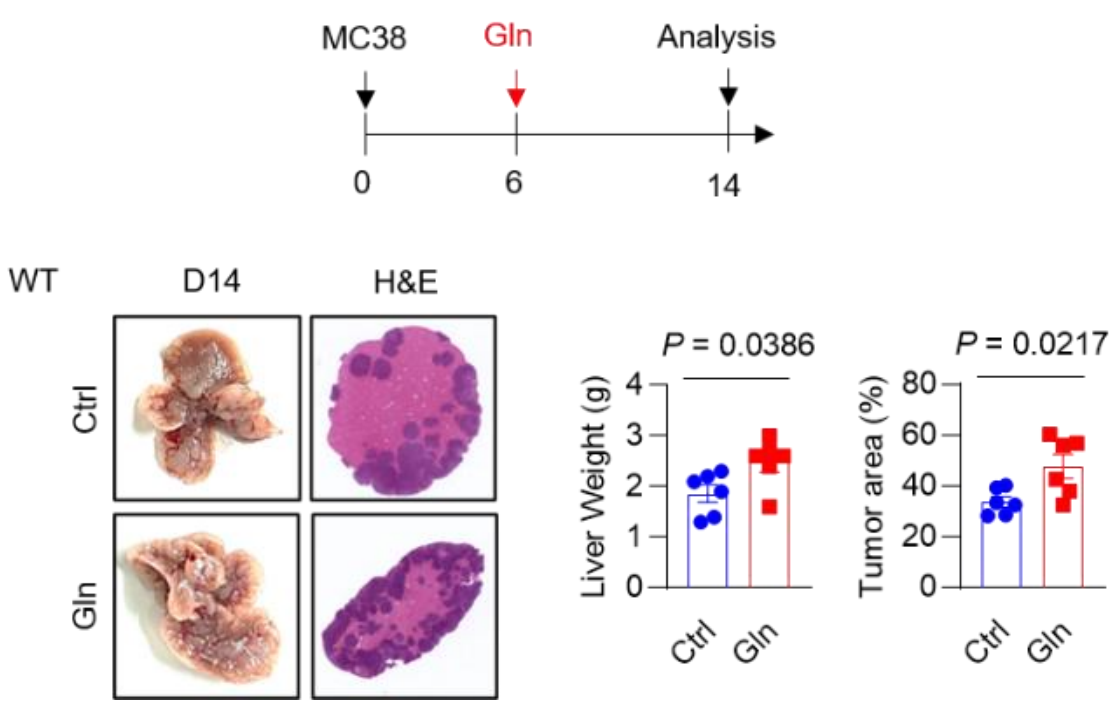

8) Lack of tumor burden comparison between sgCtrl and sgSLC38A2

The authors did not provide a direct comparison of tumor burden between sgCtrl and sgSLC38A2 groups. Therefore, the claim that “SLC38A2 deficiency profoundly inhibited metastasis formation” is not adequately supported by the presented data.

Response: We have used MC38-sgSlc38a2 versus MC38-sgCtrl cells to confirm that glutamine uptake is essential for liver metastasis formation (**Extended Data Fig. 2h**).

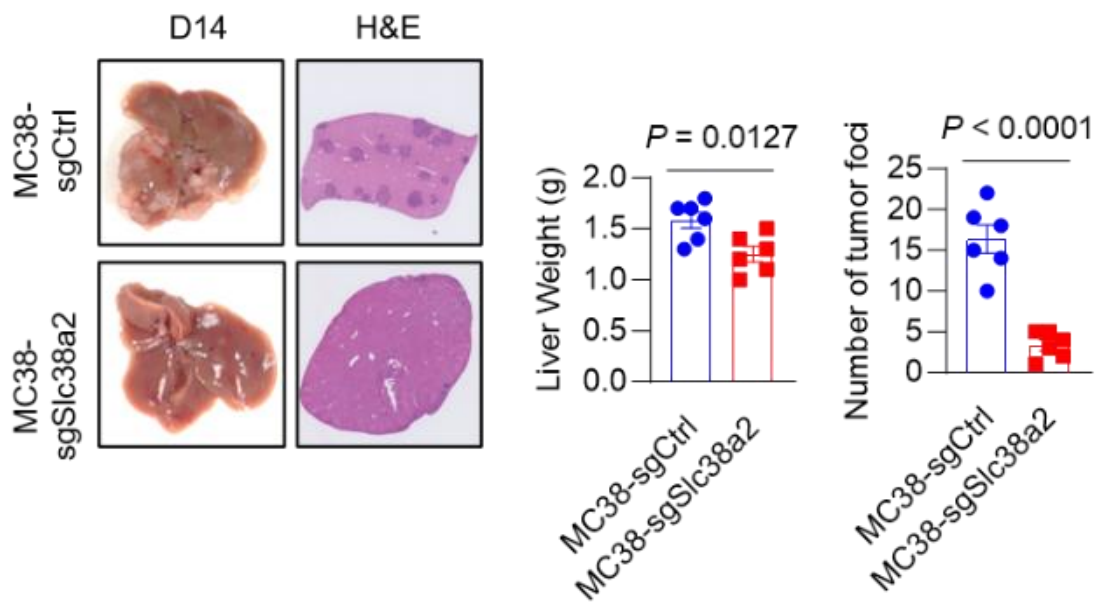

9) Interpretation of monocyte-depletion experiments (Fig. 2)

If tumor-intrinsic glutamine uptake drives tumor proliferation, as proposed, it is unclear why monocyte depletion using anti-Ly6C or pharmacologic CCR2 inhibition (RS102895) completely abolishes the ERN1 CKO associated increase in tumor burden, rather than producing only a partial effect. This questions whether the previous proposed mechanisms are relevant drivers of the found phenotype.

Response: Tumor-intrinsic glutamine uptake is essential for liver metastasis. However, it is the excessive glutamine enrichment in the *Ern1*^{CKO} liver—rather than insufficient glutamine in the *Ern1*$^{fl/fl}$ liver—that drives the differential metastatic burden. This additional effect of environmental glutamine is mediated by monocyte-driven inflammation rather than direct stimulation of cancer cell proliferation. Accordingly, depletion of monocytes is sufficient to abolish the metastatic difference between *Ern1*$^{fl/fl}$ and *Ern1*^{CKO} livers.

10) MKC8866 combination treatments

Does combining MKC8866 with DRP-104 or RS102895 re-sensitizes tumor cells to MKC8866, which would strengthen the mechanistic interpretation.

Response: We appreciate the reviewer’s suggestion and have confirmed that the combinatorial use of RS102895 can resensitize liver metastases to MKC8866 treatment (**Fig. 3h**).

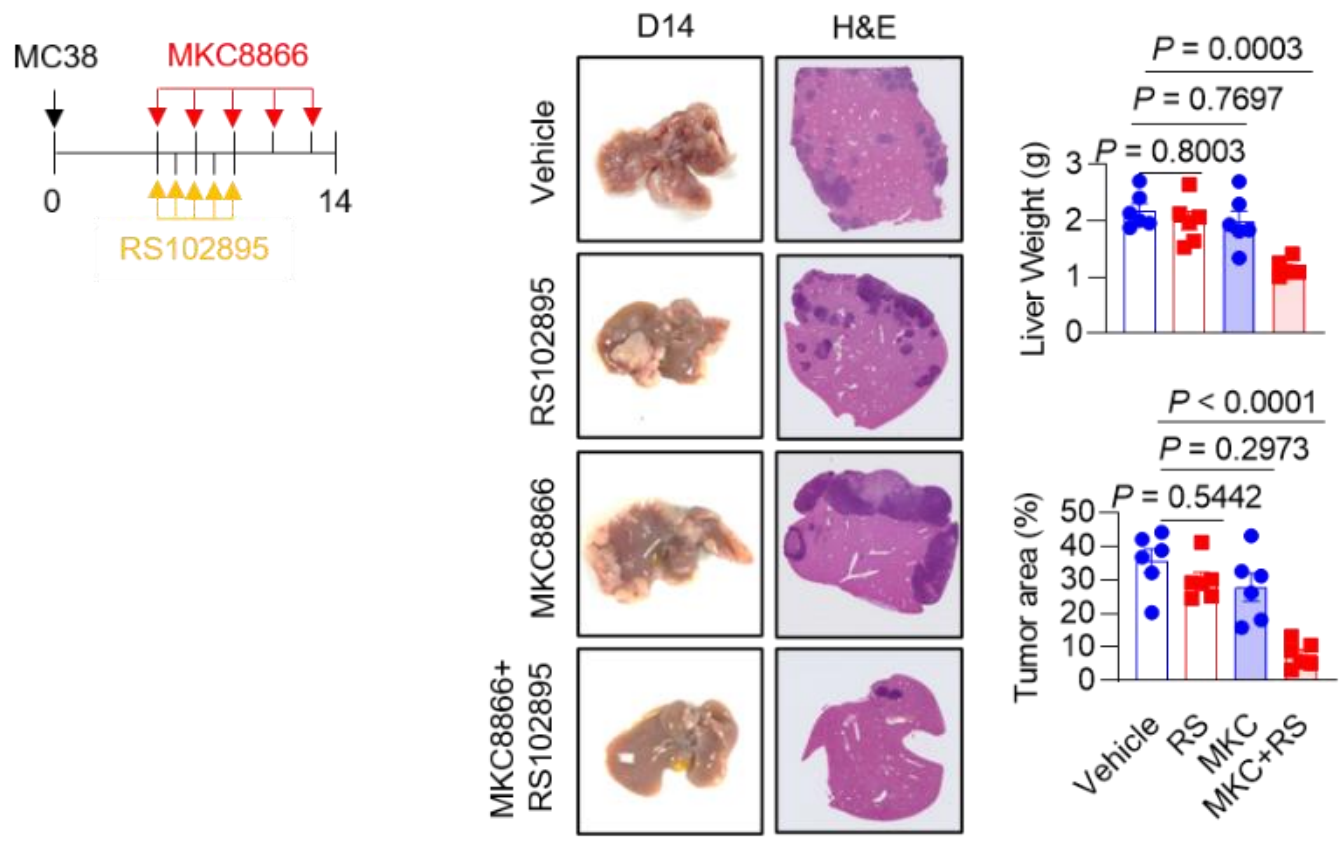

11) Flow cytometry issues in Extended Fig. 4f

In Extended Fig. 4f, the CTV-labeled cells are not clearly distinguishable from host cells. The authors should optimize CTV labeling intensity or use congenic markers (e.g., CD45.1/CD45.2) and GFP expressing cells that used in later part of the story to ensure reliable discrimination. Additionally, it would be important to determine whether ERN1 CKO mice receiving labeled Ly6C⁺ cells similarly show an increased Ki67⁺CCR2⁺ population found in XBP1 CKO mice.

Response: As suggested by the reviewers, we transferred congenic BMIMs to tumor-bearing *Ern1*$^{fl/fl}$ and *Ern1*^{CKO} mice. The latter enabled better expansion of transferred cells (in a manner dependent on the glutamine transporter SLC38A2) (**Fig. 5e**), phenocopying that of **Extended Data Fig. 5i** (previous Extended Data Fig. 4f).

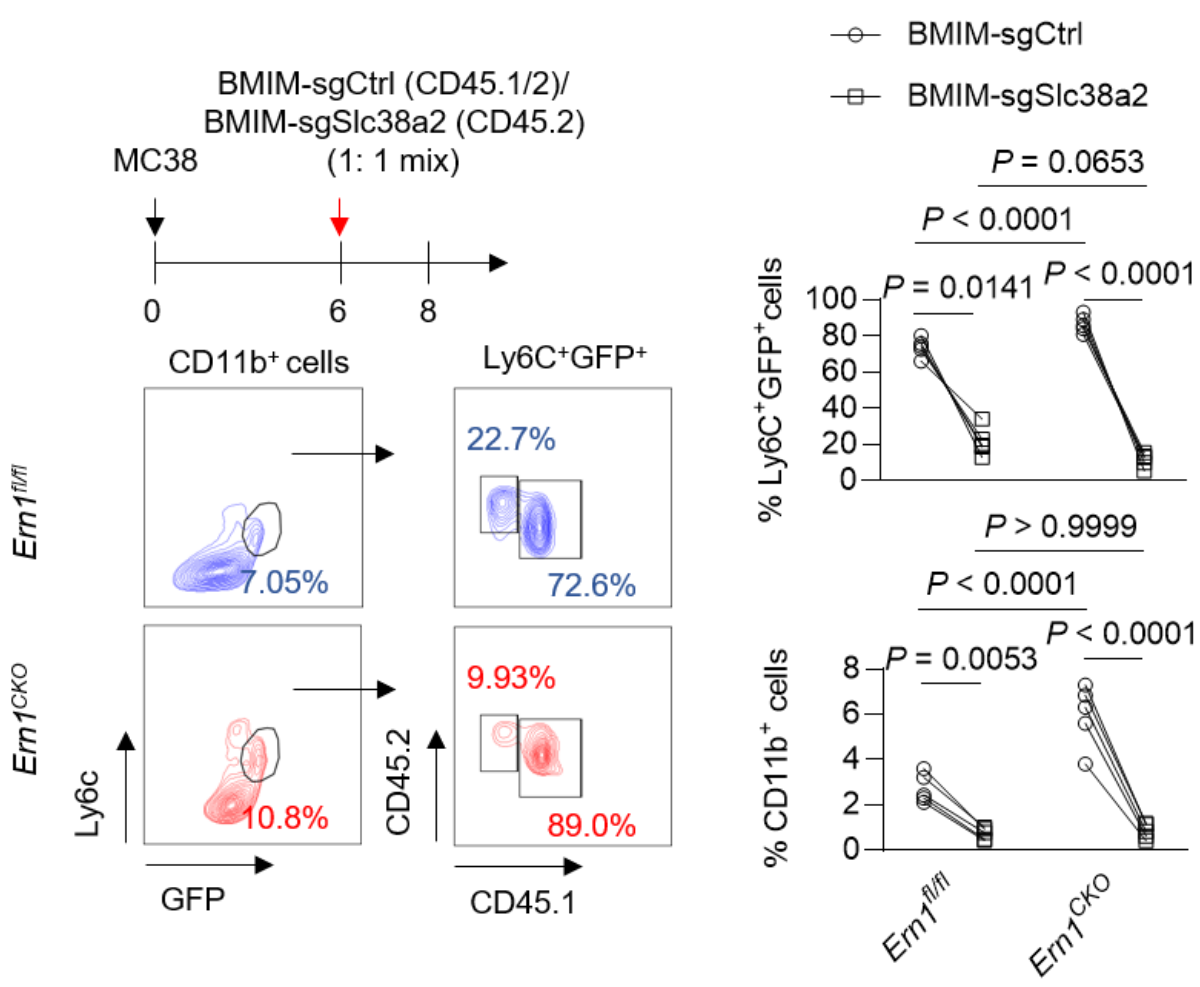

12) Overinterpretation of inflammatory phenotype (Fig. 4f–g)

The authors measure Ki67 as a marker of monocyte proliferation but do not assess additional inflammatory markers. Therefore, the statement that "extracellular glutamine… is an upstream driver of monocyte transitioning into an inflammatory state" should be moderated to reflect the limited scope of

the data.

Response: CCR2 is a well-established marker of inflammatory monocytes (IMs) (PMID: 21654748; 23081697) and has therefore been used consistently throughout our mouse models. In addition, our ex vivo single-cell RNA-seq and in vitro bulk RNA-seq analyses confirmed that IMs exhibit a glutamine-associated pro-inflammatory transcriptional program (**Extended Data Fig. 4d** and **Fig. 5c**). Nevertheless, we appreciate the reviewer's comment and have revised the manuscript (**Line 311**) to replace the term "an upstream driver" with the more appropriate and precise phrasing, "a key driver."

13) GS protein analysis (Fig. 6c)

Fig. 6c should include either a shorter WB exposure for GS or densitometric quantification to support the conclusion that GS expression increases under low extracellular Gln.

Response: We have repeated the experiment using a shorter Western blot exposure and have replaced the GS and FoxO1 bands accordingly in **Fig. 6c**. We have also provided densitometric quantification accordingly.

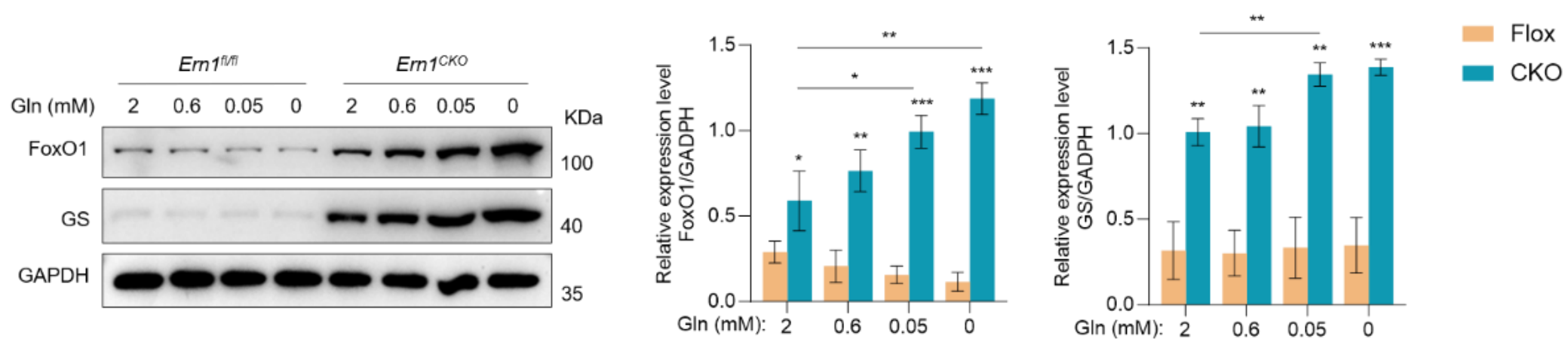

14) Inclusion of XBP1s KO in Fig. 6c

Including XBP1s KO in Fig. 6c would strengthen the conclusion that XBP1s-deficient conditions lead to elevated FOXO1 compared to WT controls.

Response: We have included XBP1-deficient hepatocytes for validation and observed co-upregulation of FoXO1 and GS, which recapitulated the IRE1α-deficient phenotype (**Extended Data Fig. 7d**). Glutamine restriction further increased FoxO1 expression in *Xbp1^{CKO}* hepatocytes but did not significantly affect GS expression. This may be because GS is already substantially derepressed following XBP1 depletion. Alternatively, compensatory activation of IRE1α (PMID: 18556558) may exert additional XBP1-independent effects on GS regulation, which is beyond the scope of the current study.

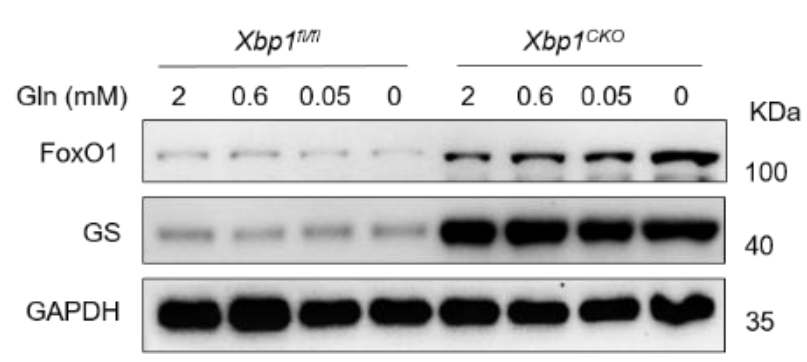

15) XBP1s overexpression rescue experiment

The authors should evaluate whether XBP1s overexpression (via ADV-XBP1s) in ERN1 KO hepatocytes reverses monocyte accumulation and tumor progression. This model may also be more suitable for immunofluorescence imaging, as ERN1 KO mice exhibit elevated Ly6C$^+$ infiltrates that should be reduced with XBP1s restoration.

Response: We appreciate this advice and have introduced a XBP1s-expressing adenoviral vector (or a control vector) into *Ern1*^{CKO} livers 7 days before intrasplenic tumor inoculation. As expected, restoring XBP1s in the IRE1α-deficient context was sufficient to mitigate metastatic progression as well as to reduce FoxO1/GS expression and monocyte infiltration (**Extended Data Fig. 1e, 7c** and **Fig. 7b**).

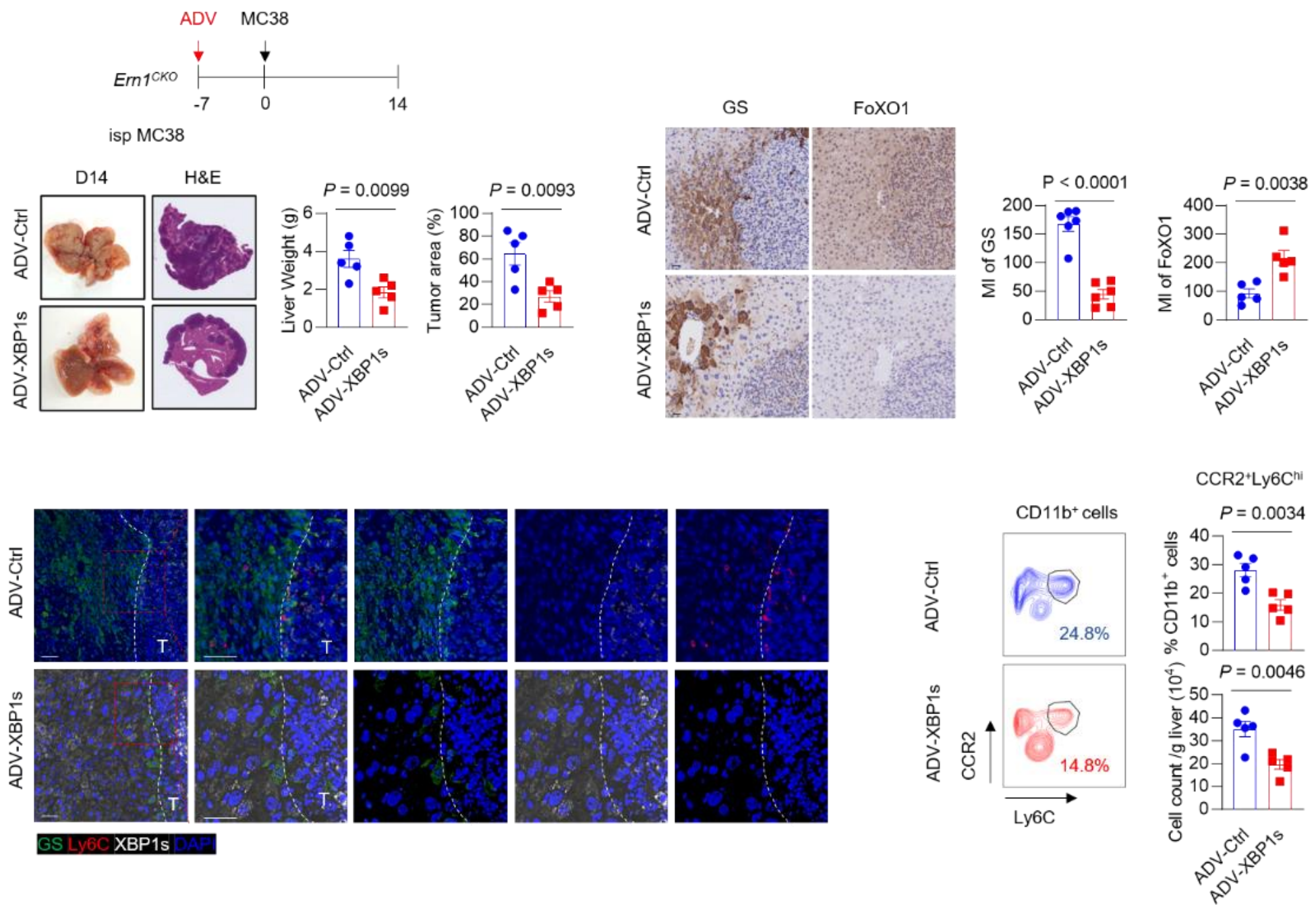

Minor Concerns:

1. Assessment of extracellular Gln consumption (Extended Fig. 1f)

The glutamine levels measured in the portal vein do not directly reflect cellular consumption. A more appropriate approach would be to quantify tumor Gln uptake in hepatocyte-specific Ern1 KO mice, similar to the analysis in Extended Fig. 1i.

Response: We observed that liver metastases-bearing *Ern1*^{CKO} mice showed comparable glutamine concentrations in tumor lysates but relatively higher glutamine concentrations in adjacent liver lysates compared to *Ern1*$^{fl/fl}$ mice. This phenotype is consistent with our overall model, in which extra glutamine is released by hepatocytes and preferentially consumed within the pro-metastatic niche.

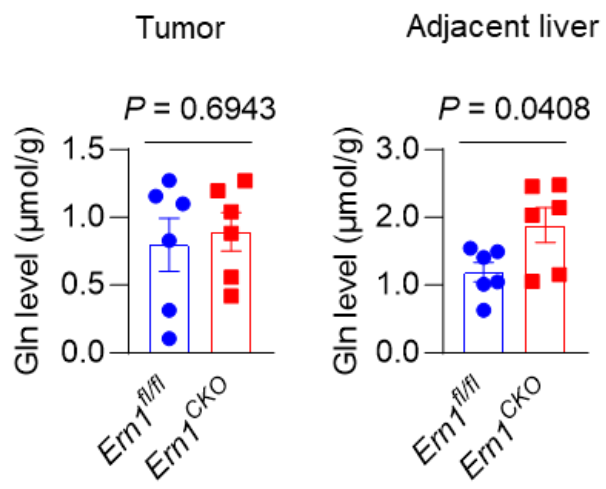

2. Justification for focusing on SLC38A2

The emphasis on SLC38A2 is derived from findings in cDC1s (ref. 32). The authors should consider evaluating other transporters relevant to glutamine uptake, such as SLC1A5 (ASCT2), SLC38A1, and SLC38A5. Inclusion of both mRNA and protein expression levels of these transporters would strengthen the rationale for prioritizing SLC38A2.

Response: We analyzed our single-cell RNA-seq dataset to evaluate the expression of known glutamine transporters. Among these transporters, SLC38A2 showed the highest frequency of expression within the monocyte cluster, despite exhibiting lower absolute transcript levels than those detected in dendritic cells. To gain an understanding at the protein level, we performed flow cytometric analyses of the major glutamine transporters. Notably, only SLC38A2, but not the other transporters, was significantly upregulated in intrahepatic monocytes from *Ern1*^{CKO} mice compared with those from *Ern1*$^{fl/fl}$ mice.

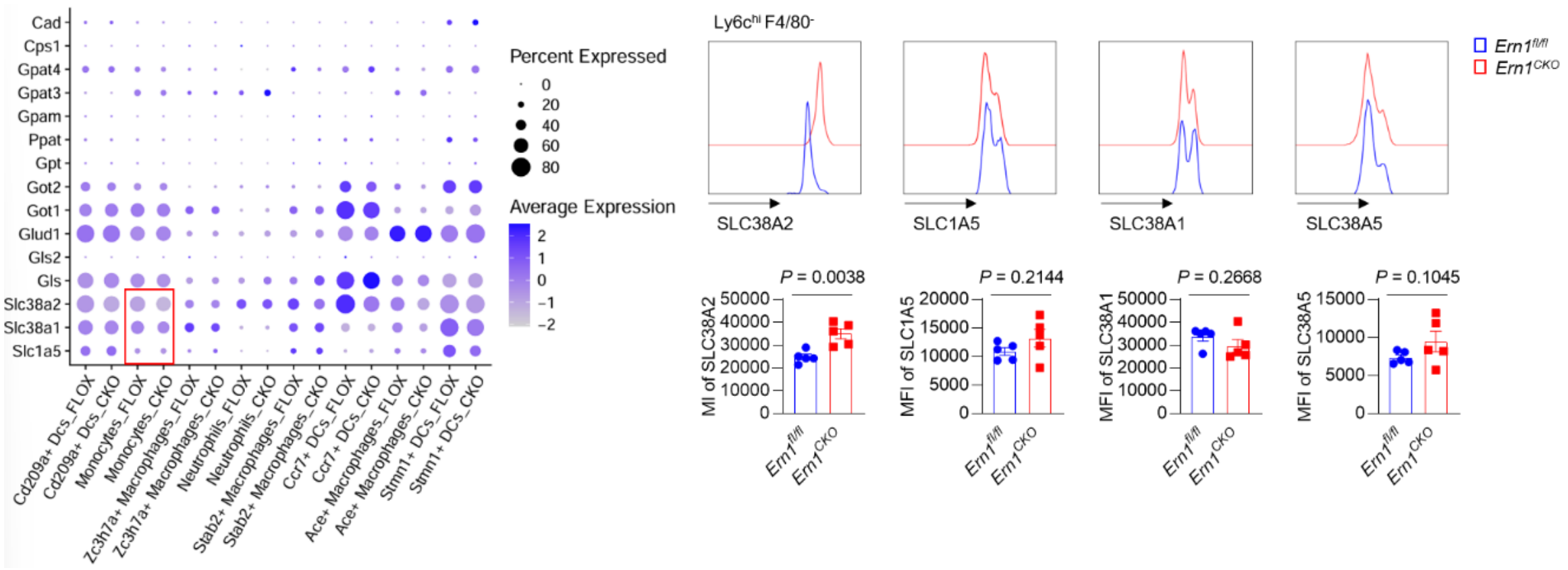

3. Mislabeled axes in Extended Fig. 1j

Extended Fig. 1j is mislabeled; the bar graph should indicate ERN1 flox and ERN1 CKO.

Response: We appreciate the reviewer's careful scrutiny and have corrected this error accordingly; the revised data are now presented in **Extended Data Fig. 2i**.

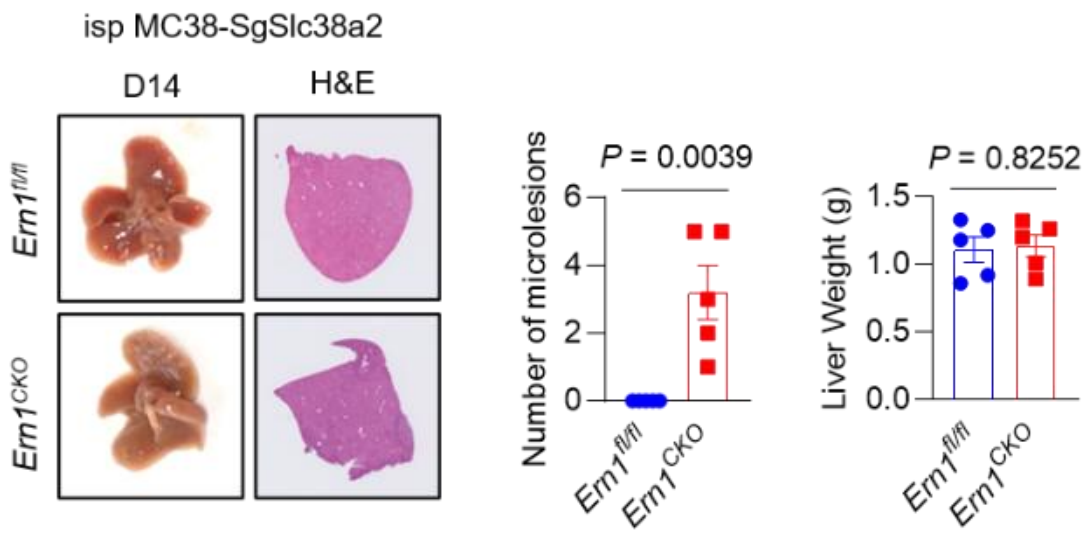

4. Verification of immune cell depletion

The authors should provide data confirming the depletion of CD8⁺ T cells, NK cells, and monocytes following administration of anti-CD8, anti-NK1.1, and anti-Ly6C antibodies, respectively.

Response: We confirmed efficient depletion of CD8⁺ T cells, NK cells, and monocytes in metastases-bearing mouse livers following administration of anti-CD8, anti-NK1.1, and anti-Ly6C antibodies (on Days -1, 3 and 6), respectively. The efficacy of depletion was validated by flow cytometric analysis, and the corresponding data are provided below.

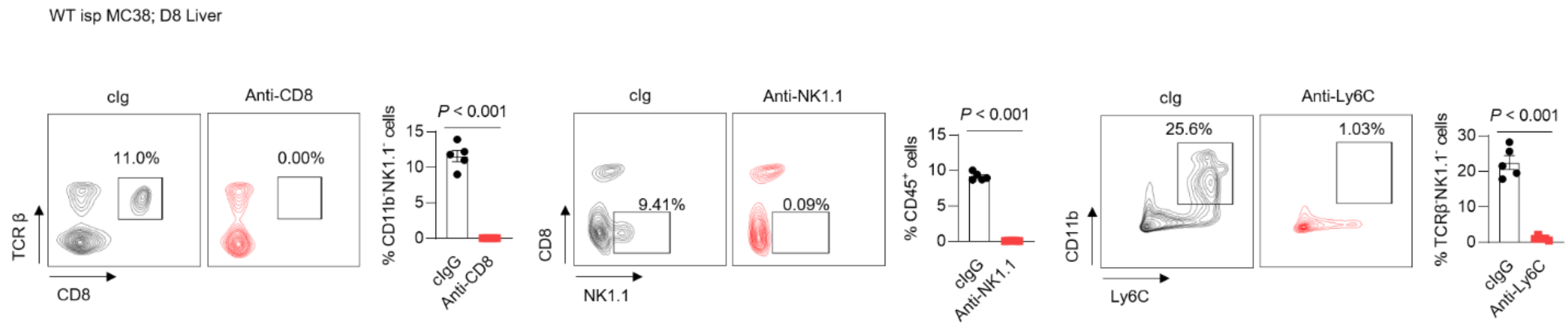

5. Flow cytometry axis inconsistency (Extended Fig. 4b)

In Extended Fig. 4b, the Day 7 CCR2/Ly6C dot plot appears to use a different X-axis scale compared to the adjacent plot. This should be corrected for consistency.

Response: Now the data display has been corrected for consistency, as shown in **Extended Data Fig. 5b**.

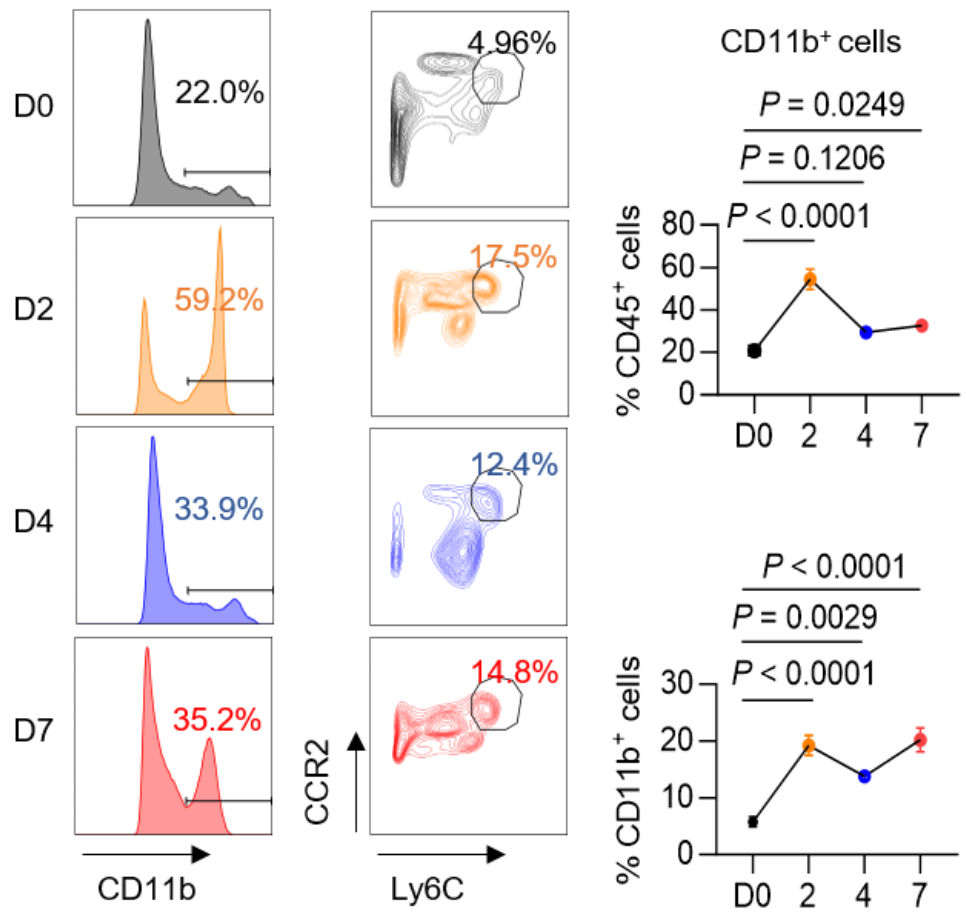

6. Figure 3e model consistency

Fig. 3e should be replaced with Extended Fig. 4c to maintain consistency of the experimental model across the figure.

Response: We have accordingly replaced **Fig. 3e** with **Extended Data Fig. 5e** (previously Extended Data Fig. 4c) to improve data presentation (*Ern1*^{CKO} in main figures and *Xbp1*^{CKO} in Extended Data).

7. Inclusion of WT controls in Fig. 4b–c

Fig. 4b and Fig. 4c should include WT controls to demonstrate the decrease in monocyte recruitment and its impact on tumor burden.

Response: The wild-type control data corresponding to **Fig. 4b** are presented in **Fig. 4a**. In CCR2-deficient mice, the Ly6C^{+} compartment comprises a different composition of immune populations and is therefore not directly comparable to that of wild-type mice. Accordingly, we quantified CD11b^{+}CCR2^{+}Ly6C^{hi} cells in **Fig. 4a**, whereas CD11b^{+}Ly6C^{+} cells were analyzed in **Fig. 4b**. In this experiment, the primary comparison was between glutamine supplementation and non-supplementation conditions rather than between genotypes.

The wild-type control data corresponding to **Fig. 4c** can be found in **Extended Data Fig. 2j**. To facilitate direct comparison, we have now included an independent wild-type control experiment in the revised **Fig. 4c**.

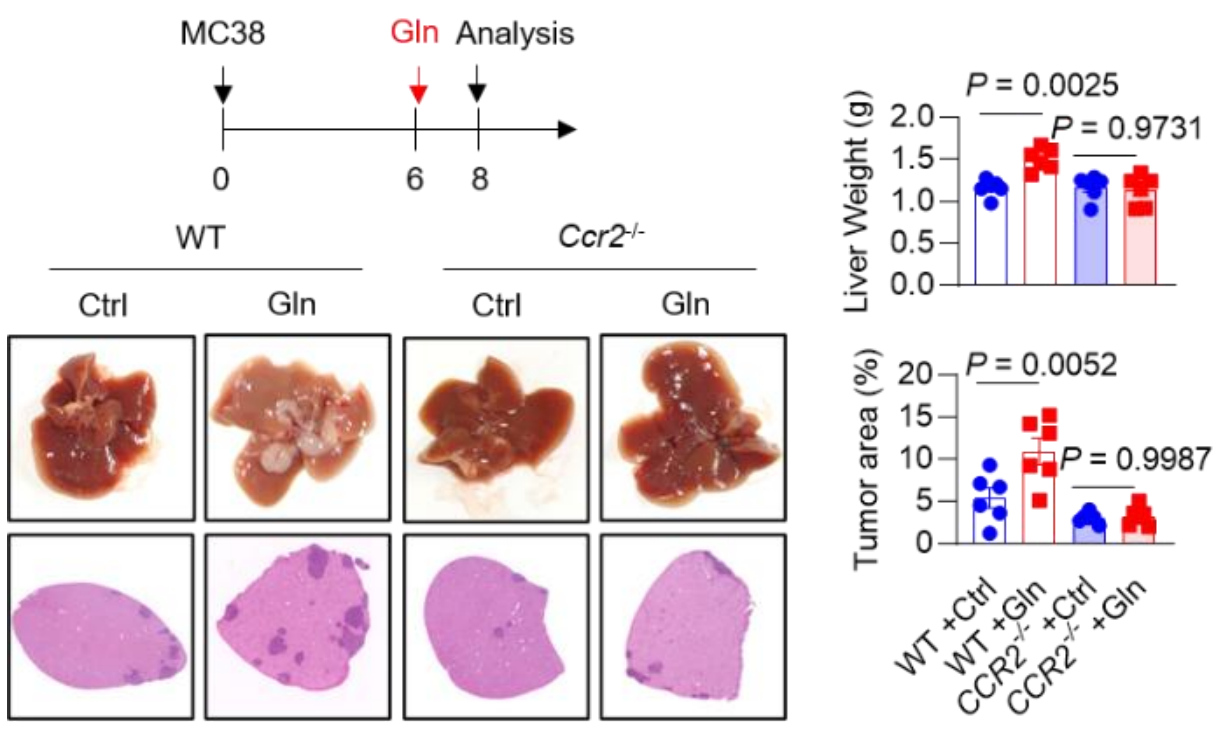

8. CCR2 KO and Gln supplementation experiment

For CCR2 KO mice, it would be informative to include outcomes at later time points (e.g., Day 14) to determine whether Gln supplementation eventually promotes tumor growth, given the ability of tumor cells to utilize Gln for proliferation.

Response: We appreciate the reviewer's suggestion and have therefore included data from a later time point. Notably, glutamine supplementation still failed to promote tumor progression in CCR2-deficient

mice, further supporting the requirement for CCR2⁺ monocytes in mediating the pro-metastatic effects of glutamine (the corresponding wild-type cohort was shown in Fig. 2f).

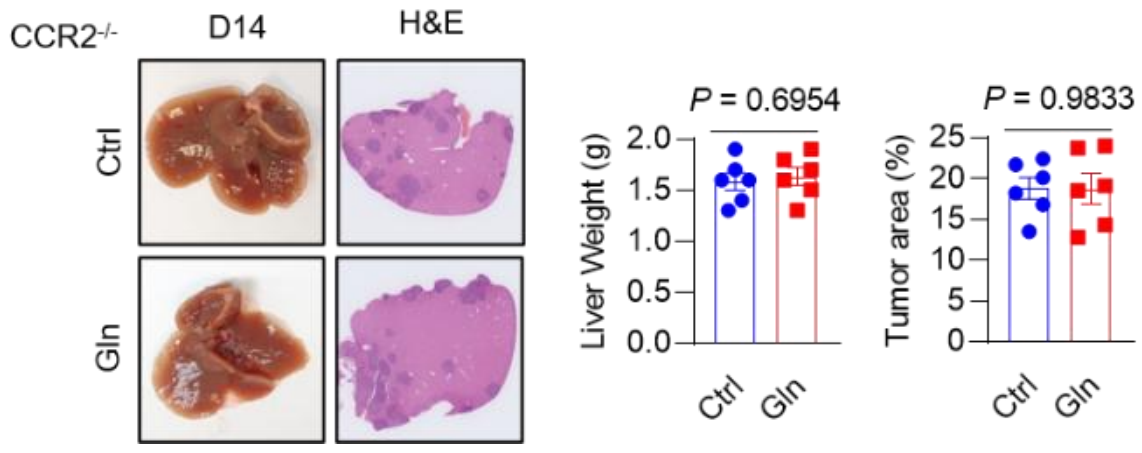

9. Missing Y-axis label in Fig. 4b

In Fig. 4b, the Y-axis label is missing for the Ki67 plot. If the axis represents Ly6C, this should be clearly indicated. Additionally, the first bar graph should be labeled as CD11b⁺Ly6C⁺ on top.

Response: We appreciate the reviewer's careful scrutiny. The Y-axis of the Ki67 plot represents Ly6C. We have now correctly annotated the corresponding gating/population as CD11b⁺Ly6C⁺ above both the Ki67 plot and the first bar graph in the revised **Fig. 4b**.

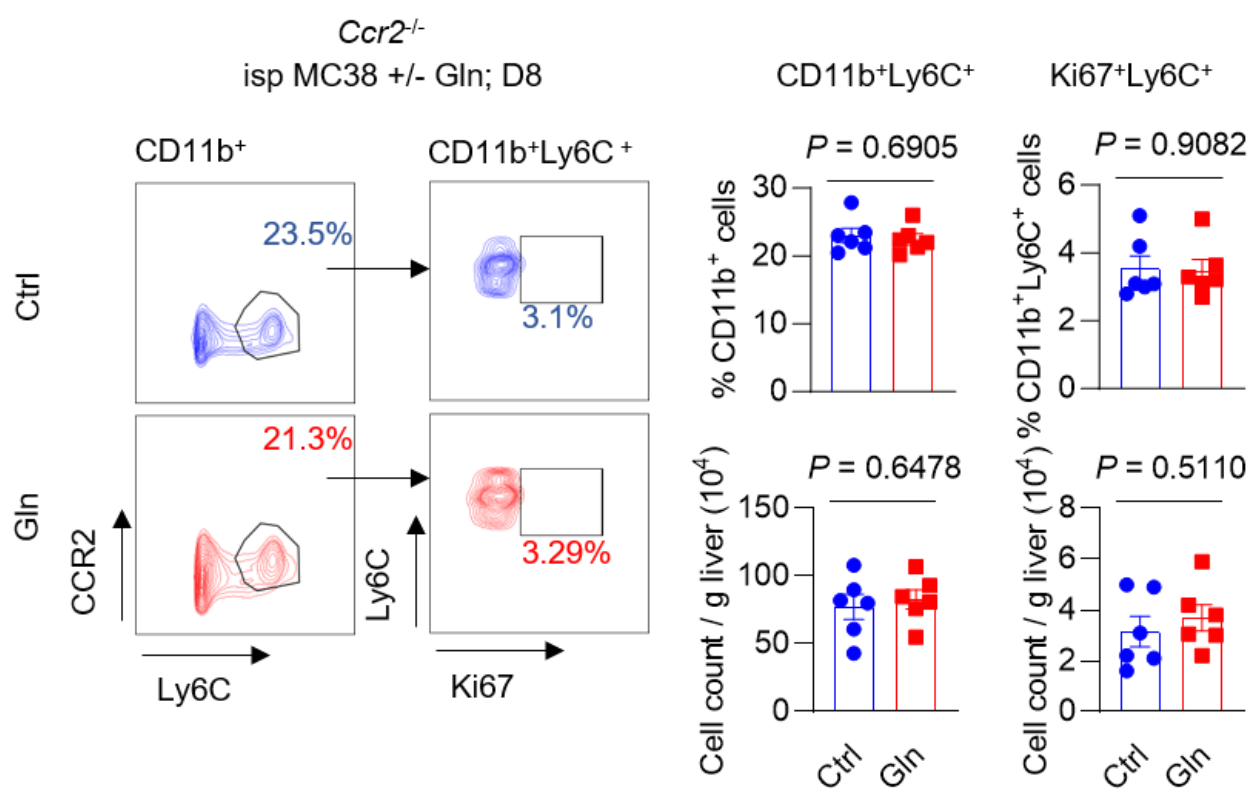

10. Missing sgCtrl control in Extended Fig. 5g

Extended Fig. 5g is incomplete without the sgCtrl control group and should be revised accordingly.

Response: We appreciate the reviewer's concern and have redone this experiment. We co-transferred sgCtrl-expressing CD45.1/2 and sgSlc38a2-expressing CD45.2 BMIMs to prove that IM expansion depends on glutamine uptake (the revised data are now presented in **Fig. 5e**).

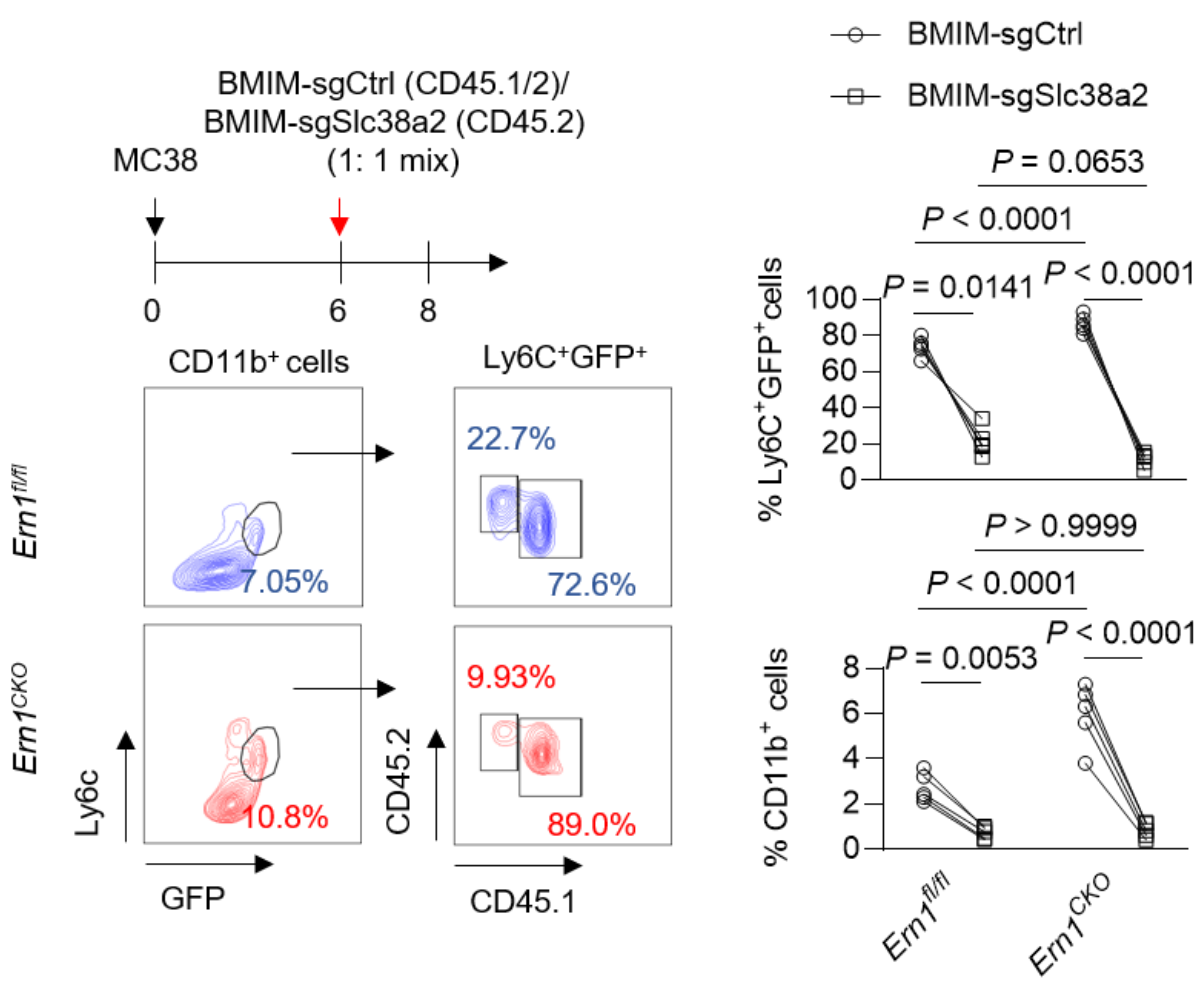

11. Mislabeled cells in Extended Fig. 5g

Extended Fig. 5g incorrectly identifies the cells as CTV-labeled; this should be corrected.

Response: We appreciate the reviewer's careful scrutiny and have corrected this error accordingly (**Fig. 5e**).

12. Overstated conclusion in Fig. 7a

The statement that "hepatocyte–IM communication did not exist in Ern1fl/fl or Xbp1fl/fl livers" is not fully supported by the data. Ly6C⁺ cells are still present in proximity to hepatocytes, and the images do not provide direct evidence of "communication." This conclusion should be moderated.

Response: We appreciate the reviewer's suggestion and have accordingly moderated the statement to read: "GS-expressing peritumoral hepatocytes and proximally infiltrating monocytes were less present in *Ern1*$^{fl/fl}$ and *Xbp1*$^{fl/fl}$ livers" (**Line 405–407**).

Reviewer #3 (Remarks to the Author):

In this manuscript, Zhang and colleagues uncover a metastasis-suppressing role for the IRE1-XBP1 axis in hepatocytes. In absence of XBP1, Foxo1 directs the expression of glutamine synthetase in response to tumor-induced glutamine starvation. In turn, increased glutamine concentration promotes metastasis growth and the accumulation of CCR2+ monocytes. Collectively, the authors have conducted a broad analysis of the effects of glutamine synthesis on hepatic metastasis. The findings are novel, however I find that the conclusions are sometimes overstated and speculative, and the data overly interpreted. Most prominently, I don't think that the experiments presented in the manuscript are enough to dissect the effect of glutamine synthesis on seeding vs growth of metastases. Moreover, the

authors should investigate the mechanisms of monocyte recruitment/accumulation further, as well as dissect how monocytes exert their antitumorigenic functions. I hope the following comments will help authors improve their manuscript.

Response: We thank the reviewer for the constructive critique.

Figure 1:

a) Minor points:

- It is mentioned in the text that MKC8866 is and RNAse inhibitor, while in the introduction the authors only mention the enzymatic activity of IRE1. An explication of the dual role of IRE1 as kinase and splicing factor of Xbp1 would help the clarity of the manuscript.

Response: We appreciate this advice and have added an explication in the Introduction section: "Although IRE1α also engages the regulated IRE1α-dependent RNA decay (RIDD) pathway, the therapeutic focus in cancer has largely centered on the IRE1α–XBP1s axis" (**Line 66–68**).

- Small note regarding nomenclature: to simplify I would simply abbreviate Alb-Cre;Ern1flox/flox mice as Ern1KO and Ern1flox/flox as Ern1wt.

Response: We defined the abbreviation at its first occurrence (**Line 134**) as follows: "*Ern1*$^{flox/flox}$;*Alb*^{Cre}, hereafter referred to as *Ern1*^{CKO}," and consistently used "*Ern1*^{CKO}" throughout the text. Correspondingly, we used "*Ern1*$^{fl/fl}$" to denote *Ern1*$^{flox/flox}$ mice. These floxed mice served as the appropriate genetic controls and are distinct from WT mice. We therefore retained this nomenclature to ensure genetic accuracy and clarity.

e) They authors delete Xbp1, observe a similar phenotype than with Ern1 deletion, and conclude that Xbp1 is the main downstream effector. However, if suggested, epistasis should be formally tested, for example by injecting Xbp1-overexpressing AAV in Ern1Cko mice.

Response: We appreciate this suggestion and have tested the proposed epistasis in *Ern1*^{CKO} mice. Consistent with our observations in wild-type mice, XBP1s overexpression significantly suppressed metastatic progression and reduced monocyte accumulation in the liver, supporting an epistatic relationship between IRE1 and XBP1s in this context (**Extended Data Fig. 1e, 7c** and **Fig. 7b**).

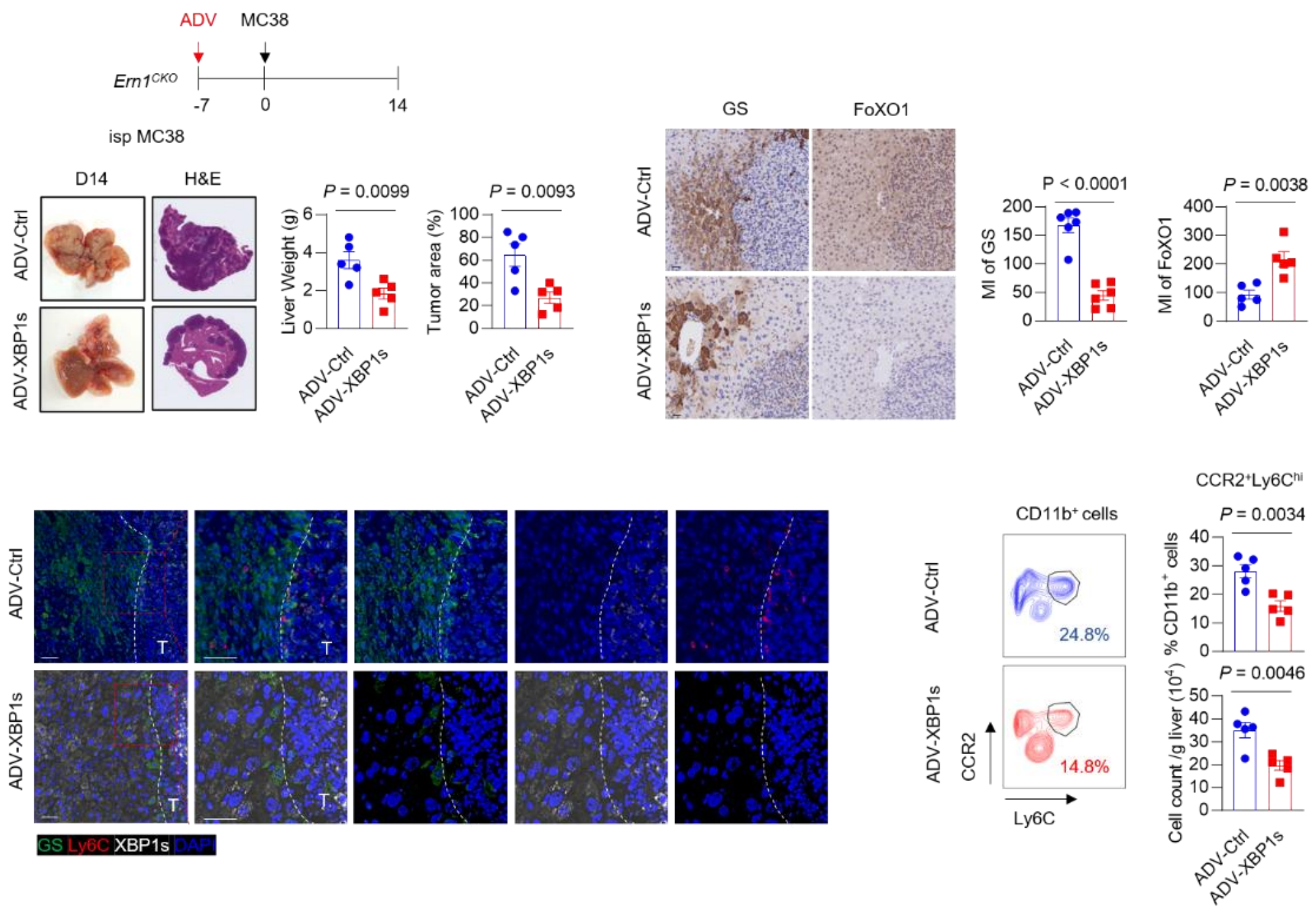

Minor point:

- The authors should mention in the methods which adenovirus capsid they used.

Response: Our adenoviral vector uses the Adenovirus Type 5 (Ad5) capsid, which possesses a natural and potent tropism for the liver. Please see from **Line 566–568** in the revised manuscript.

Figure 2:

a) Both tumor area and tumor foci are increased at D7, therefore the authors cannot exclude that there were more lesions at D2, but they were simply too small to be picked up. The authors mention in the text that metastatic foci were markedly larger, but they quantify tumor area. I would suggest that the authors divide the tumor area by number of foci to obtain the average lesion size. In general, authors should stick to the same quantification method for metastases throughout all figures. In figure 1 it is liver weight and tumor area, in figure 2 it is metastasis numbers. The authors should try to quantify tumor foci and obtain average tumor size for their Figure 1 experiments.

Response: To address the reviewer's concern, we have now quantified the average lesion size by normalizing tumor area to the number of metastatic foci, which was still comparable between *Ern1*$^{fl/fl}$ and *Ern1*^{CKO} at day 2 (**Fig. 2a**).

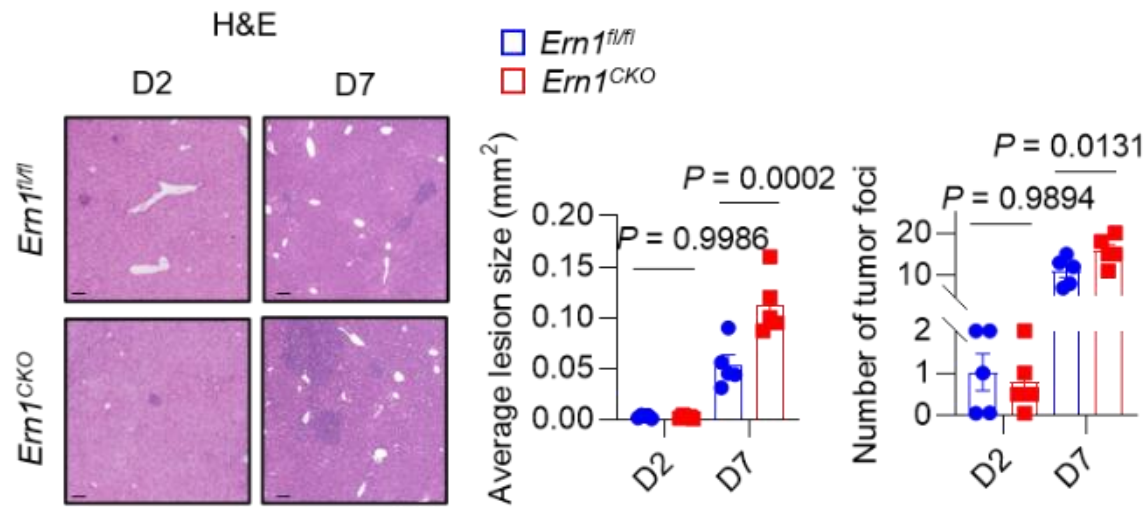

Minor points:

o Was quantification performed on two or more non-consecutive sections?

o Was it carried out always on the same lobe?

o For fair comparison, the same lobe should be shown in all figures.

Response: Adjustment We appreciate the reviewer's concern. Quantification was performed on sections collected from the same liver lobe across experimental groups to ensure consistency. Metastatic burden was determined from H&E-stained sections and expressed as the percentage of tumor area relative to total liver area. We have now clarified this point in the Methods section (**Line 597–599**). In addition, representative images were selected from the corresponding liver lobe across groups to facilitate direct comparison.

b) Authors mention survival, however Ki67 is a measure of proliferation. It is impossible to distinguish from H&E stainings whether Ki67+ cells are tumor cells or infiltrating immune cells. The authors should include a pancytokeratin staining and only quantify Ki67 positive cells among those. Especially since there is an accumulation of monocytes.

Response: We appreciate the reviewer's concern. We agree that Ki67 is a marker of proliferation rather than survival and that assessment of Ki67 alone does not distinguish tumor cells from infiltrating immune cells. To address this point, we conducted pancytokeratin staining; however, in our specimens pancytokeratin was also detected in multiple non-tumor cell populations, making it unsuitable for reliable identification of tumor cells in this setting.

As shown in **Fig. 2b**, the majority of Ki67^{+} nuclei quantified in metastatic lesions were associated with cells displaying the characteristic morphology and localization of tumor cells. Importantly, our conclusion that the *Ern1*^{CKO} liver provides a more permissive environment for metastatic progression does not rely on Ki67 staining. Instead, it is supported by multiple independent observations. We have revised the text to clarify that this interpretation should be considered supportive rather than definitive evidence of enhanced tumor cell proliferation (**Line 161–166**).

c) The authors state in the introduction that XBP1 is critical for maintaining physiological liver functions. However, they do not assess these liver metabolic functions in Xbp1 or Ern1 KO mice prior to tumor inoculation. Constitutive deletion of these factors might induce imbalances in the liver that make it more susceptible to colonization. The authors should measure metabolic parameters by LC-MS in KO vs WT livers prior to tumor colonization and subtract those baseline differences to those observed in peritumoral areas.

Response: We performed additional metabolomic analyses on tumor-free *Ern1$^{fl/f}$* and *Ern1^{CKO}* livers and found that glutamine was not among the differentially abundant metabolites (data are provided in revised **Supplementary Table 2**). Consistent with previous omics studies of hepatocyte IRE1α deficiency (PMID: 18556558; 23040070), the major metabolic differences between naïve *Ern1$^{fl/f}$* and *Ern1^{CKO}* livers were associated primarily with lipid metabolism rather than amino acid metabolism. These findings suggest that glutamine accumulation is not an intrinsic feature of the tumor-free *Ern1^{CKO}* liver but instead emerges in the context of metastatic progression and the evolving tumor microenvironment.

Minor points:

- How were peritumoral areas defined? How were tumors cells excluded?

Response: The peritumoral region was defined as tissue located within a 5 mm radius of the tumor margin; this criterion has been added to the Methods section (**Line 653**). Tumors were macroscopically distinguishable from surrounding liver tissue at the time of collection for metabolomic analysis (Day 7). In parallel, adjacent liver tissues were subjected to H&E staining to confirm the exclusion of tumor cells.

- In the volcano plot of figure 2C, the authors should label all significantly enriched metabolites/endogenous energy sources.

Response: The other enriched metabolites with the potential to serve as extracellular energy sources were palmitoylcarnitine and creatine, which have now been labeled in the revised **Fig. 2c**. However, the degree of their enrichment was substantially lower than that of glutamine.

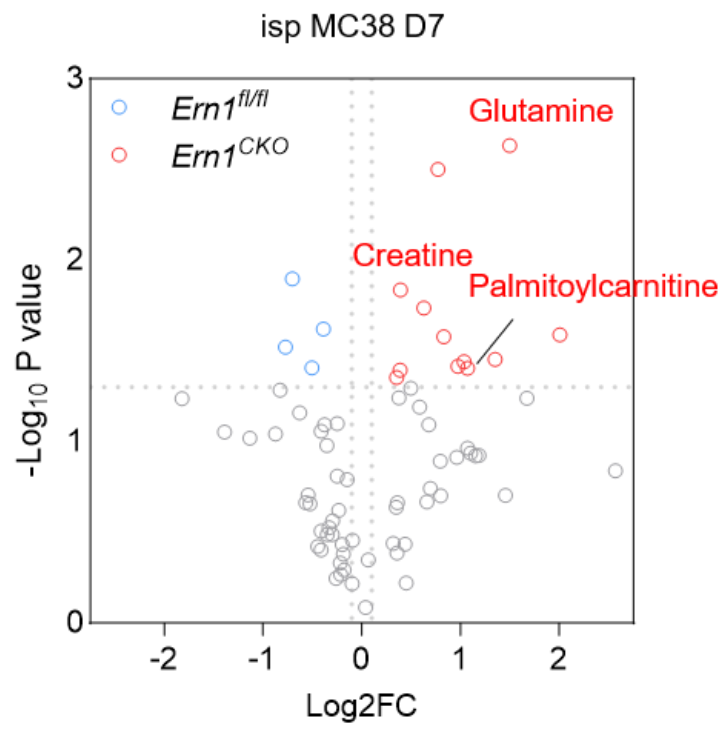

- The authors conclude that glutamine is actively consumed by the pro-metastatic niche but then go on to show that cancer cells heavily rely on it, and also refer to figure 2b. So, it would be more accurate to say that the tumor actively consumes glutamine. Further experiments are needed to elucidate whether the niche also consumes glutamine, for example measuring glutamine levels after monocyte depletion or in Ccr2 KO mice.

Response: We appreciate the reviewer's advice and have accordingly removed speculative statements regarding glutamine consumption, instead restricting our description to the objective observation of glutamine enrichment/accumulation in the metastatic niche (**Line 178–179**).

We additionally measured glutamine levels following monocyte depletion; however, glutamine concentrations in adjacent liver lysates were not altered. This is likely attributable to differences in metastatic progression between groups, as monocyte depletion exerted an anti-metastatic effect, potentially confounding the intrahepatic glutamine synthesis program in the upstream.

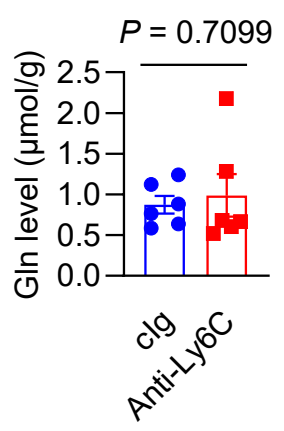

Although cancer cells are known to strongly depend on glutamine uptake, we hypothesized that the liver metastatic niche may uniquely benefit from extra glutamine availability (**Line 193**), as observed in IRE1-deficient livers. Notably, glutamine supplementation at a mid-term time point (day 6), but not at an early time point (day 1), increased liver metastatic burden (**Fig. 2e, f**). This finding highlights a specific context in which extra glutamine promotes the formation of a pro-metastatic niche. Importantly, in *Ccr2*$^{-/-}$ mice, glutamine supplementation failed to enhance liver metastatic progression, underscoring the critical role of CCR2^{+} monocytes as functional mediators of glutamine's pro-metastatic effects

(**Fig. 4c**). This conclusion is further supported by in vivo tracing experiments demonstrating that monocytes lacking the glutamine transporter SLC38A2 fail to expand and promote metastasis (**Fig. 5e**), providing direct evidence that monocyte-intrinsic glutamine uptake is required for this process.

d) Authors delete SLC38A2 form MC38 cells and show that those completely fail to metastasize. This, and the fact that increased tumor foci are observed in Ern1 KO mice actually indicates that seeding is also affected by IRE1 deficiency. I am not entirely sure that Fig 2a alone justifies the conclusion that seeding is not affected. The authors should dissect this point further, if they want to stress it.

Response: We respectfully disagree with the reviewer's interpretation. While our MC38-sgSlc38a2 experiments demonstrate that tumor-intrinsic glutamine uptake is required for successful liver colonization, necessity does not imply that increasing environmental glutamine promotes metastatic seeding.

We agree that H&E analysis at day 2 cannot definitively exclude subtle differences in metastatic seeding. Our intention was not to conclude that seeding is completely unaffected, but rather that no appreciable difference was detectable at this early time point. We have therefore moderated the corresponding statement in the revised manuscript (**Line 156–158**). Importantly, this, together with the emergence of significant differences only at later time points and the selective efficacy of day 6 (but not day 1) glutamine supplementation, collectively support the interpretation that hepatic IRE1α deficiency primarily affects metastatic outgrowth rather than early metastatic establishment.

Minor points:

- How was the fitness and proliferation of these SLC38A2 KO cells in vitro?

Response: The proliferation of SLC38A2-deficient MC38 cells was modestly reduced compared with control cells. Their dependence on glutamine uptake appears less pronounced in vitro than in vivo, likely because standard culture media provide supraphysiological levels of nutrients, including glucose and other amino acids, which can partially compensate for impaired glutamine utilization.

- Extended Data Fig 1j: quantification is either mislabeled or belonging to a different experiment.

Response: We appreciate the reviewer's careful scrutiny and have corrected the labels (now shown in **Extended Data Fig. 2i**).

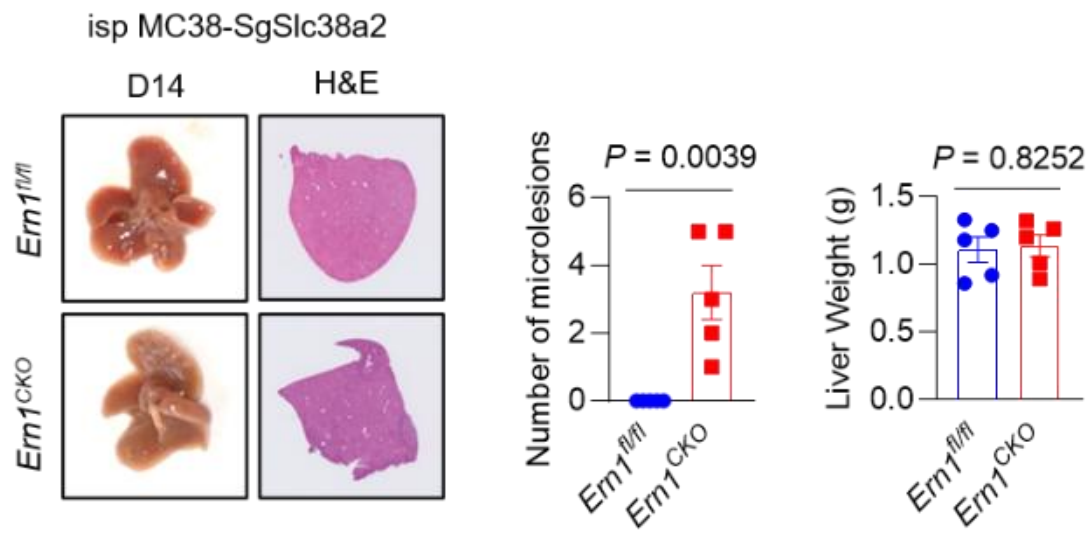

e) Metastatic seeding might happen within hours. Authors should supplement glutamine on the same day to make a statement about seeding.

Response: We appreciate the reviewer's concern. Although the earliest steps of metastatic seeding begin within hours of tumor cell arrival, metastatic establishment is a dynamic process that continues beyond the initial vascular arrest and includes survival, extravasation, and adaptation within the target organ. Therefore, glutamine supplementation on day 1 would still be expected to influence metastatic seeding if excess glutamine were a major determinant of this process.

Figure 3:

a) The authors should assess the baseline immune composition in non-tumor bearing Ern1 WT vs KO livers to assess whether altered liver metabolism affects resident immune cell types.

Response: Flow cytometry analysis revealed similarly low levels of monocyte infiltration in tumor-free *Ern1*$^{fl/fl}$ and *Ern1*^{CKO} livers (**Extended Data Fig. 5a**), as was also observed for other immune populations.

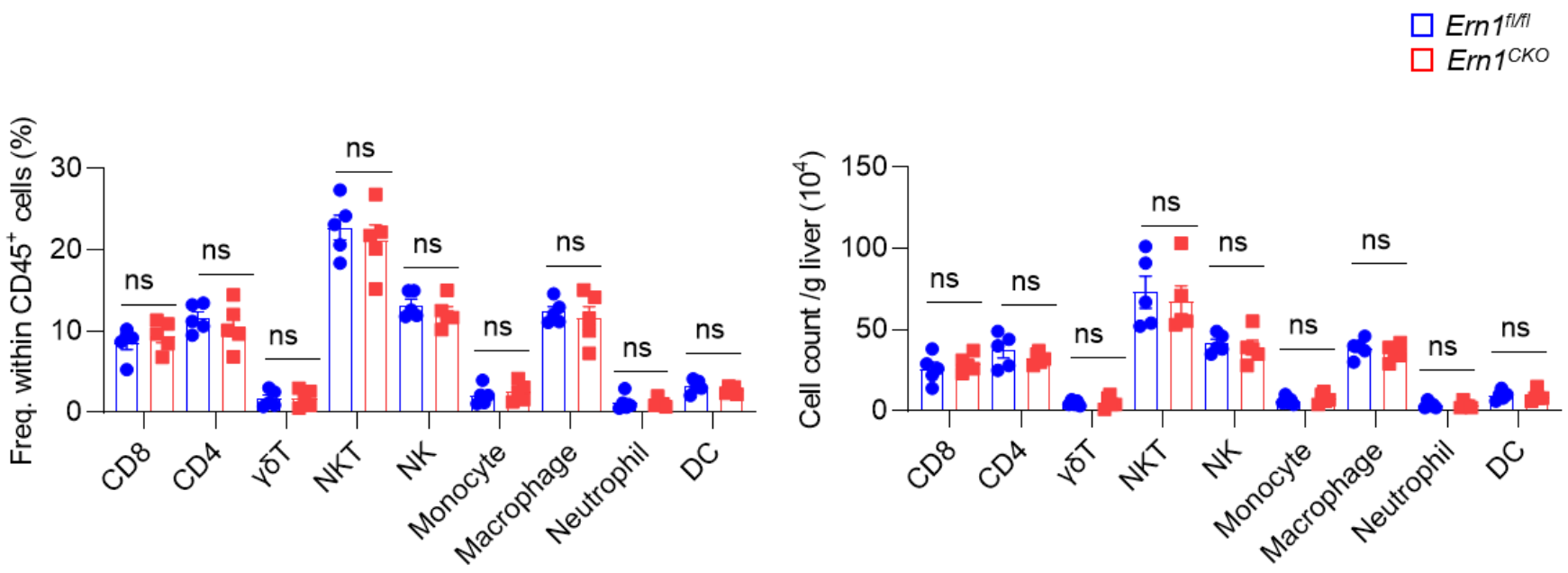

b) Extended data Figure 2b,c,d) authors should show the increase in tumor load in treated vs non-treated mice, not only the difference between Ern1 WT and KO mice.

Response: We appreciate the reviewer's suggestion that non-treated mice are needed in this immune depletion setting and therefore have added a control IgG treatment cohort (**Extended data Fig. 3c**) together with neutralizing antibody treatment cohorts (now shown in **Extended data Fig. 3d–f**).

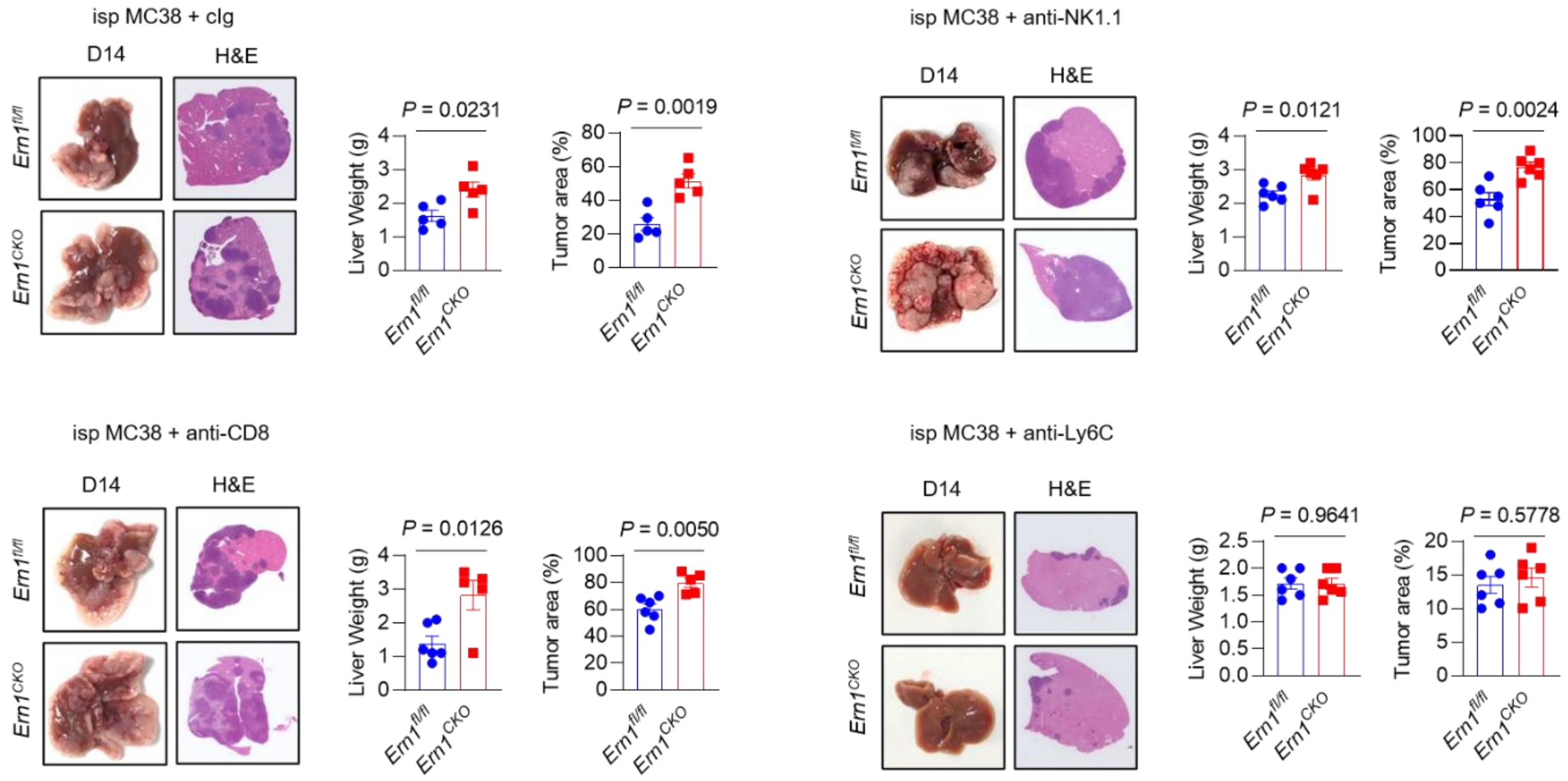

c) scRNAseq data shows an expansion of monocyte compartment at expense of neutrophil and DC compartment; this would indicate that monocytes recruited to the tumor fail to differentiate or exhibit delayed differentiation. However, this is not evident by flow, where macrophages are also slightly increased. The authors should investigate whether the accumulation of monocytes is due to increased influx or delayed differentiation.

Response: We appreciate the reviewer's insight. In the presence of extra glutamine provided by the *Ern1*^{CKO} liver, monocytes indeed exhibited a more inflammatory and proliferative phenotype. However, this does not imply a delay in their differentiation, as normal differentiation trajectories were evident in our pseudotime analysis (revised **Extended Data Fig. 4c**). Rather than impaired or delayed monocyte differentiation, the apparent changes reflect a relative dilution of other myeloid populations due to the selective expansion of inflammatory monocytes.

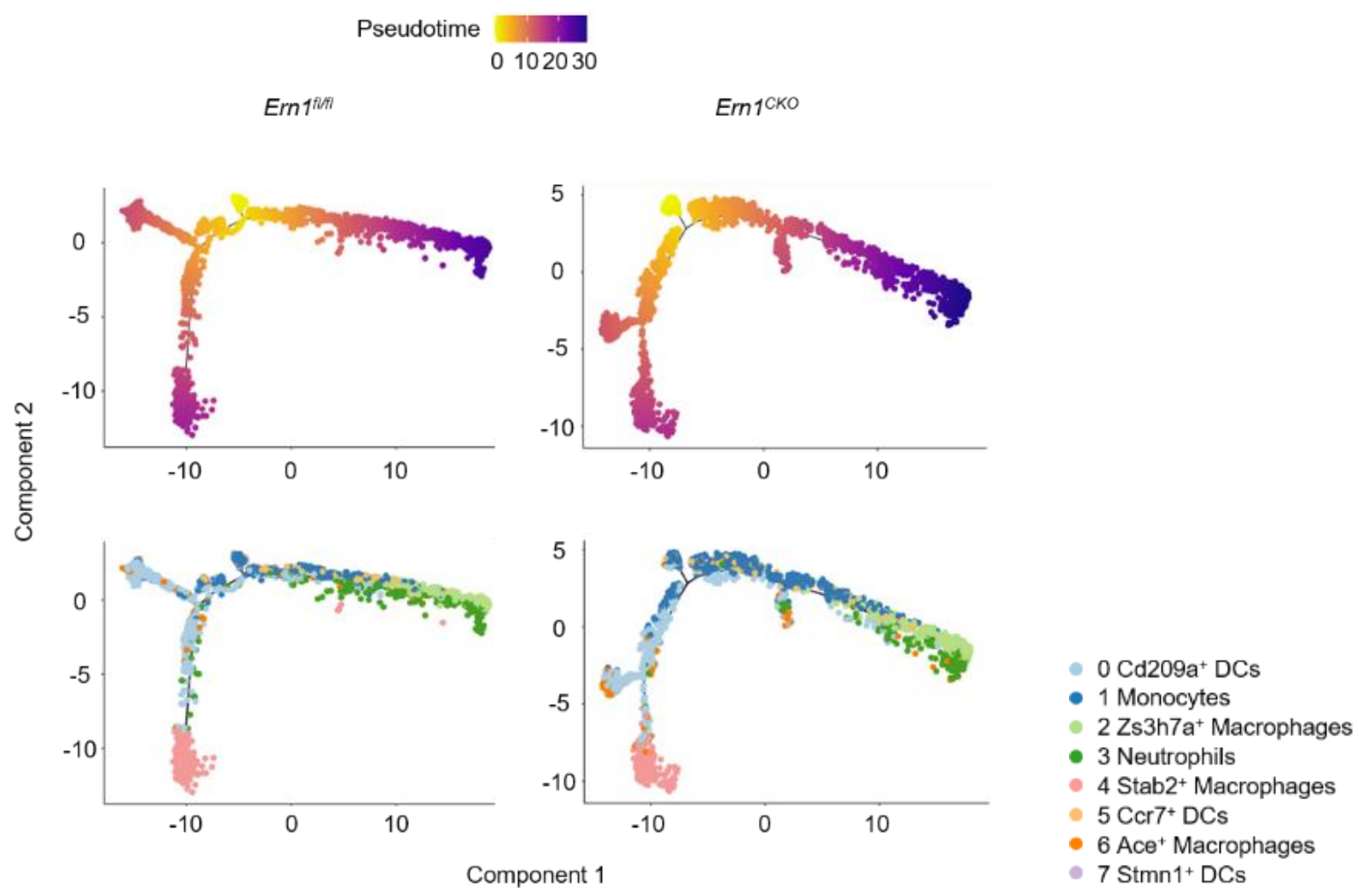

Extended data Figure 3c) did authors subset the two experimental conditions, re-scale and re-normalize and only then calculate monocle trajectories separately? It looks like a unique trajectory onto which cells of either conditions were subjected. This would represent an average trajectory, while two distinct trajectories should be calculated, and then can be aligned within Monocle.

Response: As suggested by the reviewer, we reanalyzed the two groups separately, performing independent normalization, dimensionality reduction, and Monocle trajectory reconstruction within each dataset. As shown in revised **Extended Data Fig. 4c**, the overall differentiation direction and topological structure were preserved, with no evidence of trajectory disruption or failure of monocytes to connect to terminal cell states.

d) The authors should calculate logFC of Ccr2 across clusters of interests. What other genes are differentially expressed in monocytes between experimental conditions? What about cell cycle genes? Can the authors assess proliferation the clusters of interest?

Response: The log2 fold change of Ccr2 in comparison between $Ern1^{CKO}$ and $Ern1^{fl/fl}$ groups is 0.085. In fact, Ccr2 is used as a cell identity marker for inflammatory monocytes rather than as a differentially expressed gene. Accordingly, we interpret the primary difference between the two groups to lie in cell abundance, not in per-cell levels of pro-inflammatory gene expression. In contrast, per-cell expression of E2F target genes and ATP synthesis pathway genes was significantly elevated in the $Ern1^{CKO}$ group compared with the $Ern1^{fl/fl}$ group (**Extended data Fig. 4e**), providing a mechanistic explanation for the enhanced proliferative capacity of inflammatory monocytes in the $Ern1^{CKO}$ setting.

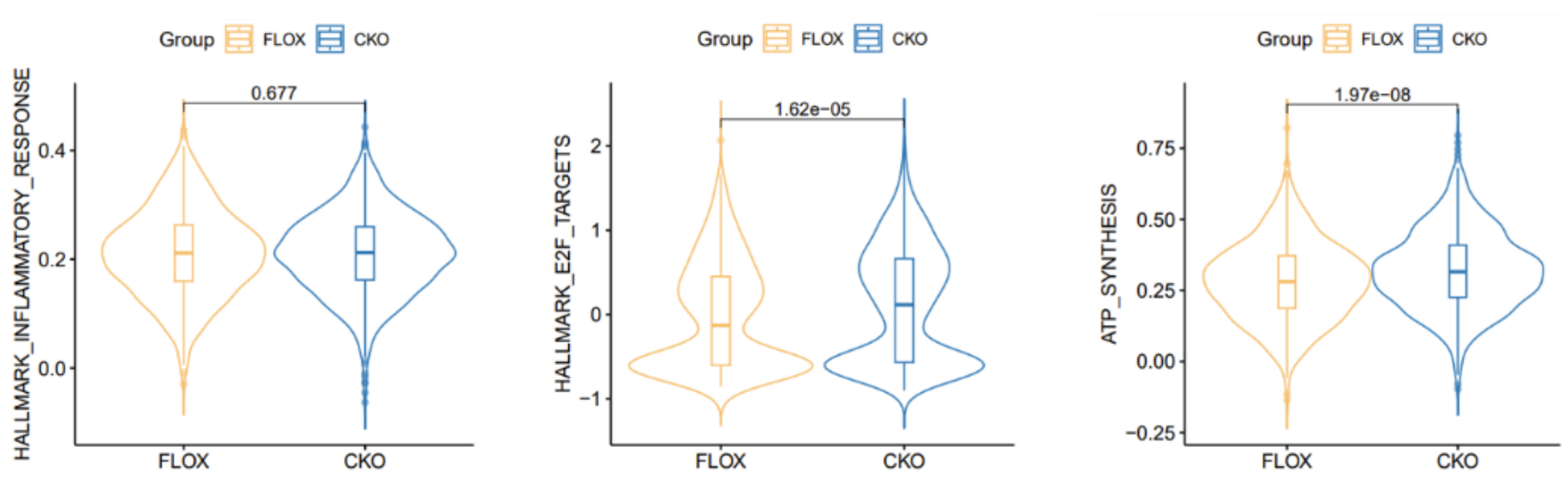

e) The accumulation of monocytes at day 2 suggests that these cells are important during seeding. Authors should quantify monocytes in Ern1 and Xbp1 KO mice at day 2 as well.

Response: As shown in **Extended Data Fig. 5e**, the percentages of liver CCR2$^+$ IMs on day 2 were comparable between $Ern1^{CKO}$ and $Ern1^{fl/fl}$ mice.

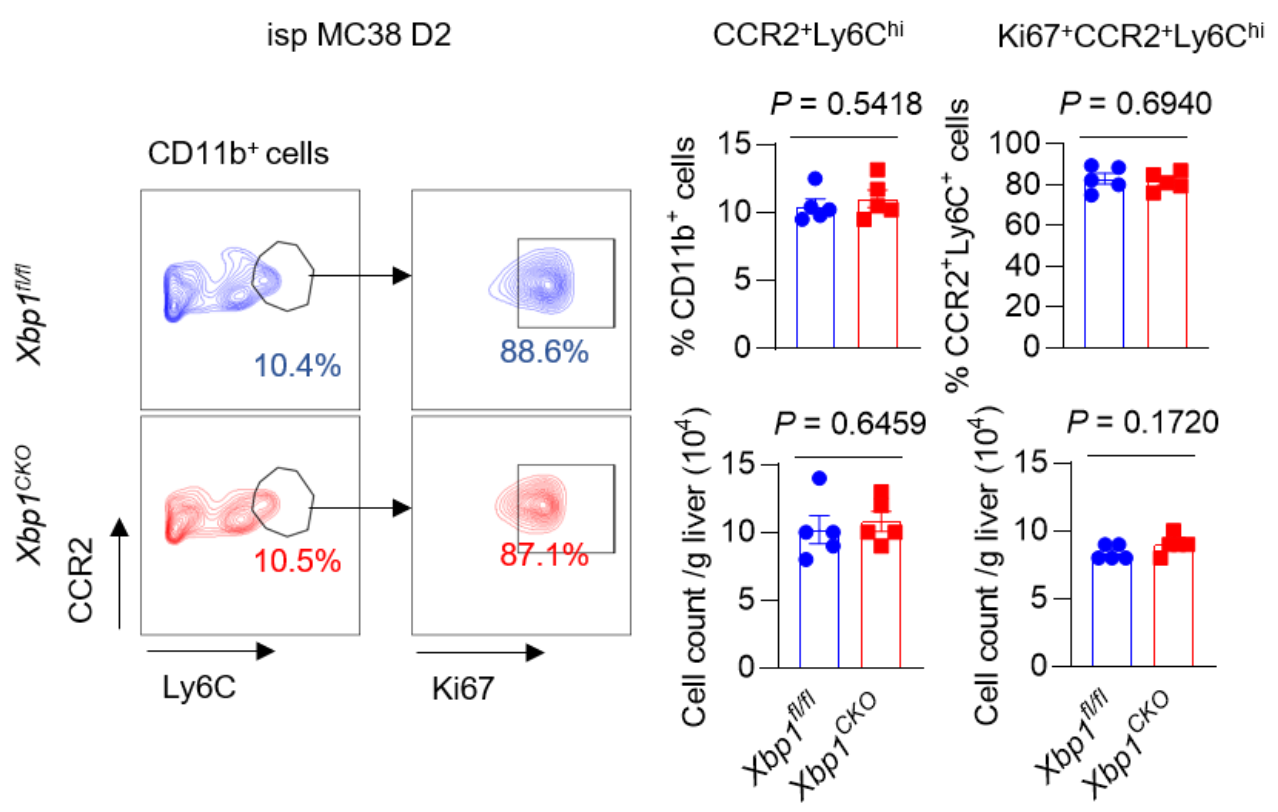

Further points:

- The authors mention a peak in monocytes in the tumor at day 2, however without a day 0 this cannot be called a peak, it might be the baseline.

- The suggested cause is acute inflammation, however the authors should include a sham operated control to support this statement. Was splenectomy performed after intrasplenic injection?

Response: We appreciate the reviewer's suggestion and have accordingly added a day 0 time point/sham control to **Extended Data Fig. 5b**. It indicates a baseline level of monocyte infiltration. Because splenectomy is performed following intrasplenic tumor injection in our model, mice in the sham group underwent splenectomy alone.

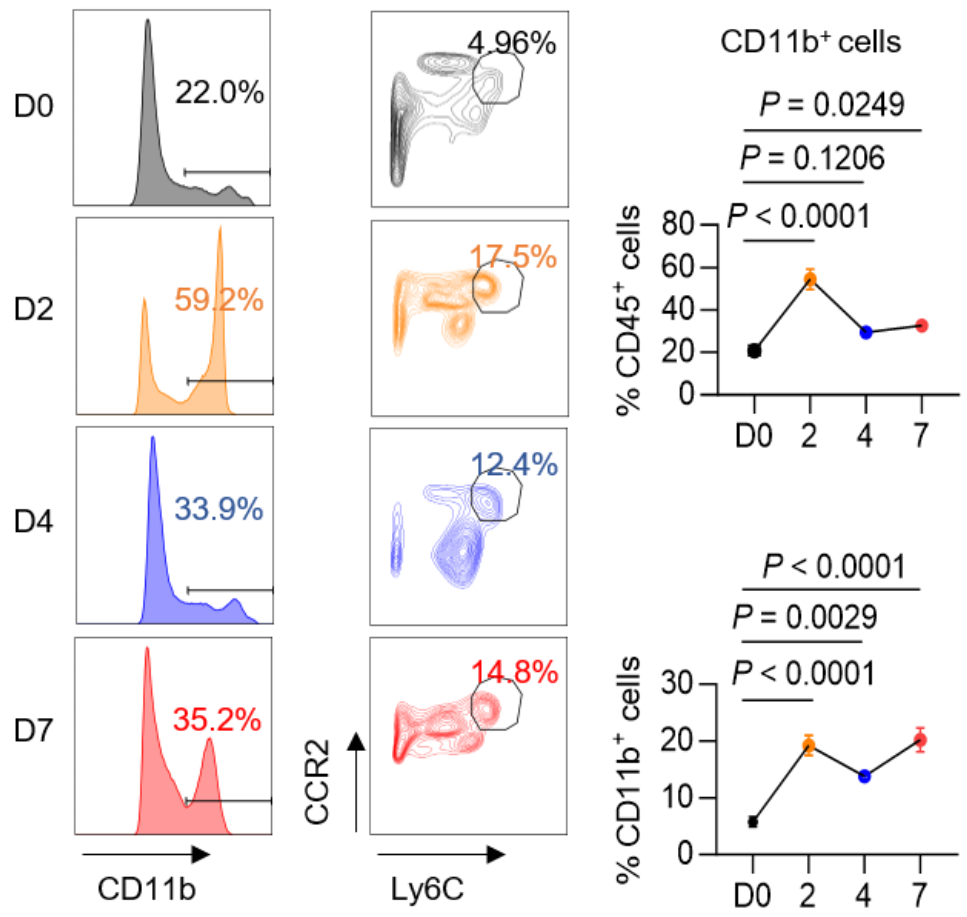

- For consistency, authors should show Ern1 KO mice in the main figure and Xbp1 KO in the supplementary.

Response: We have accordingly replaced **Fig. 3e** with **Extended Data Fig. 5e** (previously Extended Data Fig. 4c) to improve data presentation.

f) What about Ly6C negative CCR2 cells? They seem to be expanded in the KO too.

Response: In addition to monocytes (cluster 1), CCR2 expression was also detected in CD209+

dendritic cells (cluster 0), which appear to arise from monocytes (**Fig. 3d**). However, the abundance of cluster 0 cells was modestly reduced in the *Ern1*^{CKO} group compared with the *Ern1*$^{fl/fl}$ group (**Fig. 3c**).

Minor point:

- For clarity, individual channels should be shown in black and white, and the composite using colors.

Response: As suggested, we have revised the multiplex immunofluorescence figures to display the individual channels in grayscale and the merged images in color (**Fig. 3f**).

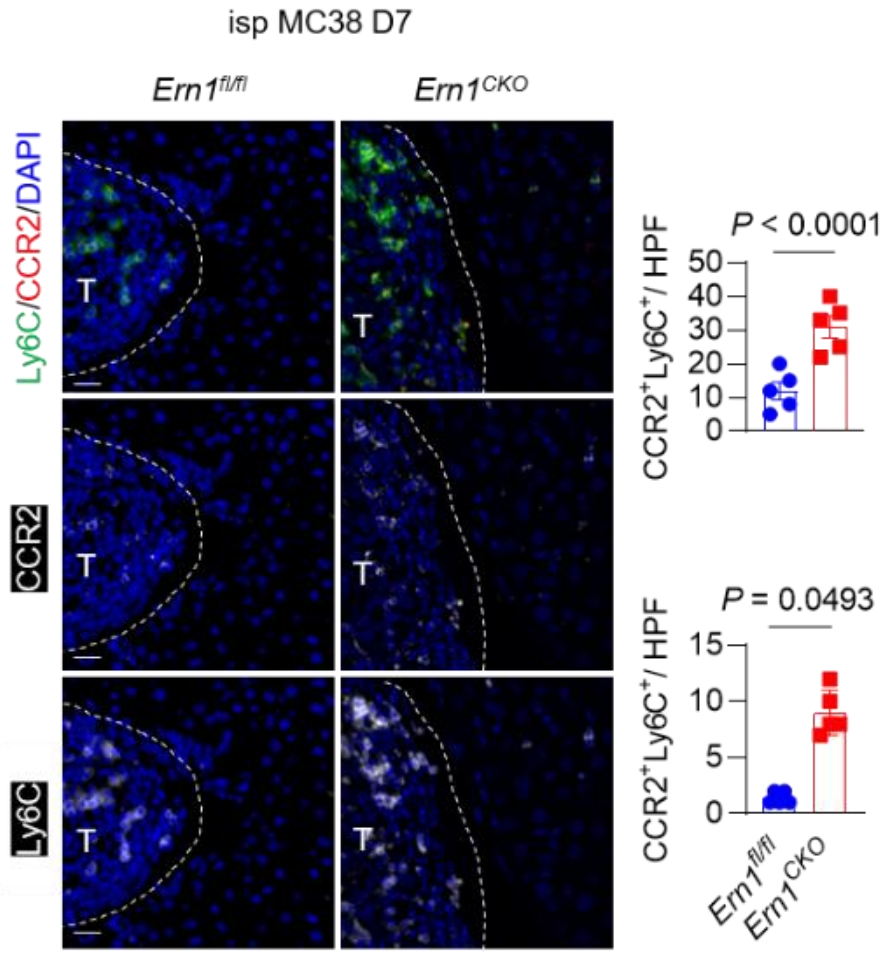

g) Also here, non-treated groups should be show.

Response: We appreciate the reviewer's suggestion and therefore have added vehicle-treated groups in this setting (**Fig. 3g**).

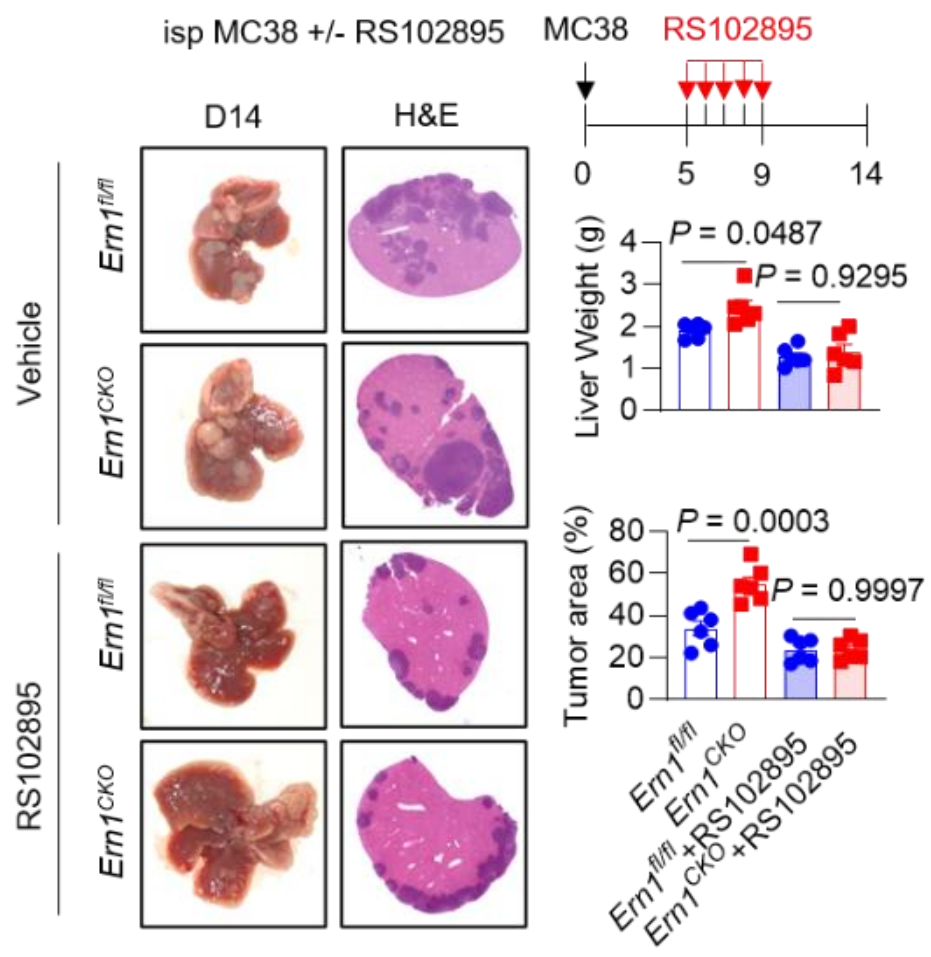

Extended data Fig.4 b and e) what is the Ccr2+Ly6Cintermediate population? It seems to be depleted in MKC treated mice. This would again suggest that monocytes recruited to the tumor fail to differentiate.

Response: As also indicated in **Fig. 3d**, the CCR2^{+}Ly6C^{int} population likely represents a subset of monocyte-derived dendritic cells. This population was altered upon IRE1α antagonism, consistent with prior evidence that intrinsic IRE1α–XBP1 signaling is required for tumor-associated dendritic cells

(PMID: 26073941).

Extended data Fig.4 f) The authors should use congenic markers of fluorophores for adoptive transfer, as CTV will be diluted if monocytes proliferate in situ. Adoptive transfer and fate mapping is instrumental to characterize the differentiation trajectories of monocytes in Ern1 KO vs WT mice.

Response: As suggested, we co-transferred sgCtrl-expressing CD45.1/2 and sgSlc38a2-expressing CD45.2 BMIMs to prove that IM expansion depends on glutamine uptake (**Fig. 5e**).

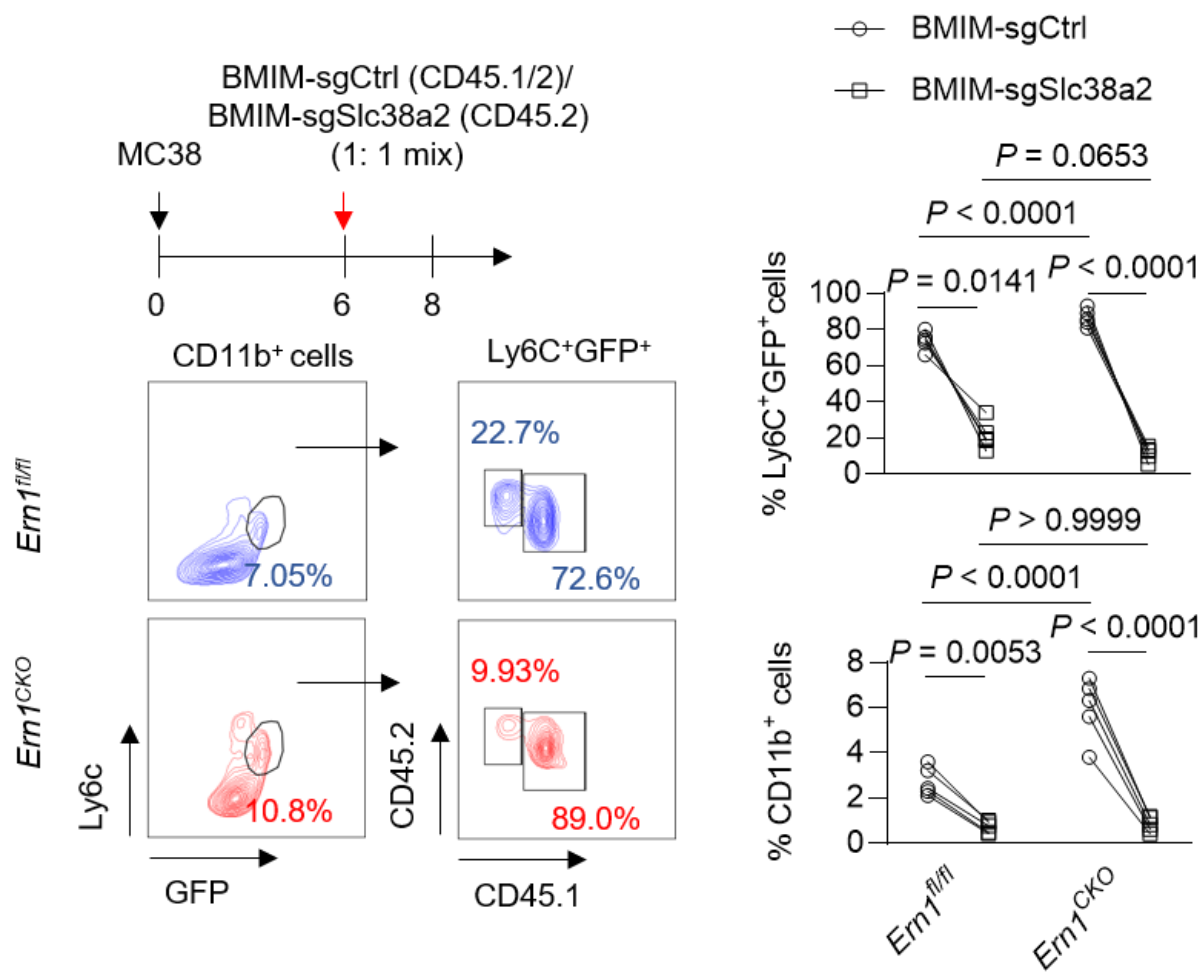

Minor point:

- The authors could also transfer monocytes from tumor bearing mice to assess systemic effects of increase glutamine production (see below).

- At the end of this paragraph, the authors state that monocytes can sustain a cancer-promoting inflammatory state. However, no data are provided to support this claim. It is unclear how monocyte accumulation promotes metastasis growth. Can the authors perform in vitro cell killing assays to show direct cytotoxicity of monocytes towards tumor cells?

Response: Our single-cell transcriptomic analyses supported a pro-inflammatory phenotype of liver monocytes (**Extended Data Fig. 4d**), characterized by elevated expression of multiple pro-inflammatory mediators, many of which have also been implicated in promoting tumor progression. Moreover, liver-derived CCR2$^+$ IMs enhanced MC38 proliferation in an ex vivo co-culture assay and promoted metastatic progression in an adoptive transfer model (**Extended Data Fig. 5c, d**).

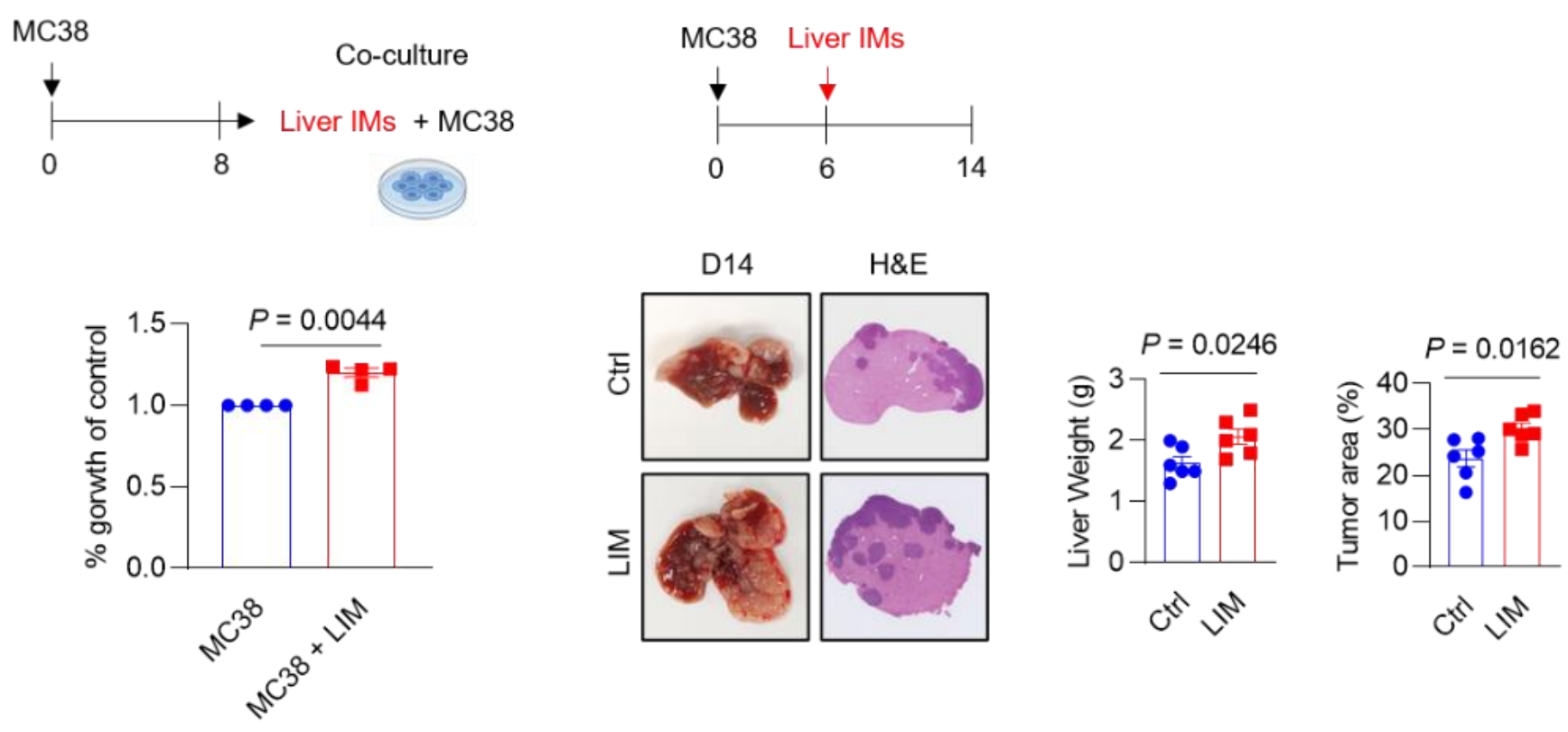

Figure 4 and 6:

To improve the flow of the manuscript, which is slightly hard to follow, I would suggest moving figure 6 after figure 4, and moving both 4 and 6 after figure 2. In figure 4, the authors suggest that glutamine consumption by tumor cells reduces local glutamine concentrations and therefore induces glutamine production by Ern1 KO but not Ern1 WT hepatocytes. In figure 6, authors show ectopic GS expression by Ern1 KO hepatocytes.

Response: We appreciate the reviewer's suggestion to prioritize the hepatocyte-focused sections. However, positioning Fig. 4 immediately after Fig. 2 would substantially weaken the logical development. The importance of CCR2$^+$ IMs is not assumed a priority but is instead established stepwise in Fig. 3, beginning with FACS-based immune composition analyses and followed by scRNA-seq-based identification and annotation of IMs. Skipping this progression would make it difficult to contextualize the monocyte-related findings in Fig. 4 and could introduce bias by moving directly from a glutamine-associated metastatic phenotype (Fig. 2) to hepatocyte–monocyte communications (Fig. 4) without first defining the relevant immune subset.

Similarly, Fig. 4 demonstrates that IRE1α-deficient hepatocytes release glutamine to support monocyte expansion, while Fig. 5 subsequently validates the functional importance of extracellular glutamine from the monocyte perspective. If the sequence were altered to jump directly from Fig. 4 to Fig. 6, which dissects the intracellular mechanism by which IRE1α-deficient hepatocytes generate excess glutamine, the logical continuity would be disrupted. We therefore believe the current figure order more appropriately conveys how meta-inflammation contributes to disease progression, moving from extracellular immune regulation (Fig. 4&5) to intracellular signal transduction (Fig. 6). Concluding with Fig. 6 also provides a coherent mechanistic endpoint, creating a closed narrative loop that begins and ends with hepatocyte-intrinsic signaling.

Figure 6c) how is GS expression regulated in vivo? Is FoxO1 only expressed in zone 3 hepatocytes?

Response: Additional immunohistochemistry analysis showed that FoxO1 protein was upregulated in tumor-adjacent hepatocytes of *Ern1^CKO* livers, but not in zone 3 hepatocytes (**Fig. 6d**), which explains excess GS expression in IRE1α-deficient hepatocytes.

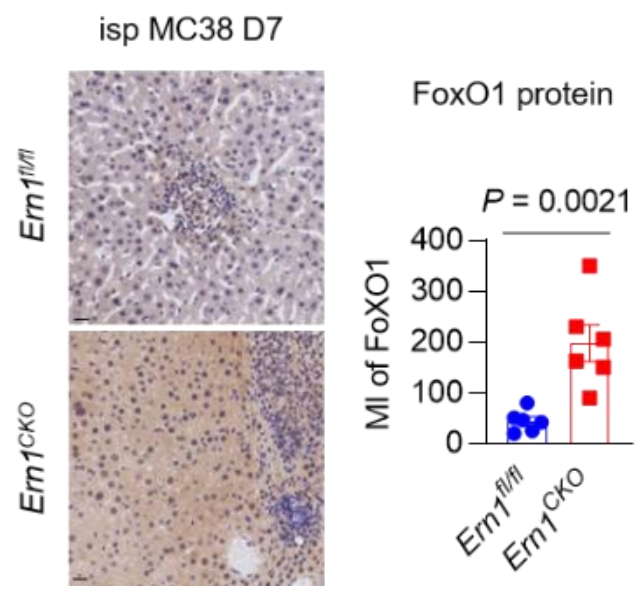

Figure 5:

e) The authors mention anti-inflammatory phenotype but only assess accumulation of CCR2+ cells.

Response: Throughout the study, CCR2 has been consistently used to define inflammatory (not anti-inflammatory) monocytes, as it is a well-established and widely accepted marker for this population (PMID: 21654748; 23081697). Based on the reviewer's concern, we have revised the wording from "an inflammatory phenotype" to "a CCR2$^+$ phenotype" (**Line 329**).

Here again, congenic markers should be used, while CTV or EdU can be used to differentiate accumulation vs. in situ proliferation.

Response: Based on reviewers' suggestions, we transferred congenic BMIMs to tumor-bearing *Ern1^fl/fl* and *Ern1^CKO* mice. The latter enabled better expansion of transferred cells, in a manner dependent on the glutamine transporter SLC38A2 (**Fig. 5e**).

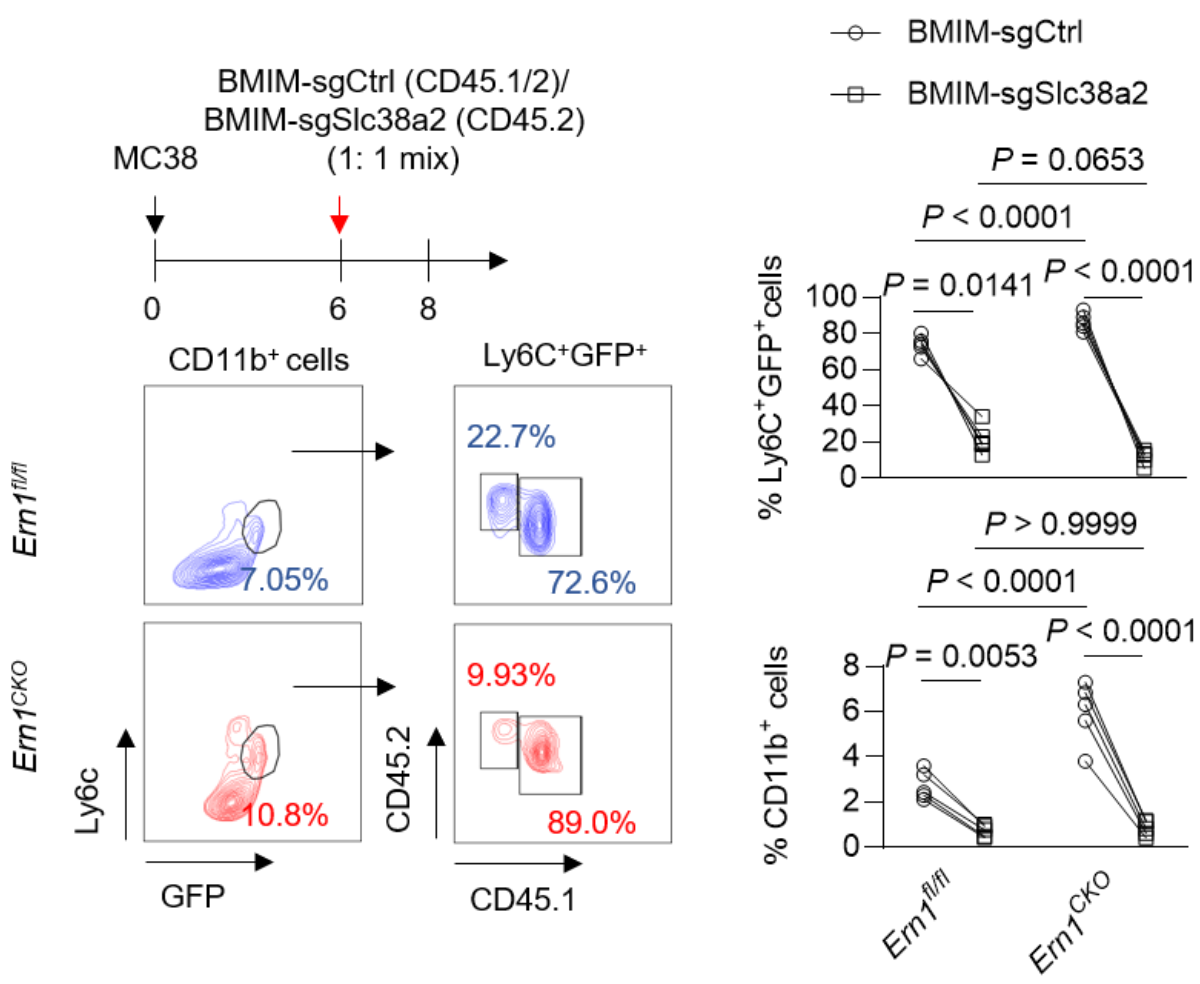

Extended Data Fig. 5e) Authors mention that Slc38A is only expressed by monocytes, but did they not

knock it out from MC38 cells as well?

Response: We apologize for the confusion and have revised the sentence to read: “Among the known glutamine transporters, SLC38A2 is preferentially expressed in both mouse and human monocytes” (**Line 332**).

Extended Data Fig. 5g) sgCtrl monocytes should also be transferred in both hosts for comparison.

Response: As suggested, we co-transferred sgCtrl-expressing CD45.1/2 and sgSlc38a2-expressing CD45.2 BMIMs for comparison (**Fig. 5e**).

Extended Data Fig. 5h) non-treated should also be shown for comparison.

Response: Non-treated groups have been included in revised **Extended Data Fig. 6h**.

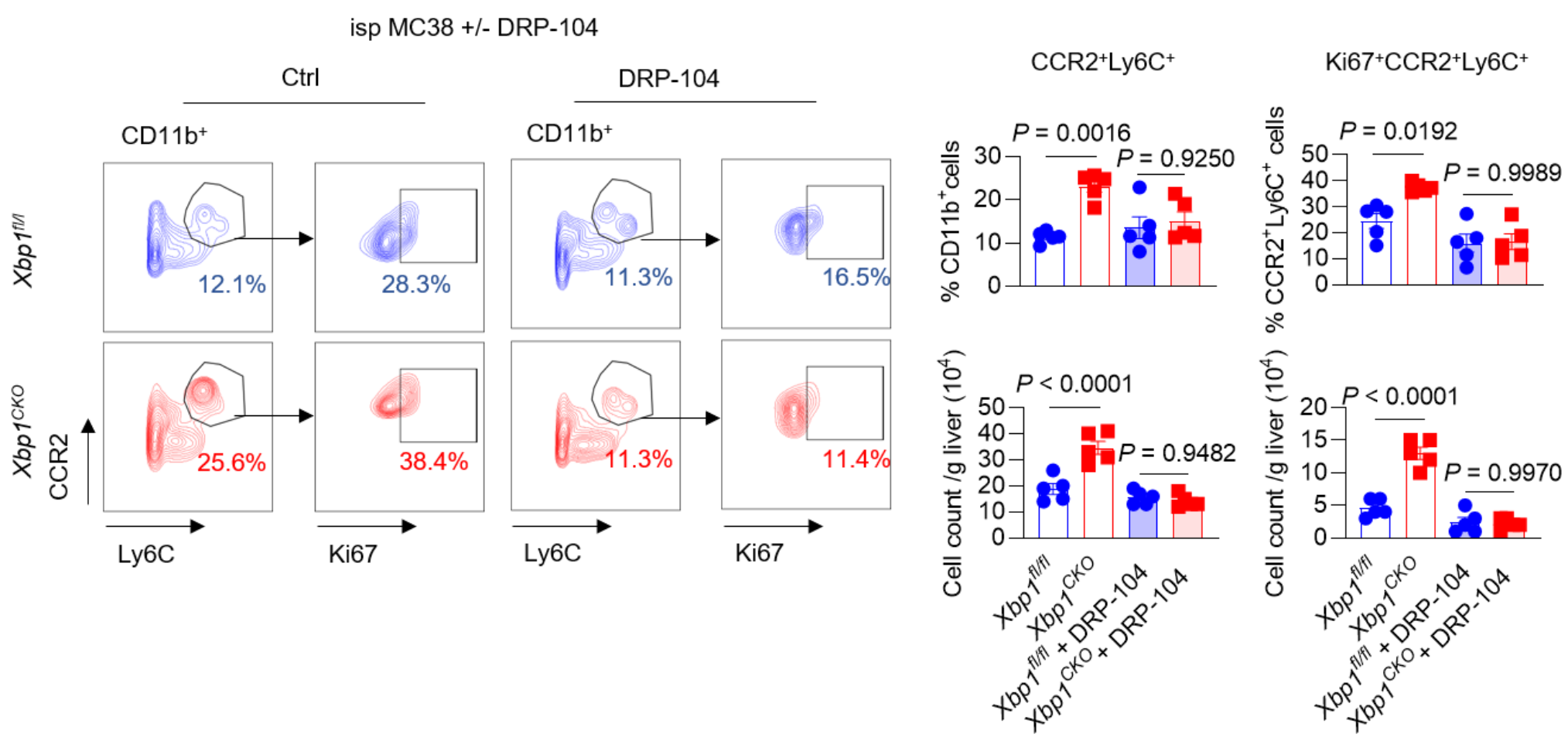

f) Authors suggest that extracellular glutamine supports expansion of monocytes in the liver. However, they previously show that glutamine levels are elevated in the portal vein of Ern1 KO mice. This suggests that there might be systemic effects. The authors should assess changes in myelopoiesis by looking at the bone marrow of Ern1 KO vs WT mice. The authors should clarify whether accumulation of monocytes in the metastatic liver is due to increased recruitment or in situ proliferation. Of note, the fact that CTV+ monocytes accumulate suggests that these do not proliferate in situ, otherwise CTV would be diluted.

Response: We measured glutamine concentrations in the portal vein because its low pressure allows portal blood metabolites to more closely reflect the composition of liver interstitial fluid. Given that the genetic manipulation and disease progression in our study are both liver-restricted, we consider this approach a direct and physiologically relevant way to assess changes in liver immunometabolism.

In response to the reviewer’s suggestion, we also examined bone marrow monocytes at day 7 of our

model and found no detectable differences between *Ern1*$^{fl/fl}$ and *Ern1*^{CKO} mice.

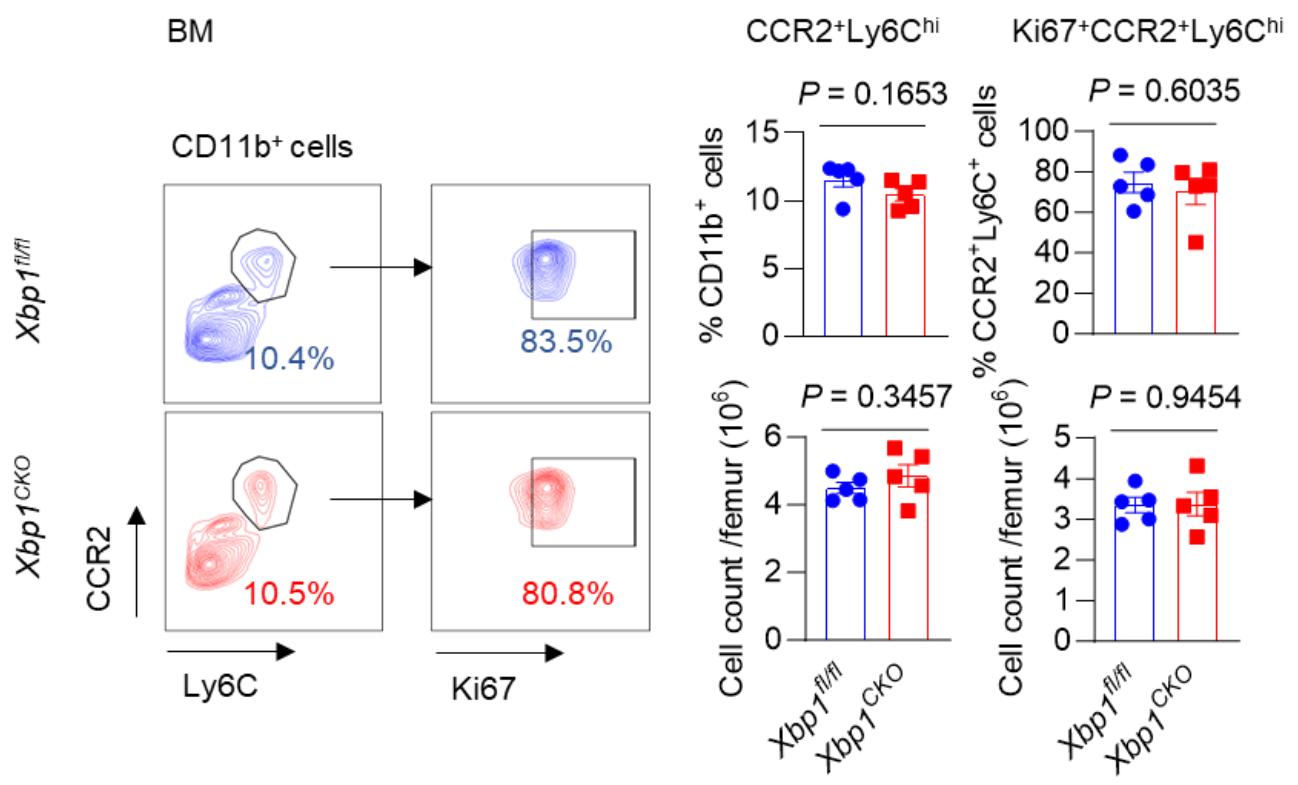

Figure 7:

a) and b) and Extended Fig 7a) The authors talk about hepatocyte IM-communication, however their data show increased IM infiltration in the tumor, which suggests IM-tumor communication. There is no data supporting the notion that hepatocytes and IM directly communicate, only that, in response to tumor-induced glutamine starvation, Ern1 KO hepatocytes produce glutamine that induces accumulation of IM.

Response: This study highlights hepatocyte–monocyte communication as an underappreciated mechanism in liver metastasis, without excluding or contradicting the established role of tumor–monocyte communication at later stages. As shown in Fig. 3 and Extended Data Fig. 5, IM accumulation in *Ern1*^{CKO} livers emerges around day 7, a time point that coincides with metastatic progression and permits monocyte infiltration into tumor sites. Consistently, monocytes were detected in both intratumoral and peritumoral regions (**Fig. 3f**, **Fig. 7a** and **Extended Data Fig. 8a**), supporting the coexistence of tumor–monocyte and hepatocyte–monocyte communications during metastatic outgrowth.

Minor points:

- the amount of tumor infiltrating Ly6C monocytes should be quantified.

Response: As suggested, quantification of immunofluorescence data on metastasis-bearing liver sections has been shown in **Extended Data Fig. 7c–f**, including Ly6C^{+} cell counts per HPF across different liver zones.

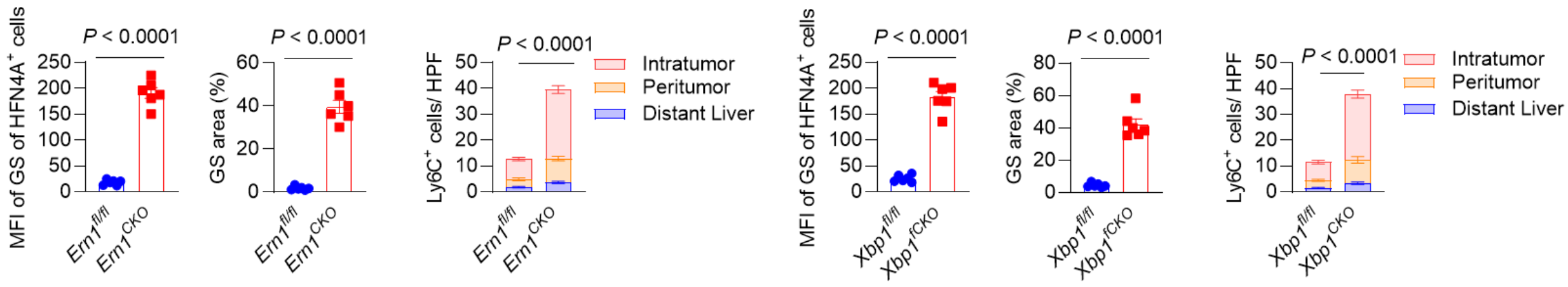

Extended Data Fig 7b) the authors state that Xbp1 expression in patients is heterogenous, however from the images show it does not look like it. Is this heterogeneity also observed in WT mice? What is the baseline expression pattern of Xbp1 and IRE1 in non-tumor bearing human/mice liver? Does it correlate with GS expression or liver zonation? How do the authors explain the suggested heterogeneity in expression? Is there a link to ER stress, or to local tumor cell proliferation?

Response: We apologize for the confusion and have revised the sentence to read: "XBP1s protein expression in hepatocytes was heterogeneous across patients but appeared modestly elevated and more uniformly distributed within tumor regions" (**Line 416**). We would like to clarify that the observed heterogeneity reflects inter-individual variability rather than variability within a single immunohistochemical field.

In contrast, XBP1s expression was undetectable in healthy, tumor-free mouse livers.

- Can the authors stratify patients in microsatellite stable vs unstable? MC38 cells are a model for microsatellite unstable tumors.

Response: All colorectal cancer specimens included in this study were microsatellite stable and were obtained from patients who underwent standardized neoadjuvant chemotherapy followed by radical surgical resection. We acknowledge that MC38 is a microsatellite unstable and relatively immunogenic cell line. However, we do not believe that the mechanisms identified here are dependent on the immunogenicity of MC38. Rather, our study focuses on non-tumor cell-autonomous regulation within the liver metastatic niche. Supporting this notion, liver-specific resistance to MKC8866 was also observed in the poorly immunogenic 4T1 spontaneous metastasis model (**Extended Data Fig. 1c, d**).

- The authors mention in the discussion that monocytes are known to instruct the pre-metastatic niche, however in this manuscript they only use intrasplenic injection, no primary tumor model. This should be either tested (for example by transferring monocytes from tumor-bearing mice), or stated in the discussion as a limitation of the study.

Response: Intrasplenic injection-based liver metastasis proceeds through the physiologically relevant portal vein route and has been extensively validated in landmark studies for investigating the

immunobiology of liver metastases derived from gastrointestinal cancers and melanoma (PMID: 29443964, 41203813, 23081697, 30842658, 41690948, 39048831), including the seminal work that first established the fundamental role of $CCR2^+$ IMs. Although this approach does not recapitulate the initial intravasation stage, it provides a unique and well-controlled experimental window to interrogate intercellular communication within the liver.

In particular, our data indicate that hepatocyte-instructed monocytes act predominantly during metastatic outgrowth within the liver, a stage that is mechanistically distinct from the initial intravasation processes occurring at the primary tumor site. Therefore, we consider that inclusion of a primary tumor model would be unlikely to add substantial mechanistic insight.

Reviewer #4 (Remarks to the Author):

The manuscript by Zhang et al. investigates the role of the hepatocyte IRE1α–XBP1s axis in liver metastasis and reveals an unexpected anti-tumorigenic function. Using genetic hepatocyte-specific knockouts (Ern1CKO and Xbp1CKO) and pharmacologic inhibitors, the authors show that this pathway suppresses metastatic outgrowth by restricting glutamine synthesis and release. Loss of hepatocyte IRE1α–XBP1s activates a non-canonical FoxO1–glutamine synthetase (GS) pathway, leading to increased hepatocyte-derived glutamine, which promotes inflammatory monocyte (IM) survival and proliferation. These IMs, in turn, create a pro-metastatic microenvironment that enhances cancer cell growth. The study presents a novel and conceptually important mechanism suggesting that hepatocyte IRE1α–XBP1s exerts tumor-suppressive effects by limiting glutamine-driven inflammatory monocyte expansion and pro-metastatic niche formation. However, several key mechanisms remain unresolved, including:

- How inflammatory monocytes (typically anti-tumor) promote metastasis in this context, and whether macrophage differentiation and reprogramming is involved.
- Direct mechanistic proof of the FoxO1–GS axis.
- Clarification if monocyte recruitment rather than proliferation is necessary.

These issues, along with missing methodological and quantification details, justify a recommendation of major revision.

Response: We thank the reviewer for the appreciation of our work and the suggestions for improving

it.

Major Comments

1. Monocytes versus macrophages: missing mechanistic explanation

The manuscript attributes tumor promotion to increased inflammatory monocytes, but it remains unclear how pro-inflammatory monocytes promote tumor growth, and how this is distinguished from monocyte-derived (M2) macrophages, which are classical mediators of metastatic niche formation.

Response: The role of CCR2$^+$ monocytes in regulating liver metastasis has been well established in prior work (PMID: 23081697). As anticipated, liver-derived CCR2$^+$ IMs enhanced MC38 proliferation in an ex vivo co-culture assay and promoted metastatic progression in an adoptive transfer model (**Extended Data Fig. 5c, d**).

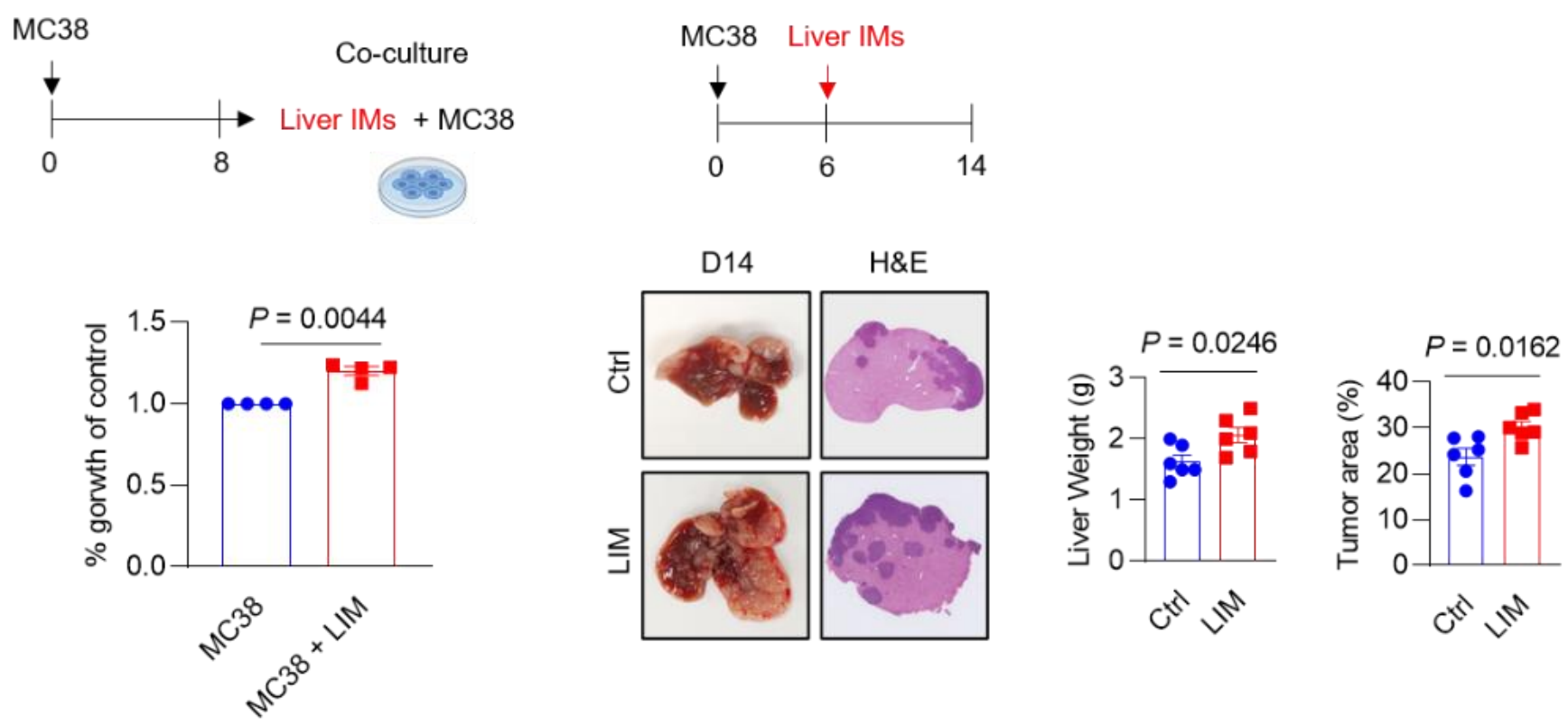

As shown in **Extended Data Fig. 3b**, flow cytometry analyses revealed no differences in the percentages of M2 macrophages between *Ern1*$^{fl/fl}$ and *Ern1*^{CKO} livers. As downstream effector immune populations were not required to account for the differential metastatic burden observed between the two groups (**Extended Data Fig. 3d, e**), immunosuppressive cells such as M2 macrophages are unlikely to be the primary drivers of the phenotype described in this study.

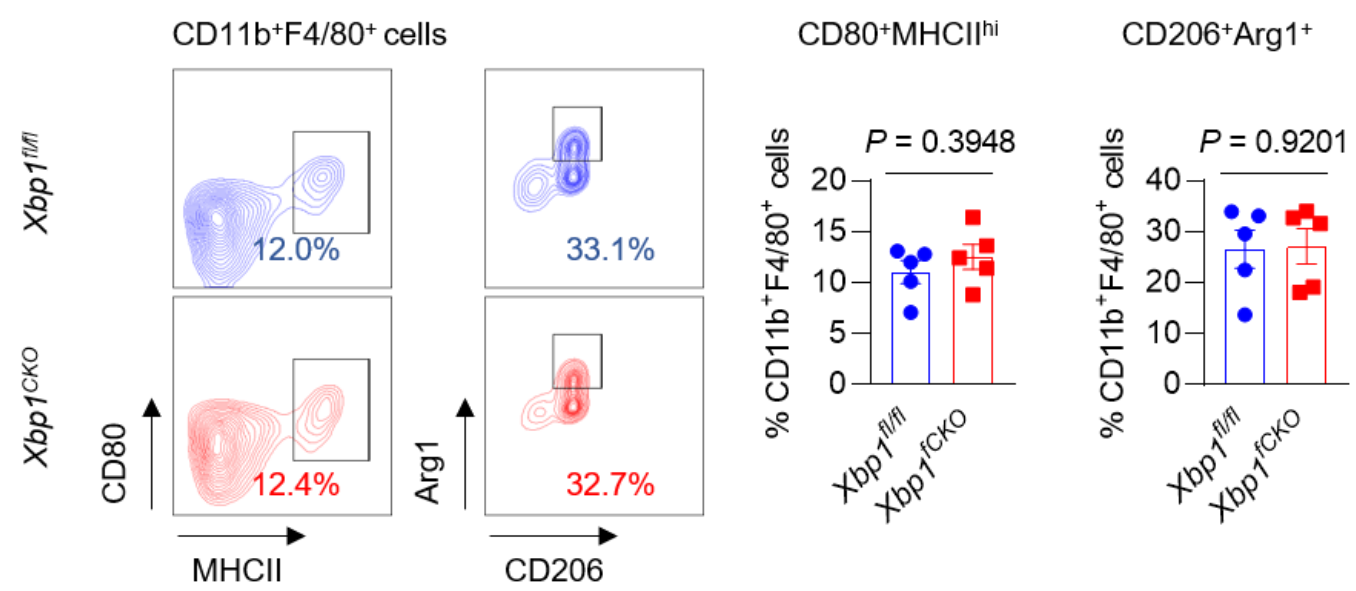

- The anti-Ly6C antibody (Extended Fig. 2c) and CCR2 inhibitor (Fig. 3g) experiments prevent monocytes and consecutevly prevent differentiation of macrophages. Therefore, a destinction can not

be made basted on those experiments.

Response: We acknowledge that anti-Ly6C antibody treatment may not only block monocyte differentiation but also deplete a subset of Ly6C-expressing macrophages. Similarly, CCR2 inhibition could potentially affect additional immune populations (**Fig. 3c, d**). However, as explained in the manuscript (**Line 213–217**), hepatocyte IRE1α depletion selectively altered the abundance of monocytes, whereas other immune cell populations remained unchanged. Therefore, despite their known breadth, both Ly6C- and CCR2-based depletion strategies are appropriate and predominantly effective for eliminating the differential monocyte frequencies observed between *Ern1*$^{fl/fl}$ and *Ern1*^{CKO} livers.

- The authors should clarify the mechanistic link between glutamine, IM accumulation, and tumor promotion. If the monocytes are indeed pro-inflammatory, what specific cytokines, factors, or interactions make them pro-tumorigenic?

Response: Comprehensive in vivo and in vitro experiments demonstrated that glutamine directly supports the expansion of IMs (**Fig. 4a, 5a–d**). Consistently, their expansion was markedly attenuated upon ablation of the glutamine transporter SLC38A2 (**Extended Data Fig. 6f and Fig. 5e**).

Our ex vivo single-cell RNA-seq and in vitro bulk RNA-seq analyses confirmed that IMs exhibit a pro-inflammatory transcriptional program (**Extended Data Fig. 4d and Fig. 5c**). Many of the upregulated pro-inflammatory mediators (*C1qa*, *C1qb*, *C1qc*, *Mif*, *Ccl2*, *Siglec1*, *Ccl8, etc*) have been implicated in promoting tumor progression. Consistently, liver-derived CCR2^{+} IMs enhanced MC38 proliferation in an ex vivo co-culture assay and promoted metastatic progression in an adoptive transfer model (**Extended Data Fig. 5c, d**).

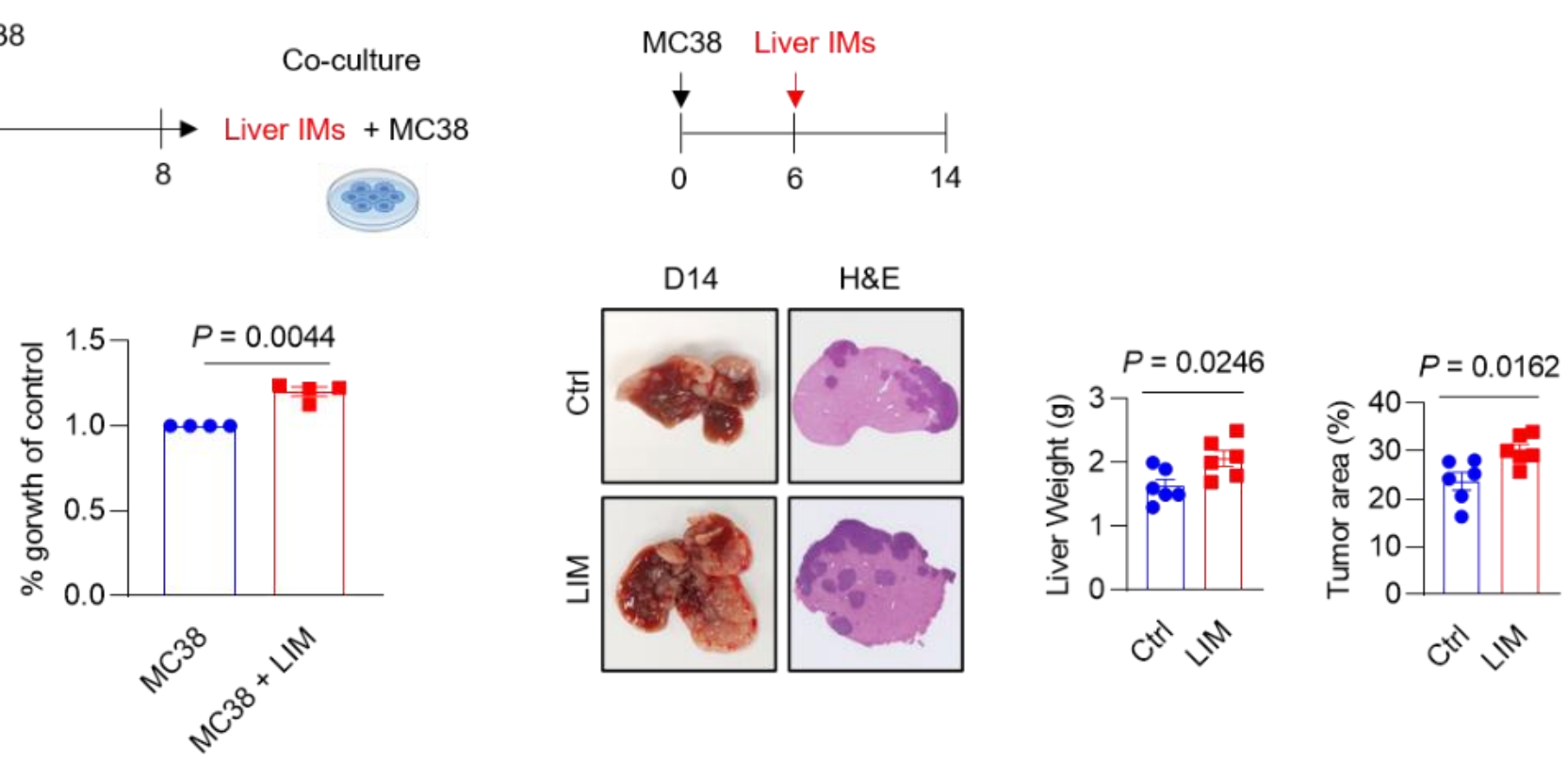

Our data indicated that the primary difference between *Ern1*$^{fl/fl}$ and *Ern1*^{CKO} livers lies in the expansion of IMs, rather than in per-cell expression levels of pro-inflammatory genes (**Lines 244–246**). As a

functionally defined immune subset, IMs exert their cancer-promoting effects through the coordinated action of multiple mediators rather than a single molecule; therefore, we consider that specification of individual effector genes is not required for the scope of this study.

- The single-cell RNA-seq data could be leveraged to examine macrophage polarization (e.g. CD206, Mrc1, Arg1, CD80, CD86, MHCII, CD163).

Response: As suggested, macrophage-related markers are presented in the following bubble chart encompassing the relevant clusters, revealing a similarity between *Ern1*$^{fl/fl}$ and *Ern1*^{CKO} livers.

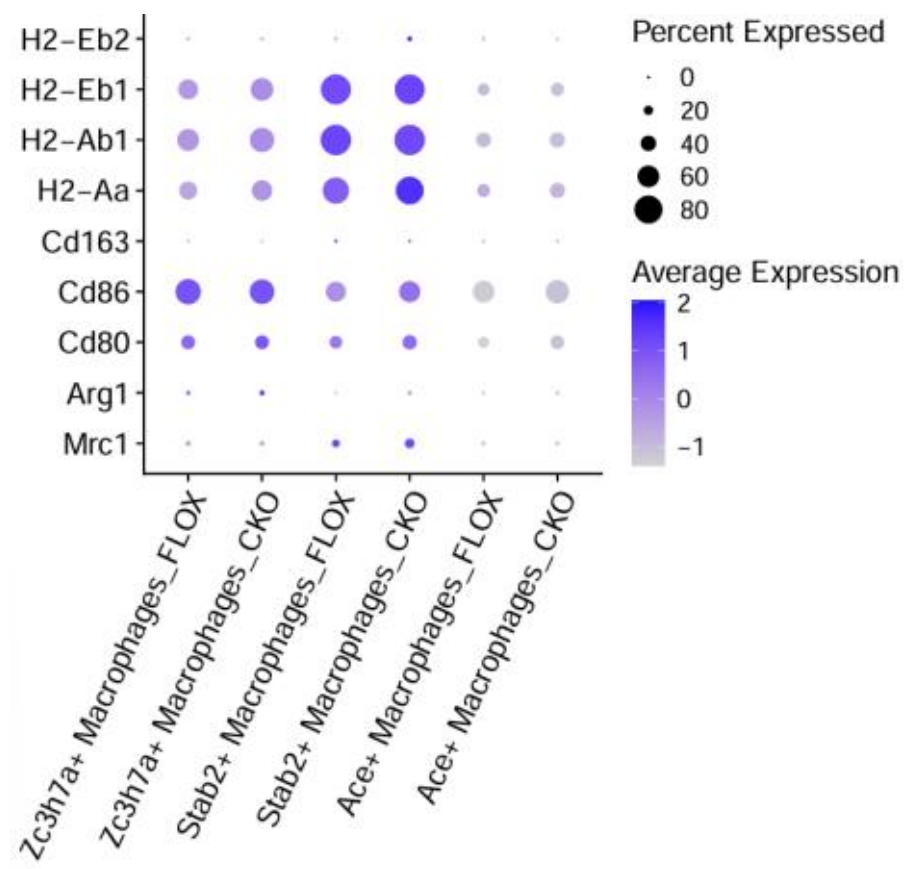

- Additional flow cytometry using these markers should be included to verify macrophage abundance and polarization (M1 vs M2).

Response: As shown in **Extended Data Fig. 3b** and below, flow cytometry analyses revealed no differences in the percentages of M2 macrophages between *Ern1*$^{fl/fl}$ and *Ern1*^{CKO} livers.

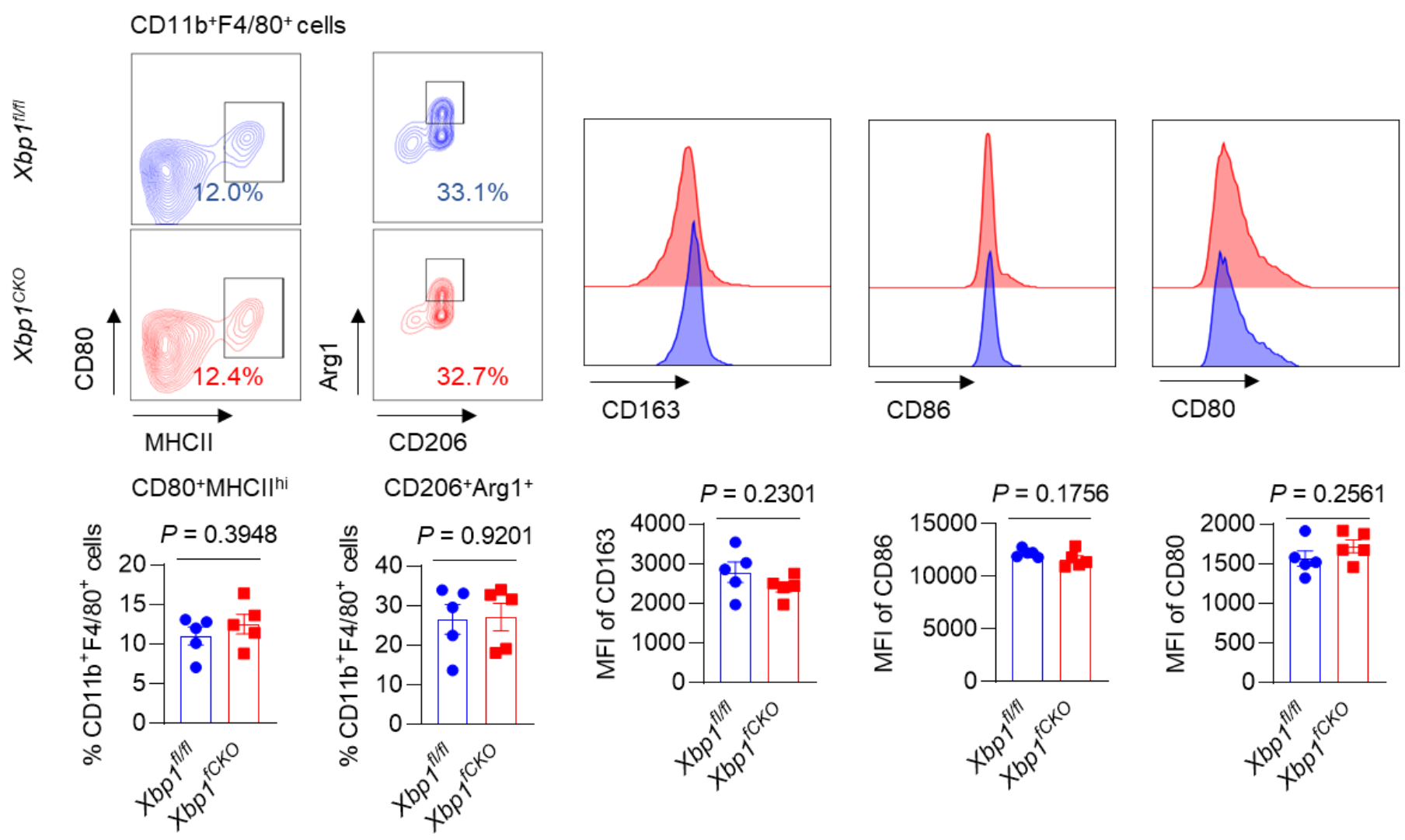

- Given that glutamine metabolism is known to drive M2-like macrophage polarization, macrophage reprogramming may also contribute to the observed pro-metastatic phenotype and should be thorougly investigated.

Response: Although glutamine has been reported to promote M2-like macrophage polarization (PMID: 25786174) and dendritic cell activation (PMID: 37407815), we did not observe increased frequencies of these immune populations in *Ern1*^{CKO} livers. In our system, extracellular glutamine becomes excessive in *Ern1*^{CKO} livers at a stage that coincides with monocyte-mediated metastatic progression. This temporal alignment may explain why the predominant glutamine-responsive phenotype was observed in inflammatory monocytes.

2. Monocyte recruitment versus local proliferation

The differentiation between recruitment and local proliferation of IMs in IRE1α/XBP1-hepatocyte deficient livers remains ambiguous. Fig. 4c shows that glutamine effects are lost in CCR2-/- mice, suggesting recruitment is required.

- Fig. 3g demonstrates that CCR2 inhibition (RS102895) abolishes the metastatic increase in Ern1CKO mice, further implying dependency on recruitment.

- The adoptive transfer experiment (Extended Fig. 4f) is showing elevated monocyte numbers, but this could equally reflect increased recruitment rather than proliferation. If most cells within the livers were really derived from proliferation, this could be shown by the decrease of CTV in those newly devided cells.

- Real proliferation should also be shown by CTV assay not indirectly by Ki67 in all in-vitro experiments.

Response: We acknowledge that monocyte recruitment is a prerequisite for the observed hepatic phenotypes. However, this does not necessarily imply that monocyte recruitment itself is directly regulated by disturbed intrahepatic signaling. Importantly, this interpretation does not contradict our central conclusion that intrahepatic metabolic cues—rather than altered recruitment per se—constitute the dominant force driving monocyte accumulation in the *Ern1*^{CKO} liver.

Moreover, because inflammatory monocytes are not generally considered rapidly dividing cells, CTV-based division assays may not reliably capture their proliferative behavior, even though these cells can undergo proliferation both in vitro and in vivo.

- Investigate whether IRE1α/XBP1-deficient hepatocytes secrete higher levels of CCL2 or other chemokines responsible for increased monocyte recruitment.

Response: To address whether monocyte recruitment was substantially affected, we detected CCL2 levels in the peripheral blood. However, serum CCL2 levels were comparable between liver metastasis-bearing *Ern1*$^{fl/fl}$ and *Ern1*^{CKO} mice, suggesting that IRE1α/XBP1-deficient hepatocytes are

unlikely to differentially regulate CCL2 production in a manner sufficient to alter monocyte recruitment.

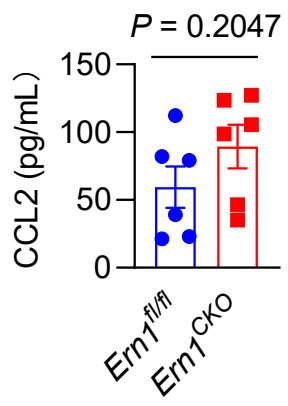

- Extended Fig. 4e: Since MKC8866 treatment increases IMs, also test MKC8866 in CCR2-/- mice to proof resistance to MKC8866 in the liver is really monocyte-depndent (effect should be restored in this setup, as the direct tumor effect remains).

Response: We appreciate the reviewer’s suggestion and have confirmed that the combinatorial use of the CCR2 inhibitor RS102895 can resensitize liver metastases to MKC8866 treatment (**Fig. 3h**).

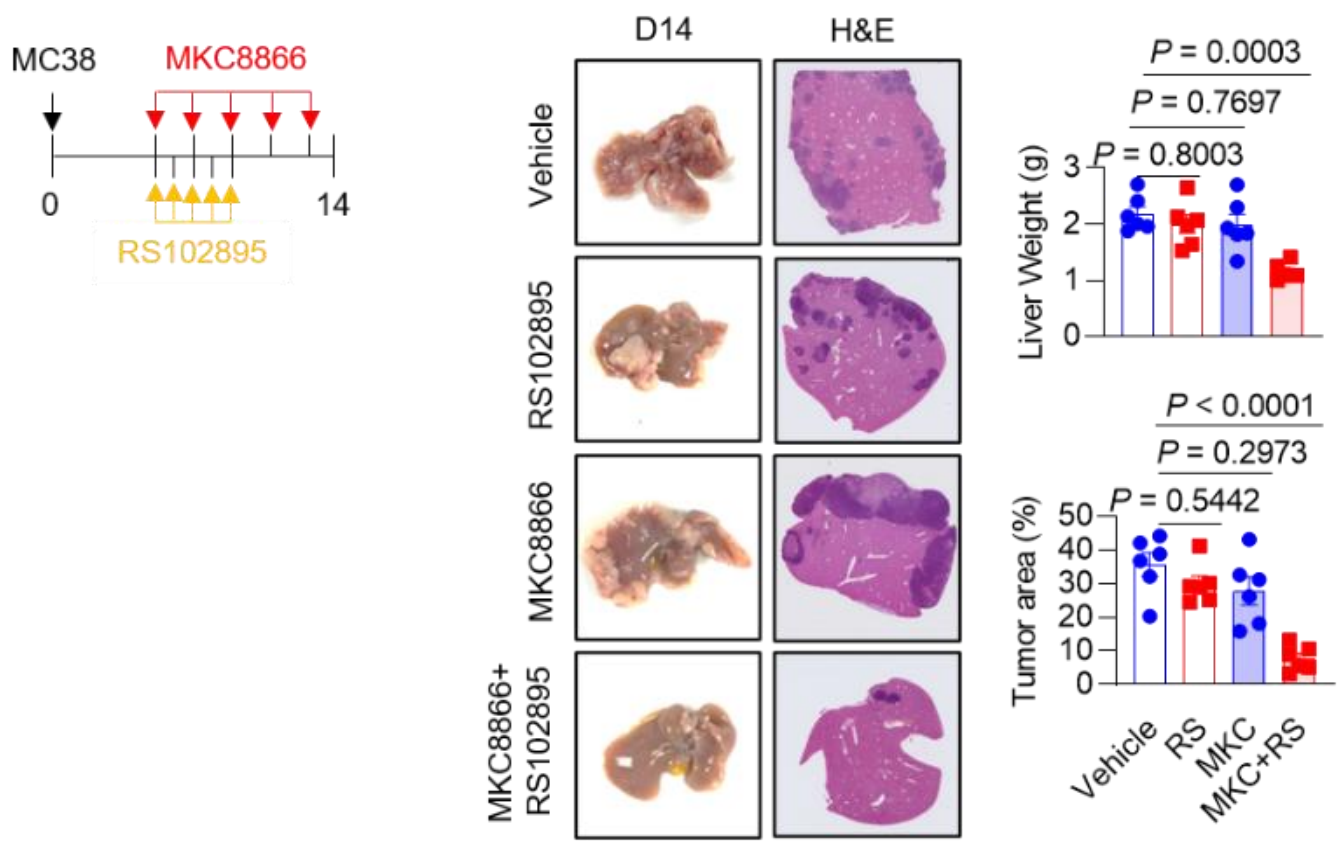

Given loss of effect in CCR-/- mice and with CCR2 inhibition, together with the common concept that mature monocytes outside the bone marrow rarely proliferate, a proliferation-based hypothesis needs more substantial proof.

Response: Monocyte recruitment functions as a prerequisite for any monocyte-related phenotypes observed in the liver. However, this does not necessarily imply that monocyte recruitment itself is directly regulated by disturbed intrahepatic signaling.

Inflammatory monocytes are considered to be proliferative in the context of inflammation (PMID: 17082649, PMID: 23695680).

3. Mechanistic link between IRE1α–XBP1s and FoxO1–GS signalling

The connection between IRE1α–XBP1s loss and FoxO1–GS activation is not directly shown.

- Fig. 6c demonstrates that FoxO1 inhibition reduces GS and glutamine in vitro, but it does not establish that FoxO1 mediates GS induction in Ern1CKO hepatocytes.

- → Show that FoxO1 inhibition restores GS expression and glutamine secretion in Ern1CKO

hepatocytes to WT levels.

Response: We appreciate the reviewer's suggestion and have confirmed that FoxO1 inhibition mitigates GS expression and glutamine secretion in *Ern1*^{CKO} hepatocytes (**Fig. 6e, f**).

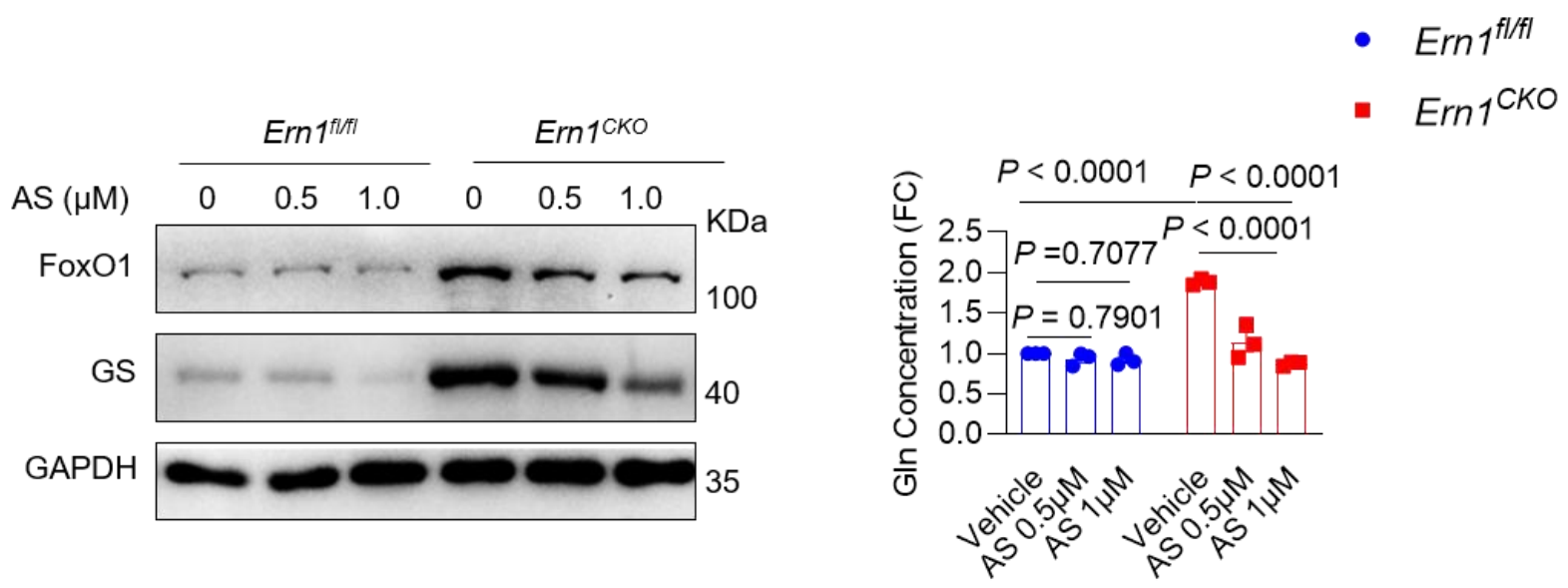

- → In vivo, validate that FoxO1 intensity correlates in histology with GS expression in peritumoral hepatocytes (Fig. 6a).

Response: As shown in revised **Fig. 6d**, FoxO1 protein was predominantly localized to tumor-adjacent hepatocytes in *Ern1*^{CKO} livers, consistent with the newly acquired GS expression pattern.

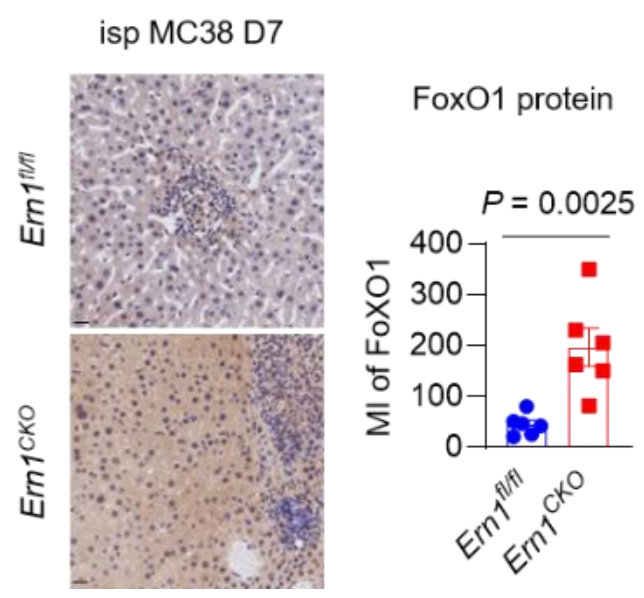

4. Validation of the tumor-suppressive hepatocyte model

To support the claim that hepatocyte IRE1α–XBP1s signaling is tumor-suppressive and causes resistance to MKC8866 in the liver, the authors should directly test MKC8866 treatment in Ern1CKO and Xbp1CKO mice.

- Include four experimental groups per model (WT ± inhibitor, CKO ± inhibitor).

- If IRE1α–XBP1s protection is lost in knockout hepatocytes, MKC8866 should once again reduce tumor growth by acting directly on tumor cells.

- This is essential to confirm the interpretation of Fig. 1c–f and to support the claim that the MKC8866 inefficacy in liver metastases is due to loss of hepatocyte protection.

Response: We appreciate the reviewer's suggestion and have confirmed that MKC8866 regained therapeutic efficacy against liver metastases in *Xbp1*^{CKO} mice (**Extended Data Fig. 1f**).

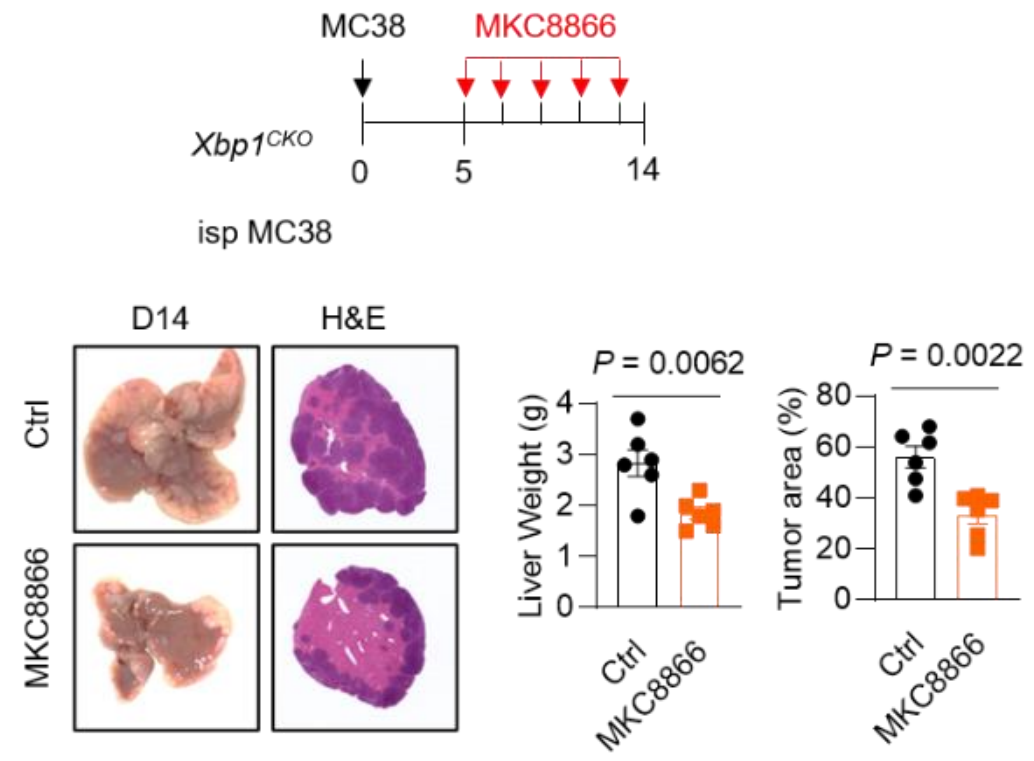

5. Consistency between models

The manuscript alternates between Ern1CKO and Xbp1CKO models.

→ Use one consistently in main figures and include confirmatory results from the other model in the Supplementary Figures.

This would improve narrative flow and clarity.

Response: We appreciate the reviewer's suggestion and have accordingly reorganized the figure layout to improve clarity.

Minor Comments

6. Metabolomics (Fig. 2c) – report all metabolites and rationale

- The metabolomics analysis focuses solely on glutamine, but the Supplementary Table with complete data was not provided (to the reviewer).

Response: We have provided Supplementary Tables along with the revised manuscript.

- Justify the specific focus on glutamine over other altered metabolites.

7. Experimental controls and missing methodological details

- Fig. 4f: Include WT ± glutamine control groups to confirm that glutamine supplementation was effective in this setup.

Response: The WT ± glutamine condition was originally presented in **Extended Data Fig. 2j**. To facilitate direct comparison within the same experimental setting, we have now included a repeated WT ± glutamine cohort as a control in **Fig. 4c** (We note that the reviewer may have inadvertently referred to Fig. 4c as Fig. 4f).

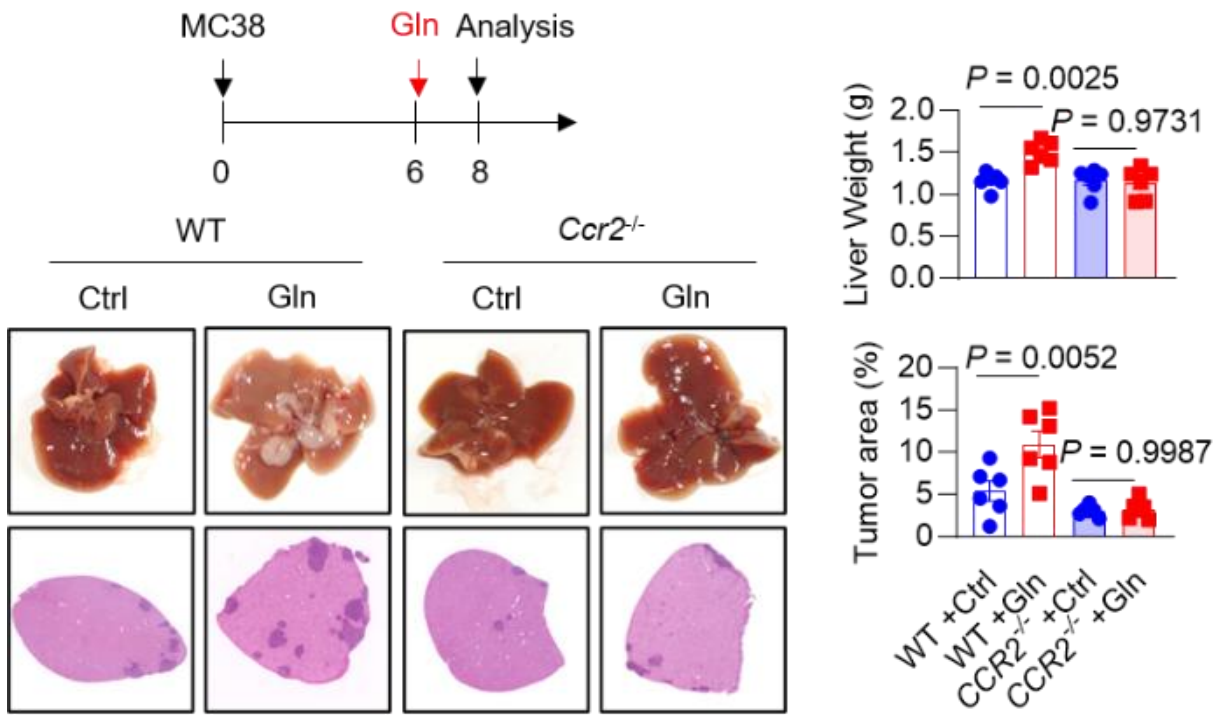

- Hepatocyte-conditioned medium: Culture conditions, baseline glutamine levels, and glutamine concentrations during co-culture with BMIMs are insufficiently described.

- → Include media controls with equivalent glutamine concentrations (compareble to the difference in hepatocyte-conditioned medium) ± L-asparaginase to confirm that observed effects are glutamine-specific.

Response: We appreciate the reviewer's concern. Information regarding hepatocyte culture conditions has been provided in the Methods section. The baseline glutamine concentration in the culture medium was 0.6 mM, which approximates a physiological glutamine level and was also used as the starting condition for BMIM cultures prior to supplementation with conditioned media (**Extended Data Fig. 6b**). In response to the reviewer's suggestion, we have included a hepatocyte medium-control condition containing 0.6 mM glutamine. Under this condition, the treatment with L-asparaginase also suppressed BMIM expansion, though the baseline level was relatively low.

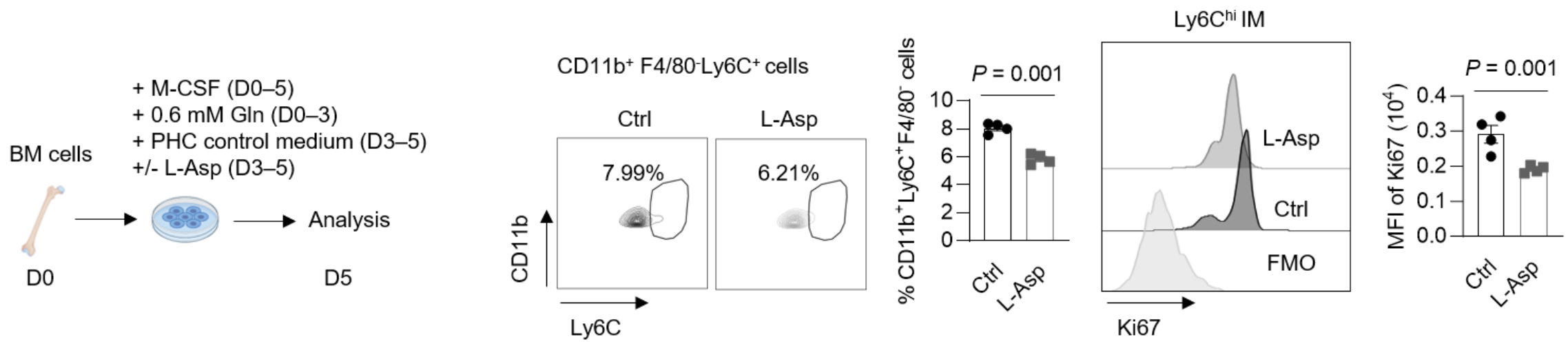

Moreover, we corroborate that glutamine directly impacts monocyte function by exposing BMIMs to different concentrations of glutamine (**Fig. 5a, b**). Whereas glutamine at concentrations comparable to those in conditioned media was sufficient to induce BMIMs, glutamine restriction markedly suppressed monocyte expansion. We did not include L-asparaginase in this setting as a dose-response design more cleanly isolates glutamine-specific effects.

8. Fig. 3f: Quantify CCR2⁺ IMs both within tumor lesions and in surrounding tissue to confirm local enrichment.

Response: As suggested, quantification has been carried out to present IM abundance in both tumor lesions and the surrounding tissue (**Fig. 3f**).

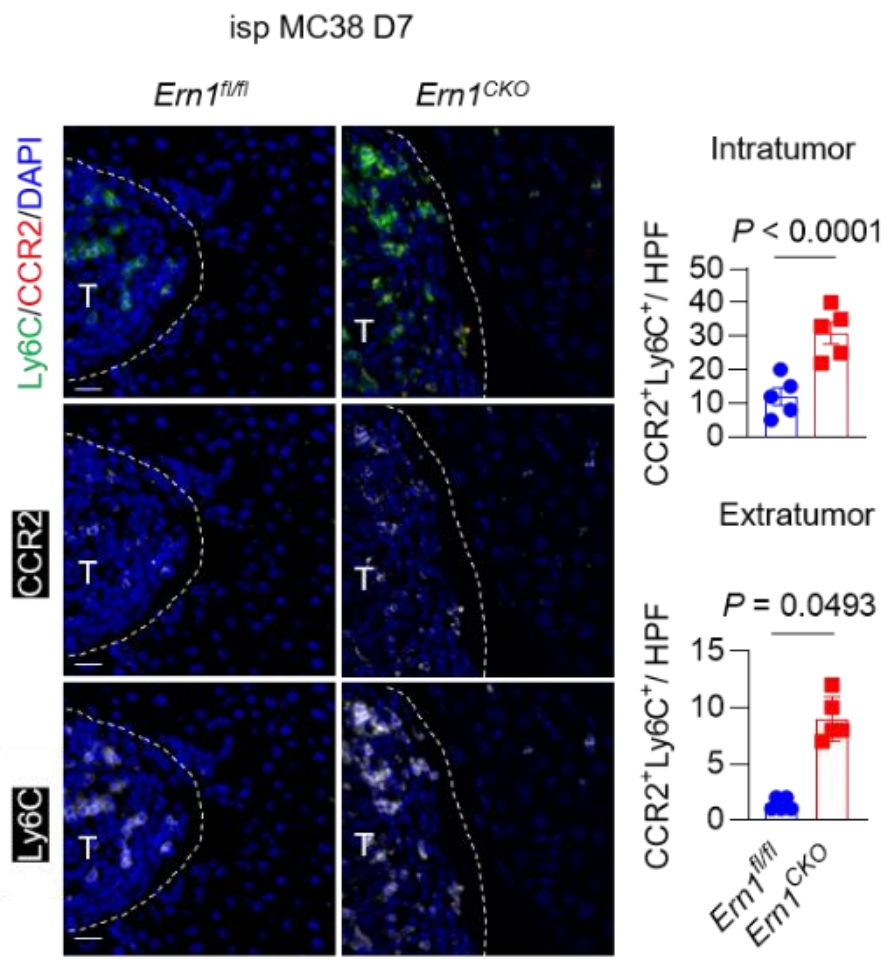

9. Blood and spleen: Include IM frequencies to determine whether changes are local or systemic.

Response: We have added this analysis and found the frequency of circulating IMs was modestly increased in *Xbp1*^{CKO} mice. However, the absolute number of circulating IMs remained comparable between the two groups.

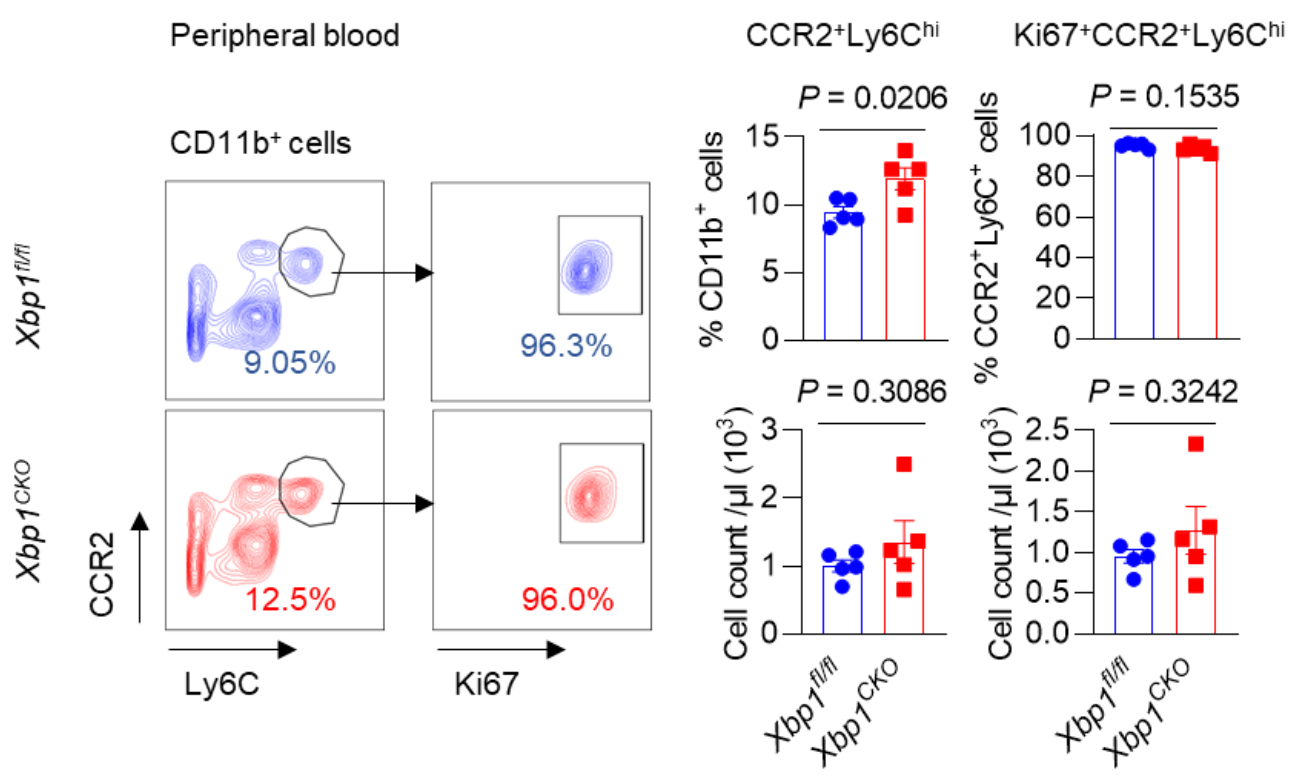

10. Fig. 6c: Provide full Western blot images, importantly quantifications, and potentially reduced exposure times to enhance band distinction.

Response: We have repeated the experiment using a shorter Western blot exposure and have replaced the GS and FoxO1 bands accordingly in **Fig. 6c**. We have also provided densitometric quantification accordingly. Uncropped Western blot images are available from our supplementary data.

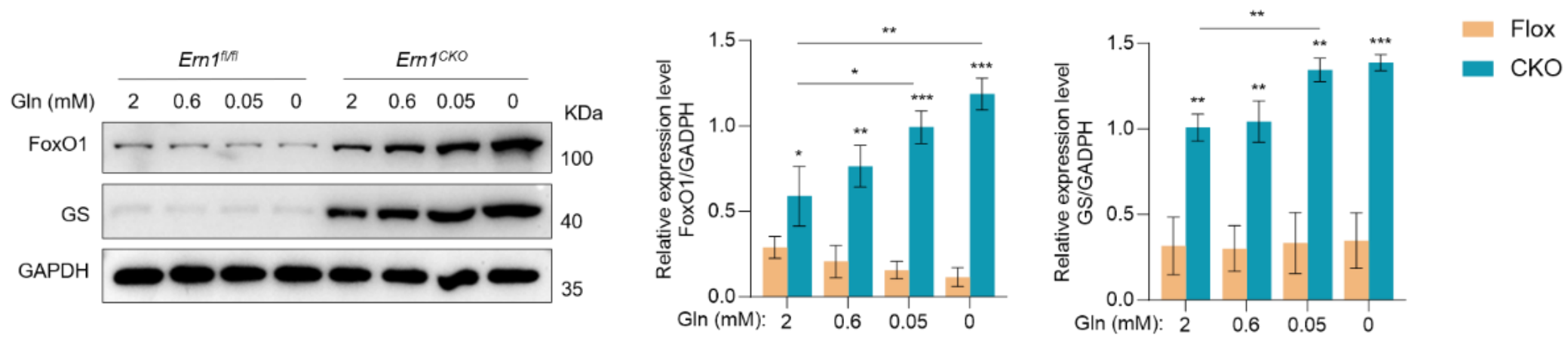

11. Fig. 4f: Monocytes are expressed as % of CD45⁺ cells—could this reflect a differentiation defect rather than proliferation? Include cell counts, show macrophages, increase sample size for statistical robustness (there is a clear trend in the L-Asp treated groups as well).

Response: The left plot graph in **Fig. 4f** depicts CD11b⁺Ly6C⁺F4/80⁻ cells (indicated in the y-axis of the right bar graph) rather than the entire CD11b⁺ population, thereby excluding the macrophage fraction and minimizing analytical bias. Within this undifferentiated CD11b⁺Ly6C⁺F4/80⁻ compartment, the proportion of Ly6C^{hi} cells serves as an indicator of IM turnover. To improve clarity, we have described this gating strategy in the figure legend.

To further address the reviewer's concern, we collected both the loosely adherent and firmly adherent cell fractions from the BMIM culture system for additional analyses. As shown below, the percentage of F4/80⁺ macrophages within the CD11b⁺ population remained unchanged across different culture conditions, consistent with their relevant stable proliferation (**Extended Data Fig. 6c, d**).

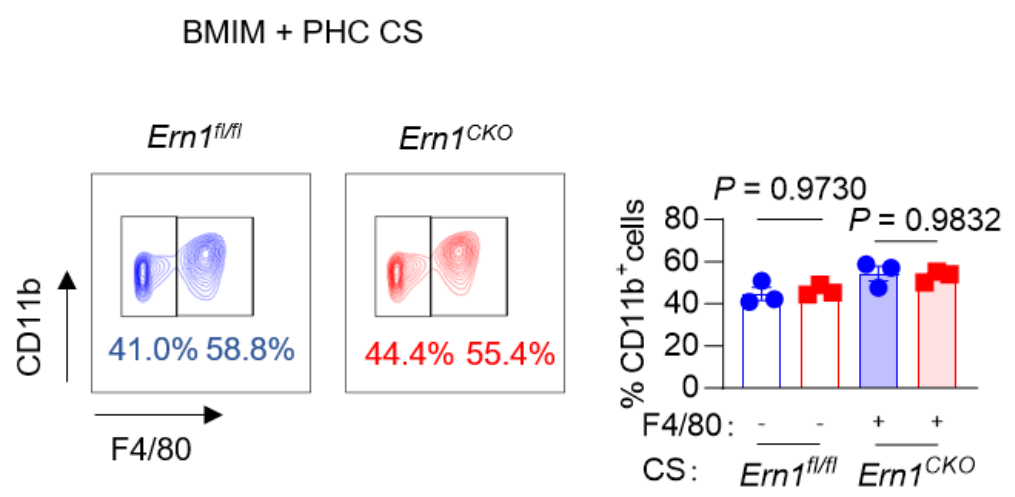

12. Extended Fig. 4f: Confirm whether data are expressed as % of CD45⁺ or CD11b⁺ cells, as CD11b⁺ would better match the representative plots in Extended Fig. 4b.

Response: We appreciate the reviewer's scrutiny and have now corrected the y-axis labeling (now shown in revised **Extended Data Fig. 5i**).

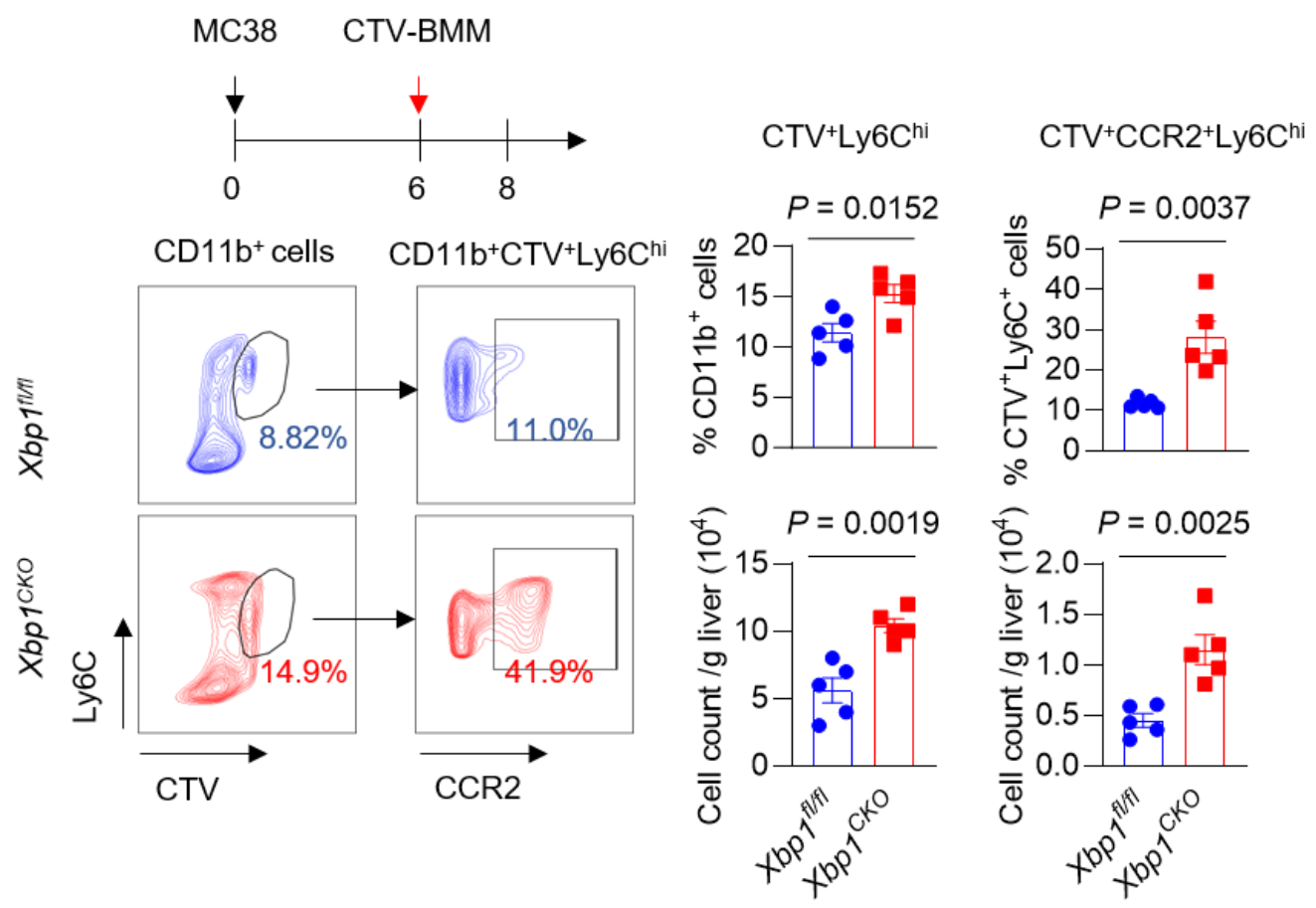

13. Clinical trial rationale:

- The rationale that IRE1α inhibitors (e.g., ORIN1001 / MKC8866) are ineffective in metastasis is insufficiently supported.

- NCT03950570: published only as a safety abstract with limited efficacy data.

- NCT04834778: investigates HC-5404, a PERK inhibitor, not an IRE1α inhibitor.

- ◊ Please provide accurate references or clarify that no clinical data on anti-metastatic efficacy are yet available.

Response: We apologize for the incorrect citation of NCT04834778 and have now removed it from the manuscript. In addition, we have updated the Discussion to clarify that no clinical data on anti-metastatic efficacy are currently available (**Line 492–494**).

14. Timing of glutamine supplementation (Fig. 2e–f)

- If glutamine is crucial for IMs, and IM frequency peaks at day 2 (Extended Fig. 4b), why is supplementation at day 1 ineffective?

- Harmonize time points between Fig. 2e and Fig. 2f (e.g., day 5 and 7, as used in Fig. 2a). Currently, the timely distance between early Gln and sacrefice is 11 days and for late Gln only 2 days.

- → Discuss whether monocyte glutamine dependence changes dynamically during metastatic niche formation.

Response: The absence of an effect indicates that extra glutamine does not functionally promote metastatic seeding, potentially due to the glutamine-saturated microenvironment at this early stage. In contrast, when glutamine became relatively limiting at an intermediate stage (day 6), exogenous glutamine supplementation was able to facilitate liver metastatic progression.

We selected day 1 and day 6 as the time points for glutamine supplementation, and day 2 and day 7, respectively, for analyses, because exogenously administered glutamine requires sufficient time to impose measurable metabolic and immune phenotypes in the tumor microenvironment.

Moreover, we have included a later endpoint (day 14) to validate the effect of mid-term glutamine supplementation (**Fig. 2f**).

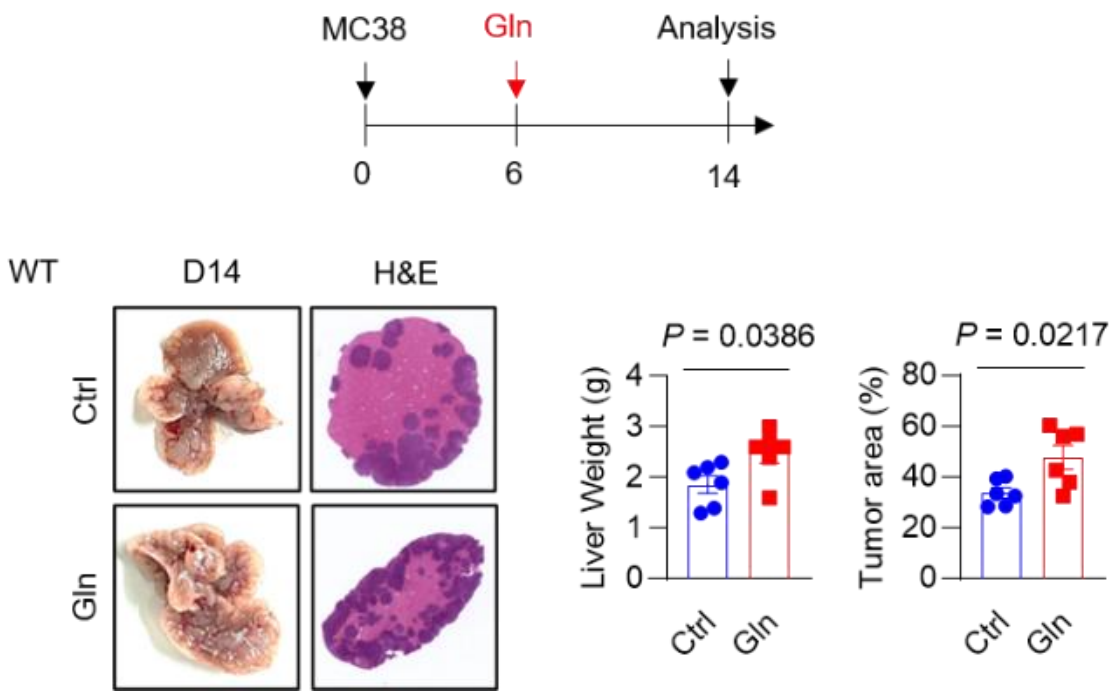

15. Surgery-induced hepatic stress (Fig. 3)

- Since intrasplenic injection and surgery can independently cause ER stress, control sham-groups are needed.

- → Include sham-operated WT and CKO mice, measure ALT, AST, serum glutamine, and assess monocyte flow and counts at day 2 post-surgery to exclude stress-related artifacts.

Response: We have added sham controls at day 2 post-surgery and found that the relevant parameters were comparable between *Xbp1*$^{fl/fl}$ and *Xbp1*^{CKO} mice.

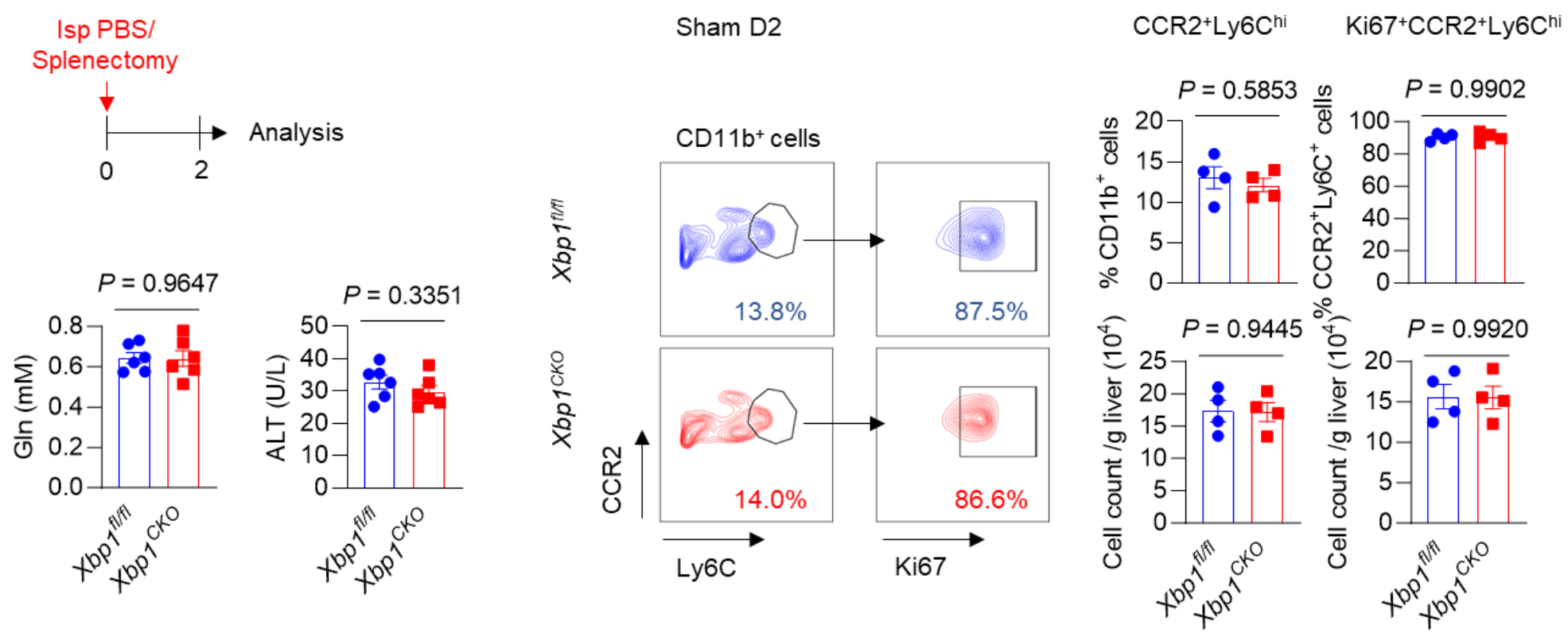

16. Extended Fig. 1j: The manuscript refers to Ern1CKO mice, but only Ctrl and Gln groups are shown—correct this labeling.

Response: We apologize for this mistake and have now corrected the labeling (**Extended Data Fig. 2i**).

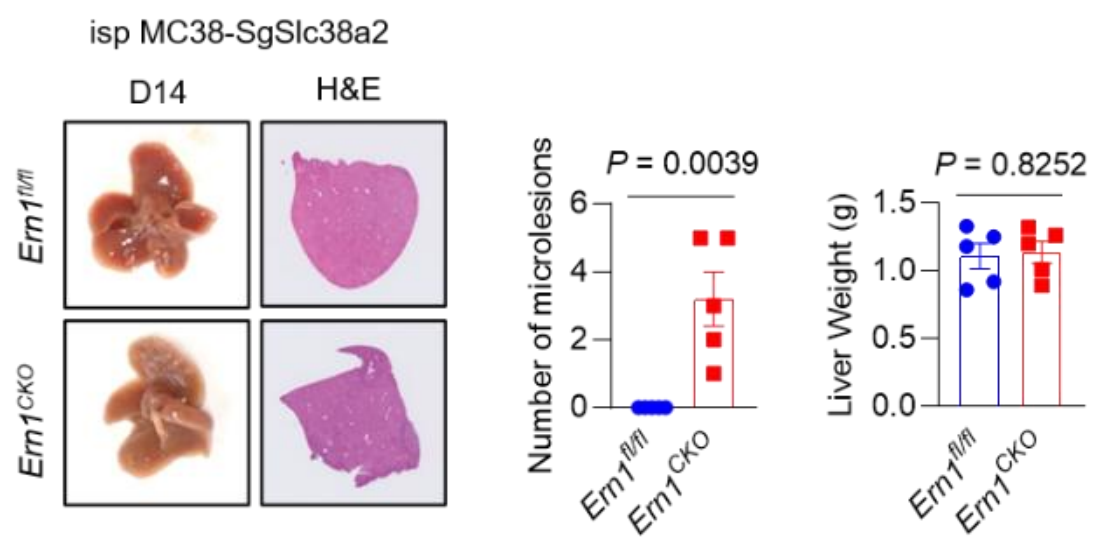

17. Fig. 5c: Provide a supplementary table with transcriptomic data. The upregulated macrophage

genes (Enpp1, Treml2, Scd1/2) indicate a lipid metabolism–biased, M2-like macrophage profile, consistent with published data linking glutamine to M2 polarization.

Response: We have added **Supplementary Table 3** to summarize glutamine-regulated genes in monocytes and macrophages. Although M2 macrophages are known as glutamine consumers, the pro-inflammatory milieu in this setting does not strongly support their polarization.

18. Extended Fig 5.: The authors should perform co-transfer experiments (e.g., CD45.1.2 WT and CD45.1 sgSlc38a2 monocytes 1:1 ratio in the same host, ideally Ern1CKO). Then, compare counts, % and proliferation within this host to disentangle cell-intrinsic versus environmental effects.

Response: As suggested, we co-transferred sgCtrl-expressing CD45.1/2 and sgSlc38a2-expressing CD45.2 BMIMs to prove that IM expansion depends on glutamine uptake (**Fig. 5e**).

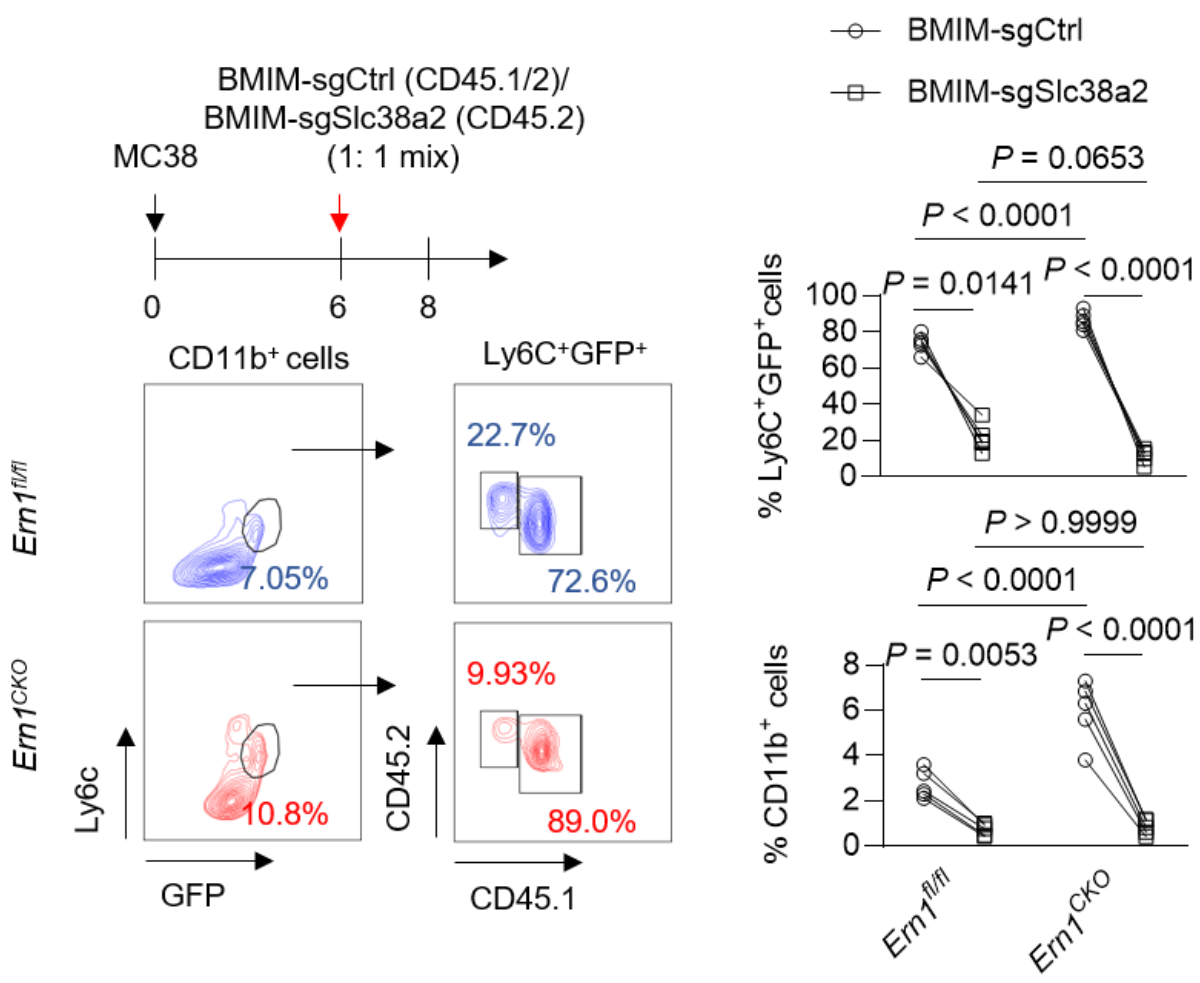

Extended Data Fig. 6f demonstrates a significant reduction in the proliferative capacity of SLC38A2-deficient BMIMs relative to controls under glutamine-replete condition.

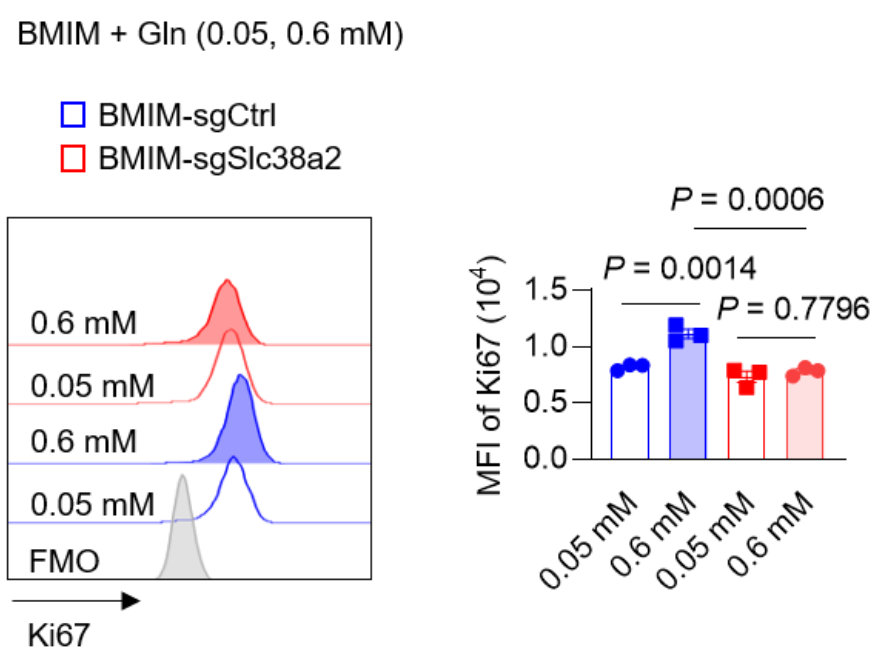

Reviewer #5&6 (Remarks to the Author):

The study investigates how hepatocyte-intrinsic IRE1α- XBP1 signaling shapes the liver metastatic

microenvironment. Contrary to the commonly tumor-promoting roles of IRE1a and XBP1, the authors propose that hepatocyte IRE1a XBP1 activity suppresses liver metastasis by limiting local metainflammation. They report that hepatocyte-specific deletion of IRE1a or XBP1 increases inflammatory monocyte accumulation and metastatic burden through a proposed non-canonical FoxO1 glutamine synthetase pathway that enhances glutamine secretion. Restoration of XBP1 or inhibition of glutamine metabolism is suggested to reduce monocyte levels and limit metastasis. Limited patient data are presented to support a positive correlation between hepatocyte XBP1 expression and survival. The manuscript therefore aims to redefine hepatocyte unfolded protein response signaling as a regulator of monocyte-supported metastatic growth.

While the manuscript uses an impressive number of mouse models, a central concern is the lack of essential control experiments validating the mouse models.

Response: We appreciate the reviewer's concern and have accordingly included the appropriate control groups where necessary.

The Ern and Xbp1 hepatocyte specific knockout models are not adequately confirmed. It remains unclear how hepatocyte specific deletion was verified, whether IRE1 signaling is demonstrably reduced, and whether baseline liver phenotypes differ in a way that could influence metastatic susceptibility. These questions directly affect interpretation of downstream findings.

Response: The *Xbp1*$^{fl/fl}$ and *Ern1*$^{fl/fl}$ mice mouse strains used in this study were kindly provided by Prof. Laurie Glimcher (Dana-Farber Cancer Institute) and Prof. Yong Liu (Wuhan University), respectively. Both strains have been well characterized for efficient hepatocyte-specific gene deletion in the original reports (PMID: 18556558; PMID: 24670948). We still validated the expression of IRE1α and XBP1s in *Ern1*$^{fl/fl}$ and *Ern1*^{CKO} hepatocytes. Because basal IRE1α levels were undetectable in *Ern1*$^{fl/fl}$ hepatocytes, tunicamycin (Tm, a classic ER stress inducer) treatment was used as a positive control (**Extended Data Fig. 7e**).

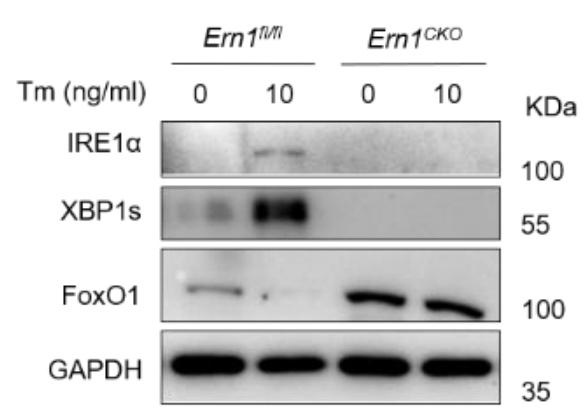

Our previous flow-cytometric analyses showed similarly low levels of monocyte infiltration in tumor-free *Ern1*$^{fl/fl}$ and *Ern1*^{CKO} livers (**Extended Data Fig. 5a**). Consistent with this, liver TUNEL and Ki67 staining

indicated that hepatocyte-specific IRE1α depletion does not induce overt liver damage or compensatory proliferation.

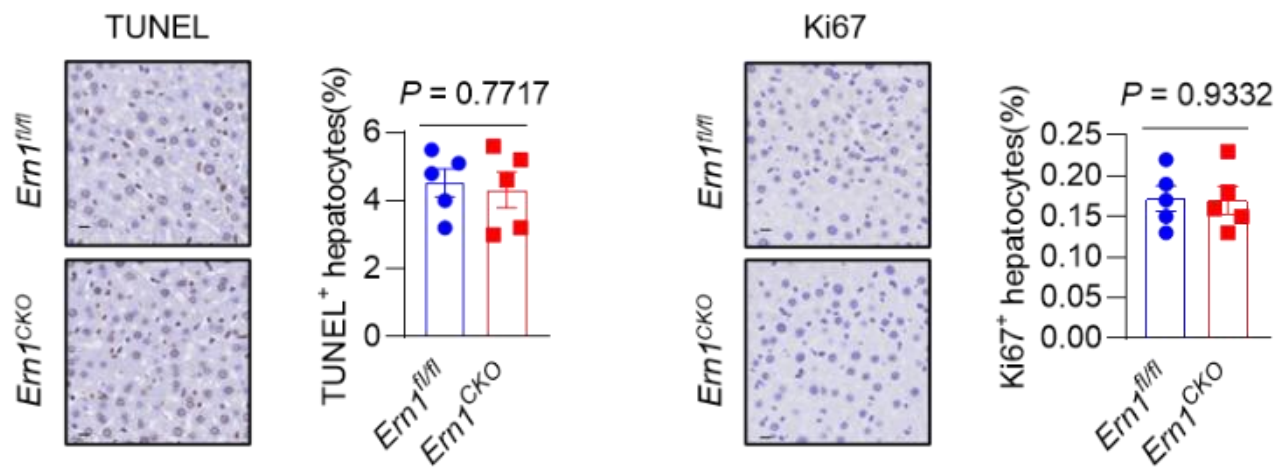

Flow cytometry analysis revealed similarly low levels of monocyte infiltration in tumor-free *Ern1*$^{fl/fl}$ and *Ern1*^{CKO} livers (**Extended Data Fig. 5a**), as was also observed for other immune populations.

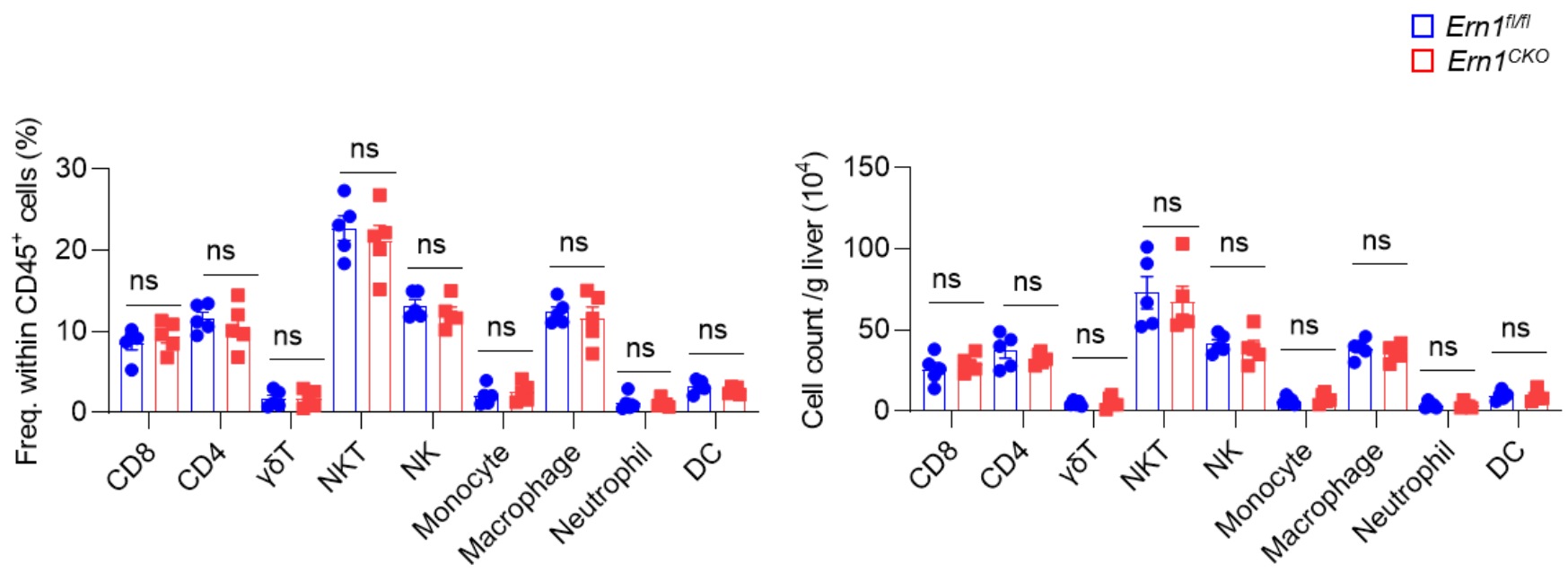

Other major points

• L133: The XBP1 overexpression model seems to lack validation. The manuscript does not seem to convincingly show that functional XBP1 is increased. Since both knockout and overexpression models underpin major mechanistic claims, they require much stronger characterization.

Response: We employed a well-established approach to achieve in vivo overexpression of XBP1s in mouse hepatocytes, as previously described (PMID: 21317886). In addition to elevated XBP1s expression in hepatocytes (Fig. 1h), the canonical XBP1s transcriptional target *Dnajb9* was also upregulated in ADV-XBP1s-treated mice, indicating enhanced XBP1s signaling activity in hepatocytes.

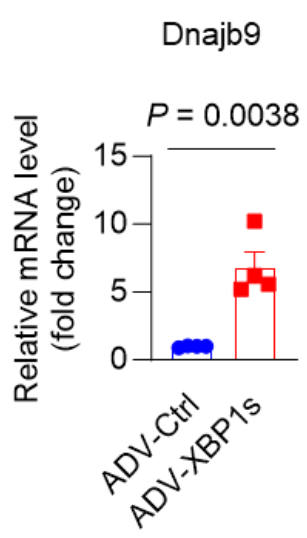

• Control experiments for SLC38A2 depletion are missing.

Response: We used MC38-sgSlc38a2 versus MC38-sgCtrl cells to confirm that glutamine uptake is essential for liver metastasis formation (**Extended Data Fig. 2h**).

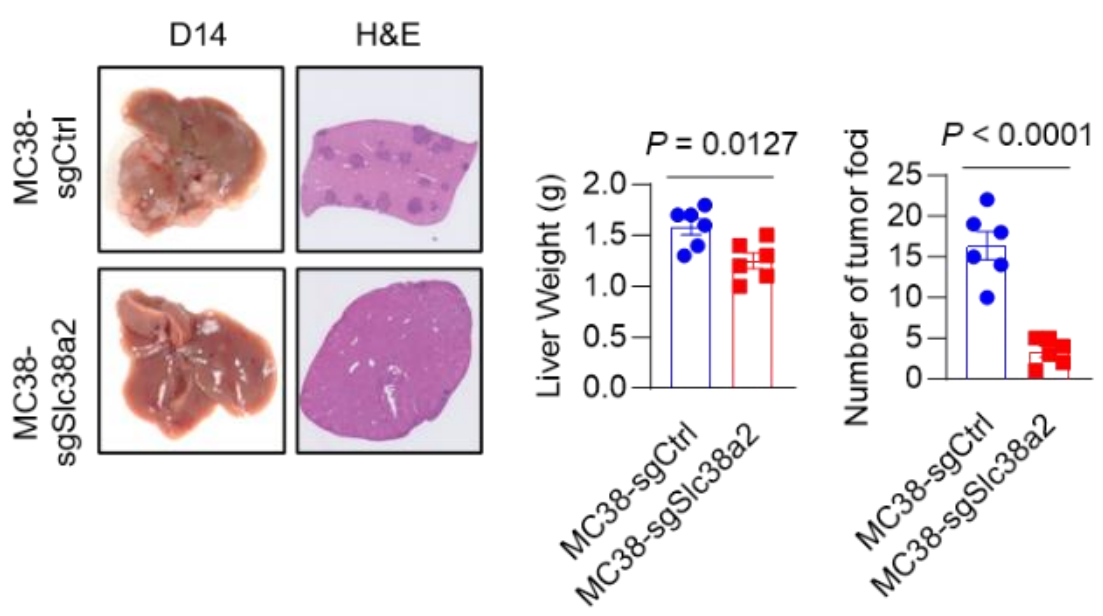

• Extended Data Fig 2b to f: Control tumor areas vary from ten to fifty percent despite samples being collected the same day. Intra experimental variation is smaller than inter experimental variation. Additional antibody treatment controls would improve interpretability.

Response: In the experiments previously shown in Extended Data Fig. 2b–f (now **Extended Data Fig. 3c–h**), both *Ern1*$^{fl/fl}$ and *Ern1*^{CKO} mice were treated with the indicated antibodies. These experiments were conducted alongside wild-type controls and treatment-naïve *Ern1*$^{fl/fl}$ mice. Although all comparisons within this experimental framework were conducted between *Ern1*$^{fl/fl}$ and *Ern1*^{CKO} mice, we have now included an isotype control condition as requested by the reviewer (**Extended Data Fig. 3c**).

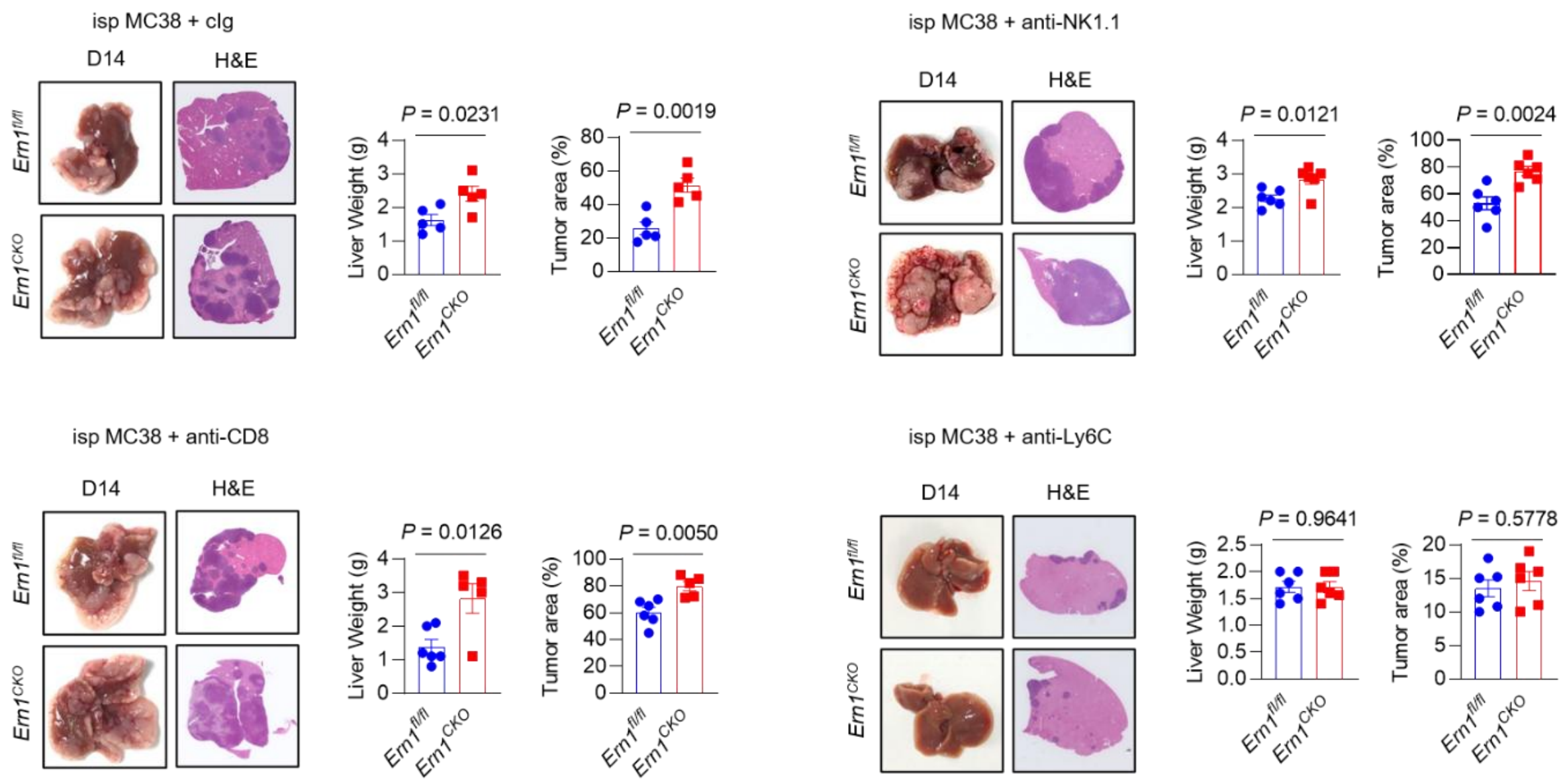

• l370 and l374: Claims regarding hepatocytes with abundant GS expression and invasive margins heavily infiltrated by Ly6C positive monocytes rely on a single small image (roughly two hundred fifty micrometers squared) without quantification. Assertions about XBP1 low GS positive areas are not supported by shown images. GS localization differs between ADV Ctrl and ADV XBP1s treated mice, and monocyte distribution appears similar across most images except for the XBP1 fl/fl example. Proper quantification across multiple samples is required.

Response: Quantification has been conducted based on six samples per group (**Extended Data Fig. 8b, f**). It should be noted that zone 3 hepatocytes in mice of all genotypes intrinsically express GS (As indicated in the legend, Zone 3 GS expression was excluded for quantification). In this setting, XBP1s overexpression significantly reduced peritumoral GS expression (and monocyte infiltration), while leaving the constitutive zone 3 GS expression unaffected.

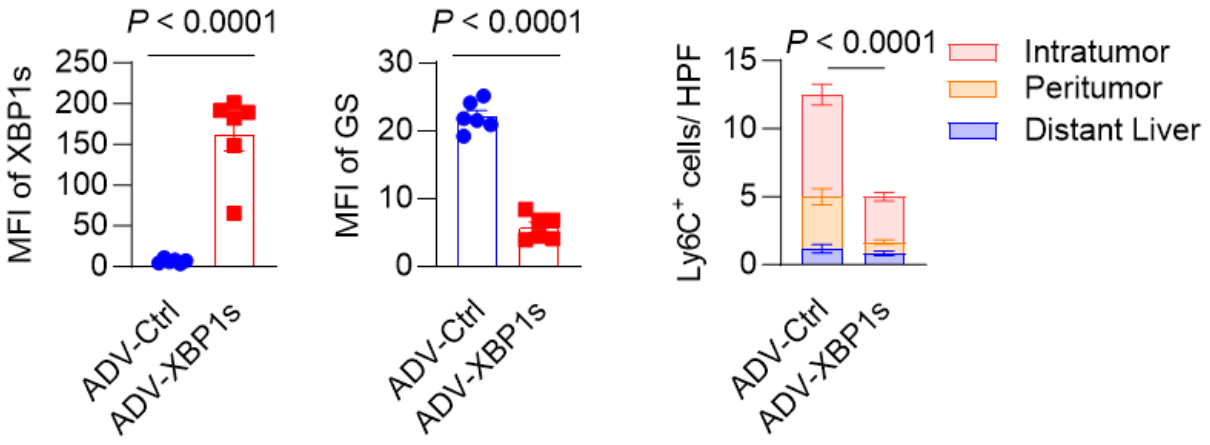

• CCR2 antagonist and related treatments: Figures lack side by side comparisons between treated and control animals. For instance, treated mice show a mean tumor area of around seven point five percent versus roughly forty percent in untreated mice, yet the direct comparison is not displayed (Fig 3g).

Response: We appreciate the reviewer's suggestion and therefore have added vehicle-treated groups in this setting (**Fig. 3g**).

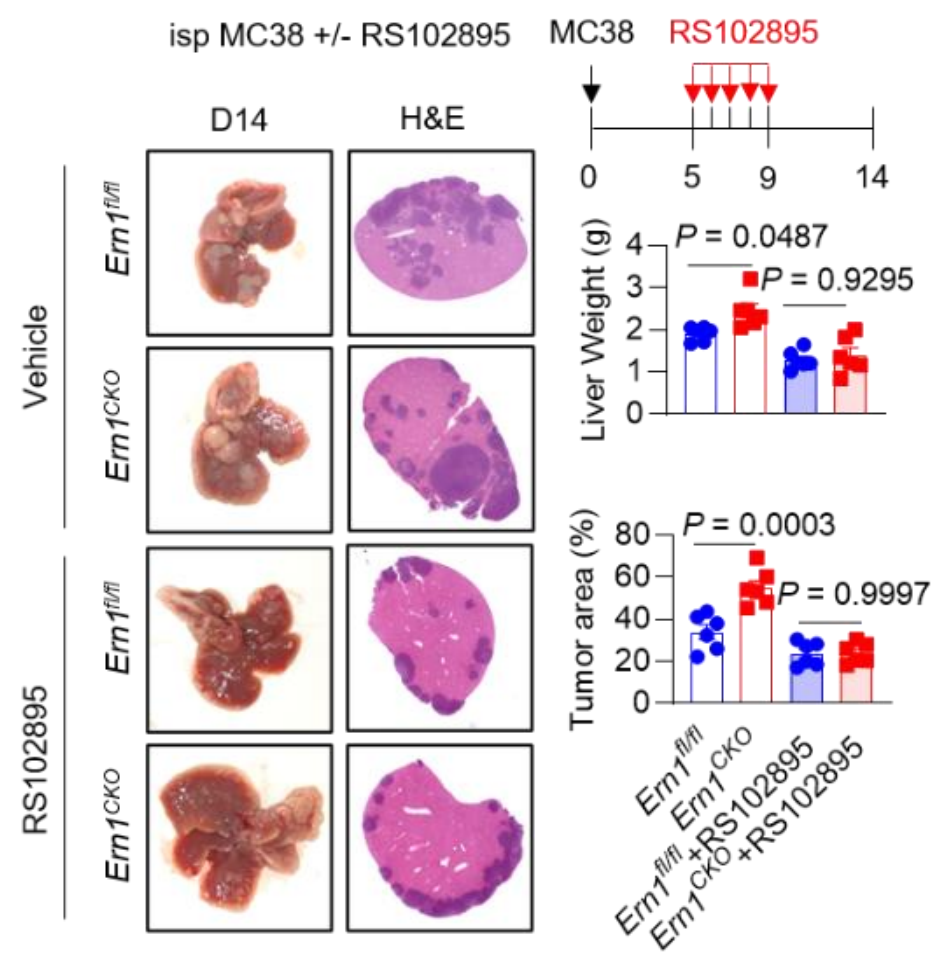

• Figure 6a: The upper panel does not convincingly show tumor cells. Morphology differs from verified tumor cells in earlier figures. In the lower panel, GS positive cells are perivascular, and the inset suggests a chance alignment with regions designated as tumor. These images do not convincingly support the stated differences in GS positivity.

Response: The upper panel of **Fig. 6a** (*Ern1*$^{fl/fl}$ group) was generated from the same batch of liver sections as those shown in Fig. 2a and exhibits tumor sizes comparable to those observed in *Xbp1*$^{fl/fl}$ livers in Fig. 6b. We have replaced the lower panel of Fig. 6a (*Ern1*$^{fl/fl}$ group), which displayed larger tumor sizes than expected at day 7.

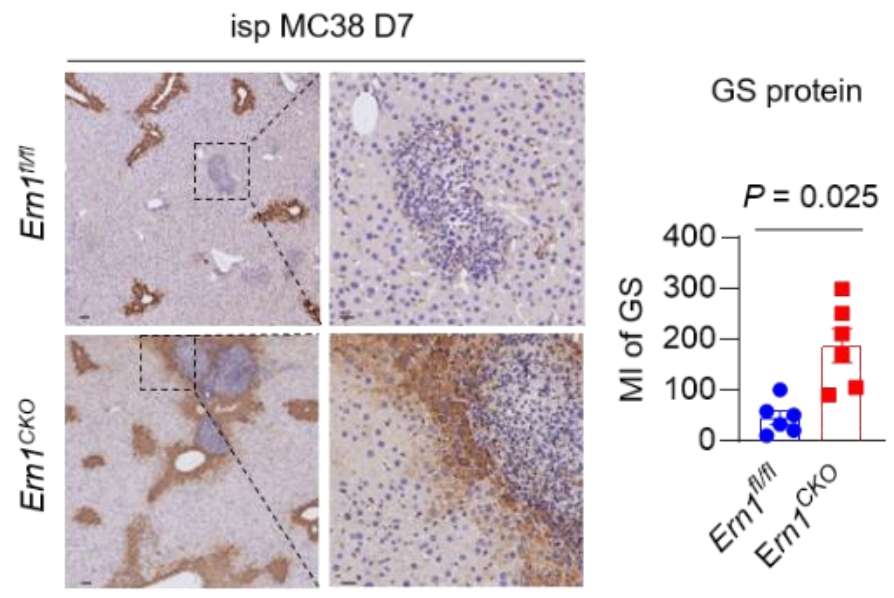

• Figure 7: The Kaplan Meier plots lack essential information. It is not clear how many patients were censored or the size of each group. Censoring indicators for the red curves are nearly invisible.

Response: We appreciate the reviewer's insight and have accordingly added the relevant information and adjusted the corresponding indicators (**Fig. 7d, e**).

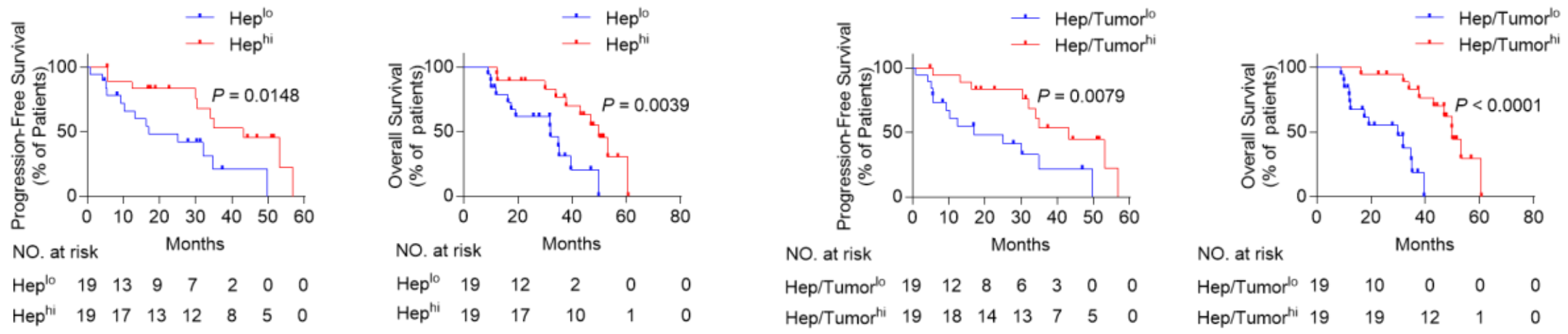

• Extended Data Fig 7b: Tissue labeled as tumor is in fact a fibroinflammatory rim or stromal capsule, not tumor. Interpretation of related immunofluorescence panels is difficult because HE images are missing.

Response: We have accordingly repositioned the labels to the bona fide tumor regions, as stromal tissue is present between the peritumoral liver and metastatic lesions (shown in revised **Extended Data Fig 7g**).

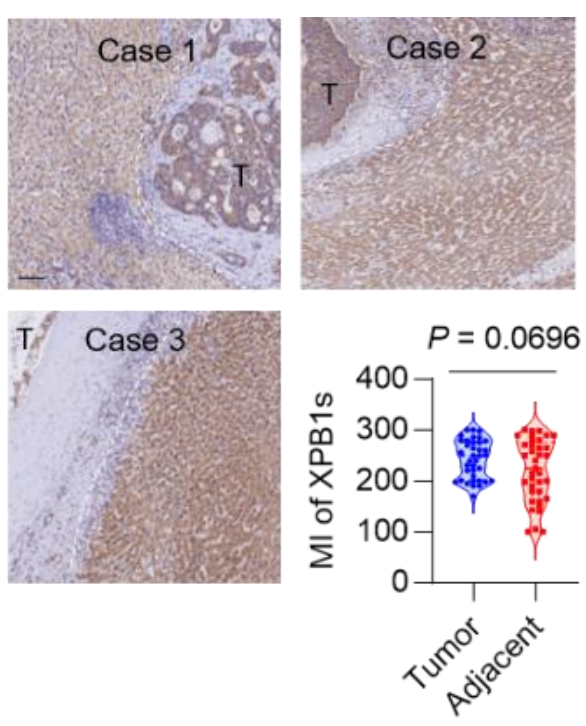

Other points:

• l87: Hepatocytes may not be functionally intact in premetastatic niches. They can already be altered, as shown for example by Bojmar in Nature Medicine twenty twenty four.

Response: We have accordingly changed "functionally intact" into "free of ER stress" (**Line 90**).

• l119: Liver weights are used as surrogate markers for tumor burden, which is not established. Tumor size should be quantified directly.

Response: In each experimental setting, metastatic burden was quantified based on H&E staining and is presented as the percentage of tumor area relative to total liver area.

• The protein folding roles of XBP1 and IRE1 should be mentioned for context.

Response: As a general function of the canonical ER machinery, protein-folding capacity was briefly introduced at the beginning of the Introduction. Because this aspect is not directly relevant to the specific functionality of the IRE1α–XBP1s axis examined in this study, we did not expand on it further.

• XBP1s should be referred to as XBP1.

Response: As introduced in the first paragraph, the spliced form of X-box binding protein 1 is referred to as XBP1s, which is distinct from total XBP1 and from XBP1u, the unspliced form of XBP1, as defined in the ER stress field.

• l190: The term metastatic outbreak is unclear and should be defined.

Response: The explanation is embedded in the particular sentence (**Line 150**), where metastatic outbreak refers to the transition from clinically invisible or quiescent micrometastases to overt, actively growing macrometastases. Notably, the term "metastatic outbreak" has also been used in a recent publication (PMID: 40707738), supporting its usage in this context.

• l237: The phrase disease associated expansion is misleading. The data only show the presence of cells, not their expansion. The figure also does not compare tumor versus normal liver but rather shows very limited regions containing a few double positive cells.

Response: We have replaced the phrase "disease-associated expansion" with "a disease-accompanying pattern" (**Line 257**), as $CCR2^{+}Ly6C^{+}$ inflammatory monocytes were primarily detected at tumor edges (**Fig. 3f**).

The normal liver parenchyma outside the metastatic niche is not expected to substantially accumulate inflammatory monocytes, as tumor cell presence remains the key trigger for the hepatocyte–monocyte communication identified in this study.

• l75 to l76: The phrase in the presence of hepatocyte IRE1a may be imprecise. It might be more accurate to refer to conditions where IRE1a is activated.

Response: We have rephrased it to "elevated hepatocyte IRE1α expression sensitizes the liver to hepatocellular carcinoma (HCC) development" (**Line 77–78**).

• The manuscript does not clarify which liver regions were used for single cell RNA sequencing or flow cytometry. It is unclear whether whole liver tissue including tumor and surrounding parenchyma was analyzed.

Response: We indicated that "we sorted CD11b$^+$ liver-infiltrating immune cells from *Ern1$^{fl/fl}$* and *Ern1^{CKO}* mice on day 7 post isp of MC38 cells". At this time point, whole liver tissues were processed for myeloid cell isolation, corresponding to the transition of metastatic lesions from micrometastases to overt macrometastases.

• Fig 3f immunofluorescence: The precise location of the imaged region is unclear, likely intratumoral but not confirmed.

Response: As shown in revised **Fig. 3f**, quantification has been carried out to present IM abundance in both tumor lesions and the surrounding tissue. CCR2$^+$Ly6C$^+$ inflammatory monocytes were predominantly enriched in intratumoral regions, with the highest density observed at the tumor edges.

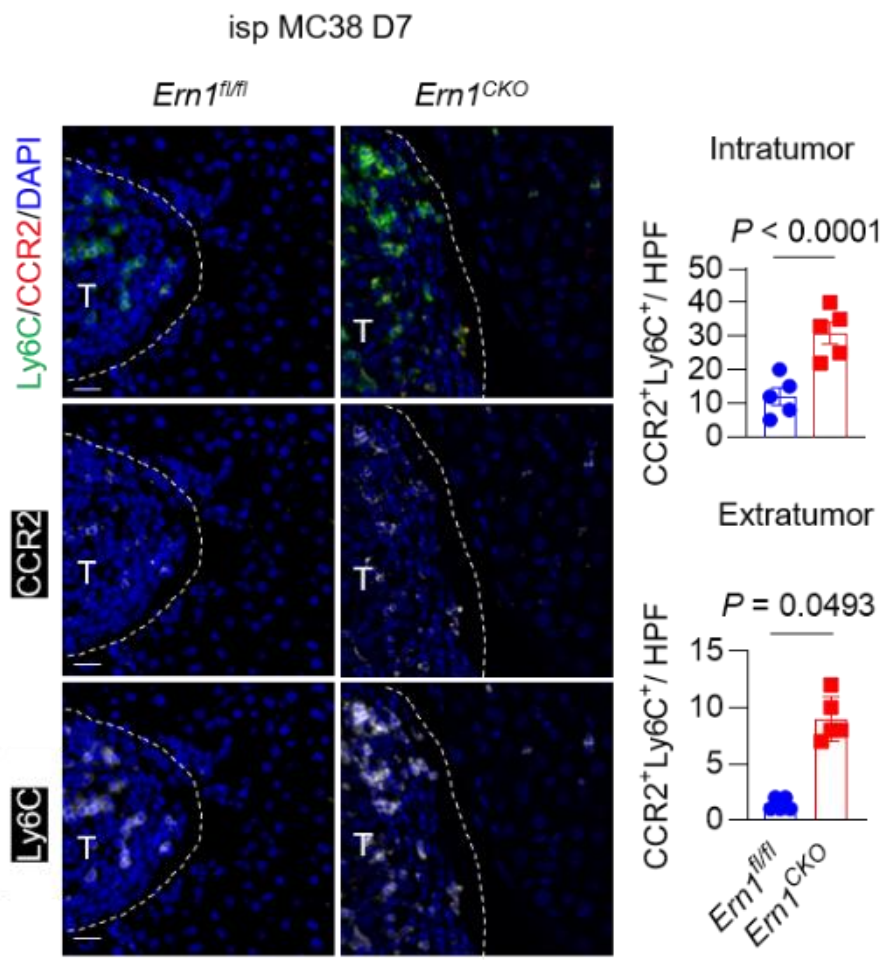

• HE quantification based size appears in Fig 1b and Extended Data Fig 1b, not in Fig 1a and Extended Data Fig 1a as stated in l123 to l124.

Response: We appreciate the reviewer for pointing this out and have accordingly removed "Fig. 1a" and "Extended Data Fig. 1a."

• In vitro SLC38A2 transporter depletion is shown in Extended Data Fig 1h, i, j, not 1h and I as written in l181.

Response: We appreciate the reviewer for pointing this out and have edited it accordingly (now shown in **Extended Data Fig. 2f–i**).

• Extended Data Fig 1j is labeled Gln on the x axis, but appears instead to compare Ern1 fl fl in blue versus Ern1 CKO in red.

Response: We appreciate the reviewer's scrutiny and have corrected the labels (now shown in **Extended Data Fig. 2i**).

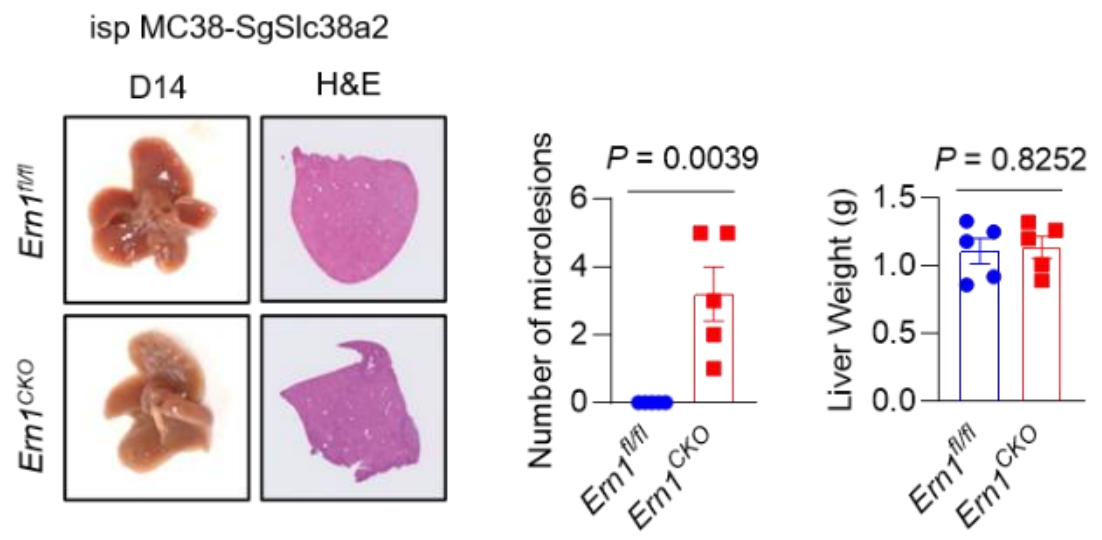

• l310 and following: The figure does not show a reduction in Ki67 positive CCR2 positive cells relative to BMM sgCtrl injection. Instead, it compares Ern1 fl fl to Ern1 CKO. The text therefore does not accurately describe the displayed data.

Response: We have co-transferred sgCtrl-expressing CD45.1/2 and sgSlc38a2-expressing CD45.2 bone marrow monocytes to prove that IM expansion depends on glutamine uptake (**Fig. 5e**).

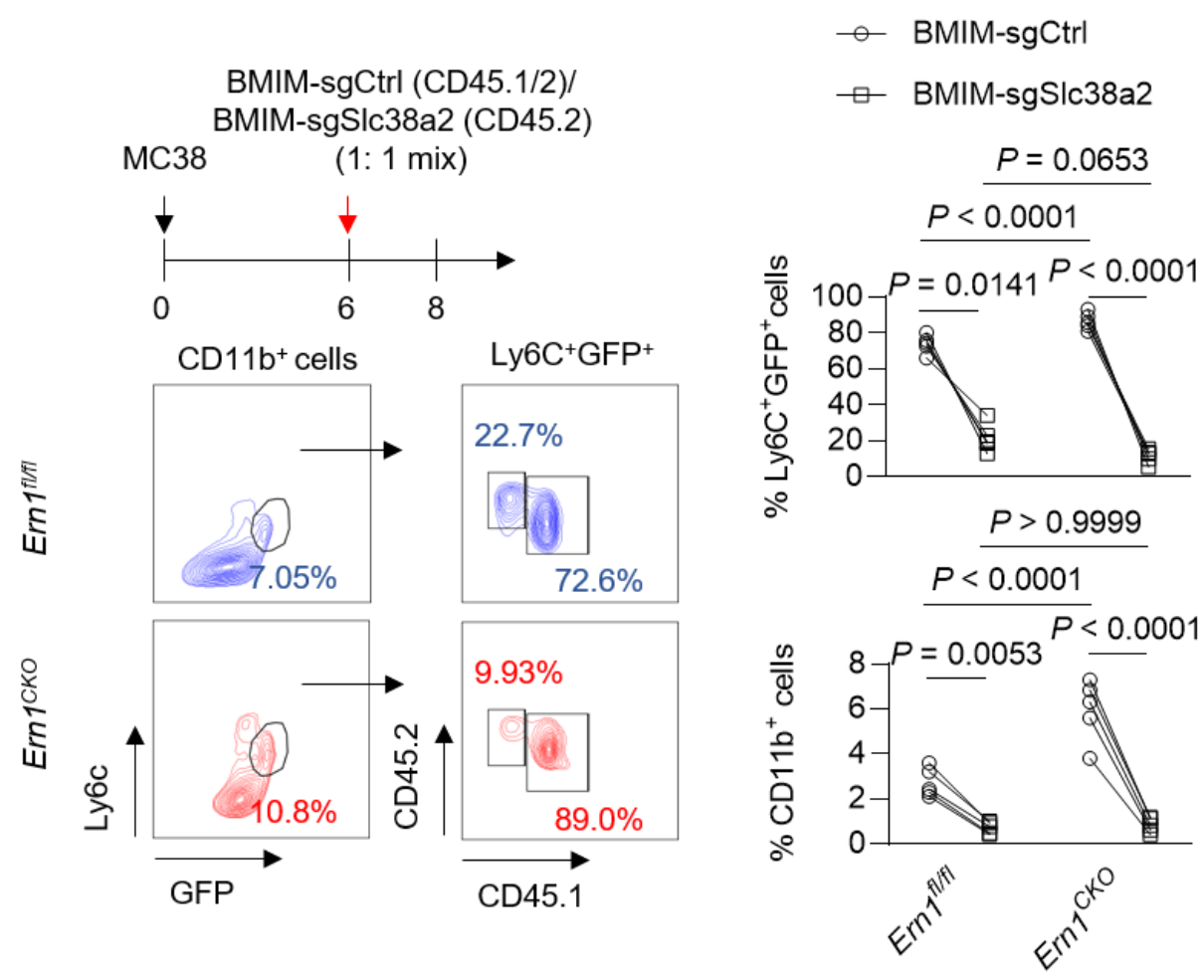

Extended Data Fig. 6f demonstrates a significant reduction in the proliferative capacity of SLC38A2-deficient BMIMs relative to controls under glutamine-replete condition.

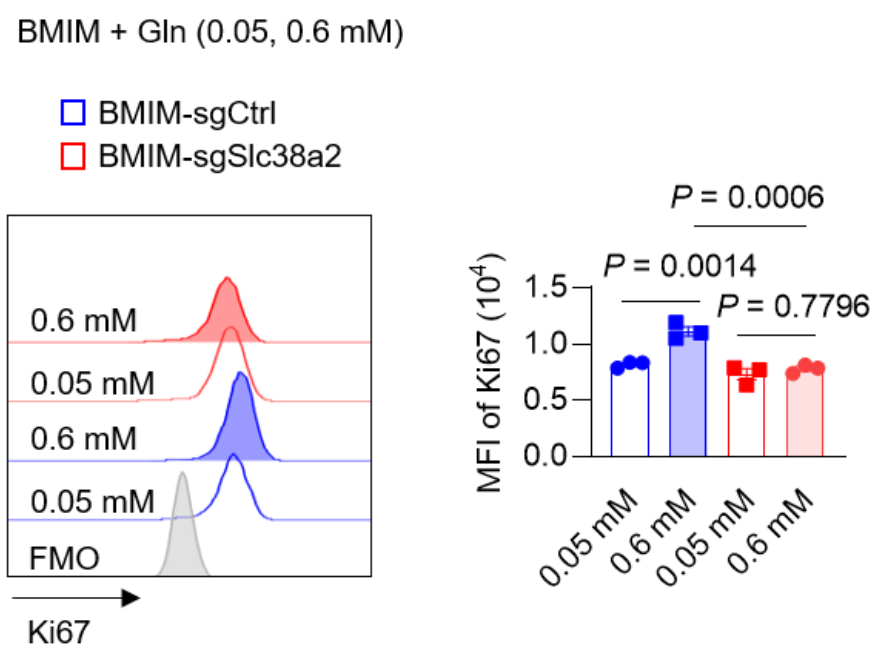

Similarly, in Fig 5f with DRP 104 treatment, the authors claim a significant reduction in endogenous monocytes, but the comparison shown does not support these conclusions.

Response: As suggested, we have now provided control groups in **Fig. 5f** to facilitate comparison.

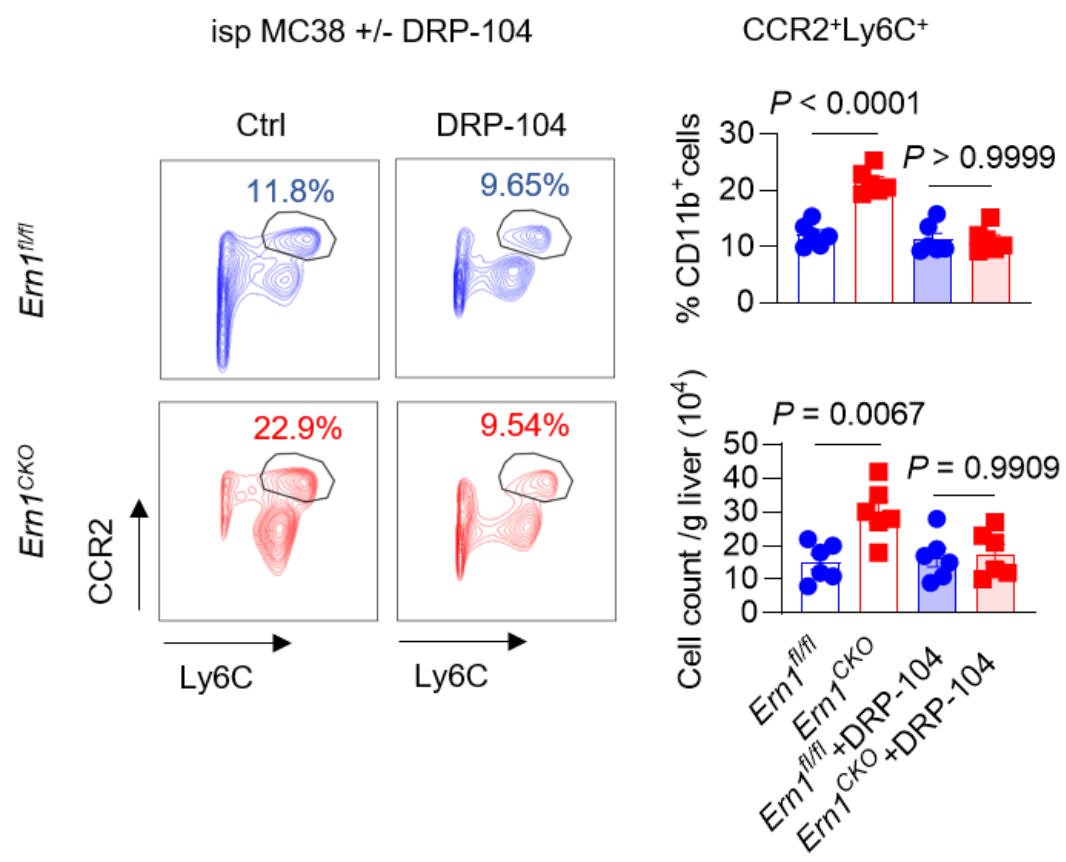

• l226 and following: Cluster one is described as expressing lineage markers and pro inflammatory signatures. However, complement components such as C1qa, C1qb, and C1qc, as well as Mif and Ccl2, are commonly expressed by macrophages and do not necessarily reflect a pro inflammatory or cancer promoting phenotype. The interpretation should be refined.

Response: Lineage markers can be used to distinguish inflammatory monocytes from macrophages, as inflammatory monocytes express substantially higher levels of Ly6c and Ccr2. While complement components (C1qa/b/c), Mif, and Ccl2 are not exclusive to inflammatory monocytes, their coordinated expression within the expanded CCR2$^+$ monocyte population is consistent with a tumor-supportive inflammatory program previously described in metastatic niches. Accordingly, we have refined the sentence to "characterize them as cancer-promoting IMs in this context" (**Line 243**).

• The manuscript proposes that IRE1a and XBP1 deficient hepatocytes foster monocyte accumulation, but the causal evidence remains incomplete.

Response: We have provided multiple complementary lines of evidence supporting a causal role for hepatocyte IRE1α/XBP1 deficiency in fostering monocyte accumulation. In vivo, both endogenous analyses and exogenous tracing experiments consistently demonstrate that loss of hepatocyte IRE1α or XBP1 increases inflammatory monocyte (IM) accumulation, whereas restoration of this signaling exerts the opposite effect. In addition, in vitro conditional culture experiments showed that supernatants derived from IRE1α-deficient hepatocytes are sufficient to accelerate IM expansion. Having established in both systems that environmental glutamine is necessary, we further provided mechanistic evidence demonstrating that a hepatocyte-intrinsic XBP1s–FoxO1–GS axis is fundamental for glutamine regulation. Interference with this axis at multiple nodes—including inhibition of IRE1α, GS, or glutamine availability—consistently abolished monocyte accumulation, thereby reinforcing the causal link.

• Fig 5d: The y axis range obscures transcripts above the significance threshold. Genes such as Siglec1 appear likely to exceed the cutoff but cannot be clearly seen. Plot scaling should be adjusted.

Response: We have now adjusted the y-axis range accordingly (now shown in **Extended Data Fig. 6g**), and the underlying dataset has been provided in **Supplementary Table 3**.

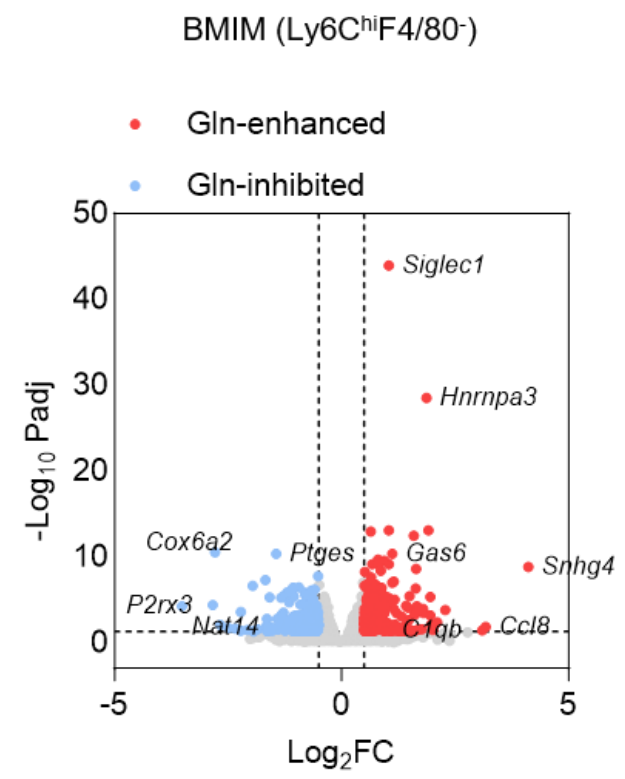

• Fig 6a immunohistochemistry quantification: The marker index is not defined, and measurement locations are unclear.

Response: In addition to the existing y-axis label of the histogram, we have now added an annotation ("GS protein") above the plot. Quantification was performed across entire liver sections, although GS expression is predominantly localized to zone 3 hepatocytes.

• Fig 6 western blots: Quantitative summaries of reproducible experiments seem to be missing. Reported changes, such as GS increases with decreasing glutamine or higher FoxO1 in Ern1 CKO, are not evident in the images. Replicate numbers must be stated.

Response: We repeated the experiment using a shorter western blot exposure and have replaced the GS and FoxO1 bands accordingly in **Fig. 6c**. We have also provided densitometric quantification below accordingly (replicates number = 3).

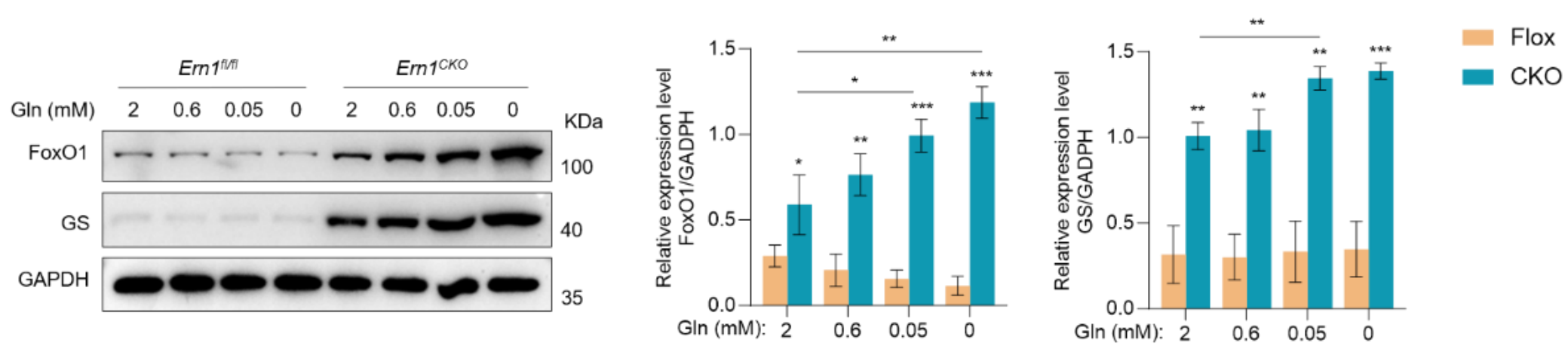

• Fig 6f: Non treated control data should be shown alongside treated data.

Response: We have accordingly included the appropriate control groups for comparison, which are now presented in **Fig. 6g**.

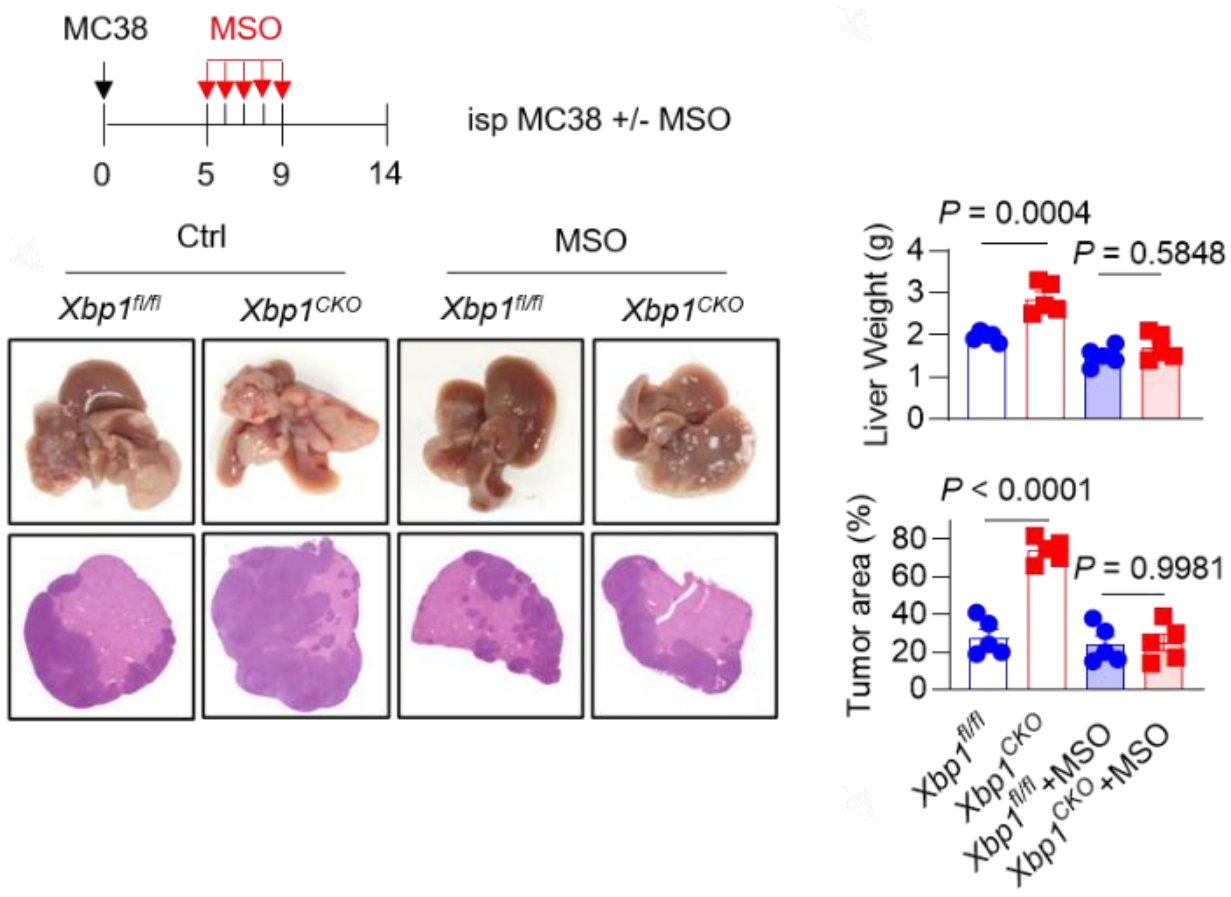

• Fig 7b: Control mice treated with ADV Ctrl do not express XBP1s near metastases, which seems inconsistent with Fig 1h. This discrepancy needs to be addressed.

Response: In control ADV-treated wild-type mice, hepatocytes express XBP1s at low basal levels. This expression is readily detectable by conventional immunohistochemistry but falls below the detection threshold of multiplex immunofluorescence. This slight discrepancy therefore reflects differences in sensitivity between these two detection methods rather than a biological inconsistency.

• In situ data generally show small tissue regions with limited quantification. Monocyte phenotyping is restricted to Ly6C or CD14, although CD14 is also expressed by macrophage subsets. More detailed phenotyping is needed.

Response: CCR2 is a well-established marker of inflammatory monocytes (IMs) (PMID: 21654748; 23081697). Our multiplex immunofluorescence was employed as a validation approach to visualize hepatocyte–IM contact. Consistent with this, accumulation of CCR2^{+}Ly6C^{hi} inflammatory monocytes was clearly evident in **Fig. 3**, as demonstrated by both immunofluorescence and flow cytometric analyses. Quantification has now been carried out to present IM abundance in both tumor lesions and the surrounding tissue (**Fig. 3f**).

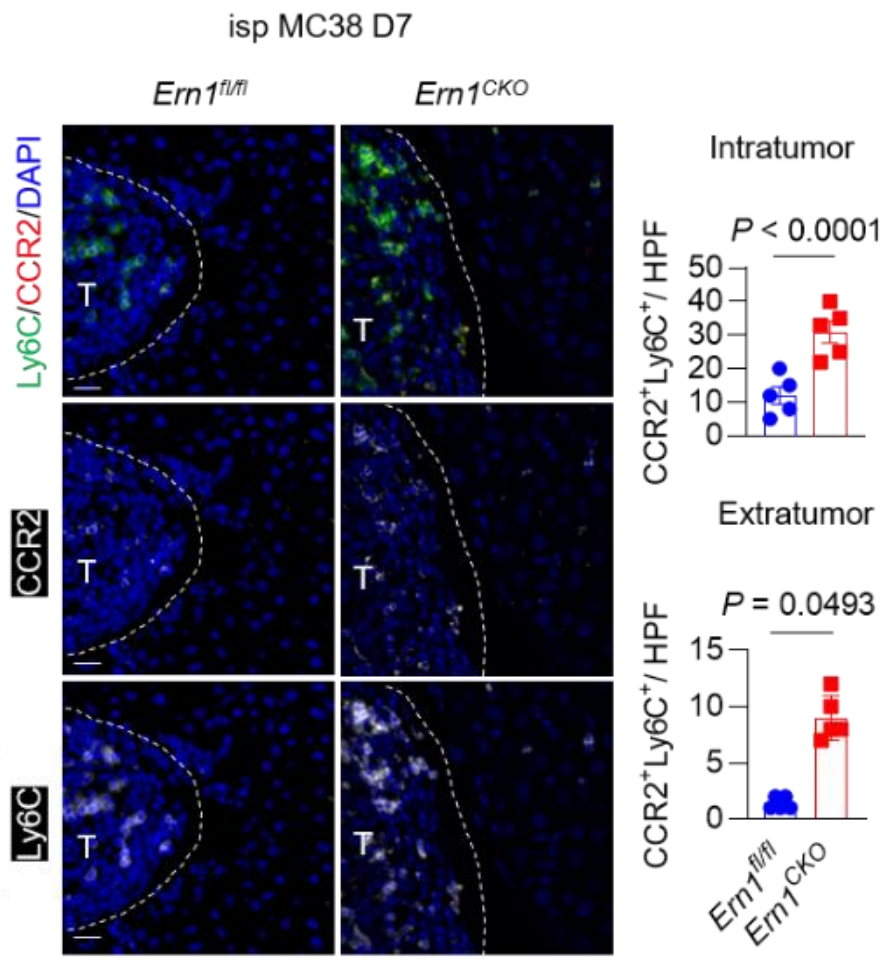

• The term metainflammation is used without sufficient introduction. It is primarily associated with metabolic disease and needs clearer explanation when invoked in the context of hepatocyte derived signals near metastases.

Response: We appreciate the reviewer's point. In this study, we refer specifically to hepatocyte-derived metabolic regulation of inflammatory monocytes within the liver metastatic niche. To avoid ambiguity, we have replaced the term "metainflammation" with "local metainflammation" (**Line 398**), consistent with our usage in the Abstract.

Reviewer #1 (Remarks to the Author):

The authors have experimentally addressed all the questions and concerns that were raised previously. I have no further questions with the revised manuscript. Thanks for their effort for improving the work before publication.

Response: We thank the reviewer for the appreciation of our work.

Reviewer #2 (Remarks to the Author):

Authors have addressed most of the major raised comments. However, I still have reservations about their metastasis model, which is does not endogenously occur. Also, I don't understand why they have removed all the axis labels in the revised figures to address the formatting issues pointed out in the original manuscript. That said, the additional experiments have significantly strengthened the study, and I think the manuscript has now reached an acceptable standard for publication.

Response: We appreciate the reviewer's continued attention to model selection. We respectfully note that spontaneous liver metastasis models are not the prevailing standard for mechanistic studies of hepatic niche biology in high-impact journals. The majority of seminal papers defining liver immune regulation including studies in *Nature*, *Nature Immunology*, and *Science Immunology* have relied on portal-vein or intrasplenic delivery models rather than spontaneous metastasis. These models are widely accepted because they (i) faithfully recapitulate the physiological route of liver colonization, (ii) provide experimental control over timing and metastatic burden, and (iii) avoid systemic confounders inherent to spontaneous models.

Importantly, we have already validated our findings in the 4T1 spontaneous metastasis model, which is one of the few systems that reliably produces liver metastases. The liver-specific resistance to IRE1α inhibition observed in this spontaneous model fully corroborates our mechanistic conclusions.

Together, the combination of a spontaneous model and the field-standard portal-vein model provides rigorous and sufficient evidence. We therefore believe the current model selection aligns with community practice and is appropriate for the mechanistic scope of this study.

Regarding formatting issues, we would like to clarify that axis labels have not been intentionally removed from any revised figure. All plots retain their original axis labels (two plots may share one same y-axis label). If any labels appear missing in the reviewer's PDF, this may be due to resolution or compression during the manuscript upload process, as the source files contain complete axis

annotations. To avoid any ambiguity, we have re-exported all figures at higher resolution and verified that every axis label is present and legible. We hope this resolves the reviewer's concern.

Reviewer #3 (Remarks to the Author):

The authors have addressed my concerns.

Response: We thank the reviewer for the appreciation of our work.

Reviewer #4 (Remarks to the Author):

The authors address my concerns and this current format present compelling results for supporting their exciting findings.

Response: We thank the reviewer for the appreciation of our work.

Reviewer #5 (Remarks to the Author):

The authors have responded to all concerns raised during the previous review round. Any points from the first revision not revisited below are considered satisfactorily addressed.

In response to the point that the authors use liver weight as a surrogate for tumor burden, they state that they quantify tumor burden directly, which is acknowledged. However, the text continues to focus on liver weight when comparing experimental groups, e.g., in the first section of the results (L121 and following). As mentioned earlier, liver weight is not an established readout of tumor burden, and its baseline values vary markedly across experimental groups. This is especially evident in the control group shown in Fig. 1g when compared to the control groups shown in Fig. 1b–f, among others. We suggest amending the wording and focusing on tumor size throughout instead.

Response: We thank the reviewer for this helpful clarification. In all experiments, tumor burden was quantified directly by histological measurement of metastatic area or foci number, and these values form the basis of our conclusions.

Liver weight was originally included as an auxiliary descriptor of overall organ involvement, as is common in liver metastasis studies. While liver weights of MKC8866-treated mice were initially reported in Line 121, histologically quantified tumor burdens were subsequently provided in Line 124, and both measurements are presented within the same figure panel. We also appreciate the reviewer's observation that liver weights were comparable among wild-type and floxed control groups at the

experimental endpoint of MC38 metastases, with the exception of the control group shown in Fig. 1g. In this experiment, control mice received a control adenoviral vector, which may slightly affect liver physiology and consequently influence tumor progression.

Regarding validation of the hepatocyte-specific knockout mouse models, the authors state that the models have been characterized previously. They also provide one single Western blot showing that hepatocytes isolated from Ern1CKO mice no longer respond to tunicamycin treatment (at one selected concentration) with upregulation of IRE1α and XBP1 protein levels, while showing increased FoxO1 protein levels compared to hepatocytes isolated from Ernfl/fl mice. This result validates the loss of stress response pathway components in the main knockout model used. In addition, the authors provide Ki67 and TUNEL immunolabeling results as indicators of hepatocyte proliferation and apoptosis in tumor-distant livers, as well as broad immune cell composition data from the tumor microenvironment. Both datasets compare Ernfl/fl and Ern1CKO mice in the metastatic context. However, whether baseline liver phenotypes that may affect metastatic seeding, outgrowth, or progression are present in the models used in vivo remains incompletely resolved. The manuscript would benefit from additional characterization of the naïve (non-tumor-bearing) livers of Ern1fl/fl versus Ern1CKO mice, for example, liver architecture/histology, and markers of hepatocyte stress or the unfolded protein response, to clearly establish whether loss of Ern1 alters the hepatic microenvironment independent of metastatic colonization.

Response: We thank the reviewer for highlighting the importance of baseline liver characterization. We would like to clarify that the hepatocyte-specific *Ern1* knockout model used in our study has already been comprehensively validated in the original report (*Journal of Hepatology*, 2015, PMID: 25457211), including full histological assessment of naïve *Ern1*^{CKO} livers. In that publication, H&E staining and western blots demonstrated normal liver architecture, no spontaneous inflammation, and no evidence of ER stress-associated pathology in the absence of tunicamycin. Importantly, we have independently reproduced these findings in our own facility using the same strain provided by Prof. Liu. Naïve *Ern1*^{CKO} livers in our hands show normal histology, unchanged hepatocyte proliferation and apoptosis (Ki67 and TUNEL), and no alterations in baseline immune composition. These results confirm that hepatocyte-specific *Ern1* deletion does not create a pre-existing pathological microenvironment that could influence metastatic seeding or outgrowth.

We argued previously that the in situ evidence provided does not sufficiently support the cell sorting–based findings on monocyte infiltration, and that the abundant presence of these cells in the tumor microenvironment upon hepatocyte modification was not adequately demonstrated. In particular, the methodological description of these results was insufficient. The revised manuscript still does not provide sufficient detail on how multiplex immunofluorescence labeling results were quantified. In particular, the terms MFI and HPF are not explained in sufficient detail, limiting reproducibility. As it stands, the manuscript does not describe the size of the analyzed tissue regions or the methods used to quantify labeling intensity.

Response: As already specified in our manuscript, monocytes and hepatocytes were clearly distinguished using classical lineage-specific markers: Ly6C (mouse) / CD14 (human) for monocytes and HNF4A for hepatocytes. In the revised text, we have further detailed that Mean Fluorescence Intensity (MFI) was quantified at single-cell resolution, representing the average pixel grayscale value extracted strictly within automated single-cell segmentation masks generated via DAPI and specific markers. Each High-Power Field (HPF) corresponds to a standardized 40× objective field measuring exactly 310 μm × 310 μm. For each liver section, 5 independent, non-overlapping HPFs at the tumor–liver interface were batch-processed to determine the average values for GS and XBP1s expression densities, as well as the absolute monocyte counts. Data integrity was strictly maintained across all cohorts using uniform exposure settings, identical laser acquisition parameters, and locked batch-processing scripts.

The authors commented that zone 3 GS expression was excluded from quantification; however, Extended Data Fig. 7b appears to show that liver zonation is lost in peritumoral liver. Thus, exclusion of GS-positive areas based on GS labeling intensity is no longer a valid approach in the tumor microenvironment. The authors are encouraged to provide a more detailed justification for this exclusion than is currently presented.

Response: As indicated in **Extended Data Fig. 7b**, both zone 1 and zone 3 regions remain clearly identifiable, and the canonical pericentral GS^+ hepatocyte band is preserved. In addition, metastatic remodeling induces ectopic GS expression in non-zone 3 hepatocytes, which is the biologically relevant population for our mechanistic analysis.

Our exclusion of zone 3 GS was therefore based on anatomical localization rather than GS intensity. For all quantification, central veins were first identified morphologically, and a fixed 2–3 hepatocyte-layer

pericentral region was excluded prior to GS measurement. This approach remains valid even when GS intensity increases in peri-tumoral hepatocytes, because the exclusion is grounded in vascular anatomy, not zonation-dependent intensity patterns.

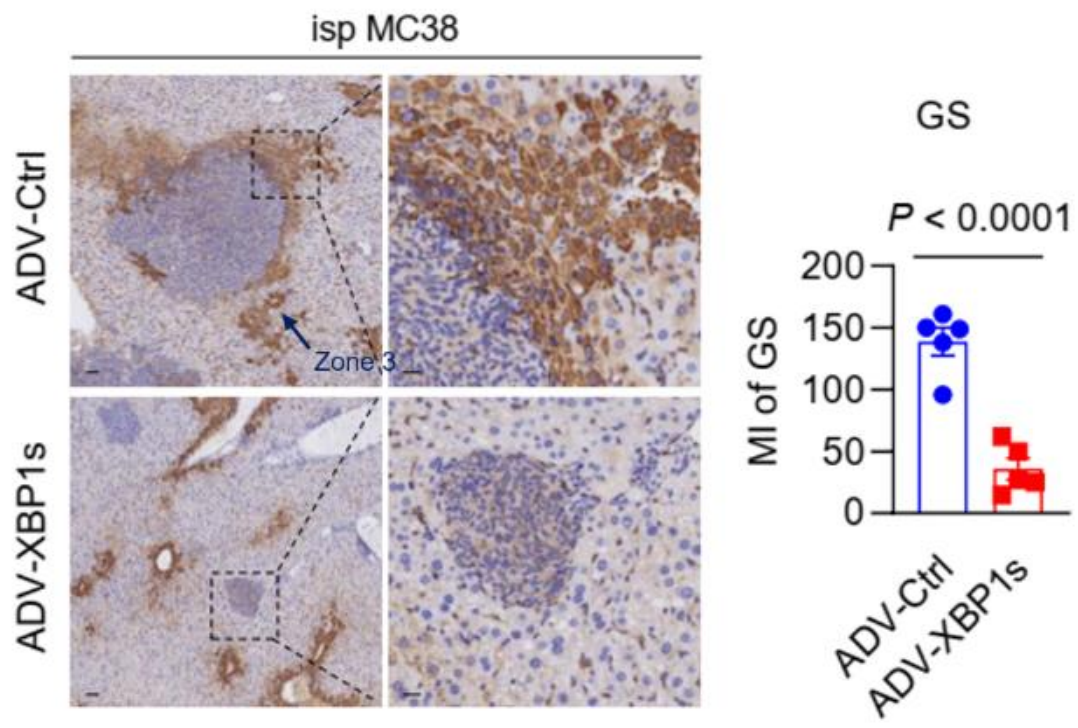

We suggested providing evidence for tumor cell presence and fractions within the images shown in Fig. 6a, and noted that the areas shown were not representative of the quantification data presented in the adjacent graph. In response, the authors replaced the representative image in the Ern1fl/fl panel. To interpret the new data, the manuscript would benefit from tumor cell–specific immunolabeling on a consecutive tissue section to unambiguously confirm tumor cell identity in the images shown. In addition, the method used to quantify the GS labeling signal in the accompanying graph is not described in sufficient detail. In particular, it remains unclear how the MI is defined (see comment above), and which image areas and of what size were included in the quantification.

Response: The regions shown in the upper panel of Fig. 6a correspond to day 7 peritumoral areas in *Ern1*$^{fl/fl}$ livers, where metastatic lesions are still small. At this early stage, tumor morphology differs from the fully developed lesions shown in later figures, which may explain the reviewer's impression.

The reviewer may suggest us confirm tumor cell presence by cytokeratin immunolabeling on a consecutive section. However, we consider that cytokeratin is not a tumor-specific marker in the liver, as it is expressed by biliary epithelial cells and can be upregulated in reactive hepatocytes. Instead, tumor cells were identified on the adjacent H&E section using pathological criteria, which remain the gold standard for tumor identification in both research and clinical practice. Consistent with prior studies using MC38 intrasplenic delivery, small metastatic nodules that we observed on day 7 subsequently expand into larger lesions, matching the expected temporal progression of this model.

As suggested, we have expanded the Methods section to describe the GS quantification workflow in full detail, including: definition of the quantified region (40× HPF), number of fields analyzed (5 per liver),

extraction of GS mean intensity (MI) using uniform thresholds, exclusion of the anatomical zone 3 pericentral band prior to measurement. We hope this clarification resolves the reviewer's concern.

We suggested clarifying in the Methods section which liver regions were used for single-cell RNA sequencing and flow cytometry. The authors answered this question in the rebuttal but did not add this information to the current version of the manuscript.

Response: We agree that the clarification provided in our rebuttal should also appear in the manuscript. We have now updated the Methods section to explicitly state that whole-liver tissues were used for single-cell RNA sequencing and flow cytometry.

We commented that in Fig. 7b, XBP1s expression is not visible in the representative multiplex immunofluorescence images chosen from ADV-Ctrl samples, whereas it is visible by immunohistochemistry in Fig. 1h. The authors responded that this reflects methodological differences between multiplex immunofluorescence and immunohistochemistry, with the latter being more sensitive to low signal intensities. However, low signal intensities can also be corrected for in immunofluorescence-based techniques. As such, it remains a concern how consistent and spatially homogeneous XBP1 protein levels actually are.

Response: XBP1s staining reflects intrinsic methodological differences rather than biological inconsistency. IHC uses enzymatic amplification, which substantially increases sensitivity for low-abundance molecules such as XBP1s. In contrast, mIF relies on direct fluorophore detection, which does not amplify weak signals and therefore produces lower apparent intensity. XBP1s expression in hepatocytes is spatially heterogeneous and context-dependent, with a slightly higher level in peritumoral regions and minimal expression in tumor-distant liver. The pattern observed in mIF is fully consistent with this biology and matches the distribution seen in our IHC dataset.

To avoid low-signal correction, all mIF images were acquired using identical exposure settings, and segmentation thresholds were batch-processed to ensure reproducibility. Regions with low XBP1s signal were not excluded but incorporated into the quantification pipeline, which reports mean intensity (MI) across all segmented hepatocytes.

We commented that the in situ data generally show small tissue regions with limited quantification, and in the same comment addressed the choice of Ly6C and CD14 as the sole monocyte phenotype markers. The authors responded by citing literature establishing CCR2 as a well-established inflammatory monocyte marker, and referred to Fig. 3, where co-immunolabeling for CCR2 and Ly6C

is shown. However, this does not address the general concern raised in our comment. Fig. 3f, in particular, exemplifies the generally very small regions shown for in situ labeling throughout the manuscript; these small representative regions do not capture intra- and intertumoral heterogeneity. This comment was of a general nature and applies not only to Fig. 3f but also to Fig. 6d, 7a, Extended Data Fig. 2a–c, and Extended Data Fig. 8a, g, and h. Beyond showing greater heterogeneity in the in situ data, selecting larger or multiple representative regions per figure requires adding a full description of the methods used to summarize the data to the manuscript. We suggest including annotation procedures, how mean intensity was summarized per sample/region, and a transparent account of imaging field size and cell numbers included in the analysis. In this context, the Methods section also lacks a methodological definition of positive cell populations, including thresholding as shown in Fig. 7c, Fig. 3f, and Extended Data Fig. 8c–f.

Response: We thank the reviewer for reiterating this point. We note that the definitions of MI and HPF are standard imaging metrics and were already clarified in our earlier responses. To ensure full transparency, we have now consolidated these definitions and added a brief, unified description to the Methods section. This includes: (i) the HPF size used for all quantification and (ii) how MI values were extracted from segmented cells. Cell lineage identification was determined by both fluorescence signals and distinct cell morphology. GS-positive cells were unambiguously identified based on their localized green fluorescence signals. For XBP1s expression analysis, we did not apply a strict binary definition of positive versus negative cell populations; instead, CRLM patients were stratified into XBP1s-high and XBP1s-low expression cohorts based on the median value of its MFI.

We commented that the term "metainflammation" is used without sufficient introduction. The authors replied that they replaced the term with "local metainflammation." However, this still does not sufficiently introduce the term. It remains unclear whether the data on monocyte infiltration, without accompanying cytokine profiling or functional assessment of the cells support the use of the term in the way it is done in the current version of the manuscript.

Response: We would like to clarify that our use of the term *local metainflammation* is descriptive rather than conceptual. Throughout the manuscript, we show that glutamine abundance in the liver metastatic niche is necessary and sufficient for inflammatory monocyte fitness. We believe the term does not require a formal terminological introduction or additional cytokine assays beyond the cellular and spatial data already presented, as the underlying mechanism is detailed in the manuscript.

Reviewer #5 (Remarks to the Author):

We appreciate the authors' clarification of several aspects of the methodology. However, important details regarding the annotation strategy and region selection remain insufficiently described. We believe that we can assess tumor morphology irrespective of tumor size. Nevertheless, in the representative images provided, it is not always possible to unambiguously distinguish tumor cells from other cell populations, such as inflammatory infiltrates. Cytokeratins, although acknowledged by the authors as not entirely specific, are routinely used in human pathology to distinguish epithelial tumor cells from epithelial non-tumor cells. Moreover, cytokeratin immunolabeling profiles continue to play an important role in the classification and diagnosis of tumor subtypes.

Therefore, some of our concerns remain unresolved, and we leave it to the Editors to determine whether the current level of methodological clarification is sufficient.

Reviewer #6 (Remarks to the Author):

I co-reviewed this manuscript with one of the reviewers who provided the listed reports. This is part of the Nature Communications initiative to facilitate training in peer review and to provide appropriate recognition for Early Career Researchers who co-review manuscripts.

Response: We appreciate the reviewer's continued consideration of this point. We agree that cytokeratin staining is widely used in pathology to identify epithelial tumor cells and to assist in tumor classification. Our intention was not to challenge its utility, but rather to explain its limited specificity in the context of the present liver metastasis model, where cytokeratin immunoreactivity was also detected in non-tumor hepatic structures. We therefore exercised caution in using cytokeratin staining as the sole basis for quantitative tumor-cell identification.

Importantly, the conclusions of this study do not rely on morphological annotation of representative images alone. Quantification of metastatic burden was performed using histologically defined tumor areas on H&E-stained sections, together with measurements of metastatic foci and liver tumor burden across multiple independent experiments. Furthermore, the key mechanistic conclusions are supported by complementary flow cytometric, transcriptomic, metabolomic, genetic, pharmacological, and adoptive-transfer studies.

In our 2nd revision, we have further clarified the criteria used for region selection, annotation, and histological assessment in the Methods section, with a concise version previously available in figure legends. We hope these clarifications will assist readers in interpreting the representative images appropriately.
